# Single-Flask Enantioselective Synthesis of α-Amino Acid Esters by Organocatalysis

**DOI:** 10.1021/acs.orglett.3c01736

**Published:** 2023-06-29

**Authors:** Vincenzo Battaglia, Sara Meninno, Andrea Pellegrini, Andrea Mazzanti, Alessandra Lattanzi

**Affiliations:** † Dipartimento di Chimica e Biologia “A. Zambelli”, Università di Salerno, Via Giovanni Paolo II 132, 84084, Fisciano, Italy; ‡ Dipartimento di Chimica Industriale “Toso Montanari”, Università di Bologna, Viale del Risorgimento 4, 40136, Bologna, Italy

## Abstract

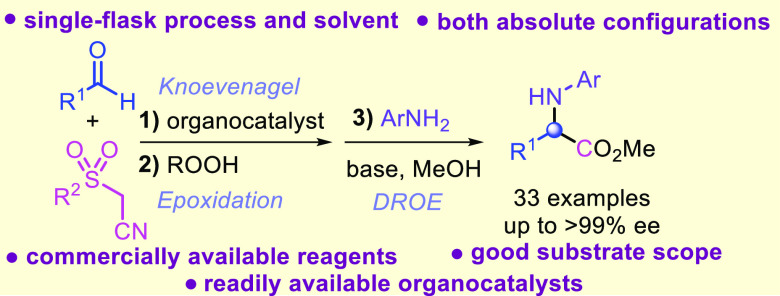

An operationally simple Knoevenagel condensation/asymmetric epoxidation/domino ring-opening esterification (DROE) approach has been disclosed to successfully access a good variety of (*R*)- and (*S*)-α-arylglycine esters from commercially available aldehydes, phenylsulfonyl acetonitrile, cumyl hydroperoxide, anilines, and readily available *Cinchona* alkaloid-based catalysts using a single solvent and reaction vessel. DFT calculations performed on the key asymmetric epoxidation showed the importance of cooperative H-bonding interactions in affecting the stereocontrol.

Among the optically active compounds, α-amino acids occupy a prominent role in life sciences by being the constituents of proteins and small peptides. Non-natural amino acids and, in particular, α-aryl glycines have been found in an increasing number of bioactive compounds, such as antibiotics and popular drugs, being able to modulate their properties and activity (Figure 1).^1^ Moreover, from a synthetic point of view, they are valuable building blocks and basic precursors of β-amino alcohols, which represent important ligands, catalysts and intermediates.^2^

**Figure 1:**
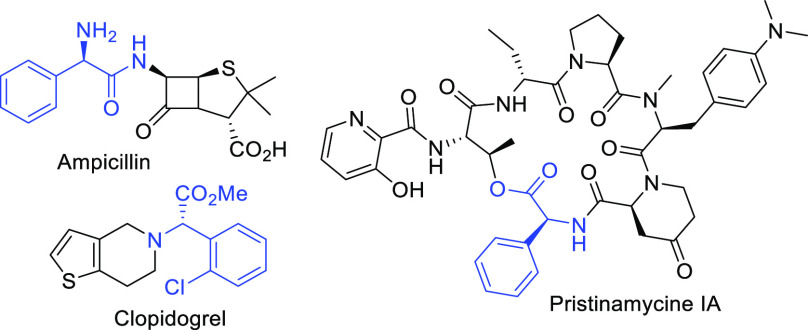
Representative bioactive compounds containing the α-arylglycine motif.

Because they are an important class of compounds, several synthetic methods have been developed over the last decades to access chiral nonracemic α-aryl glycine esters.^3^ Besides the classical asymmetric Strecker synthesis,^4^ more recently, catalytic metal–chiral ligand hydrogenation or organocatalyzed reduction of α-iminoesters have been the most investigated processes, which have provided excellent results in terms of efficiency and enantioselectivity (Scheme 1a).^5^ The catalytic metal-based multicomponent Petasis reaction, which combines easy-to-access reagents, provides α-arylglycines bearing electron-rich aromatic moieties with high enantioselectivity (Scheme 1b).^6^ A similar catalytic system assures the formation of the products with moderate to high enantiocontrol starting from *N*-arylglycine esters (Scheme 1c).^7^ Metal/chiral ligands or visible-light-induced/organocatalyst-based systems have proved to be effective combinations for a highly enantioselective N–H insertion of α-diazo α-arylacetates with different nitrogen compounds (Scheme 1d).^8^ Successful results have also been achieved when using carbonyl sulfoxonium ylides as the reagents (Scheme 1d).^9^

**Scheme 1:**
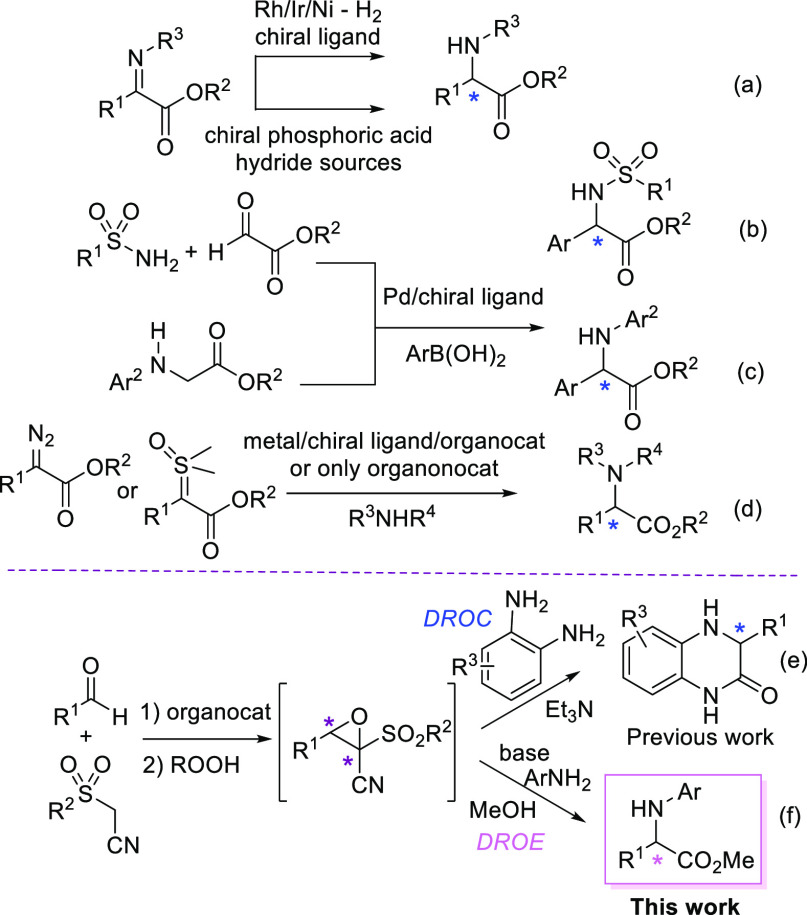
Asymmetric Routes to α-Arylglycines

Despite the significant number of asymmetric methodologies used to prepare this class of compounds, alternative catalytic processes that can be practical and efficient by using commercially available reagents in a sustainable reaction setup remains to be developed. We recently disclosed a one-pot catalytic asymmetric strategy to dihydroquinoxalinones, which involved the formation of new 1-phenylsulfonyl-1-cyano epoxides as key intermediates.^10^ They mimic α-halo acyl halide synthons^11^ and are able to undergo a domino ring-opening cyclization (DROC) to the heterocycles, thereby maintaining the level of enantioselectivity (Scheme 1e). Commercial reagents, readily available and recyclable quinine-derived urea catalyst, and a sole solvent have been used in the process. Given the practical and attractive features of the Knoevenagel condensation/asymmetric epoxidation/DROC strategy, we questioned whether this approach could meet the challenge of preparing optically active α-arylglycine esters via a domino ring-opening esterification (DROE) step (Scheme 1f).

The main issues faced when moving to a DROE sequence are in terms of (i) product selectivity because of the lack of the beneficial intramolecular nature of the bidentate nucleophile and (ii) stereointegrity because of the propensity of α-arylglycines to suffer base-catalyzed racemization.^12^ Herein, we illustrate the successful development of a one-pot catalytic enantioselective synthesis of α-arylglycine esters in both absolute configurations from commercial reagents and the applicability of the process to prepare unnatural α-alkyl amino acid esters.

We reasoned that when using anilines and methanol as the nucleophiles, the selective formation of α-arylglycine ester versus amide could be controlled by managing the reaction conditions. A preliminary study of the DROE step using racemic 3-(4-cyanophenyl)-2-(phenylsulfonyl)oxirane-2-carbonitrile **1a** and *p*-anisidine in MeOH enabled the assessment of the reagents ratio that is useful to avoid side-product formation (see the Supporting Information). The choice of the *p*-CN electron-withdrawing group in the phenyl ring of **1a** and basic *p*-anisidine would have helped to better tune the conditions to avoid racemization in the asymmetric epoxidation/DROE sequence. Accordingly, *epi*-quinine-derived urea (**eQNU**) was used under previously optimized conditions with cumyl hydroperoxide (CHP).^10^ A panel of bases was checked, with *p*-anisidine and MeOH added in the optimal ratio found in the preliminary study (Table 1). When using Et_3_N, the product was obtained in good yield and with 80% ee (entry 1). More basic and sterically demanding 1,8-bis(dimethylamino)naphthalene (proton sponge) negatively affected the conversion, and a significant degree of racemization was observed (entry 2). Surprisingly, a similar result was achieved with the addition of poorly basic 2,6-lutidine (entry 3).^12a^ Pleasingly, when diisopropyl ethyl amine (DIPEA) was used, the product was recovered in 53% yield and 91% ee (entry 4), which could be improved to 79% yield and 89% ee, although after a prolonged reaction time (entry 5). These results prompted us to check sterically hindered dicyclohexyl methyl amine [(Cy)_2_NMe], which afforded the product in 80% yield and 90% ee (entry 6), thereby attesting that epimerization had been essentially avoided.

**Table 1:**
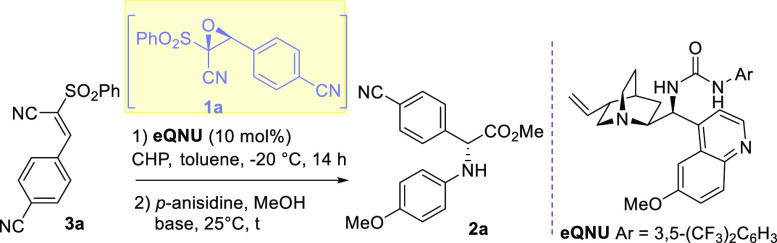
Optimization of the One-Pot Enantioselective Epoxidation/DROE Process on Alkene **3a**^a^

entry	base	*t* (h)	yield (%)^b^	ee (%)^c^
1	Et_3_N	7.5	72	80
2	proton sponge	11	33	68
3	2,6-lutidine	7.5	48	72
4	DIPEA	7.5	53	91
5	DIPEA	31	79	89
6	(Cy)_2_NMe	7.5	80	90

a Reaction conditions: step (1) alkene **3a** (0.1 mmol), **eQNU** (0.01 mmol), CHP (0.11 mmol) in anhydrous toluene (5 mL); step (2) *p*-anisidine (0.11 mmol), base (0.2 mmol), MeOH (100 equiv).

b Yield determined by ^1^H NMR analysis of crude reaction mixture using 1,3,5-(MeO)_3_C_6_H_3_ as standard.

c HPLC analysis on a chiral stationary phase.

With the optimized conditions in hand, the one-pot asymmetric synthesis of α-arylglycine methyl esters from aldehydes, (phenylsulfonyl)acetonitrile, CHP, and aniline was investigated (Scheme 2).

**Scheme 2:**
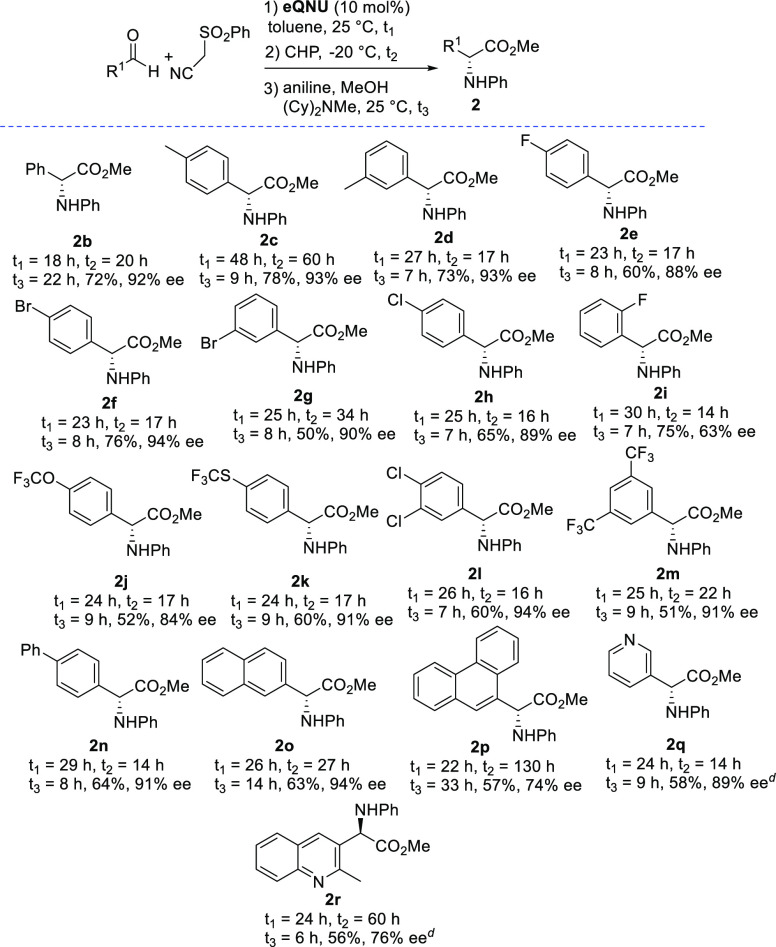
One-Pot Enantioselective Catalytic Synthesis of α-Arylglycines Using Aniline^-^ Reaction conditions: (I) Knoevenagel step of (phenylsulfonyl)acetonitrile (0.1 mmol), aldehyde (0.1 mmol), and **eQNU** (0.01 mmol) in anhydrous toluene (*C* = 0.3 M); (II) Epoxidation step of dilution of the reaction with toluene (*C* = 0.02 M) at −20 °C, then addition of CHP (0.11 mmol); and (III) DROE step of addition of aniline (0.12 mmol), (Cy)_2_NMe (0.15 mmol), and MeOH (100 equiv) at 25 °C. Yield of isolated product after chromatography. HPLC analysis on a chiral stationary phase. The DROE step was carried out at 0 °C.

α-Arylglycine esters **2b**–**i**, unsubstituted or bearing an electron-donating group and halogen atoms in the phenyl ring, were recovered in good to high overall yields and high enantioselectivity with the exception of the *ortho*-fluoro **2i** derivative, which showed 63% ee. Pleasingly, α-arylglycines bearing different electron-withdrawing groups **2j**–**m** could be satisfactorily isolated with a comparable level of enantioselectivity. Polycyclic aromatic residues at the α-position of the amino acid esters **2n**–**p** were also tolerated, even when the sterically demanding 9-phenathrenecarboxaldehyde was used as the reagent, and the product **2p** was obtained with 74% ee. Interestingly, challenging heteroaromatic 3-pyridine and 2-methylquinoline-based α-amino acid esters **2q** and **2r** were isolated in good yields, 89% and 76% ee, respectively.^13^ The α-arylglycine methyl esters were confirmed to be (*R*)-configured by comparison with optical rotations in the literature.

The suitability of substituted anilines as reagents was next investigated (Scheme 3).

**Scheme 3:**
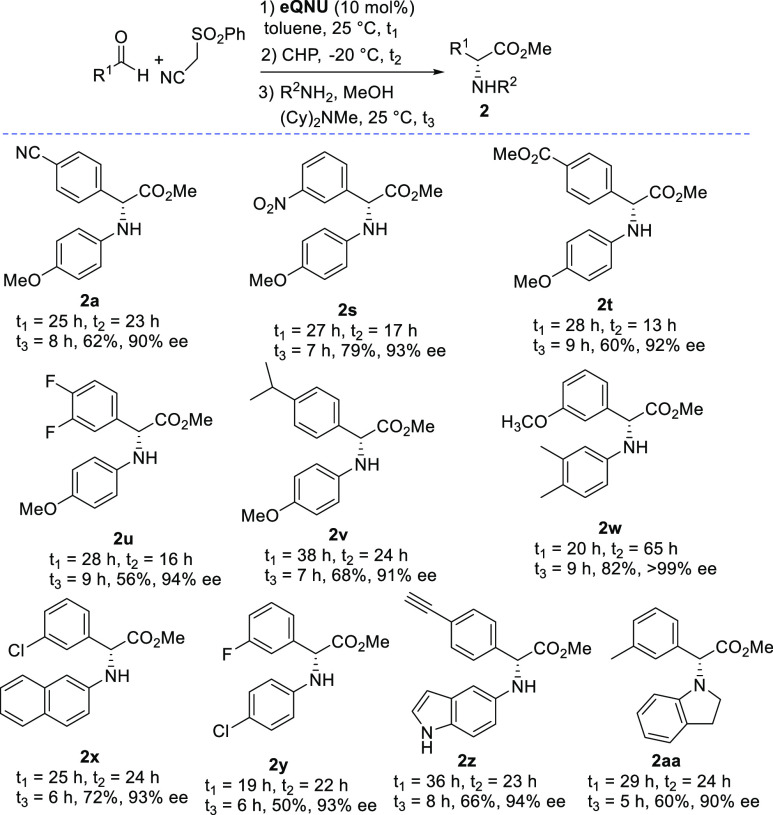
Scope of the Enantioselective Synthesis of α-Arylglycines Using Anilines

Delightfully, anisidine and the electron-rich 3,4-dimethyl aniline when used with aldehydes bearing strong or moderate electron-withdrawing and -donating groups provided the final α-amino acid esters **2a** and **2s**–**w** with ee values higher than 90%, up to >99%. These results confirmed the reliability of the mild reaction conditions adopted to avoid epimerization. 2-Naphthyl or less reactive *para*-chloro anilines proved to be suitable reagents, thereby leading to products **2x** and **2y** with 93% ee. Interestingly, α-arylglycine **2z**, which is of potential use in “click reactions”, and **2aa** were isolated in good overall yield and high ee values when employing heterocyclic-based 5-aminoindole or the secondary amine indoline. The feasibility of the one-pot procedure for the synthesis of unnatural α-alkyl amino acid esters was then examined from alkenes **3** (Scheme 4).

**Scheme 4:**
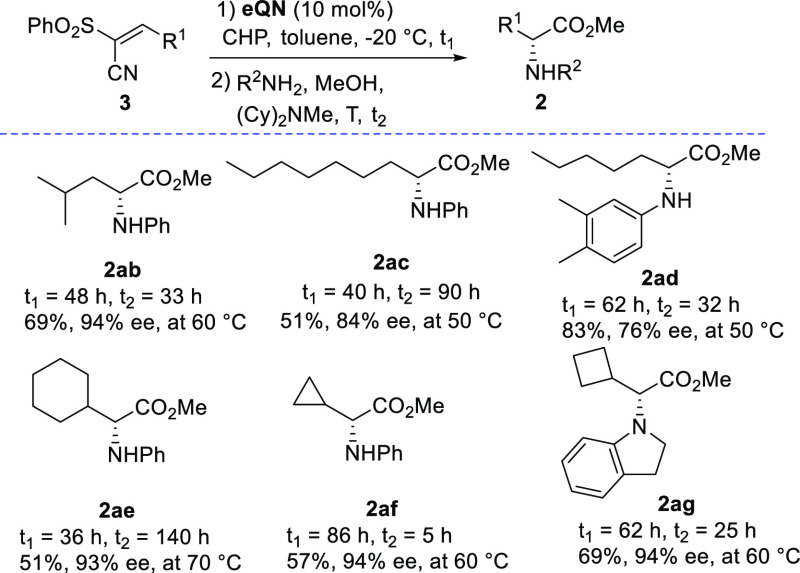
One-Pot Enantioselective Catalytic Synthesis to Unnatural α-Alkyl Amino Acid Esters from Alkenes Reaction conditions as reported in Scheme 2 starting from 0.1 mmol of the alkene **3**.

Given the higher temperatures required for ring opening of the epoxides,^10^ epimerization would have been more problematic to control. Initially, leucine methyl ester (*R*)-**2ab** was prepared in 69% yield and 94% ee by carrying out the DROE step at 60 °C.^14^ When we turned to unnatural derivatives bearing linear alkyl chains, satisfactory conversions and 84% and 76% ee for **2ac** and **2ad**, were achieved, respectively.^15^ These results could be ascribed to a faster racemization rate of less sterically demanding α-amino acid esters **2ac** and **2ad**. To further corroborate this hypothesis, alkenes bearing bulkier cyclic moieties were subjected to the usual conditions. Consistent with this hypothesis, the corresponding products **2ae**, **2af**, and **2ag** were recovered with excellent ee values. This protocol enables a facile asymmetric synthesis of challenging α-cycloalkyl amino acid esters,^16^ which are building blocks of interest in peptide and medicinal chemistry. With a view to developing a general protocol to afford both enantioenriched products, a study of the asymmetric epoxidation was undertaken (see the Supporting Information). A quinidine-derived catalyst **4**(17) bearing a (*R*,*R*)-diamine fragment and a NH_2_ group provided some representative (*S*)-α-amino acid esters in one-pot approaches either from 1-naphthylsulfonylacetonitrile and the aldehyde or the alkene as the reagents (Scheme 5).

**Scheme 5:**
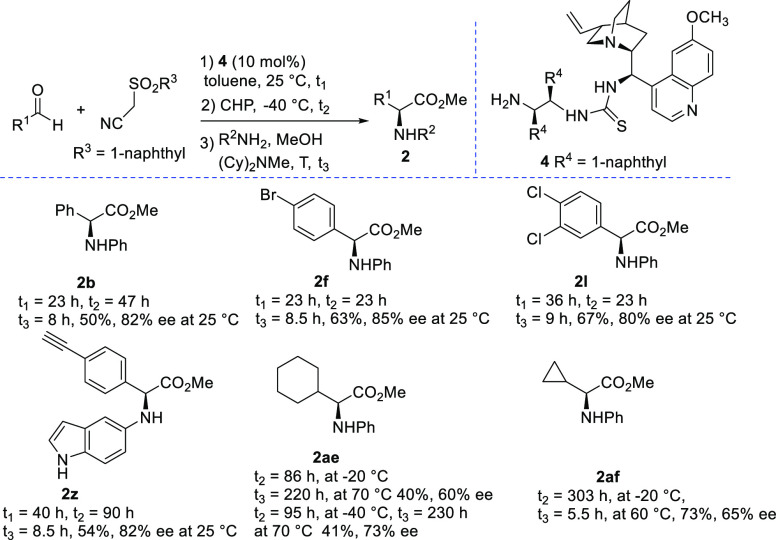
One-Pot Enantioselective Catalytic Synthesis of α-Amino Acid Esters with Catalyst **4**

α-Arylglycine esters **2b**, **2f**, **2l**, and **2z** were isolated in good yield and ee values ranging from 80% to 85% by working at −40 °C in the epoxidation step. Sterically hindered α-cycloalkyl-substituted amino acid esters **2ae** and **2af** were recovered in satisfactory overall yield with 60% and 65% ee by starting from the corresponding alkenes **3** and working at −20 °C in the epoxidation step. The enantioselectivity can be improved by carrying out the epoxidation at −40 °C, as exemplified by compound **2ae** isolated with 73% ee. To demonstrate the synthetic utility of the one-pot process, 1 mmol-scale preparation of compound **2f** was carried out under standard conditions (Scheme 6). Gratifyingly, the product was isolated with the same yield and enantioselectivity, as observed in Scheme 2. Next, α-aryl glycine esters were elaborated under reductive and oxidative conditions. Reduction of enantioenriched α-amino acid esters **2d**, **2f**, and **2af** with diisobutylaluminium hydride (DIBALH) smoothly afforded the aromatic and cyclopropyl-substituted β-amino alcohols **5a**–**c** in good yields and without erosion of the enantioselectivity.

**Scheme 6:**
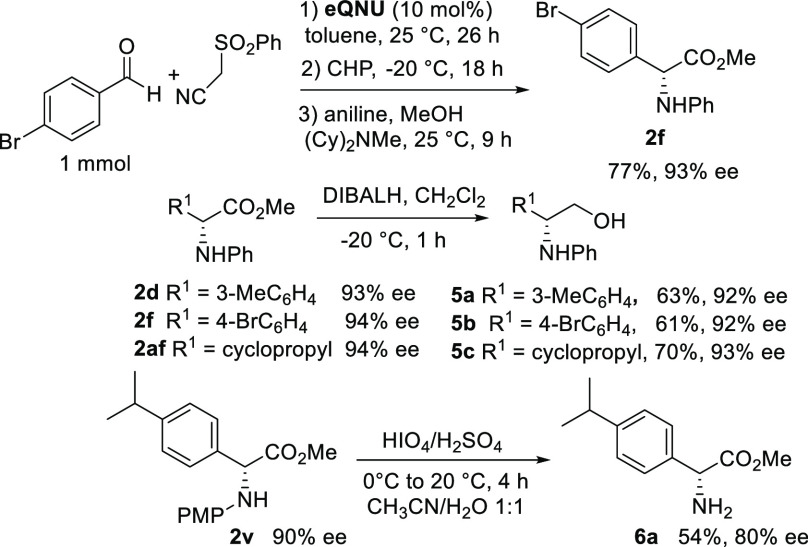
Scale-up and Synthetic Elaborations of α-Amino Acid Esters

The PMP deprotection of enantioenriched compound **2v** performed with CAN^5a,5b^^f^ disappointingly led to NH_2_-free **6a** in modest yield and marked erosion of the ee value. However, in agreement with Smith et al.’s results,^18^ arylglycine ester **6a** was more efficiently obtained in 54% yield and 80% ee when using periodic acid as the oxidant.

Catalyst **4** bears a bulky 1,2-bis(1-naphthyl) moiety with a free NH_2_ group. It is, therefore, foreseeable that the active conformation of the catalyst, the hydrogen bond network, and the geometries of transition states could be quite different from those already identified for the **eQNU** catalyst.^10^ DFT calculations (see Supporting Information) showed that in both transition states (TSs) for *tert*-butyl hydroperoxide (TBHP) addition to model alkene **3b′** (TS1-R and TS1-S in Figure 2, top), the free NH_2_ is able to engage an additional hydrogen bond with the oxygens of sulfone. The TS1-R geometry is lower in energy by 2.95 kcal/mol with respect to TS1-S, which is in agreement with the final experimental outcome after the DROE process. The intramolecular S_N_2 process, in the ring closure step to epoxide, occurs with higher energy with respect to the oxa-Michael reaction.^10^ In the two TSs yielding the *S*,*S*- and *R,R*-epoxides, the TS2-RR geometry turns out again to be 2.41 kcal/mol lower in energy with respect to the TS2-SS (Figure 2, bottom).^19^

**Figure 2:**
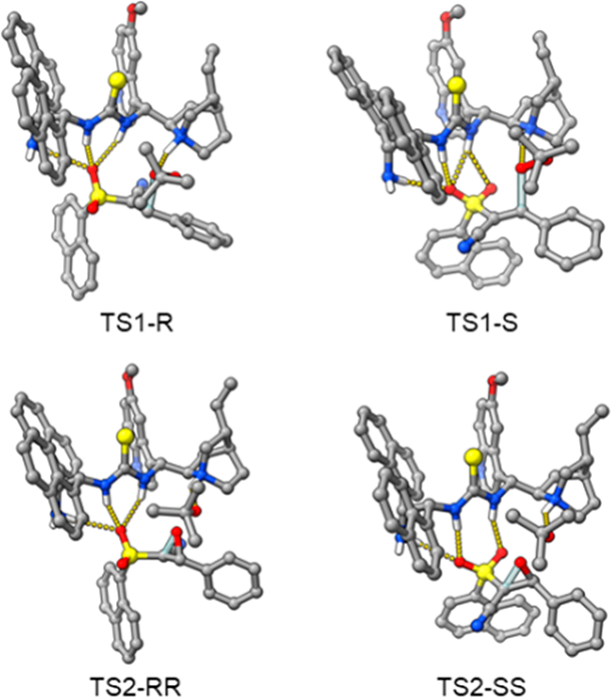
3D representations of the TS geometries for the addition and ring-closure steps of alkene **3b′** (R^1^ = Ph, R^3^ = 1-naphthyl) and TBHP with catalyst **4**. Dotted lines indicate hydrogen bonds.

In conclusion, we developed a catalytic asymmetric single-pot sequence to conveniently prepare α-arylglycine esters of both absolute configurations in good to high yields and enantioselectivity.

Notable features of the process are the employment of readily available organocatalysts and access to a great pool of commercially available aldehydes and anilines, as well as CHP and phenylsulfonylacetonitrile as the reagents. Moreover, the use of a sole organocatalyst, solvent, and a single purification at the end of three steps are required. The approach can be applied for the asymmetric synthesis of unnatural α-alkyl amino acid esters starting from the alkenes. DFT calculations showed the importance of the free NH_2_ group in catalyst **4**, which is useful to reinforce the hydrogen bond network in the TSs of the epoxidation.

## Data Availability

The data underlying this study are available in the published article and its Supporting Information.

## Supporting Information Available

Full experimental procedures, characterization data, NMR spectra, HPLC traces, and computational details (PDF)
