# Supplementary material for: Single-Flask Enantioselective Synthesis of α-Amino Acid Esters by Organocatalysis
Source: Org Lett. 2023 Jun 29;25(27):5038–43. doi: 10.1021/acs.orglett.3c01736 (PMC10353036; doi:10.1021/acs.orglett.3c01736)
Supplement: Supplementary file 1 — ol3c01736_si_001.pdf [file ol3c01736_si_001.pdf]

Supporting Information

Single-Flask Enantioselective Synthesis of α-Amino Acid Esters by Organocatalysis

Vincenzo Battaglia,‡ Sara Meninno,‡ Andrea Pellegrini,† Andrea Mazzanti,† and Alessandra Lattanzi*‡

‡*Dipartimento di Chimica e Biologia "A. Zambelli", Università di Salerno, Via Giovanni Paolo II, I-84084, Fisciano, Italy*
†*Dipartimento di Chimica Industriale, Università di Bologna, Viale Risorgimento 4 – 40136, Bologna, Italy*

*E-mail: lattanzi@unisa.it

General Methods S2

Experimental Procedures and Compounds Characterization S3

General procedure for the synthesis of racemic epoxides 1 from alkenes 3 .... S3
Table S1. Screening of DROE process on epoxide **1a** S3
General procedure for the asymmetric synthesis of epoxides 1b, 1b', 1b'' from alkenes 3b, 3b', 3b'' . S4
Table S2. Screening of the reaction conditions for the asymmetric epoxidation of alkenes **3** S5
General procedure for the racemic synthesis of arylglycines 2a-z, 2aa and 2ab-ag S6
General procedure for one-pot asymmetric Knoevenagel/epoxidation/ring-opening/esterification reaction and epoxidation/ring-opening/esterification reaction to arylglycines 2a-2z, 2aa and 2ab-2ag S6
General procedure for one-pot asymmetric Knoevenagel/epoxidation/ring-opening/esterification reaction and epoxidation/ring-opening/esterification reaction to arylglycines (*S*)-2b, (*S*)-2f, (*S*)-2l, (*S*)-2z, (*S*)-2ae, (*S*)-2af S20
General procedure for the synthesis of alkenes 3b', 3b'' S22
General procedure for the synthesis of alkenes 3af, 3ag, 3ae', 3af' S23
General procedure for the synthesis of aminoalcohols 5a-c from arylglycines 2 .... S25
Procedure for the deprotection of arylglycine 2v to product 6a S26
Scale-up of the one-pot asymmetric Knoevenagel/epoxidation/ring-opening/esterification reaction to arylglycine 2f S27
NMR Spectra S28

HPLC Chromatograms S122

DFT Calculations S167

Computational methods S167
Results S167
Geometries S172

General Methods

All reactions requiring dry or inert conditions were conducted in flame-dried glassware under a positive pressure of nitrogen. Anhydrous toluene was purchased from Aldrich and used as received, all other solvents were dried over molecular sieves. Molecular sieves (Aldrich Molecular Sieves, 3 Å, 1.6 mm pellets) were activated under vacuum at 200 °C overnight. Reactions were monitored by thin layer chromatography (TLC) on Macherey-Nagel pre-coated silica gel plates (0.25 mm) and visualized by UV light. Flash chromatography was performed on Merck silica gel (60, particle size: 0.040–0.063 mm). Preparative chromatography was performed on Macherey-Nagel pre-coated silica gel plates (2 mm). ^{1}H NMR and ^{13}C NMR spectra were recorded on Bruker Avance III HD 600, Bruker Avance-400, Bruker Avance-300 in $CDCl_3$. Chemical shifts for protons are reported using residual solvent protons (δ = 7.26 ppm for $CDCl_3$) as internal standard. Carbon spectra were referenced to the shift of the ^{13}C signal of $CDCl_3$ (δ = 77.0 ppm).

The following abbreviations are used to indicate the multiplicity in NMR spectra: s - singlet; d - doublet; t - triplet; q - quartet; dd – double doublet; ddd – doublet of doublet of doublets; m - multiplet; bs - broad signal. Optical rotation of compounds was performed on a Jasco P-2000 digital polarimeter using WI (Tungsten-Halogen) lamp (λ = 589 nm). High resolution mass spectra (HRMS) were acquired using a Bruker solariX XR Fourier transform ion cyclotron resonance mass spectrometer (Bruker Daltonik GmbH, Bremen, Germany) equipped with a 7T refrigerated actively-shielded superconducting magnet. The samples were ionized in positive ion mode using a MALDI or ESI ionization sources. Melting points were measured with a Stuart Model SMP 30 melting point apparatus and are uncorrected. Petrol ether (PE) refers to light petroleum ether (boiling point 40-60 °C). All starting materials (unless otherwise noted) were purchased from Merck-SigmaAldrich or TCI-Europe and used as received. Alkenes **3** are known compounds, they were prepared according to the literature. [1] Aniline, *p*-anisidine, 3,4-dimethylaniline, indoline, 5-aminoindole and 4-chloroaniline were purchased from TCI and used as received. 2-(naphthalene-2-ylsulfonyl)acetonitrile and 2-(naphthalene-1-ylsulfonyl)acetonitrile are known compound, they were prepared according to the literature.[2] (Phenylsulfonyl)acetonitrile was purchased from Merck-Sigma Aldrich and it was used as received. Cinchona alkaloids were purchased from Aldrich and used as received. Organocatalysts **eQNU**[3] and **4**[4] were prepared according to the literature.

[1] (a) Volpe, C.; Meninno, S.; Crescenzi, C.; Mancinelli, M.; Mazzanti, A.; Lattanzi, A. *Angew. Chem. Int. Ed.* **2021**, *60*, 23819; (b) Pandit, K. S.; Kupwade, R. V.; Chavan, P. V.; Desai, U. V.; Wadgaonkar, P. P.; Kodam, K. M. *ACS Sust. Chem. Eng.* **2016**, *4*, 3450; (c) Rajkumar, S.; Shankland, K.; Goodman, J. M.; Cobb, A. J. A. *Org. Lett.* **2013**, *15*, 1386; (d) Nemcsok, T.; Rapi, Z.; Bagi, P.; Guan, Y. H.; Orbán, I.; Keglevich, G.; Bakó, P. *Tetrahedron* **2020**, *76*, 130965.

[2] Diosdado, S.; López, R.; Palomo, C. *Chem. Eur. J.*, **2014**, *20*, 6526.

[3] (a) Miyaji, R.; Asano K.; Matsubara, S. *Org. Lett.* **2013**, *15*, 3658. (b) Amere, M.; Lasne, M.-C.; Rouden, J. *Org. Lett.* **2007**, *9*, 2621. (c) Wu, W.; Min, L.; Zhu, L.; Lee, C.-S. *Adv. Synth. Catal.* **2013**, *353*, 1135.

[4] Meninno, S.; Zullo, L.; Overgaard, J.; Lattanzi, A. *Adv. Synth. Catal.* **2017**, *359*, 913.

Enantiomeric excess of epoxides **1b'**, **1b''**, products **2**, **5**, **6a** was determined by HPLC (Waters-Breeze 2487, UV dual λ absorbance detector and 1525 Binary HPLC Pump) using Daicel chiral columns.

Experimental Procedures and Compounds Characterization

General procedure for the synthesis of racemic epoxides 1 from alkenes 3

In a sample vial containing the alkene (0.1 mmol, 1 eq.) and 2-piperidinemethanol (2.3 mg, 0.02 mmol, 0.2 eq.) in anhydrous toluene (1 mL, C = 0.1 M), TBHP (~5.5 M in decane, 23 μL, 0.12 mmol, 1.2 eq.) was added. The reaction was stirred at room temperature for 7-14 hours, monitored by TLC (eluent PE/ethyl acetate 80/20). The product was isolated by flash chromatography (eluting from PE/ethyl acetate 100/0 to 80/20).

3-(4-Cyanophenyl)-2-(phenylsulfonyl)oxirane-2-carbonitrile 1a

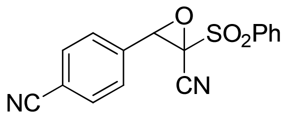

Yellow solid (flash chromatography eluent: hexanes/EtOAc, 100/0 to 80/20), 29.8 mg, 96% yield. **mp**: 137.8-138.5 .**^{1}H NMR** ($CDCl_3$, 300 MHz): *δ* 8.06 (d, 2H, J = 7.4 Hz), 7.87 (t, 1H, J = 7.5 Hz), 7.77-7.70 (m, 4H), 7.53 (d, 2H, J = 8.3 Hz), 5.09 (s, 1H). **^{13}C NMR** ($CDCl_3$, 75 MHz): *δ* 136.2, 133.7, 133.5, 132.7, 130.0, 129.9, 127.4, 117.7, 114.7, 110.4, 67.2, 62.4. **HRMS (ESI-FT ICR)** m/z: $[M+H]^+$ calculated for $C_{16}H_{11}N_2O_3S$: 311.0485, found: 311.0479.

Table S1. Screening of DROE process on epoxide **1a**[a]

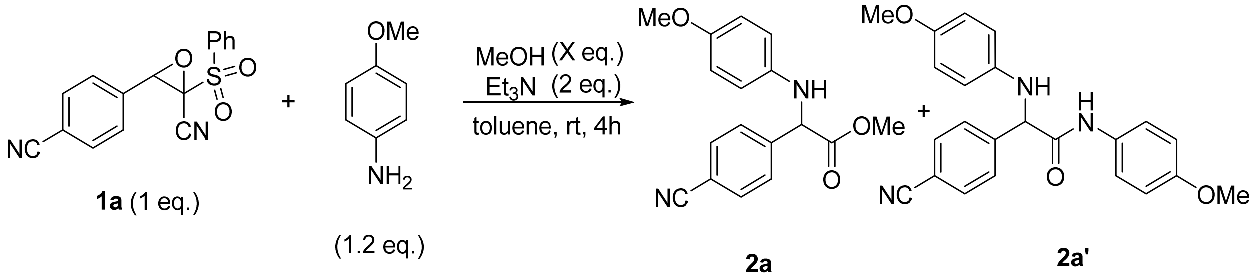

Entry	MeOH (eq.)	Yield **2a** [%][b]	Yield **2a'** [%][c]
1	30	57	15
2	60	61	11
3	100	68	7

[a] *Reaction conditions*: **1** (0.1 mmol, 1 eq.), anisidine (0.12 mmol, 1.2 eq.), triethylamine (0.02 mmol, 2 eq.) and methanol in anhydrous toluene (5 mL, C = 0.02 M). [b] Yield of isolated product. [c] Yields determined by ^{1}H NMR analysis on the crude mixture.

The ring-opening /esterification reaction of epoxide **1a** was carried in the presence of different amounts of methanol in toluene at C = 0.02 M at room temperature with *p*-anisidine (1.2 eq.) and

triethylamine (2 eq.), which are necessary to quench HCN and HSO_2Ph produced in the reaction. By increasing the amount of methanol in the reaction mixture, a reduced amount of amide by-product **2a'** was detected, hence 100 equivalents of methanol (entry 3) were chosen as the optimal value to carry out the ring-opening/esterification step in the one-pot asymmetric one-pot process to arylglycines **2**. Compound **2a'** was isolated in an experiment carried out using 20 eq. of MeOH in 20% yield.

A preliminar investigation on the applicability of other nitrogen sources in the DROE step has been carried out using benzyl amine and racemic epoxide **1b** under usual conditions. At the end of the process, ^{1}H-NMR analysis of the crude mixture showed the presence of the expected α-aryl-glycine methyl ester **7** in ~20% yield and of the corresponding amide **8** in a 1/1 ratio. This result is in line with higher nucleophilicity and reactivity of benzylamines with respect to anilines. Hence, further optimization of alkyl amine/MeOH ratio and reaction parameters will be necessary either to reduce the amount of amide side-product and control potential epimerization occurring on the final product **7**.

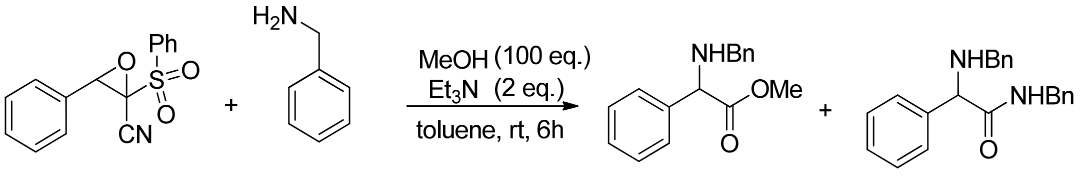

Reaction conditions: **1b** (28.5mg, 0.1 mmol, 1 eq.), benzylamine (0.12 mmol, 1.2 eq.), triethylamine (28 µL 0.02 mmol, 2 eq.) and methanol (405 µL, 10 mmol, 100 eq.) in anhydrous toluene (5 mL, C = 0.02 M). Yield and ratio determined by ^{1}H NMR analysis on the crude mixture.

General procedure for the asymmetric synthesis of epoxides 1b, 1b', 1b'' from alkenes 3b, 3b', 3b''

The optimization process has been carried out selecting catalyst **4**, which has been found the most effective for the synthesis of (*R*,*R*)-configured epoxides.[5] In a sample vial containing the proper alkene (0.1 mmol, 1 eq.) and catalyst **4** (7.8 mg, 0.01 mmol, 0.1 eq.) in anhydrous toluene (5 mL), cumene hydroperoxide (tech. 80%, 20 µL, 0.11 mmol, 1.1 eq.) was added at -20 °C or -40 °C and the solution was stirred for 15-64 hours, monitored by TLC (eluent PE/ethyl acetate 80/20). The product was isolated by flash chromatography (eluting from PE/ethyl acetate 100/0 to 90/10).

[5] Meninno, S.; Lattanzi, A. *J. Org. Chem.* **2023**, *88*, 7888.

Table S2. Screening of the reaction conditions for the asymmetric epoxidation of alkenes **3**[a]

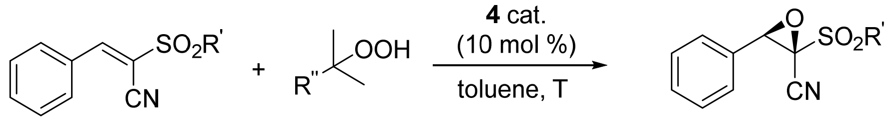

3 (1 eq.) (1.1 eq.) **1**

entry	R'	3	R''	T	*t* [h]	yield **1** [%][b]	ee **1** [%]
1	Ph	**3b**	Me	-20	16	97	60
2	Ph	**3b**	Ph	-20	64	>98	55
3	2-Naft	**3b''**	Me	-20	25	49[c]	60
4	2-Naft	**3b''**	Ph	-20	15	80	66
5	1-Naft	**3b'**	Me	-20	63	>98	72
6	1-Naft	**3b'**	Ph	-20	20	>98	72
7	1-Naft	**3b'**	Me	-40	24	43[c]	77
8	1-Naft	**3b'**	Ph	-40	23	>98	82
9	1-Naft	**3b'**	Ph	-55	41	72	80

[a] *Reaction conditions*: **3** (0.1 mmol, 1 eq.), TBHP or CHP (0.11 mmol, 1.1 eq.) and **4** (7.8 mg, 10 mol%, 0.01 mmol) in anhydrous toluene (5 mL, C = 0.02 M). [b] Yield of isolated product. [c] Yield determined by ^{1}H NMR analysis on the crude mixture.

(2*R*,3*R*)-2-(Naphthalen-1-ylsulfonyl)-3-phenyloxirane-2-carbonitrile 1b'

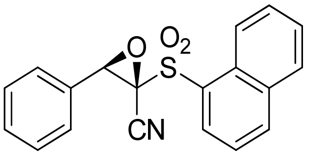

White wax (flash chromatography eluent: hexanes/EtOAc, 100/0 to 90/10), 31.5 mg, 94% yield. $[\alpha]_D^{20}$ = -80.1 (C = 0.60, $CHCl_3$), 82% ee. **^{1}H NMR** ($CDCl_3$, 300 MHz): δ 8.85 (d, 1H, J = 8.6 Hz), 8.49 (dd, 1H, J = 7.3, 1.0 Hz), 8.28 (d, 1H, J = 8.1 Hz), 8.03 (d, 1H, J = 8.1 Hz), 7.81-7.76 (m, 1H), 7.73-7.70 (m, 2H), 7.46-7.41 (m, 5H), 5.12 (s, 1H).**^{13}C NMR**: ($CDCl_3$, 150 MHz): δ 137.5. 134.4, 133.9, 130.8, 130.0, 129.5, 129.3, 129.1, 129.0, 128.4, 127.6, 126.7, 125.0, 124.5, 110.8, 68.4, 62.5. **HRMS (ESI-FT ICR)** m/z: $[M+Na]^+$ calculated for $C_{19}H_{13}NaNO_3S$: 358.0508, found: 358.0538. HPLC (OD-H, 2-propanol/n-hexane = 5/95, flow rate = 1.0 mL/min, l = 220 nm) t_R = 17.1 min (major), 18.4 min (minor).

(2*R*,3*R*)-2-(Naphthalen-2-ylsulfonyl)-3-phenyloxirane-2-carbonitrile 1b''

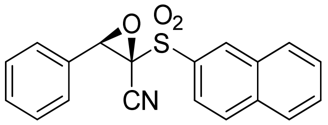

White solid (flash chromatography eluent: hexanes/EtOAc, 100/0 to 90/10), 26.8 mg, 80% yield. **mp:** 103.3-104.2. $[\alpha]_D^{21}$ = -2.5 (C = 0.60, $CHCl_3$), 66% ee. **^{1}H NMR** ($CDCl_3$, 600 MHz): δ 8.67 (s, 1H), 8.13 (d, 1H, J = 8.6 Hz), 8.10 (d, 1H, J = 8.2 Hz), 8.01 (d, 2H, J = 8.3 Hz), 7.78 (t, 1H, J = 7.6), 7.73-7.70 (m, 1H), 7.48-7.44 (m, 3H), 7.41 (d, 2H, J = 7.0 Hz), 5.10 (s, 1H). **^{13}C NMR**: ($CDCl_3$, 150 MHz): δ 136.3. 132.7, 132.2, 131.1, 130.8, 130.4, 130.2, 129.9, 129.0, 128.5, 128.2, 128.1, 126.7, 123.4, 111.1, 67.6, 63.7. **HRMS (ESI-FT ICR)** m/z: $[M+Na]^+$ calculated for $C_{19}H_{13}NaNO_3S$: 358.0508, found: 358.0539. HPLC (OD-H, 2-propanol/n-hexane = 5/95, flow rate = 1.0 mL/min, l = 220 nm) t_R = 15.1 min (major), 20.7 min (minor).

General procedure for the racemic synthesis of arylglycines 2a-z, 2aa and 2ab-ag

In a sample vial containing the proper alkene (0.10 mmol, 1 eq.) in anhydrous toluene (1 mL, **C** = 0.1 M), TBHP (~5.5 M in decane, 23 µL, 0.12 mmol, 1.2 eq.) and 2-piperidinemethanol (2.3 mg, 0.02 mmol, 0.2 eq.) were added. The reaction mixture was stirred until consumption of the alkene (TLC eluent PE/ethyl acetate 8/2). Then the proper nucleophile - aniline, *p*-anisidine, 3,4-dimethylaniline, indoline, 5-aminoindole or 4-chloroaniline - (0.12 mmol, 1.2 eq.), methanol (405 µL, 10 mmol, 100 eq.) and triethylamine (28 µL, 0.20 mmol, 2 eq.) were added to the reaction mixture and it was stirred at room temperature (stirred in an oil bath at 60 °C for products **2ab-ag**) and monitored by TLC (eluent PE/ ethyl acetate 8/2). After completion, the mixture was diluted with ethyl acetate (10 mL) and washed with saturated NH_4Cl solution (2x10 mL) and water (1x10 mL), dried over anhydrous Na_2SO_4 and concentrated under vacuum. The crude racemic products **2** were purified by flash chromatography (hexane/ethyl acetate 100/0 to 80/20 (30-87% yields).

General procedure for one-pot asymmetric Knoevenagel/epoxidation/ring-opening/esterification reaction and epoxidation/ ring-opening/esterification reaction to arylglycines 2a-2z, 2aa and 2ab-2ag

In a sample vial containing (phenylsulfonyl)acetonitrile (18.5 mg, 0.10 mmol, 1 eq.) and the catalyst **eQNU** (5.8 mg, 0.010 mmol, 0.1 eq.) in anhydrous toluene (0.35 mL, C = 0.3 M), aldehyde (0.10 mmol, 1 eq.) was added. The reaction was stirred in an oil bath at 30 °C for 18-48 hours, monitored by TLC (eluent PE/ethyl acetate 8/2). After completion, the solution was diluted in toluene (5 mL, C = 0.02 M) and 1.1 equivalents of cumene hydroperoxide (tech. 80%, 20 µL, 0.11 mmol, 1.1 eq.) were added at –20 °C. The solution was stirred at -20 °C until consumption of the alkene **3** (TLC eluent PE/ ethyl acetate 8/2). Then the nucleophile - aniline, *p*-anisidine, 3,4-dimethylaniline, indoline, 5-aminoindole or 4-chloroaniline - (0.12 mmol, 1.2 eq.), methanol (405

µL, 10 mmol, 100 eq.) and N,N-dicyclohexylmethylamine (33 µL, 0.15 mmol, 1.5 eq.) were added and the reaction mixture was stirred for 5-33 hours at room temperature (at 0°C for compounds **2q** and **2r**), monitored by TLC (eluent PE/ ethyl acetate 8/2).

For the synthesis of compounds **2ab-ag**, in a sample vial containing the alkene **3** (0.10 mmol, 1 eq.) and the catalyst **eQNU** (5.8 mg, 0.010 mmol, 0.1 eq.) in anhydrous toluene (5 mL, C = 0.02 M), CHP (tech. 80%, 20 µL, 0.11 mmol, 1.1 eq.) was added at -20 °C and the solution was stirred at -20 °C for 36-86 hours until consumption of the alkene **1** (TLC eluent PE/ethyl acetate 8/2). Then the nucleophile - aniline, 3,4-dimethylaniline or indoline - (0.12 mmol, 1.2 eq.), methanol (405 µL, 10 mmol, 100 eq.) and N,N-dicyclohexylmethylamine (33 µL, 0.15 mmol, 1.5 eq.) were added and the reaction mixture was stirred in an oil bath for 5-140 hours at 50 °C for products **2ac-ad**, 60 °C for products **2ab-af-ag** and 70 °C for product **2ae**, monitored by TLC (eluent PE/ethyl acetate 8/2). After completion, the mixture was diluted with ethyl acetate (10 mL) and washed with saturated NH_4Cl solution (2x10 mL) and water (1x10 mL), dried over anhydrous Na_2SO_4 and concentrated under vacuum. The reaction mixture was purified by flash chromatography (eluent: hexane/ethyl acetate 100/0 to 80/20; products **2v** and **2ae** were purified by a 2 mm thick preparative TLC plate, eluent mixture hexane/ethyl acetate 80/20) to give the final products in 50-82% yield and 63-99% ee.

(*R*)-Methyl 2-(4-cyanophenyl)-2-((4-methoxyphenyl)amino)acetate 2a

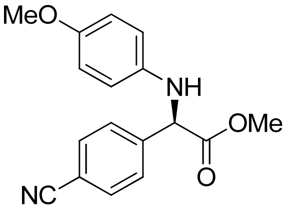

Data for this compound are consistent with those reported in the literature.[6]

Brown oil (flash chromatography eluent: hexanes/EtOAc, 100/0 to 90/10), 18.4 mg, 62% yield. $[\alpha]_D^{20}$= -101.6 (C = 0.92, CH_2Cl_2), 90% ee. **^{1}H NMR** ($CDCl_3$, 400 MHz): δ 7.64 (s, 4H), 6.71 (d, 2H, J = 8.9 Hz), 6.46 (d, 2H, J = 8.9 Hz), 5.05 (s, 1H), 4.81 (s, 1H), 3.75 (s, 3H), 3.70 (s, 3H). **^{13}C NMR** ($CDCl_3$, 100 MHz): δ 171.1, 152.8, 143.4, 139.3, 132.6, 128.1, 118.5, 114.9, 114.7, 112.2, 61.3, 55.6, 53.2. **HRMS (MALDI-FT ICR)** m/z: $[M]^+$ calculated for $C_{17}H_{16}N_2O_3$: 296.1155, found: 296.1176. HPLC (IC, 2-propanol/n-hexane = 30/70, flow rate = 1.0 mL/min, l = 220 nm) t_R = 16.3 min (major), 19.1 min (minor).

[6] Guo, W.; Wang, M.; Han, Z.; Huang, H.; Jiang, F.; Sun J. *Chem. Sci.* **2021**, *12*, 1193.

2-(4-Cyanophenyl)-N-(4-methoxyphenyl)-2-((4-methoxyphenyl)amino)acetamide 2a’

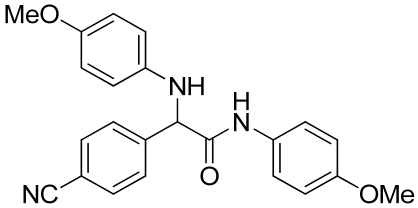

Yellow oil (flash chromatography eluent: hexanes/EtOAc, 90/10 to 60/40), 7.8 mg, 20% yield. **^{1}H NMR** ($CDCl_3$, 600 MHz): δ 8.75 (s, 1H), 7.67 (d, 2H, J = 8.3 Hz), 7.61 (d, 2H, J = 8.3), 7.41 (d, 2H, J = 8.9 Hz), 6.84 (d, 2H, J = 8.9 Hz), 6.81 (d, 2H, J = 8.9 Hz) ,6.67 (d, 2H, J = 8.9 Hz), 4.82 (s, 1H), 4.24 (s, 1H), 3.78 (s, 3H), 3.75 (s, 3H). **^{13}C NMR** ($CDCl_3$, 150 MHz): δ 168.2, 156.8, 153.9, 143.6, 139.7, 132.9, 130.0, 128.2, 121.7, 118.3, 115.6, 115.0, 114.2, 112.6, 65.5, 55.6, 55.5. **HRMS (MALDI-FT ICR)** m/z: $[M+H]^+$ calculated for $C_{23}H_{22}N_3O_3$: 388.1656, found: 388.1667.

(*R*)-Methyl 2-phenyl-2-(phenylamino)acetate 2b

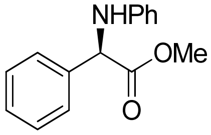

Data for this compound are consistent with those reported in the literature.[7,8]

Colourless oil (flash chromatography eluent: hexanes/EtOAc, 100/0 to 98/2), 17.4 mg, 72% yield. $[\alpha]_D^{20}$= -95.6 (C = 0.79, $CHCl_3$), 92% ee. **^{1}H NMR** ($CDCl_3$, 600 MHz): δ 7.50 (d, 2H, J = 7.6 Hz), 7.37-7.35 (m, 2H), 7.33-7.30 (m, 1H), 7.14-7.11 (m, 2H), 6.70 (t, 1H, J = 7.3 Hz), 6.57 (d, 2H, J = 8.2 Hz), 5.09 (s, 1H), 4.96 (bs, 1H), 3.74 (s, 3H). **^{13}C NMR** ($CDCl_3$, 150 MHz): 172.3, 145.9, 137.6, 129.2, 128.9, 128.3, 127.2, 118.1, 113.4, 60.7, 52.8. **HRMS (MALDI-FT ICR)** m/z: $[M+H]^+$ calculated for $C_{15}H_{16}NO_2$: 242.1181, found: 242.1171. HPLC (IC, 2-propanol/n-hexane = 5/95, flow rate = 1.0 mL/min, l = 220 nm) t_R = 6.3 min (major), 6.8 min (minor).

(*R*)-Methyl 2-(phenylamino)-2-(*p*-tolyl)acetate 2c

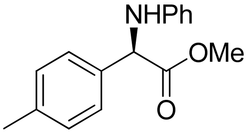

Data for this compound are consistent with those reported in the literature.[9]

Yellow oil (flash chromatography eluent: hexanes/EtOAc, 100/0 to 98/2), 20.0 mg, 78% yield. $[\alpha]_D^{19}$ = -97.0 (C = 0.96, CH_2Cl_2), 93% ee. **^{1}H NMR** ($CDCl_3$, 400 MHz): δ 7.38 (d, 2H, J = 8.0 Hz), 7.17 (d, 2H, J = 8.0 Hz), 7.12 (m, 2H), 6.70 (t, 1H, *J* = 7.3 Hz), 6.56 (d, 2H, J = 7.8 Hz), 5.05 (d, 1H, J = 5.6 Hz), 4.93 (bs, 1H), 3.73 (s, 3H), 2.34 (s, 3H). **^{13}C NMR** ($CDCl_3$, 150 MHz): δ 172.5,

[7] Liu, D., Li, B., Chen, J.; Gridnev, I. D.; Yan, D.; Zhang, W. *Nat. Commun.* **2020**, *11*, 5935.
[8] Morrill, L. C.; Lebl, T.; Slawin, A. M. Z.; Smith A. D. *Chem. Sci.* **2012**, *3*, 2088.
[9] Guo, W.; Zhou, Y.; Xie, H.; Yue, X.; Jiang, F.; Huang, H.; Han, Z.; Sun J. *Chem. Sci.* **2023**, *14*, 843.

146.0, 138.1, 134.6, 129.6, 129.2, 127.1, 118.0, 113.3, 60.4, 52.7, 21.1. **HRMS (MALDI-FT ICR)** m/z: $[M+H]^+$calculated for $C_{16}H_{18}NO_2$: 256.1332, found: 256.1327. HPLC (OD-H, 2-propanol/n-hexane = 5/95, flow rate = 1.0 mL/min, l = 220 nm) t_R = 10.6 min (major), 9.1 min (minor).

(*R*)-Methyl 2-(phenylamino)-2-(*m*-tolyl)acetate 2d

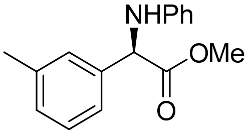

Colourless oil (flash chromatography eluent: hexanes/EtOAc, 100/0 to 98/2), 18.6 mg, 73% yield. $[\alpha]_D^{25}$ = -91.3 (C = 0.64, $CHCl_3$), 93% ee. **^{1}H NMR** ($CDCl_3$, 400 MHz): δ 7.29-7.22 (m, 3H), 7.14-7.12 (m, 3H), 6.70 (t, 1H, J = 7.1 Hz), 6.56 (d, 2H, J = 8.4 Hz), 5.02 (d, 1H, J= 5.5 Hz), 4.91 (bs, 1H), 3.73 (s, 3H), 2.34 (s, 3H). **^{13}C NMR**: ($CDCl_3$, 100 MHz): δ 172.4, 146.0, 138.6, 137.5, 129.2, 129.1, 128.7, 127.8, 124.4, 118.0, 113.3, 60.7, 52.8, 21.4. **HRMS (MALDI-FT ICR)** m/z: $[M+H]^+$ calculated for $C_{16}H_{18}NO_2$: 256.1332, found: 256.1326. HPLC (IC, 2-propanol/n-hexane = 3/97, flow rate = 1.0 mL/min, l = 220 nm) t_R = 7.1 min (major), 7.8 min (minor).

(*R*)-Methyl 2-(4-fluorophenyl)-2-(phenylamino)acetate 2e

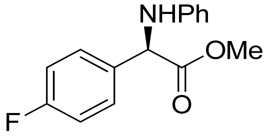

Yellow oil (flash chromatography eluent: hexanes/EtOAc, 100/0 to 98/2), 15.6 mg, 60% yield. $[\alpha]_D^{25}$ = -64.1 (C = 0.54, $CHCl_3$), 88% ee. **^{1}H NMR** ($CDCl_3$, 600 MHz): δ 7.49-7.47 (m, 2H), 7.15-7.12 (m, 2H), 7.05 (t, 2H, J = 8.6 Hz), 6.72 (t, 1H, J = 7.3 Hz), 6.54 (d, 2H, J = 8.3 Hz), 5.07 (s, 1H), 4.98 (bs, 1H), 3.75 (s, 3H). **^{13}C NMR**: ($CDCl_3$, 150 MHz): δ 172.0. 162.6 (d, $^1J_{CF}$ = 246 Hz), 145.7, 133.3 (d, $^4J_{CF}$ = 3.0 Hz), 129.2, 128.9 (d, $^3J_{CF}$ = 8.2 Hz), 118.2, 115.8 (d, $^2J_{CF}$ = 21.7 Hz), 113.4, 60.0, 52.9. **HRMS (MALDI-FT ICR)** m/z $[M+H]^+$ calculated for $C_{15}H_{15}FNO_2$: 260.1081, found: 260.1098. HPLC (IC, 2-propanol/n-hexane = 5/95, flow rate = 1.0 mL/min, l = 254 nm) t_R = 5.9 min (major), 6.5 min (minor).

(*R*)-Methyl 2-(4-bromophenyl)-2-(phenylamino)acetate 2f

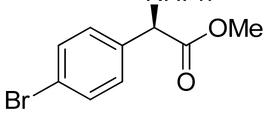

Data for this compound are consistent with those reported in the literature.[10]

Yellow oil (flash chromatography eluent: hexanes/EtOAc, 100/0 to 98/2), 24.3 mg, 76% yield. $[\alpha]_D^{25}$ = -100.4 (C = 0.86, $CHCl_3$), 94% ee. **^{1}H NMR** ($CDCl_3$, 300 MHz): δ 7.49 (d, 2H, J = 8.5 Hz), 7.39 (d, 2H, J = 8.5 Hz), 7.13 (m, 2H), 6.72 (t, 1H, J = 7.4 Hz), 6.52 (d, 2H, J = 7.7 Hz), 5.03 (m,

[10] Shen, G.; Liu, H.; Chen, J.; He, Z.; Zhou, Y.; Wang, L.; Luo, Y.; Su, Z.; Fan, B. *Org. Biomol. Chem.* **2021**, *19*, 3601.

2H), 3.74 (s, 3H). **^{13}C NMR** ($CDCl_3$, 75 MHz): δ 171.7, 145.5, 136.7, 131.9, 129.2, 128.9, 122.2, 118.3, 113.3, 60.1, 53.0. **HRMS (MALDI-FT ICR)** m/z $[M+H]^+$ calculated for $C_{15}H_{15}BrNO_2$:320.0281, found: 320.0290. HPLC (IE-3, 2-propanol/n-hexane = 5/95, flow rate = 0.8 mL/min, l = 254 nm) t_R = 9.5 min (major), 10.6 min (minor).

(*R*)-Methyl 2-(3-bromophenyl)-2-(phenylamino)acetate 2g

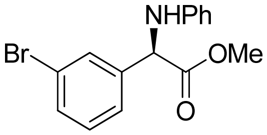

Yellow oil (flash chromatography eluent: hexanes/EtOAc, 100/0 to 98/2), 16.1 mg, 50% yield. $[\alpha]_D^{24}$ = -74.8 (C = 0.68, $CHCl_3$), 90% ee. **^{1}H NMR** ($CDCl_3$, 600 MHz): δ 7.67 (t, 1H, J = 1.7 Hz), 7.45-7.43 (m, 2H), 7.23 (dd, 1H, J = J = 7.9 Hz), 7.15-7.12 (m, 2H), 6.73 (t, 1H, J = 7.3 Hz), 6.54 (dd, 2H, J = 8.7, 1.1 Hz), 5.03 (d, 1H, J = 5.2), 5.00 (bs, 1H), 3.75 (s, 3H). **^{13}C NMR** ($CDCl_3$, 150 MHz): δ 171.6, 145.5, 140.1, 131.5, 130.4, 130.3, 129.3, 125.9, 122.9, 118.4, 113.4, 60.2, 53.0. **HRMS (MALDI-FT ICR)** m/z $[M+H]^+$ calculated for $C_{15}H_{15}BrNO_2$: 320.0281, found: 320.0272. HPLC (IC, 2-propanol/n-hexane = 5/95, flow rate = 1.0 mL/min, l = 220 nm) t_R = 5.5 min (major), 6.3 min (minor).

(*R*)-Methyl 2-(4-chlorophenyl)-2-(phenylamino)acetate 2h

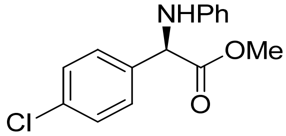

Data for this compound are consistent with those reported in the literature.[10]

Colourless oil (flash chromatography eluent: hexanes/EtOAc, 100/0 to 98/2), 17.9 mg, 65% yield. $[\alpha]_D^{19}$ = -95.4 (C = 0.62, $CHCl_3$), 89% ee. **^{1}H NMR** ($CDCl_3$, 400 MHz): δ 7.44 (d, 2H, J = 8.4 Hz), 7.32 (d, 2H, *J* = 8.4 Hz), 7.12 (m, 2H), 6,71 (t, 1H, J = 7.3 Hz), 6.53 (d, 2H, J = 8.2 Hz), 5.05 (d, 1H, J = 4.2 Hz), 4.99 (bs, 1H), 3.74 (s, 3H). **^{13}C NMR** ($CDCl_3$, 100 MHz): δ 171.8, 145.6, 136.2, 134.1, 129.3, 129.0, 128.6, 118.4, 113.4, 60.1, 52.9. **HRMS (MALDI-FT ICR)** m/z: $[M+H]^+$ calculated for $C_{15}H_{15}ClNO_2$: 276.0786, found: 276.0772. HPLC (IC, 2-propanol/n-hexane = 5/95, flow rate = 1 mL/min, l = 254 nm) t_R = 6.0 min (major), 6.6 min (minor).

(*R*)-Methyl 2-(2-fluorophenyl)-2-(phenylamino)acetate 2i

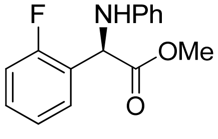

Yellow solid (flash chromatography eluent: hexanes/EtOAc, 100/0 to 98/2), 19.5 mg, 75% yield. **mp:** 82.5-83.4 $[\alpha]_D^{19}$ = -89.5 (C = 0.77, $CHCl_3$), 63% ee. **^{1}H NMR** ($CDCl_3$, 400 MHz): δ 7.46-7.42 (m, 1H), 7.32-7.28 (m, 1H), 7.16-7.09 (m, 4H), 6.72 (t, 1H, J = 7.4 Hz), 6.60 (d, 2H, J = 8.0 Hz),

5.46 (s, 1H), 4.99 (bs, 1H), 3.74 (s, 3H). **^{13}C NMR**: ($CDCl_3$, 100 MHz): δ 171.8. 160.8 (d, $^1J_{CF}$=247 Hz), 145.6, 129.9 (d, $^3J_{CF}$=8.3 Hz), 129.3, 128.2 (d, $^3J_{CF}$=3.2 Hz), 125.2 (d, $^2J_{CF}$=13.7 Hz), 124.6 (d, $^4J_{CF}$=3.4 Hz), 118.4, 115.8 (d, $^2J_{CF}$=21.9 Hz), 113.3, 53.7 (d, $^3J_{CF}$=3.0 Hz), 52.9. **HRMS (MALDI-FT ICR)** m/z: $[M+H]^+$calculated for $C_{15}H_{15}FNO_2$: 260.1081, found: 260.1104. HPLC (IC, 2-propanol/n-hexane = 10/90, flow rate = 1.0 mL/min, l = 220 nm) t_R = 4.8 min (major), 5.2 min (minor).

(*R*)-Methyl 2-(phenylamino)-2-(4-(trifluoromethoxy)phenyl)acetate 2j

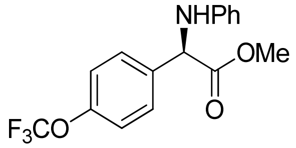

Colourless oil (flash chromatography eluent: hexanes/EtOAc, 100/0 to 98/2), 16.9 mg, 52% yield. $[\alpha]_D^{24}$ = -66.6 (C = 0.79, $CHCl_3$), 84% ee. **^{1}H NMR** ($CDCl_3$, 600 MHz): δ 7.54 (d, 2H, J = 8.5 Hz), 7.21 (d, 2H, J = 8.5 Hz), 7.15-7.12(m, 2H), 6.73 (t, 1H, J = 7.3 Hz), 6.54 (dd, 2H, J = 8.5, 0.9 Hz), 5.09 (bs, 1H), 5.01 (bs, 1H), 3.76 (s, 3H). **^{13}C NMR** ($CDCl_3$, 75 MHz): δ 171.8. 149.1, 145.6, 136.3, 129.3, 128.6, 121.3, 120.4 (q, $^1J_{CF}$=257 Hz), 118.3, 113.4, 60.0, 53.0. **HRMS (MALDI-FT ICR)** m/z: $[M+H]^+$ calculated for $C_{16}H_{15}F_3NO_3$: 326.0999, found: 326.1018. HPLC (OD-H, 2-propanol/n-hexane = 10/90, flow rate = 1.0 mL/min, l = 220 nm) t_R = 7.9 min (major), 10.0 min (minor).

(*R*)-Methyl 2-(phenylamino)-2-(4-((trifluoromethyl)thio)phenyl)acetate 2k

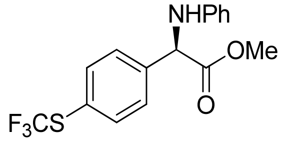

Yellow oil (flash chromatography eluent: hexanes/EtOAc, 100/0 to 98/2), 20.5 mg, 60% yield. $[\alpha]_D^{22}$ = -87.1 (C = 0.65, $CHCl_3$), 91% ee. **^{1}H NMR** ($CDCl_3$, 600 MHz): δ 7.64 (d, 2H, J = 8.2 Hz), 7.58 (d, 2H, J = 8.2 Hz), 7.15-7.12 (m, 2H), 6.73 (t, 1H, J = 7.3 Hz), 6.53 (d, 2H, J = 7.8 Hz), 5.12 (d, 1H, J = 3.3 Hz), 5.04 (bs, 1H), 3.77 (s, 1H). **^{13}C NMR** ($CDCl_3$, 150 MHz): δ 171.4. 145.5, 140.8, 136.7, 129.5 (q, $^1J_{CF}$ = 308 Hz), 129.3, 128.3, 124.3, 118.4, 113.4, 60.3, 53.1. **HRMS (MALDI-FT ICR)** m/z: $[M+H]^+$ calculated for $C_{16}H_{15}F_3NO_2S$: 342.0770, found: 342.0768. HPLC (OD-H, 2-propanol/n-hexane = 10/90, flow rate = 1.0 mL/min, l = 220 nm) t_R = 8.9 min (major), 11.5 min (minor).

(*R*)-Methyl 2-(3,4-dichlorophenyl)-2-(phenylamino)acetate 2l

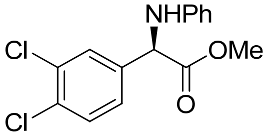

Colourless oil (flash chromatography eluent: hexanes/EtOAc, 100/0 to 98/2), 18.5 mg, 60% yield. $[\alpha]_D^{24}$ = -91.7 (C = 0.65, $CHCl_3$), 94% ee. ^{1}**H NMR** ($CDCl_3$, 400 MHz): δ 7.62 (s, 1H), 7.43 (d, 1H, J = 8.3 Hz), 7.35 (d, 1H, J = 8.3 Hz), 7.14 (m, 2H), 6.74 (t, 1H, J = 7.2 Hz), 6.52 (d, 2H, J = 7.9 Hz), 5.02 (s, 2H), 3.76 (s, 3H). ^{13}**C NMR** ($CDCl_3$, 75 MHz): δ 171.2. 145.3, 138.1, 133.0, 132.4, 130.8, 129.3, 129.2, 126.5, 118.5, 113.4, 59.7, 53.2. **HRMS (MALDI-FT ICR)** m/z: $[M+H]^+$ calculated for $C_{15}H_{14}Cl_2NO_2$: 310.0396, found: 310.0420. HPLC (IC, 2-propanol/n-hexane = 5/95, flow rate = 1.0 mL/min, l = 254 nm) t_R = 5.5 min (major), 6.3 min (minor).

(*R*)-Methyl 2-(3,5-bis(trifluoromethyl)phenyl)-2-(phenylamino)acetate 2m

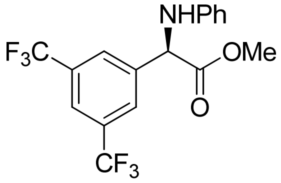

Yellow oil (flash chromatography eluent: hexanes/EtOAc, 100/0 to 98/2), 19.2 mg, 51% yield. 91% ee. ^{1}**H NMR** ($CDCl_3$, 600 MHz): δ 8.00 (s, 2H), 7.84 (s, 1H), 7.17-7.14 (m, 2H), 6.77 (d, 1H, J = 7.3 Hz), 6.52 (d, 2H, J = 8.3 Hz), 5.17 (d, 1H, J = 3.3 Hz), 5.12 (s, 1H), 3.79 (s, 3H). ^{13}**C NMR**: ($CDCl_3$, 75 MHz): δ 170.7. 145.2, 140.8, 132.2 (q, $^2J_{CF}$ = 33.5 MHz), 129.4, 127.4, 123.1 (q, $^1J_{CF}$ = 272 Hz), 122.5 (q, $^3J_{CF}$ = 3.6 Hz), 119.0, 113.5, 60.3, 53.4. **HRMS (MALDI-FT ICR)** m/z: $[M+H]^+$ calculated for $C_{17}H_{14}F_6NO_2$: 378.0923, found: 378.0940. HPLC (OD-H, 2-propanol/n-hexane = 10/90, flow rate = 1.0 mL/min, l = 220 nm) t_R = 8.0 min (major), 10.6 min (minor).

(*R*)-Methyl 2-([1,1'-biphenyl]-4-yl)-2-(phenylamino)acetate 2n

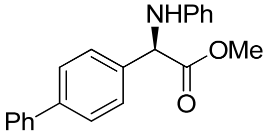

Data for this compound are consistent with those reported in the literature.[10]

White solid (flash chromatography eluent: hexanes/EtOAc, 100/0 to 98/2), 20.3 mg, 64% yield. **mp:** 118.5-119.8. $[\alpha]_D^{23}$ = -119.0 (C = 0.78, $CHCl_3$), 91% ee. ^{1}**H NMR** ($CDCl_3$, 400 MHz): δ 7.60-7.57 (m, 6H), 7.46-7.42 (m, 2H), 7.37-7.35 (m, 1H), 7.17-7.13 (m, 2H), 6.72 (t, 1H, J = 7.3 Hz), 6.59 (d, 2H, J = 7.8 Hz) 5.14 (s, 1H), 3.77 (s, 3H). ^{13}**C NMR** ($CDCl_3$, 100 MHz): δ 172.2, 145.8, 141.2, 140.5, 136.5, 129.2, 128.7, 127.5 (3C), 127.4, 127.0, 118.1, 113.4, 60.4, 52.9. **HRMS (MALDI-FT ICR)** m/z: $[M+H]^+$ calculated for $C_{21}H_{20}NO_2$: 318.1489, found: 318.1473. HPLC

(OD-H, 2-propanol/n-hexane = 5/95, flow rate = 1.0 mL/min, l = 254 nm) t_R = 16.9 min (major), 15.2 min (minor).

(*R*)-Methyl 2-(naphthalen-2-yl)-2-(phenylamino)acetate 2o

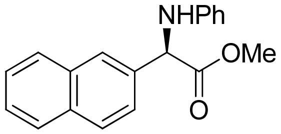

Data for this compound are consistent with those reported in the literature.[10]

White solid (flash chromatography eluent: hexanes/EtOAc, 100/0 to 98/2), 18.4 mg, 63% yield. **mp:** 72.2-72.9. $[\alpha]_D^{20}$= -115.5 (C = 0.91, $CHCl_3$), 94% ee. **^{1}H NMR** ($CDCl_3$, 600 MHz): δ 7.99 (s, 1H), 7.87-7.83 (m, 3H), 7.63 (dd, 1H, J = 8.5, 1.5 Hz), 7.51-7.48 (m, 2H), 7.14-7.12 (m, 2H), 6.71 (t, 1H, J = 7.3 Hz), 6.62 (d, 2H, J = 7.9 Hz), 5.26 (s, 1H), 5.11 (bs, 1H), 3.75 (s, 3H). **^{13}C NMR** ($CDCl_3$, 150 MHz): δ 172.1, 145.9, 135.1, 133.3, 133.2, 129.2, 128.7, 128.0, 127.7, 126.4, 126.3, 126.2, 124.9, 118.1, 113.4, 60.9, 52.8. **HRMS (MALDI-FT ICR)** m/z: $[M+H]^+$ calculated for $C_{19}H_{18}NO_2$: 292.1332, found: 292.1327. HPLC (IC, 2-propanol/n-hexane = 5/95, flow rate = 1.0 mL/min, l = 254 nm) t_R = 7.3 min (major), 8.5 min (minor).

(*R*)-Methyl 2-(phenanthren-9-yl)-2-(phenylamino)acetate 2p

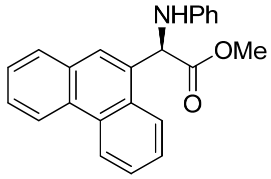

White solid (flash chromatography eluent: hexanes/EtOAc, 100/0 to 98/2), 19.5 mg, 57% yield. **mp:** 146.5-147.6. $[\alpha]_D^{19}$ = -44.7 (C = 0.87, $CHCl_3$), 74% ee. **^{1}H NMR** ($CDCl_3$, 300 MHz): δ 8.80-8.77 (m, 1H), 8.68 (d, 1H, J = 8.4 Hz), 8.38-8.34 (m, 1H), 7.93 (s, 1H), 7.86 (d, 1H, J = 7.5 Hz), 7.73-7.59 (m, 4H), 7.16-7.11 (m, 2H), 6.72 (t, 1H, J = 7.3 Hz), 6.63 (d, 2H, J = 7.8 Hz), 5.87 (d, 1H, J = 5.0 Hz), 5.03 (d, 1H, J = 5.0 Hz), 3.74 (s, 3H). **^{13}C NMR**: ($CDCl_3$, 75 MHz): δ 172.7. 146.2, 131.6, 131.2, 131.1, 130.4, 130.0, 129.3, 129.0, 127.2, 127.0, 126.8, 126.6, 126.3, 124.1, 123.4, 122.4, 118.3, 113.3, 57.7, 52.8. **HRMS (MALDI-FT ICR)** m/z: $[M+H]^+$ calculated for $C_{23}H_{20}NO_2$: 342.1489, found: 342.1475. HPLC (IE-3, 2-propanol/n-hexane = 5/95, flow rate = 0.6 mL/min, l = 254 nm) t_R = 19.0 min (major), 18.2 min (minor).

(*R*)-Methyl 2-(phenylamino)-2-(pyridin-3-yl)acetate 2q

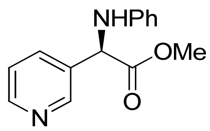

Yellow oil (flash chromatography eluent: hexanes/EtOAc, 98/2 to 80/20), 14.1 mg, 58% yield. $[\alpha]_D^{19}$ = -62.7 (C = 0.29, $CHCl_3$), 89% ee. **^{1}H NMR** ($CDCl_3$, 400 MHz): δ 8.79 (s, 1H), 8.57 (d, 1H,

J = 4.0 Hz), 7.80 (d, 1H, J = 7.9 Hz), 7.29-7.27 (m, 1H), 7.15-7.11 (m, 2H), 6.73 (t, 1H, J = 7.3 Hz), 6.54 (d, 2H, J = 7.7 Hz), 5.12 (d, 1H, J = 4.7 Hz), 5.00 (d, 1H, J = 4.7 Hz), 3.76 (s, 3H). **^{13}C NMR** (CDCl$_3$, 100 MHz): δ 171.4. 149.6, 149.3, 145.3, 134.4, 133.5, 129.3, 123.8, 118.5, 113.4, 58.5, 53.1. **HRMS (MALDI-FT ICR)** m/z: [M+H]$^+$ calculated for $C_{14}H_{15}N_2O_2$: 243.1128, found: 243.1137. HPLC (IC, 2-propanol/n-hexane = 30/70, flow rate = 1.0 mL/min, l = 254 nm) t_R = 10.3 min (major), 15.6 min (minor).

(*R*)-Methyl 2-(2-methylquinolin-3-yl)-2-(phenylamino)acetate 2r

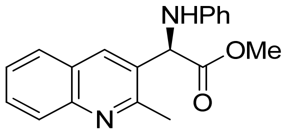

Red wax (flash chromatography eluent: hexanes/EtOAc, 98/2 to 80/20), 17.2 mg, 56% yield. $[\alpha]_D^{19}$ = -21.42 (C = 0.43, CHCl$_3$), 76% ee. **^{1}H NMR** (CDCl$_3$, 400 MHz): δ 8.20 (s, 1H), 8.03 (d, 1H, J=8.4 Hz), 7.73-7.66 (m, 2H), 7.48-7.44 (m, 1H), 7.15-7.11 (m, 2H), 6.72 (t, 1H, J = 7.2 Hz), 6.55 (d, 2H, J = 8.5 Hz), 5.42 (d, 1H, J = 5.2 Hz), 5.07 (d, 1H, J = 5.2 Hz), 3.75 (s, 3H), 2.98 (s, 3H). **^{13}C NMR**: (CDCl$_3$, 100 MHz): δ 171.9. 157.6, 147.2, 145.6, 134.1, 130.1, 129.8, 129.3, 128.3, 127.7, 126.9, 126.1, 118.6, 113.3, 57.3, 53.0, 23.4. **HRMS (MALDI -FT ICR)** m/z: [M+H]$^+$ calculated for $C_{19}H_{19}N_2O_2$: 307.1441, found: 307.1455. HPLC (OD-H, 2-propanol/n-hexane = 20/80, flow rate = 1.0 mL/min, l = 254 nm) t_R = 7.7 min (major), 12.9 min (minor).

(*R*)-Methyl 2-((4-methoxyphenyl)amino)-2-(3-nitrophenyl)acetate 2s

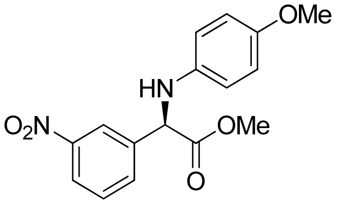

Data for this compound are consistent with those reported in the literature.[7,11]

Orange oil (flash chromatography eluent: hexanes/EtOAc, 98/2 to 92/8), 24.9 mg, 79% yield. $[\alpha]_D^{24}$ = -62.3 (C = 0.94, CHCl$_3$), 93% ee. **^{1}H NMR** (CDCl$_3$, 400 MHz): δ 8.39 (m, 1H), 8.17 (d, 1H, J = 8.1 Hz), 7.85 (d, 1H, J = 7.6 Hz), 7.53 (dd, 1H, J = J = 7.9 Hz), 6.71 (d, 2H, J = 8.8 Hz), 6.48 (d, 2H, J = 8.8 Hz), 5.11 (s, 1H), 4.88 (bs, 1H), 3.75 (s, 3H), 3.70 (s, 3H). **^{13}C NMR**:(CDCl$_3$, 150 MHz): δ 171.1, 152.8, 148.6, 140.4, 139.3, 133.2, 129.8, 123.3, 122.4, 114.9, 114.8, 61.0, 55.6, 53.2. **HRMS (MALDI-FT ICR)** m/z: [M]$^+$ calculated for $C_{16}H_{16}N_2O_5$: 316.1054, found: 316.1076. HPLC (IC, 2-propanol/n-hexane = 30/70, flow rate = 1.0 mL/min, l = 254 nm) t_R = 13.6 min (major), 14.9 min (minor).

[11] Shang, G.; Yang, Q.; Zhang, X. *Angew. Chem. Int. Ed.* **2006**, *45*, 6360.

(*R*)-Methyl 4-(2-methoxy-1-((4-methoxyphenyl)amino)-2-oxoethyl)benzoate 2t

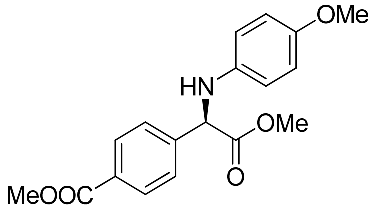

Data for this compound are consistent with those reported in the literature.[12]

Orange wax (flash chromatography eluent: hexanes/EtOAc, 98/2 to 90/10), 19.8 mg, 60% yield. $[\alpha]_D^{23}$ = -82.3 (C = 0.73, $CHCl_3$), 92% ee. **^{1}H NMR** ($CDCl_3$, 400 MHz): δ 8.02 (d, 2H, J = 8.3 Hz), 7.57 (d, 2H, J = 8.3 Hz), 6.71 (d, 2H, J = 8.9 Hz), 6.49 (d, 2H, J = 8.9 Hz), 5.07 (s, 1H), 4.77 (bs, 1H), 3.90 (s, 3H), 3.73 (s, 3H), 3.70 (s, 3H). **^{13}C NMR**: ($CDCl_3$, 100 MHz): δ 171.7. 166.5, 152.6, 143.0, 139.7, 130.1, 127.3, 116.4, 114.8, 114.7, 61.4, 55.6, 52.9, 52.1. **HRMS (ESI-FT ICR)** m/z: $[M+H]^+$ calculated for $C_{18}H_{20}NO_5$: 330.1336, found: 330.1342. HPLC (IC, 2-propanol/n-hexane = 30/70, flow rate = 1.0 mL/min, l = 220 nm) t_R = 13.0 min (major), 15.9 min (minor).

(*R*)-Methyl 2-(3,4-difluorophenyl)-2-((4-methoxyphenyl)amino)acetate 2u

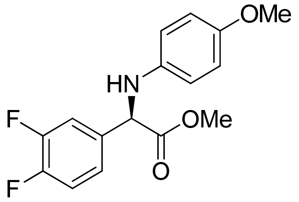

Data for this compound are consistent with those reported in the literature.[7]

Yellow liquid (flash chromatography eluent: hexanes/EtOAc, 100/0 to 98/2), 17.2 mg, 56% yield. $[\alpha]_D^{18}$ = -56.4 (C = 0.81 M, CH_2Cl_2), 94% ee. **^{1}H NMR** ($CDCl_3$, 600 MHz): δ 7.35-7.32 (m, 1H), 7.26-7.23 (m, 1H), 7.16-7.12 (m, 1H), 6.73 (d, 2H, J = 8.9 Hz), 6.49 (d, 2H, J = 8.9 Hz), 4.96 (s, 1H), 4.74 (bs, 1H), 3.75 (s, 3H), 3.71 (s, 3H). **^{13}C NMR** ($CDCl_3$, 150 MHz): 171.7, 152.7, 150.5 (dd, $^1J_{CF}$ = 249 Hz, $^3J_{CF}$ = 12.8 Hz), 150.2 (dd, $^1J_{CF}$ = 249 Hz, $^3J_{CF}$ = 12.7 Hz), 139.6, 134.9 (dd, $^3J_{CF}$ = $^4J_{CF}$ = 4.1 Hz), 123.3 (dd, $^3J_{CF}$ = 5.8 Hz, $^4J_{CF}$ = 3.5 Hz), 117.6 (d, $^2J_{CF}$ = 17.4 Hz), 116.3 (d, $^2J_{CF}$ = 18.2 Hz), 114.9, 114.7, 60.6, 55.7, 53.0. **HRMS (MALDI-FT ICR)** m/z: $[M]^+$ calculated for $C_{16}H_{15}F_2NO_3$: 307.1015, found: 307.1015. HPLC (IC, 2-propanol/n-hexane = 5/95, flow rate = 1.0 mL/min, l = 220 nm) t_R = 10.4 min (major), 12.7 min (minor).

[12] Ishikawa, H.; Touge, T.; Kuriyama, W.; Kida, M.; Matsumura, K. *Org. Lett.* **2023**, *25*, 2357.

(*R*)-Methyl 2-(4-isopropylphenyl)-2-((4-methoxyphenyl)amino)acetate 2v

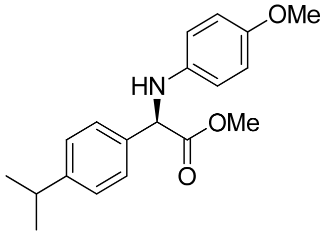

Light brown wax (TLC plate, eluent: hexanes/EtOAc, 80/20), 21.3 mg, 68% yield. $[\alpha]_D^{23}$ = -61.9 (C = 0.78, $CHCl_3$), 91% ee. **^{1}H NMR** ($CDCl_3$, 400 MHz): δ 7.39 (d, 2H, J = 8.1 Hz), 7.21 (d, 2H, J = 8.1 Hz), 6.73 (d, 2H, J = 8.9 Hz), 6.55 (d, 2H, J = 8.9 Hz), 5.00 (s, 1H), 4.59 (bs, 1H), 3.72 (s, 3H), 3.71 (s, 3H), 2.93-2.86 (hept, 1H, J = 6.9 Hz), 1.24 (d, 6H, J = 6.9 Hz). **^{13}C NMR**: ($CDCl_3$, 100 MHz): δ 172.8. 152.4, 148.9, 140.3, 134.9, 127.1, 126.9, 114.8, 114.6, 61.3, 55.6, 52.6, 33.7, 23.9. **HRMS (MALDI-FT ICR)** m/z: $[M+H]^+$ calculated for $C_{19}H_{24}NO_3$: 314.1751, found: 314.1763. HPLC (IE-3, 2-propanol/n-hexane = 5/95, flow rate = 0.8 mL/min, l = 220 nm) t_R = 17.8 min (major), 16.6 min (minor).

(*R*)-Methyl 2-((3,4-dimethylphenyl)amino)-2-(3-methoxyphenyl)acetate 2w

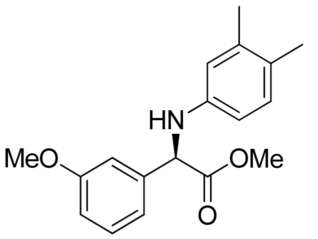

Orange oil (flash chromatography eluent: hexanes/EtOAc, 100/0 to 98/2), 24.6 mg, 82% yield. $[\alpha]_D^{23}$ = -71.5 (C = 0.75, $CHCl_3$), >99% ee. **^{1}H NMR** ($CDCl_3$, 600 MHz): δ 7.28-7.26 (m, 1H), 7.08 (d, 1H, J = 7.6 Hz), 7.04 (d, 1H, J = 1.8 Hz), 6.89 (d, 1H, J = 8.0 Hz), 6.89-6.84 (m, 1H), 6.64 (s, 1H), 6.32 (d, 1H, J = 8.0 Hz), 5.03 (s, 1H), 4.74 (bs, 1H), 3.80 (s, 3H), 3.73 (s, 3H), 2.16 (s, 3H), 2.12 (s, 3H). **^{13}C NMR**: ($CDCl_3$, 100 MHz): δ 172.4. 159.9, 144.1, 139.4, 137.3, 130.2, 129.8, 126.1, 119.5, 115.3, 113.6, 112.7, 110.6, 60.9, 55.2, 52.7, 20.0, 18.7. **HRMS (MALDI-FT ICR)** m/z: $[M+Na]^+$ calculated for $C_{18}H_{21}NaNO_3$: 322.1414, found: 322.1473. HPLC (IC, 2-propanol/n-hexane = 10/90, flow rate = 1.0 mL/min, l = 220 nm) t_R = 7.2 min (major), 12.1 min (minor).

(*R*)-Methyl 2-(3-chlorophenyl)-2-(naphthalen-2-ylamino)acetate 2x

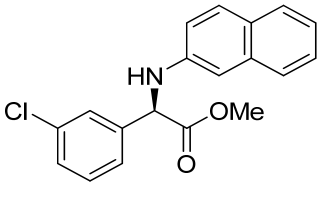

Yellow solid (flash chromatography eluent: hexanes/EtOAc, 100/0 to 98/2), 23.5 mg, 72% yield. **mp:** 112.2-112.6. $[\alpha]_D^{23}$ = -139.3 (C = 0.88, $CHCl_3$), 93% ee. **^{1}H NMR** ($CDCl_3$, 600 MHz): δ 7.67-

7.64 (m, 2H), 7.58 (s, 1H), 7.53 (d, 1H, J = 8.2 Hz), 7.47-7.45 (m, 1H), 7.35-7.34 (m, 1H), 7.31-7.30 (m, 2H), 7.23-7.20 (m, 1H), 6.97 (dd, 1H, J = 8.7 Hz, 2.3 Hz), 6.61 (d, 1H, J = 2.0 Hz), 5.20 (s, 2H). 3.79 (s, 3H). **^{13}C NMR** ($CDCl_3$, 150 MHz): δ 171.6, 143.1, 139.5, 134.9, 134.8, 130.1, 129.2, 128.6, 127.8, 127.6, 127.4, 126.4, 126.1, 125.4, 122.5, 117.9, 105.8, 60.3, 53.0. **HRMS (MALDI-FT ICR)** m/z: $[M]^+$ calculated for $C_{19}H_{16}ClNO_2$: 325.0864, found: 325.0895. HPLC (IC, 2-propanol/n-hexane = 5/95, flow rate = 1.0 mL/min, l = 254 nm) t_R = 7.2 min (major), 9.5 min (minor).

(*R*)-Methyl 2-((4-chlorophenyl)amino)-2-(3-fluorophenyl)acetate 2y

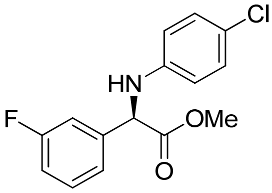

White solid (flash chromatography eluent: hexanes/EtOAc, 100/0 to 98/2), 14.7 mg, 50% yield. **mp:** 76.0-76.5. $[\alpha]_D^{20}$ = -71.5 (C = 0.79, $CHCl_3$), 93% ee. **^{1}H NMR** ($CDCl_3$, 600 MHz): δ 7.35-7.31 (m, 1H), 7.27 (d, 1H, J = 7.8 Hz), 7.19 (d, 1H, J = 9.4 Hz), 7.07 (d, 2H, J = 8.8), 7.02-7.00 (m, 1H), 6.46 (d, 2H, J = 8.8), 5.04 (bs, 1H), 5.02 (d, 1H, J = 4.7 Hz), 3.76 (s, 3H). **^{13}C NMR** ($CDCl_3$, 100 MHz): δ 171.4, 163.1 ($^1J_{CF}$ = 247 Hz), 144.1, 139.8 ($^3J_{CF}$ = 6.8 Hz), 130.4 ($^3J_{CF}$ = 8.1 Hz), 129.1, 123.0, 122.8 ($^4J_{CF}$ = 2.6 Hz), 115.5 ($^2J_{CF}$ = 21.2 Hz), 114.5, 114.2 ($^2J_{CF}$ = 22.5 Hz), 60.2, 53.1. **HRMS (MALDI-FT ICR)** m/z: $[M+H]^+$ calculated for $C_{15}H_{14}ClFNO_2$: 294.0692, found: 294.0714. HPLC (IC, 2-propanol/n-hexane = 5/95, flow rate = 1.0 mL/min, l = 220 nm) t_R = 5.6 min (major), 6.1 min (minor).

(*R*)-Methyl 2-((1H-indol-5-yl)amino)-2-(4-ethynylphenyl)acetate 2z

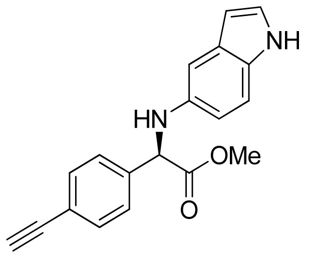

Brown wax (flash chromatography eluent: hexanes/EtOAc, 100/0 to 92/8), 20.1 mg, 66% yield. $[\alpha]_D^{21}$ = -132.5 (C = 0.81, $CHCl_3$), 94% ee. **^{1}H NMR** ($CDCl_3$, 600 MHz): δ 7.94 (bs, 1H), 7.52 (d, 2H, J = 8.3 Hz), 7.49 (d, 2H, J = 8.3 Hz), 7.18 (d, 1H, J = 8.6 Hz), 7.09-7.08 (m, 1H), 6.69 (d, 1H, J = 2.1 Hz), 6.64 (dd, 1H, J_1 = 8.6, 2.1 Hz), 6.33 (s, 1H), 5.30 (s, 1H), 5.13 (s, 1H), 3.74 (s, 3H), 3.08 (s, 1H). **^{13}C NMR**: ($CDCl_3$, 150 MHz): δ 172.3. 139.8, 139.0, 132.5, 130.4, 128.6, 127.3, 124.5, 121.9, 112.4, 111.7, 103.3, 101.9, 83.3, 77.5, 61.9, 52.7. **HRMS (MALDI-FT ICR)** m/z: $[M+H]^+$ calculated for $C_{19}H_{17}N_2O_2$: 305.1284, found: 305.1293. HPLC (IC, 2-propanol/n-hexane = 20/80, flow rate = 1.0 mL/min, l = 254 nm) t_R = 12.8 min (major), 15.9 min (minor).

(*R*)-Methyl 2-(indolin-1-yl)-2-(m-tolyl)acetate 2aa

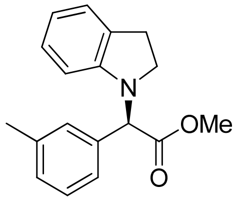

Light yellow oil (flash chromatography eluent: hexanes/EtOAc, 100/0 to 98/2), 16.9 mg, 60% yield. $[\alpha]_D^{20}$ = -91.8 (C = 0.77, $CHCl_3$), 90% ee. **^{1}H NMR** ($CDCl_3$, 600 MHz): δ 7.29-7.26 (m, 1H), 7.22-7.17 (m, 3H), 7.09-7.04 (m, 2H), 6.71-6.69 (m, 1H), 6.45 (d, 1H, J = 7.8 Hz), 5.24 (s, 1H), 3.76 (s, 3H), 3.67-3.63 (m, 1H), 3.17-3.13 (m, 1H), 3.00-2.96 (m, 1H), 2.93-2.87 (m, 1H), 2.38 (s, 3H). **^{13}C NMR**: ($CDCl_3$, 150 MHz): δ 171.8, 150.9, 138.4, 135.0, 130.2, 129.4, 129.1, 128.5, 127.2, 125.7, 124.6, 118.3, 106.7, 63.8, 51.9, 49.8 28.1, 21.4. **HRMS (MALDI-FT ICR)** m/z: $[M+H]^+$ calculated for $C_{18}H_{20}NO_2$: 282.1489, found: 282.1498. HPLC (IC, 2-propanol/n-hexane = 5/95, flow rate = 1.0 mL/min, l = 220 nm) t_R = 5.6 min (major), 6.8 min (minor).

(*R*)-Methyl 4-methyl-2-(phenylamino)pentanoate 2ab

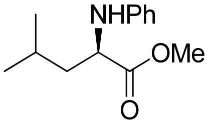

Data for this compound are consistent with those reported in the literature.[13,14]

White solid (flash chromatography eluent: hexanes/EtOAc, 100/0 to 98/2), 15.3 mg, 69% yield. **mp:** 42.7-43.2. $[\alpha]_D^{21}$ = +71.5 (C = 0.23, $CHCl_3$), 94% ee. **^{1}H NMR** ($CDCl_3$, 400 MHz): δ 7.17 (d, 2H, J = 7.4 Hz), 6.74 (m, 1H), 6.62 (d, 2H, J = 8.3 Hz), 4.13-4.07 (dd, 1H, J = 14.7, 7.2 Hz), 3.97 (d, 1H, J = 7.2 Hz), 3.70 (s, 3H), 1.81 (hept, 1H, J = 6.6 Hz), 1.67-1.63 (m, 2H), 0.97 (dd, 6H, J = 18.0, 6.6 Hz). **^{13}C NMR**: ($CDCl_3$, 100 MHz): δ 175.2. 146.9, 129.3, 118.3, 113.4, 55.1, 52.0, 42.4, 24.9, 22.7, 22.2. **HRMS (MALDI-FT ICR)** m/z: $[M+H]^+$ calculated for $C_{13}H_{20}NO_2$: 222.1489, found: 222.1501. HPLC (IC, 2-propanol/n-hexane = 5/95, flow rate = 1.0 mL/min, l = 210 nm) t_R = 5.8 min (major), 6.5 min (minor).

(*R*)-Methyl 2-(phenylamino)nonanoate 2ac

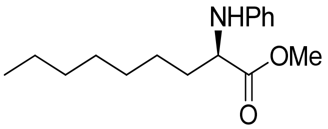

Data for this compound are consistent with those reported in the literature.[14]

Yellow oil (flash chromatography eluent: hexanes/EtOAc, 100/0 to 98/2), 13.4 mg, 51% yield. $[\alpha]_D^{21}$ = +17.6 (C = 0.26, $CHCl_3$) 84% ee. **^{1}H NMR** ($CDCl_3$, 300 MHz): δ 7.19-7.14 (m, 2H), 6.73

[13] King, S.M.; Buchwald, S. L. *Org. Lett.* **2016**, *18*, 4128.
[14] Hobson, C.; Perryman, M. S.; Kirby, G.; Clarkson, G. J.; Fox, D. J. *Tetrahedron Letters* **2018**, *59*, 3965.

(t, 1H, J = 7.2 Hz), 6.61 (d, 2H, J = 8.3 Hz), 4.06 (t, 1H, J = 6.4 Hz), 3.71 (s, 3H), 1.87-1.68 (m, 2H), 1.41-1.27 (m, 10H), 0.87 (t, 3H, J = 6.2 Hz). **^{13}C NMR**: ($CDCl_3$, 75 MHz): δ 174.8. 146.9, 129.3, 118.2, 113.3, 56.6, 52.0, 33.1, 31.7, 29.3, 29.1, 25.6, 22.6, 14.0. **HRMS (MALDI-FT ICR)** m/z: $[M+Na]^+$ calculated for $C_{16}H_{25}NaNO_2$: 286.1777, found: 286.1771. HPLC (OD-H, 2-propanol/n-hexane = 10/90, flow rate = 1.0 mL/min, l = 210 nm) t_R = 7.0 min (major), 5.2 min (minor).

(*R*)-Methyl 2-((3,4-dimethylphenyl)amino)heptanoate 2ad

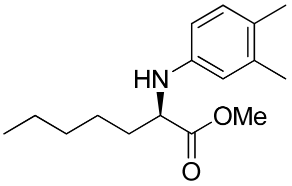

Colourless oil (flash chromatography eluent: hexanes/EtOAc, 100/0 to 98/2), 21.9 mg, 83% yield. $[\alpha]_D^{21}$ = +23.4 (C = 0.57, $CHCl_3$), 76% ee. **^{1}H NMR** ($CDCl_3$, 600 MHz): δ 6.93 (d, 1H, J = 8.0 Hz), 6.46 (d, 1H, J = 2.4 Hz), 6.38 (dd, 1H, J = 8.0, 2.4 Hz), 4.03 (t, 1H, J = 6.5 Hz), 3.92 (bs, 1H), 3.71 (s, 3H), 2.19 (s, 3H), 2.14 (s, 3H), 1.84-1.78 (m, 1H), 1.76-1.70 (m, 1H), 1.43-1.40 (m, 2H), 1.32-1.30 (m, 4H), 0.89 (t, 3H, J = 7.0 Hz). **^{13}C NMR**: ($CDCl_3$, 150 MHz): δ 175.1. 145.0, 137.4, 130.3, 126.3, 115.4, 110.7, 56.9, 52.0, 33.2, 31.5, 25.3, 22.4, 20.0, 18.7, 14.0. **HRMS (MALDI-FT ICR)** m/z: $[M+H]^+$ calculated for $C_{16}H_{26}NO_2$: 264.1958, found: 264.1963. HPLC (OD-H, 2-propanol/n-hexane = 5/95, flow rate = 1.0 mL/min, l = 210 nm) t_R = 5.7 min (major), 6.7 min (minor).

(*R*)-Methyl 2-cyclohexyl-2-(phenylamino)acetate 2ae

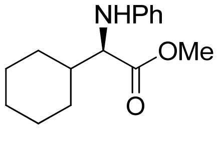

Yellow solid (TLC plate, eluent: hexanes/EtOAc, 80/20), 12.6 mg, 51% yield. **mp:** 50.4-50.8. $[\alpha]_D^{22}$ = 26.4 (C = 0.26, $CHCl_3$), 93% ee. **^{1}H NMR** ($CDCl_3$, 300 MHz): δ 7.19-7.13 (m, 2H), 6.72 (t, 1H, J = 7.3 Hz), 6.61 (d, 2H, J=7.7 Hz), 3.88 (d, 1H, J = 6.0 Hz), 3.70 (s, 3H), 1.87-1.65 (m, 5H), 1.25-1.12 (m, 5H), 0.87-0.85 (m, 1H). **^{13}C NMR**: ($CDCl_3$, 75 MHz): δ 174.2. 147.3, 129.3, 118.1, 113.4, 62.0, 51.8, 41.3, 29.6, 29.2, 26.0. **HRMS (MALDI-FT ICR)** m/z: $[M+H]^+$calculated for $C_{15}H_{22}NO_2$: 248.1645, found: 248.1657. HPLC (IC, 2-propanol/n-hexane = 5/95, flow rate = 1.0 mL/min, l = 210 nm) t_R = 6.2 min (major), 7.0 min (minor).

(*R*)-Methyl 2-cyclopropyl-2-(phenylamino)acetate 2af

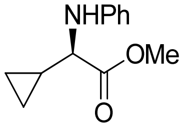

Colourless oil (flash chromatography eluent: hexanes/EtOAc, 100/0 to 98/2), 11.7 mg, 57% yield. $[\alpha]_D^{21}$ = -18,8 (C = 0.67 M, $CHCl_3$), 94% ee. **^{1}H NMR** ($CDCl_3$, 400 MHz): δ 7.18-7.14 (m, 2H), 6.74 (t, 1H, J = 7.3 Hz), 6.59 (d, 2H, J = 7.7 Hz), 4.26 (bs, 1H), 3.75 (s, 3H), 3.59 (d, 1H, J = 7.3 Hz), 1.26-1.18 (m, 1H), 0.59-0.57 (m, 3H), 0.40-0.38 (m, 1H). **^{13}C NMR**: ($CDCl_3$, 100 MHz): δ 174.0. 147.0, 129.3, 118.3, 113.2, 59.8, 52.2, 14.0, 3.0, 2.7. **HRMS (MALDI-FT ICR)** m/z: $[M+H]^+$ calculated for $C_{12}H_{16}NO_2$: 206.1176, found: 206.1179. HPLC (OD-H, 2-propanol/n-hexane = 5/95, flow rate = 1.0 mL/min, l = 220 nm) t_R = 9.2 min (major), 7.4 min (minor).

(*R*)-methyl 2-cyclobutyl-2-(indolin-1-yl)acetate 2ag

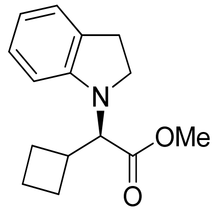

White solid (flash chromatography eluent: hexanes/EtOAc, 100/0 to 98/2), 16.9 mg, 69% yield. **mp:** 51.0-51.6. $[\alpha]_D^{22}$ = +87,9 (C = 0.83 M, $CHCl_3$), 94% ee. **^{1}H NMR** ($CDCl_3$, 400 MHz): δ 7.06-7.02 (m, 2H), 6.65-6.61 (m, 1H), 6.44 (d, 1H, J = 7.8 Hz), 4.09 (d, 1H, J = 10.7 Hz), 3.69-3.62 (m, 1H), 3.63 (s, 3H), 3.50-3.44 (m, 1H), 3.00-2.95 (m, 2H), 2.90-2.85 (m, 1H), 2.16-2.14 (m, 2H), 1.96-1.92 (m, 4H). **^{13}C NMR**: ($CDCl_3$, 100 MHz): δ 172.5. 151.0, 129.6, 127.2, 124.4, 117.5, 106.0, 63.7, 51.3, 48.2, 35.4, 28.3, 26.5, 25.9, 18.1. **HRMS (MALDI-FT ICR)** m/z: $[M+H]^+$ calculated for $C_{15}H_{20}NO_2$: 246.1489, found: 246.1497. HPLC (OD-H, 2-propanol/n-hexane = 5/95, flow rate = 0.6 mL/min, l = 220 nm) t_R = 8.1 min (major), 8.4 min (minor).

General procedure for one-pot asymmetric Knoevenagel/epoxidation/ring-opening/esterification reaction and epoxidation/ring-opening/esterification reaction to arylglycines (*S*)-2b, (*S*)-2f, (*S*)-2l, (*S*)-2z, (S)-2ae, (S)-2af

In an ace tube containing (1-naphtylsulfonyl)acetonitrile (23.1 mg, 0.10 mmol, 1 eq.) and catalyst **4** (7.8 mg, 0.010 mmol, 0.1 eq.) in anhydrous toluene (0.35 mL, C = 0.3 M), the opportune aldehyde (0.10 mmol, 1 eq.) was added. The reaction was stirred in an oil bath at 30 °C for 23-40 hours, monitored by TLC (eluent PE/ethyl acetate 8/2). After completion, the mixture was diluted with toluene (5 mL, C = 0.02 M) and 1.1 equivalents of cumene hydroperoxide (tech. 80%, 20 µL, 0.11 mmol, 1.1 eq.) were added at –20 °C. The solution was stirred at -20 °C until consumption of the alkene **3** (TLC eluent PE/ ethyl acetate 8/2). Then the aniline or 5-aminoindole (0.12 mmol, 1.2

eq.), methanol (405 μL, 10 mmol, 100 eq.) and N,N-dicyclohexylmethylamine (33 μL, 0.15 mmol, 1.5 eq.) were added and the reaction mixture was stirred for 8-9 hours at room temperature, monitored by TLC (eluent PE/ ethyl acetate 8/2).

For the synthesis of compounds **2ae** and **2af,** in an ace tube containing the alkene **3** (0.10 mmol, 1 eq.) and catalyst **4** (7.8 mg, 0.010 mmol, 0.1 eq.) in anhydrous toluene (5 mL, C = 0.02 M), CHP (tech. 80%, 20 μL, 0.11 mmol, 1.1 eq.) was added and the solution was stirred for 13 days at -20 °C for product **2af** and 4 days at -40 °C for product **2ae**, until consumption of the alkene **3** (TLC eluent PE/ethyl acetate 8/2). Then aniline (11 μL, 0.12 mmol, 1.2 eq.), methanol (405 μL, 10 mmol, 100 eq.) and N,N-dicyclohexylmethylamine (33 μL, 0.15 mmol, 1.5 eq.) were added and the reaction mixture was stirred in an oil bath for 5 hours at 60 °C for product **2af** and 230 hours at 70 °C for product **2ae**, monitored by TLC (eluent PE/ethyl acetate 8/2). After completion, the mixture was diluted with ethyl acetate (10 mL) and washed with saturated NH_4Cl solution (2x10 mL) and water (1x10 mL), dried over anhydrous Na_2SO_4 and concentrated under vacuum. The crude mixture was purified by flash chromatography (eluent: hexane/ethyl acetate 100/0 to 70/30) to give the final products in 41-73% yield and 60-85% ee.

(*S*)-Methyl 2-phenyl-2-(phenylamino)acetate 2b

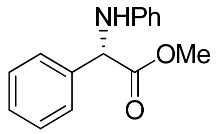

Data for this compound are consistent with those reported in the literature.[7,8]

Colourless oil (flash chromatography eluent: hexanes/EtOAc, 100/0 to 98/2), 12.1 mg, 50% yield. $[\alpha]_D^{21}$= +70.9 (C = 0.77, $CHCl_3$), 82% ee. HPLC (IC, 2-propanol/n-hexane = 5/95, flow rate = 1.0 mL/min, l = 220 nm) t_R = 6.8 min (major), 6.3 min (minor).

(*S*)-Methyl 2-(4-bromophenyl)-2-(phenylamino)acetate 2f

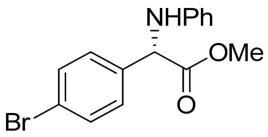

Data for this compound are consistent with those reported in the literature.[10]

Yellow oil (flash chromatography eluent: hexanes/EtOAc, 100/0 to 98/2), 20.2 mg, 63% yield. $[\alpha]_D^{21}$= +89.7 (C = 0.93, $CHCl_3$), 85% ee. HPLC (IE-3, 2-propanol/n-hexane = 5/95, flow rate = 0.8 mL/min, l = 220 nm) t_R = 10.5 min (major), 9.7 min (minor).

(*S*)-Methyl 2-(3,4-dichlorophenyl)-2-(phenylamino)acetate 2l

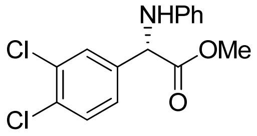

Colourless oil (flash chromatography eluent: hexanes/EtOAc, 100/0 to 98/2), 20.8 mg, 67% yield. $[\alpha]_D^{20}$= +63.5 (C = 0.37, $CHCl_3$), 80% ee. HPLC (IC, 2-propanol/n-hexane = 5/95, flow rate = 1.0 mL/min, l = 220 nm) t_R = 6.1 min (major), 5.3 min (minor).

(*S*)-Methyl 2-((1H-indol-5-yl)amino)-2-(4-ethynylphenyl)acetate 2z

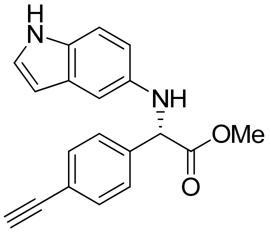

Brown wax (flash chromatography eluent: hexanes/EtOAc, 100/0 to 92/8), 16.4 mg, 54% yield. $[\alpha]_D^{21}$= +91.1 (C = 0.42, $CHCl_3$), 82% ee. HPLC (IC, 2-propanol/n-hexane = 20/80, flow rate = 1.0 mL/min, l = 254 nm) t_R = 16.2 min (major), 13.2 min (minor).

(*S*)-Methyl 2-cyclohexyl-2-(phenylamino)acetate 2ae

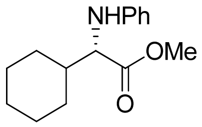

Yellow solid (TLC plate, eluent: hexanes/EtOAc, 80/20), 10.1 mg, 41% yield. **mp:** 50.4-50.8. $[\alpha]_D^{21}$= -25.1 (C = 0.49, $CHCl_3$), 73% ee. HPLC (IC, 2-propanol/n-hexane = 5/95, flow rate = 1.0 mL/min, l = 210 nm) t_R = 6.1 min (major), 5.6 min (minor).

(*S*)-Methyl 2-cyclopropyl-2-(phenylamino)acetate 2af

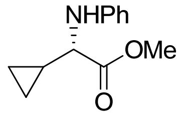

Colourless oil (flash chromatography eluent: hexanes/EtOAc, 100/0 to 98/2), 15.0 mg, 73% yield. $[\alpha]_D^{21}$= +9.1 (C = 0.37, $CHCl_3$), 65% ee. HPLC (OD-H, 2-propanol/n-hexane = 5/95, flow rate = 1.0 mL/min, l = 220 nm) tR = 7.8 min (major), 10.3 min (minor).

General procedure for the synthesis of alkenes 3b', 3b''

Alkenes **3b'** and **3b''** were prepared according to the literature.[1]

2-(Naphthalene-1-ylsulfonyl)acetonitrile or 2-(naphthalene-2-ylsulfonyl)acetonitrile (0.56 mmol, 1 eq.) and appropriate benzaldehyde (0.56 mmol, 1 eq.) were dissolved in anhydrous ethanol (2.8 mL, C = 0.2 M) and diethylamine (18 mL, 0.11 mmol, 0.2 eq.) was added. The reaction mixture was

stirred at room temperature for 4-7 hours, monitored by TLC (eluent PE/ethyl acetate 7/3). Upon completion, water was added, and the mixture was extracted with ethyl acetate (2x30 mL). The combined organic layers were dried over Na_2SO_4, filtered and concentrated under vacuum. The crude alkenes were then purified by flash chromatography (eluting from PE/ethyl acetate 100 to 80/20) and were isolated in 82-83% yield.

General procedure for the synthesis of alkenes 3af, 3ag, 3ae', 3af'

Alkenes **3af, 3ag, 3ae'** and **3af'** were prepared according to the literature.[1]

To a suspension of (phenylsulfonyl)acetonitrile or 2-(naphthalene-1-ylsulfonyl)acetonitrile (0.65 mmol, 1 eq.) and the appropriate aldehyde (0.65 mmol, 1 eq.) in toluene (2.5 mL, C = 0.26 M), 3 Å molecular sieves (200 mg), piperidine (13 µL, 0.13 mmol, 0.2 eq.) and acetic acid (12 µL, 0.21 mmol, 0.3 eq.) were added. The resulting mixture was heated in an oil bath at 50 °C for 5-63 h. Then, the reaction was cooled to room temperature and water was added. The mixture was extracted with ethyl acetate (4x30 mL) and the combined organic layers were dried over Na_2SO_4, filtered, and concentrated under vacuum. The crude reaction mixture was then purified by flash chromatography (eluting from PE/ethyl acetate 100/0 to 90/10) affording alkenes in 22-37% yield.

(*E*)-3-Cyclopropyl-2-(phenylsulfonyl)acrylonitrile 3af

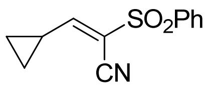

White solid (flash chromatography eluent: hexanes/EtOAc, 100/0 to 90/10), 42.5 mg, 28% yield. **mp**: 109.3-110.2. **^{1}H NMR** ($CDCl_3$, 600 MHz): δ 7.94 (dd, 2H, J = 8.5, 1.2 Hz), 7.70 (t, 1H, J = 7.5 Hz), 7.61-7.58 (m, 2H), 6.99 (d, 1H, J = 11.4 Hz), 2.02-1.96 (m, 1H), 1.38-1.35 (m, 2H), 1.06-1.03 (m, 2H). **^{13}C NMR** ($CDCl_3$, 150 MHz): δ 165.4, 138.2, 134.4, 129.6, 128.3, 116.6, 111.9, 15.9, 12.0 (2C). **HRMS (MALDI-FT ICR)** m/z: $[M]^+$ calculated for $C_{12}H_{11}NO_2S$: 233.0511, found: 233.0515.

(*E*)-3-Cyclobutyl-2-(phenylsulfonyl)acrylonitrile 3ag

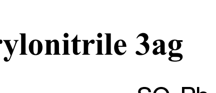

Colourless liquid (flash chromatography eluent: hexanes/EtOAc, 100/0 to 90/10), 35,3 mg, 22% yield. **^{1}H NMR** ($CDCl_3$, 400 MHz): δ 7.94 (d, 2H, J = 7.9 Hz), 7.72 (d, 1H, J = 9.5 Hz), 7.71-7.68 (m, 1H), 7.61-7.57 (m, 2H), 3.49-3.43 (m, 1H), 2.35-2.33 (m, 2H), 2.13-2.05 (m, 3H), 2.03-1.97 (m, 1H). **^{13}C NMR** ($CDCl_3$, 100 MHz): δ 162.7, 137.7, 134.6, 129.6, 128.5, 118.1, 111.2, 36.9, 27.8 (2C), 19.0. **HRMS (MALDI-FT ICR)** m/z: $[M+Na]^+$ calculated for $C_{13}H_{13}NaNO_2S$: 270.0559, found: 270.0600.

(*E*)-2-(Naphthalen-1-ylsulfonyl)-3-phenylacrylonitrile 3b'

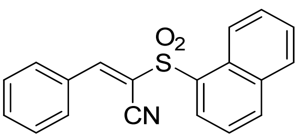

White solid (flash chromatography eluent: hexanes/EtOAc, 100/0 to 90/10), 146 mg, 82% yield. **mp**. = 130.2-130.9. **^{1}H NMR** ($CDCl_3$, 600 MHz): δ 8.63 (d, 1H, J = 8.6 Hz), 8.55 (d, 1H, J = 7.4 Hz), 8.44 (s, 1H), 8.21 (d, 1H, J=8.2), 7.99 (d, 1H, J = 8.2 Hz), 7.93 (d, 2H, J=7.7 Hz), 7.70-7.67 (m, 2H), 7.63-7.61 (m, 1H), 7.58-7.56 (m, 1H), 7.50-7.47 (m,2H). **^{13}C NMR:** ($CDCl_3$, 150 MHz): δ 151.6, 136.6, 134.3, 134.0, 132.7, 131.8, 130.9, 130.1, 129.5, 129.4, 129.0, 128.3, 127.2, 124.6, 123.6, 115.0, 112.8. **HRMS (MALDI-FT ICR)** m/z: $[M+Na]^+$ calculated for $C_{19}H_{13}NaNO_2S$: 342.0559, found: 342.0580.

(*E*)-2-(Naphthalen-2-ylsulfonyl)-3-phenylacrylonitrile 3b''

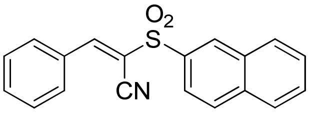

White solid (flash chromatography eluent: hexanes/EtOAc, 100/0 to 90/10), 148 mg, 83% yield. **mp**. = 118.9-119.4. **^{1}H NMR** ($CDCl_3$, 400 MHz): δ 8.65 (s, 1H), 8.30 (s, 1H), 8.06-8.03 (m, 2H), 7.96-7.92 (m, 4H), 7.74-7.65 (m, 2H), 7.61-7.57 (m, 1H), 7.53-7.49 (m, 2H). **^{13}C NMR:** ($CDCl_3$, 100 MHz): δ 151.6, 135.8, 134.7, 134.2, 132.3, 131.1 (3C), 130.3, 130.2, 130.0, 129.8, 129.6, 128.1, 128.0, 122.7, 114.9, 113.2. **HRMS (MALDI-FT ICR)** m/z: $[M+Na]^+$ calculated for $C_{19}H_{13}NaNO_2S$: 342.0559, found: 342.0578.

(*E*)-3-Cyclohexyl-2-(naphthalen-1-ylsulfonyl)acrylonitrile 3ae'

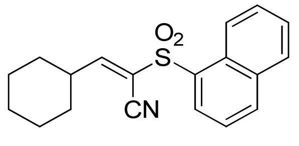

Light brown oil (flash chromatography eluent: hexanes/EtOAc, 100/0 to 90/10), 77.6 mg, 37% yield. **^{1}H NMR** ($CDCl_3$, 300 MHz): δ 8.48-8.43 (m, 2H), 8.19 (d, 1H, J = 8.1 Hz), 7.99 (d, 1H, J = 7.9 Hz), 7.72-7.62 (m, 4H), 2.60-2.58 (m, 1H), 1.76-1.68 (m, 5H), 1.34-1.27 (m, 5H). **^{13}C NMR:** ($CDCl_3$, 75 MHz): δ 164.5, 136.5, 134.2, 132.5, 131.6, 129.5, 128.8, 128.0, 127.2, 124.6, 123.4, 118.6, 111.0, 41.3, 31.1 (2C), 25.1, 24.6 (2C). **HRMS (MALDI-FT ICR)** m/z: $[M+H]^+$ calculated for $C_{19}H_{20}NO_2S$: 325.1137, found: 325.1140.

(*E*)-3-Cyclopropyl-2-(naphthalen-1-ylsulfonyl)acrylonitrile 3af'

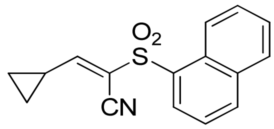

Light brown solid (flash chromatography eluent: hexanes/EtOAc, 100/0 to 90/10), 41.7 mg, 23% yield. **mp**. = 143.3-144.3. **^{1}H NMR** ($CDCl_3$, 400 MHz): δ 8.53 (d, 1H, J = 8.5 Hz), 8.45 (d, 1H, J = 7.4 Hz), 8.18 (d, 1H, J = 8.1 Hz), 7.99 (d, 1H, J = 7.9 Hz), 7.70-7.61 (m, 3H), 7.17 (d, 1H, J = 11.3 Hz), 2.00-1.97 (m, 1H), 1.36-1.35 (m, 2H), 1.06-1.05 (m, 2H). **^{13}C NMR:** ($CDCl_3$, 100 MHz): δ 165.8, 136.4, 134.4, 132.6, 132.2, 129.5, 128.9, 128.3, 127.3, 124.7, 123.7, 116.9, 111.9, 16.1, 12.1 (2C). **HRMS (MALDI-FT ICR)** m/z: $[M+H]^+$ calculated for $C_{16}H_{14}NO_2S$: 284.0740, found: 284.0747.

General procedure for the synthesis of aminoalcohols 5a-c from arylglycines 2

Products **5a-c** was prepared according to a slightly modified literature procedure.[15]

In a round-bottom flask containing a stir bar, the arylglycine (0.1 mmol, 1 eq.) was added. The flask was evacuated, backfilled with nitrogen, and capped with a septum. Then, anhydrous DCM (0.7 mL, C = 0.15 M) was added. The solution was stirred and cooled to -20 °C. Upon cooling, DIBAL-H (1M in heptane, 0.4 mL, 4 eq.) was added. The resulting solution was stirred for 1 hour until consumption of starting material, monitored by TLC (eluent PE/ethyl acetate 70/30). Upon completion, MeOH was added dropwise at 0 °C to quench unreacted DIBAL-H. The reaction was warmed to room temperature and then a saturated solution of sodium potassium tartrate was added (0.3 mL, 0,15mmol, 1.5 equiv.) and the reaction was stirred vigorously for 5 hours. The mixture was then extracted with ethyl acetate (3x15 mL) and the combined organic layers were washed with brine (3 x 15 mL) and dried over Na_2SO_4, filtered and concentrated under vacuum. The crude reaction mixture was then purified by flash chromatography (eluting from PE/ethyl acetate 90/10 to 60/40), affording aminoalcohols **5a-c** in 61-70% yield.

(*R*)-2-(Phenylamino)-2-(*m*-tolyl)ethanol 5a

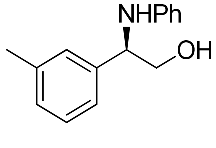

Data for this compound are consistent with those reported in the literature.[16]

Yellow oil (flash chromatography eluent: hexanes/EtOAc, 90/10 to 60/40), 14.3 mg, 63% yield. $[\alpha]_D^{21}$ = -15.5 (C = 0.51, $CHCl_3$), 92% ee. **^{1}H NMR** ($CDCl_3$, 600 MHz): δ 7.25-7.22 (m, 1H), 7.18 (s, 1H), 7.17 (d, 1H, J = 9.1 Hz), 7.13-7.10 (m, 2H), 7.08 (d, 1H, J = 7.5 Hz), 6.69 (t, 1H, J = 7.3

[15] Arredondo, V.; Hiew, S. C.; Gutman, E. S.; Premachandra, I. D. U. A.; Van Vranken, D. L. *Angew. Chem. Int. Ed.* **2017**, *56*, 4156.
[16] Guo, Z.; Xie, J.; Hu, T.; Chen, Y.; Tao, H.; Yang, X. *Chem. Commun.* **2021**, *57*, 9394.

Hz), 6.59 (d, 2H, J = 7.7), 4.48 (dd, 1H, J = 6.9, 4.2 Hz), 3.94 (dd, 1H, J = 11.1, 4.2 Hz), 3.76 (dd, J = 11.1, 6.9 Hz), 2.34 (s, 3H). **^{13}C NMR**: ($CDCl_3$, 150 MHz): δ 147.3. 140.1, 138.5, 129.1, 128.7, 128.4, 127.3, 123.8, 117.8, 113.8, 67.4, 59.9, 21.5. **HRMS (MALDI-FT ICR)** m/z: $[M+H]^+$ calculated for $C_{15}H_{18}NO$: 228.1383, found: 228.1391. HPLC (IA-3, 2-propanol/n-hexane = 10/90, flow rate = 0.8 mL/min, l = 210 nm) t_R = 11.5 min (major), 9.8 min (minor).

(*R*)-2-(4-Bromophenyl)-2-(phenylamino)ethan-1-ol 5b

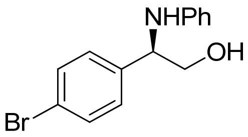

Data for this compound are consistent with those reported in the literature.[16]

Yellow oil (flash chromatography eluent: hexanes/EtOAc, 90/10 to 60/40), 17.8 mg, 61% yield. $[\alpha]_D^{23}$ = -21.8 (C = 0.71, $CHCl_3$), 92% ee. **^{1}H NMR** ($CDCl_3$, 600 MHz): δ 7.47 (d, 2H, J = 8.3 Hz), 7.26 (d, 2H, J = 8.3 Hz), 7.13-7.10 (m, 2H), 6.71 (t, 1H, J = 7.3 Hz), 6.54 (d, 2H, J = 7.8 Hz), 4.46 (dd, 1H, J = 7.0, 4.1 Hz), 3.93 (dd, 1H, J = 11.1, 4.1 Hz), 3.73 (dd, 1H, J = 11.1, 7.0 Hz). **^{13}C NMR**: ($CDCl_3$, 150 MHz): δ 146.8. 139.2, 131.9, 129.2, 128.5, 121.4, 118.2, 113.9, 67.1, 59.4. **HRMS (MALDI-FT ICR)** m/z: $[M+H]^+$ calculated for $C_{14}H_{15}BrNO$: 219.0259, found: 219.0263. HPLC (IA-3, 2-propanol/n-hexane = 10/90, flow rate = 0.8 mL/min, l = 210 nm) t_R = 13.7 min (major), 12.1 min (minor).

(*R*)-2-Cyclopropyl-2-(phenylamino)ethanol (5c)

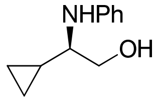

Yellow wax (flash chromatography eluent: hexanes/EtOAc, 90/10 to 60/40), 12.4 mg, 70% yield. $[\alpha]_D^{21}$ = -15.8 (C = 0.36, $CHCl_3$), 93% ee. **^{1}H NMR** ($CDCl_3$, 600 MHz): δ 7.18-7.16 (m, 2H), 6.73 (t, 1H, J=7.3 Hz), 6.67 (dd, 2H, J = 8.5, 0.9 Hz), 3.85 (dd, 1H, J = 11.0, 4.3 Hz), 3.64 (dd, 1H, J = 11.0, 5.5 Hz), 2.91 (ddd, 1H, J = 8.1, 5.5, 4.3 Hz), 1.00-0.95 (m, 1H), 0.57-0.50 (m, 2H), 0.33-0.30 (m, 2H). **^{13}C NMR**: ($CDCl_3$, 150 MHz): δ 148.0. 129.3, 118.0, 113.9, 64.9, 59.9, 13.7, 2.8, 2.7. **HRMS (MALDI-FT ICR)** m/z: $[M+H]^+$ calculated for $C_{11}H_{16}NO$: 178.1226, found: 178.1232. HPLC (AD-H, 2-propanol/n-hexane = 5/95, flow rate = 1.0 mL/min, l = 210 nm) t_R = 13.9 min (major), 16.4 min (minor).

Procedure for the deprotection of arylglycine 2v to product 6a

Products **6a** was prepared according to a slightly modified literature procedure.[8]

In a sample vial containing a solution of arylglycine **2v** (31.3 mg, 0.1 mmol, 1 eq.) in MeCN:H_2O (1:1, 7.7 mL, C = 0.013 M) was added periodic acid (22.2 mg, 0.1 mmol, 1 eq.) and 1M H_2SO_4 (0.1 mL, 0.1 mmol, 1 eq.) at 0° C, then the reaction mixture was gradually warmed up to room

temperature and stirred for 4 hours. The reaction mixture was washed with CH_2Cl_2 (2 x 10 mL), then the aqueous layer was retained, basified with a saturated solution of $NaHCO_3$ and extracted with ethyl acetate (3 x 15 mL), dried over anhydrous Na_2SO_4 and concentrated under vacuum to give the final product **6a** in 54% yield.

(*R*)-Methyl 2-amino-2-(4-isopropylphenyl)acetate 6a

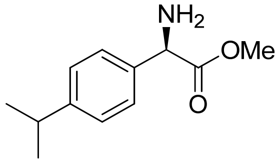

Light yellow oil, 11.2 mg, 54% yield. $[\alpha]_D^{19}$ = -21.6 (C = 0.78, CH_2Cl_2), 80% ee. **^{1}H NMR** ($CDCl_3$, 600 MHz): δ 7.29 (d, 2H, J=8.1 Hz), 7.22 (d, 2H, J = 8.1 Hz), 4.61 (s, 1H), 3.71 (s, 3H), 2.90 (hept, 1H, J = 6.9 Hz), 2.07 (bs, 2H), 1.24 (d, 6H, J = 6.9 Hz). **^{13}C NMR**: ($CDCl_3$, 150 MHz): δ 174.6. 148.8, 137.6, 126.9, 126.7, 58.4, 52.4, 33.8, 23.9. **HRMS (MALDI-FT ICR)** m/z: $[M+H]^+$ calculated for $C_{12}H_{18}NO_2$: 207.1254, found: 206.1258. HPLC (AS-H, 2-propanol/n-hexane = 20/80, flow rate = 1.0 mL/min, l = 220 nm) t_R = 6.6 min (major), 5.4 min (minor).

Scale-up of the one-pot asymmetric Knoevenagel/epoxidation/ring-opening/esterification reaction to arylglycine 2f

In a round bottomed flask containing a solution of (phenylsulfonyl)acetonitrile (184.9 mg, 1.0 mmol, 1 eq.) and **eQNU** (58.6 mg, 0.10 mmol, 0.1 eq.) in anhydrous toluene (3.3 mL, C = 0.3 M), 4-bromobenzaldehyde (187 mg, 1 mmol, 1 eq.) was added. The reaction was stirred at room temperature for 26 hours, monitored by TLC (eluent PE/ethyl acetate 80/20). After completion, the solution was diluted with additional toluene (46.7 mL, C = 0.02 M) and 1.1 equivalents of cumene hydroperoxide (tech. 80%, 203 μL, 1.1 mmol) were added at – 20 °C. The mixture was stirred at -20 °C for 18 hours, monitored by TLC (eluent PE/ethyl acetate 80/20). Then aniline (110 μL, 1.2 mmol, 1.2 eq.), methanol (4.2 mL, 100 mmol, 100 eq.) and N,N-dicyclohexylmethylamine (330 μL, 1.5 mmol, 1.5 eq.) were added and the reaction mixture was stirred at room temperature for 9 hours, monitored by TLC (eluent PE/ ethyl acetate 8/2). After completion, the mixture was diluted with ethyl acetate (100 mL) and washed with saturated NH_4Cl solution (2x80 mL) and water (1x80 mL), dried over anhydrous Na_2SO_4 and concentrated under vacuum. The reaction mixture was purified by flash chromatography (eluent: hexane/ethyl acetate 100/0 to 98/2) to give the final products in 77% yield (246.5 mg) and 93% ee.

NMR Spectra

^{1}H NMR $CDCl_3$ (300 MHz)

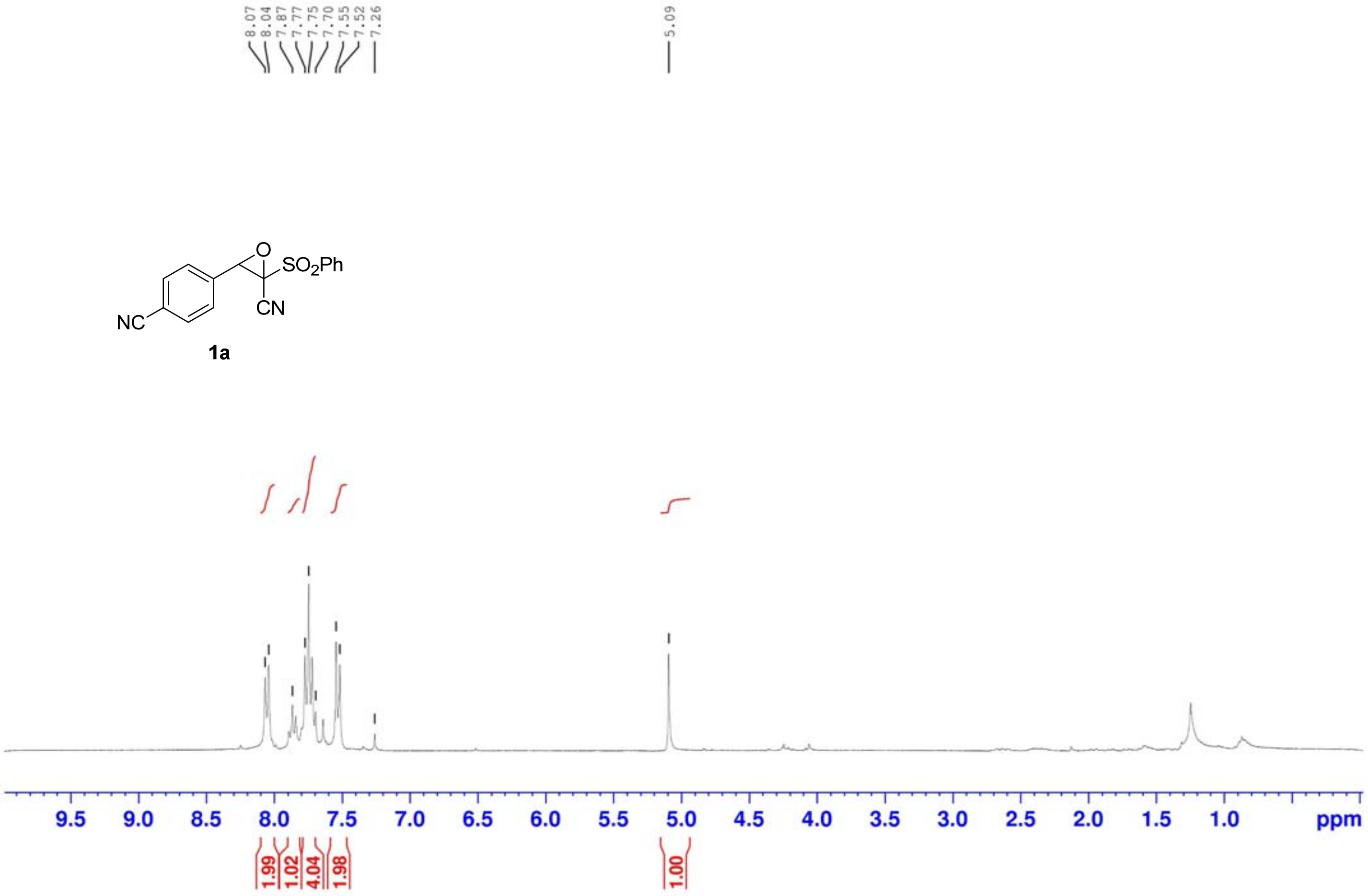

^{13}C NMR $CDCl_3$ (75 MHz)

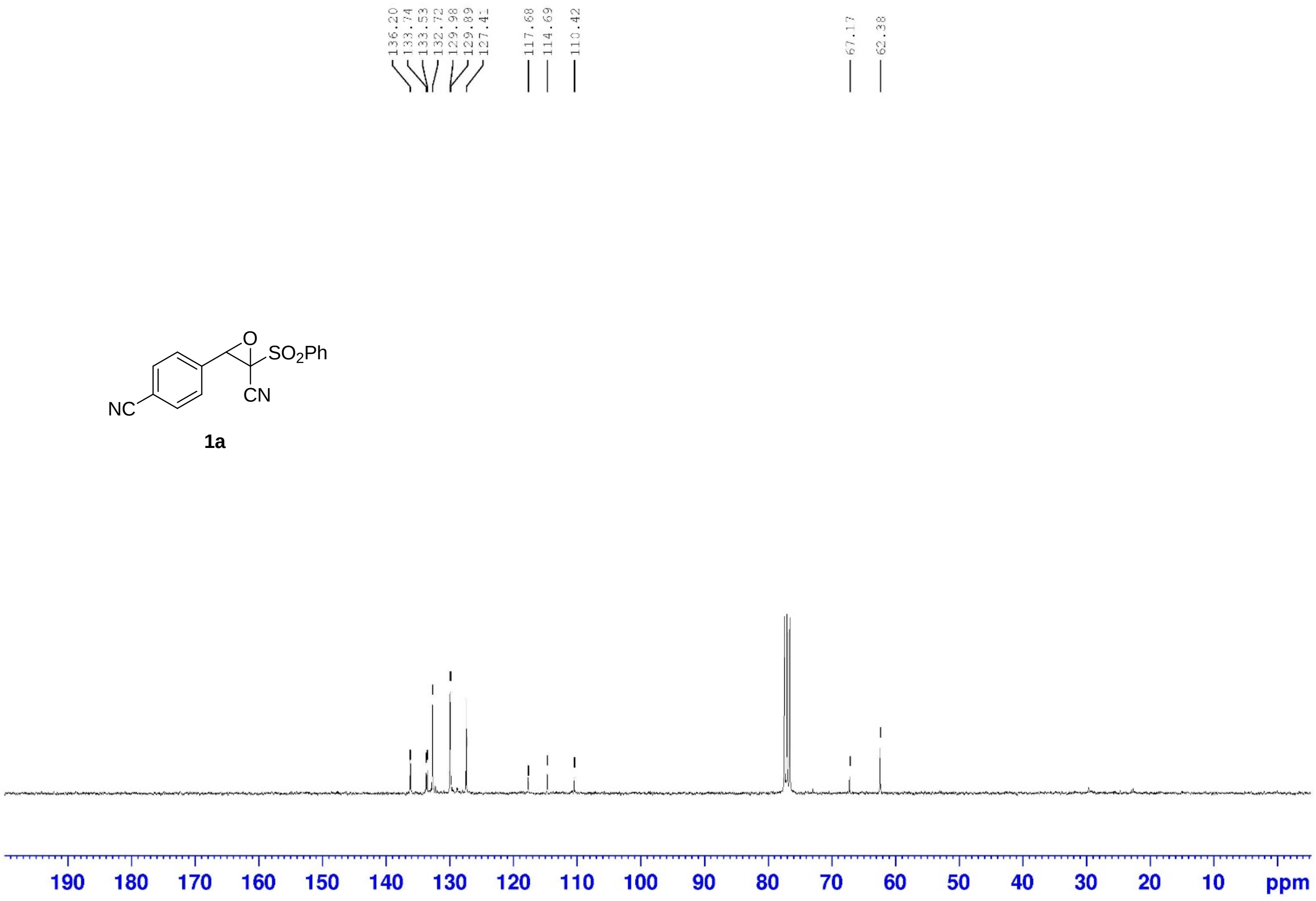

^{1}H NMR $CDCl_3$ (300 MHz)

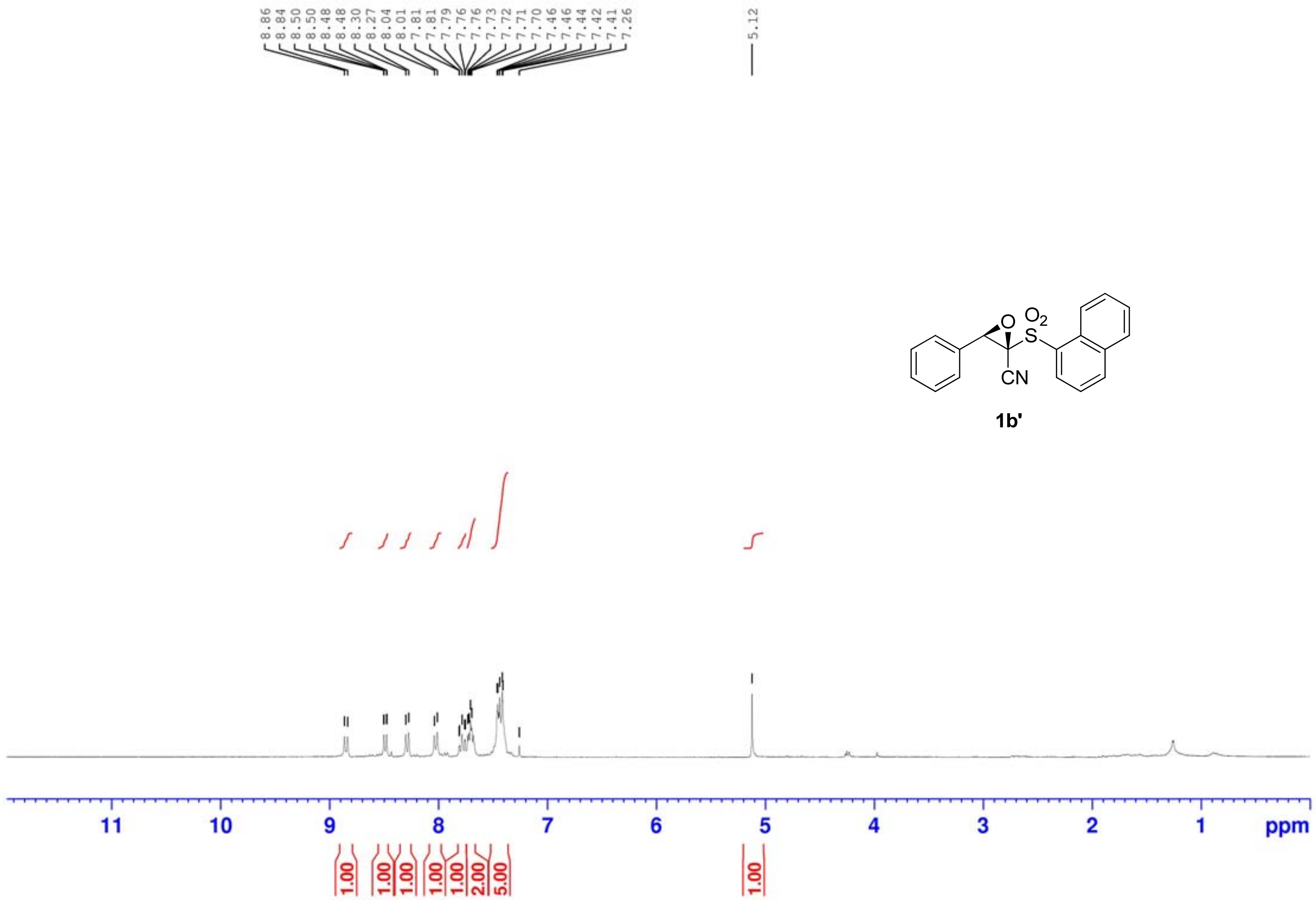

1b'

^{13}C NMR $CDCl_3$ (150 MHz)

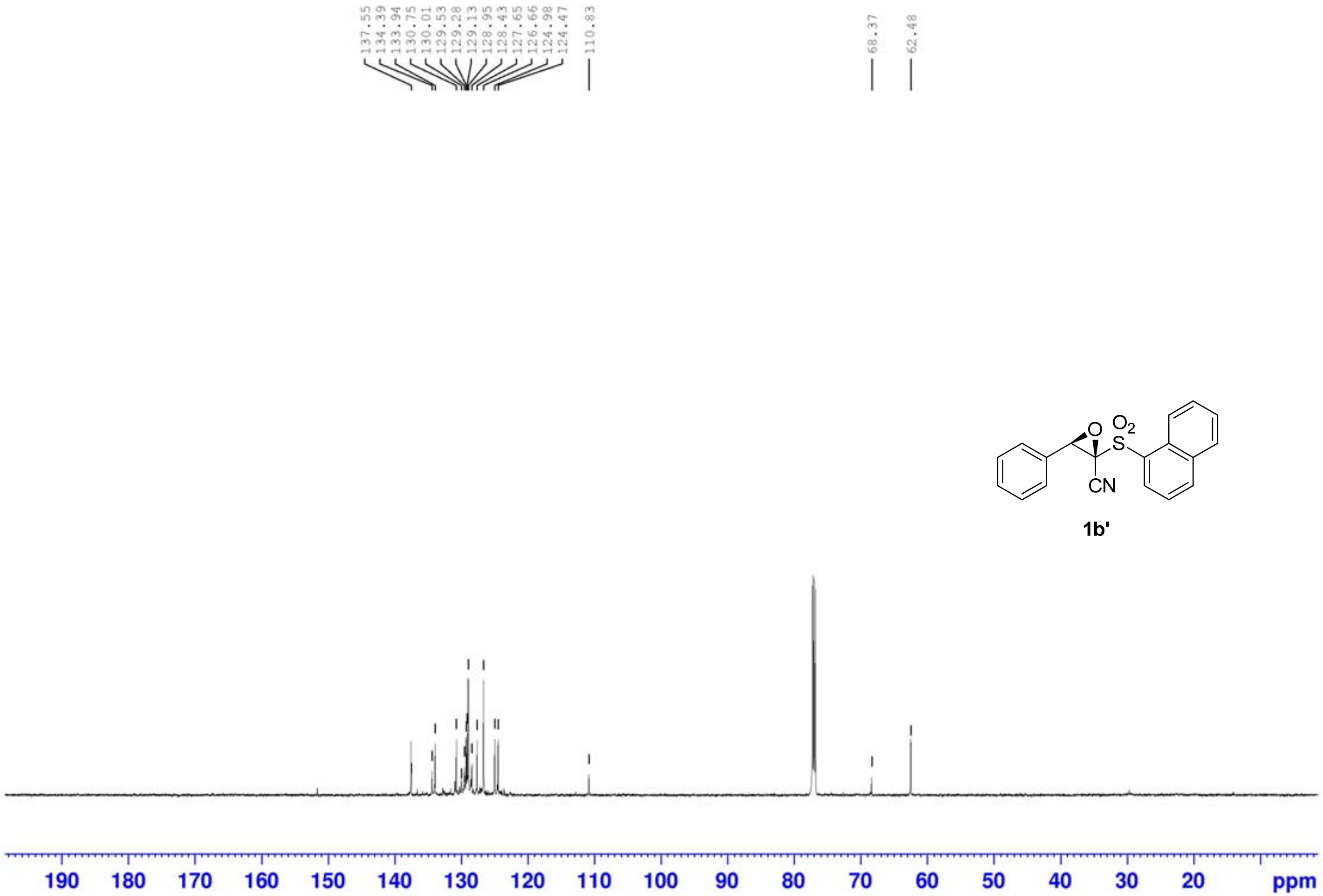

^{1}H NMR $CDCl_3$ (600 MHz)

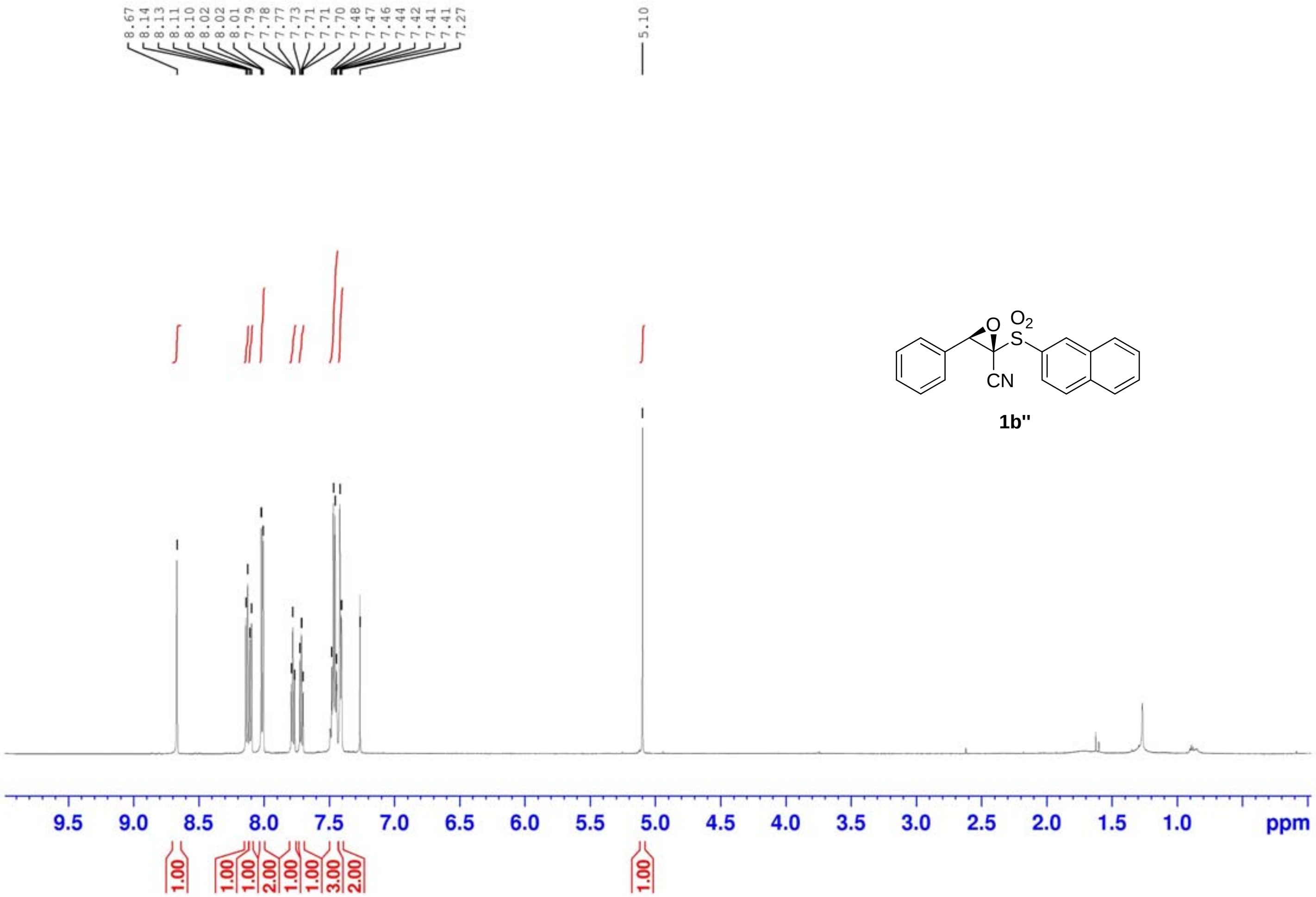

1b''

^{13}C NMR $CDCl_3$ (150 MHz)

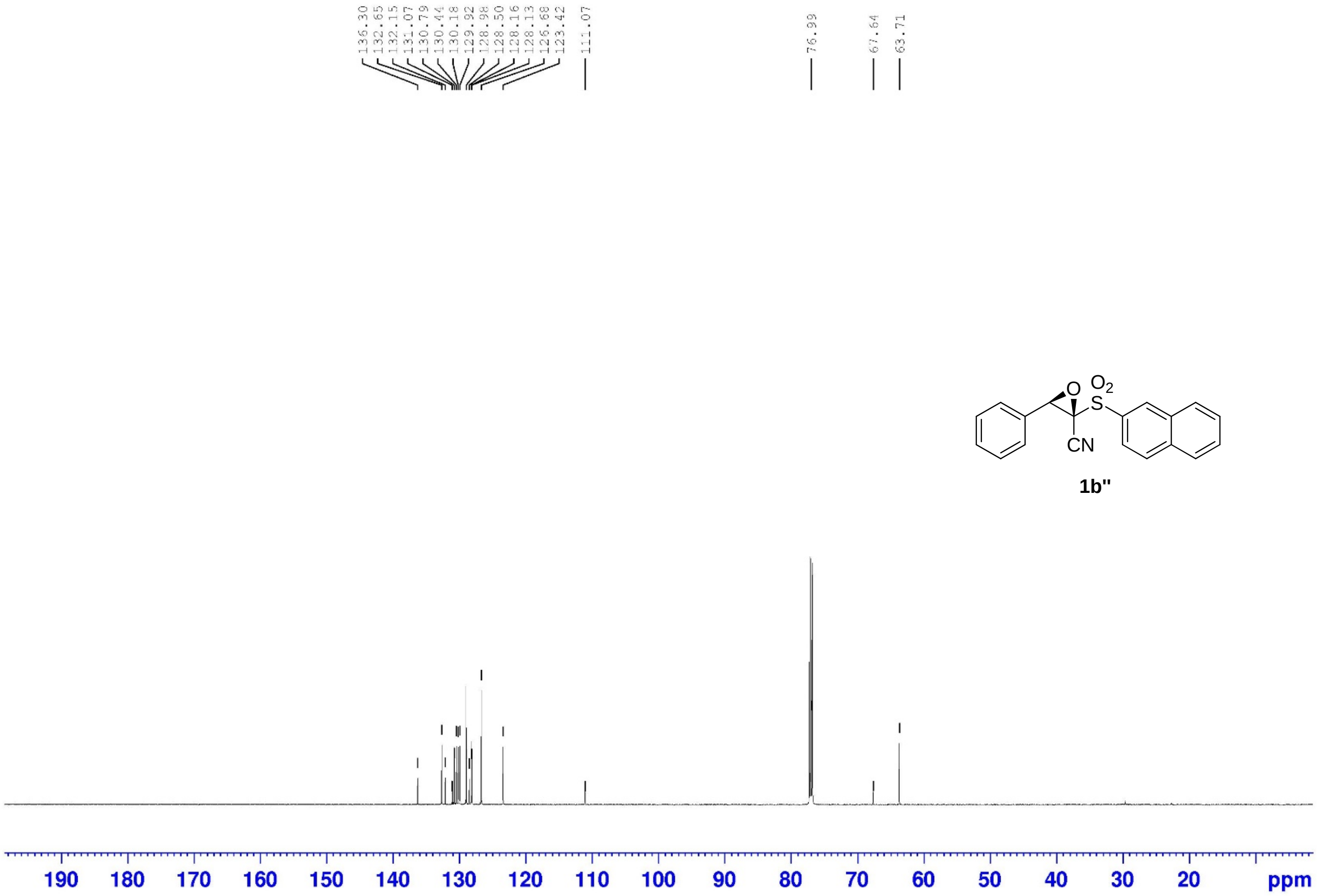

^{1}H NMR $CDCl_3$ (400 MHz)

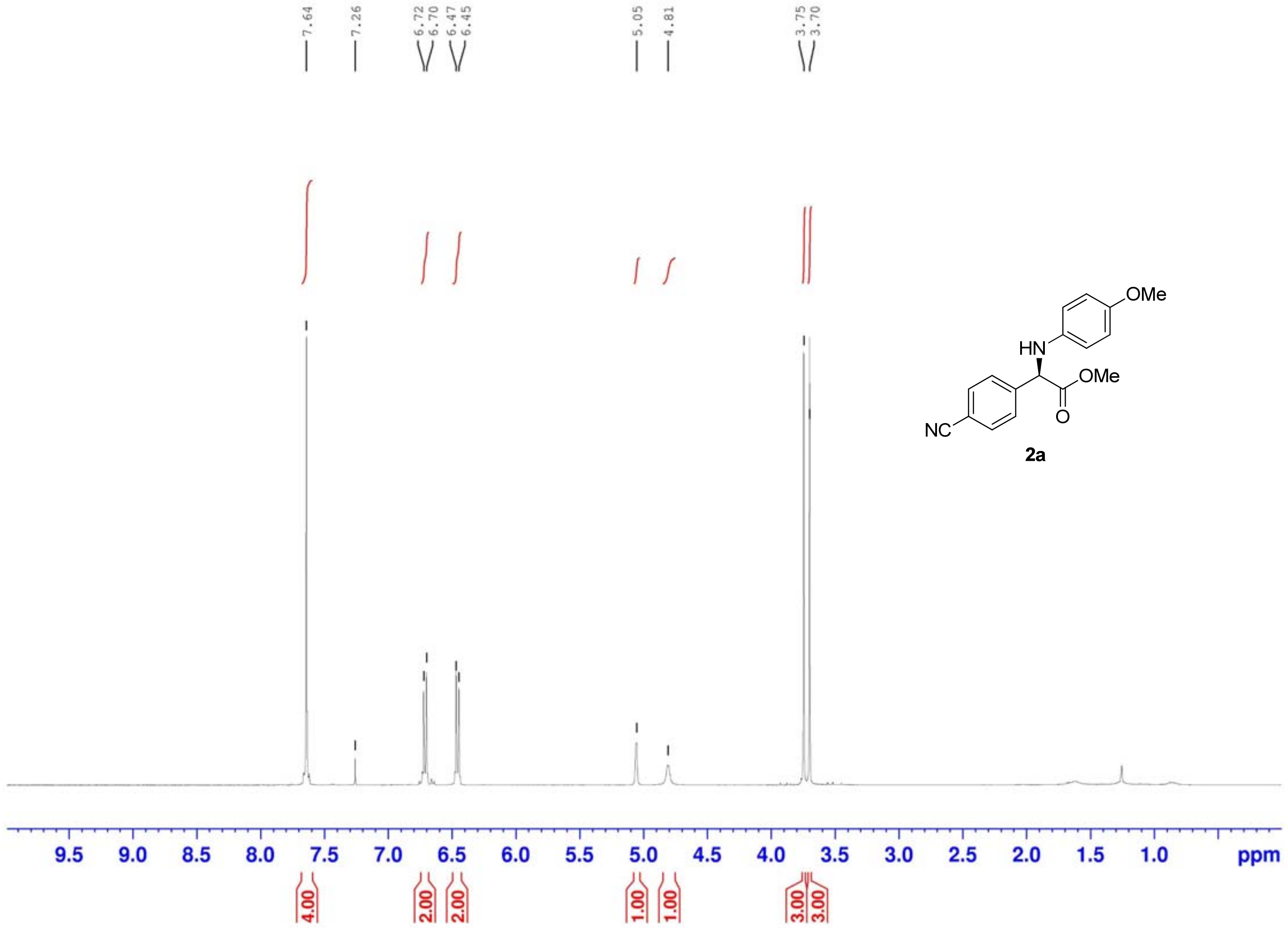

2a

^{13}C NMR $CDCl_3$ (100 MHz)

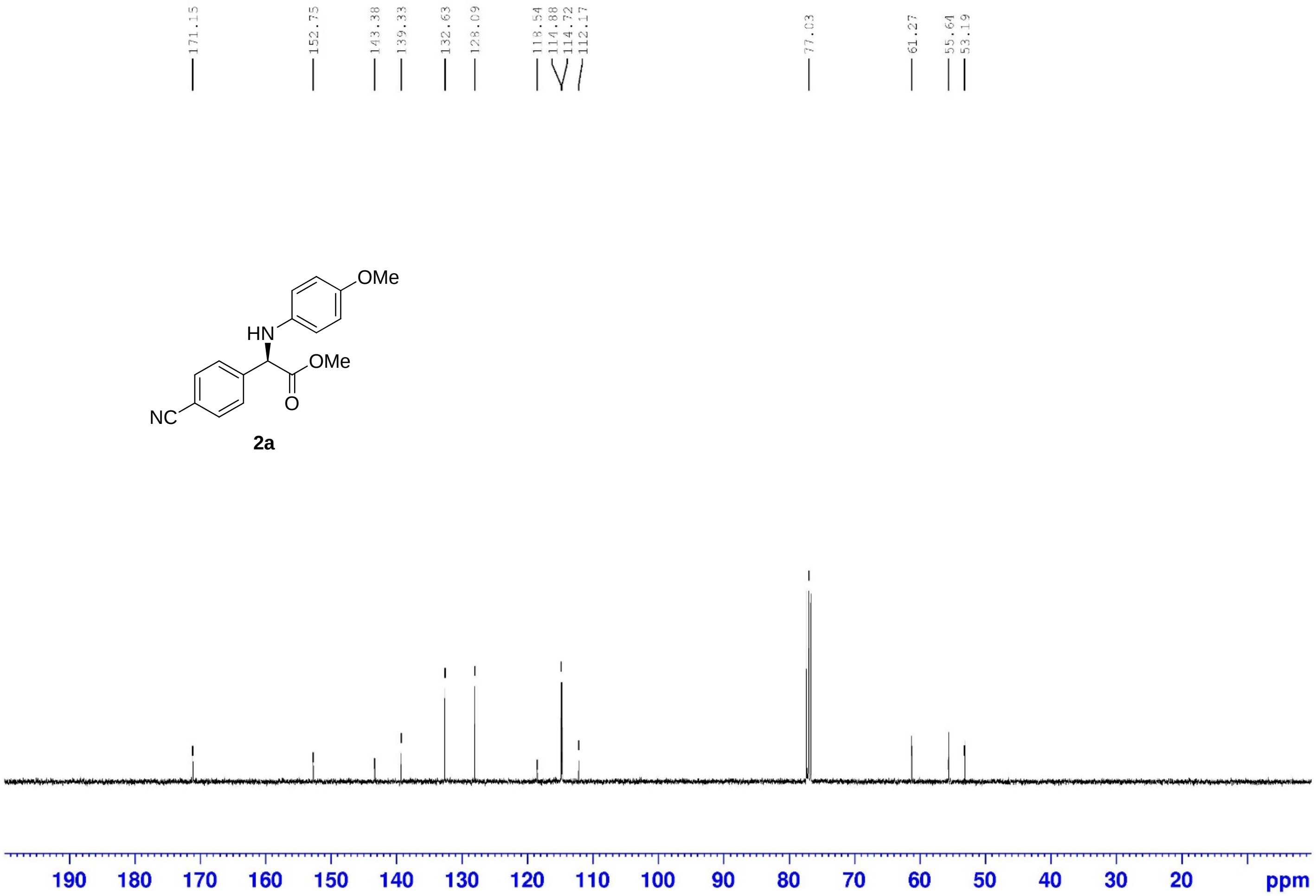

2a

^{1}H NMR $CDCl_3$ (600 MHz)

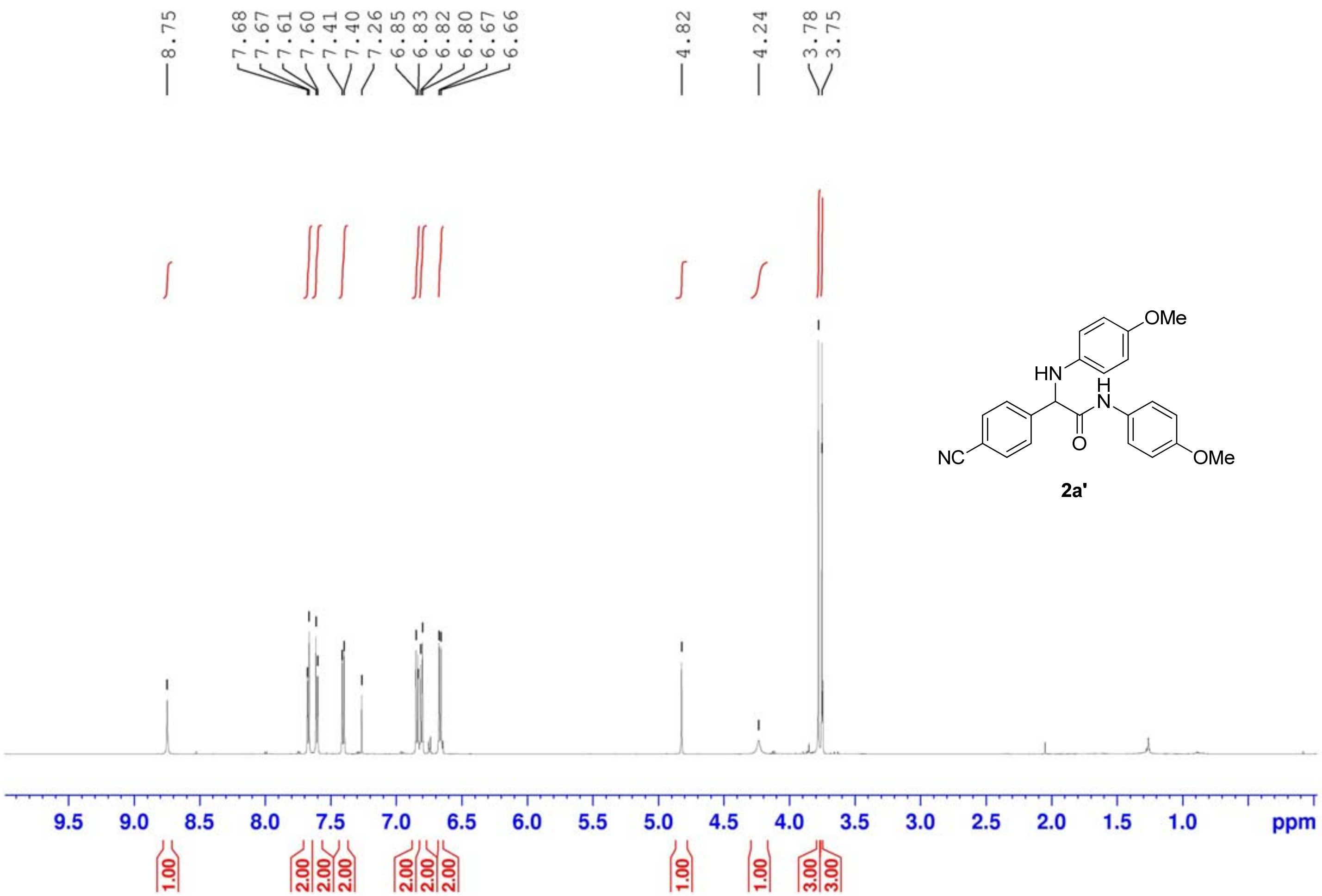

^{13}C NMR $CDCl_3$ (150 MHz)

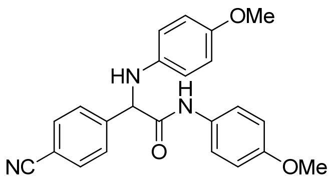

2a'

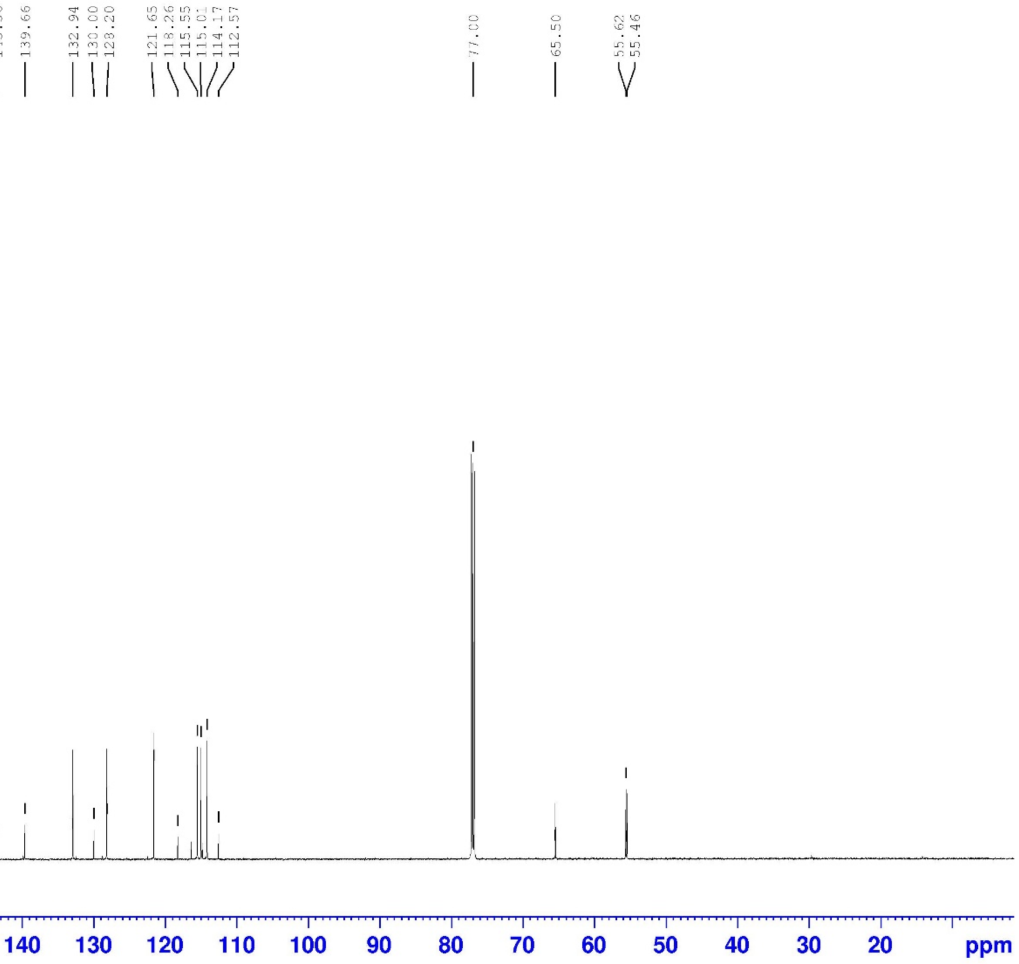

^{1}H NMR $CDCl_3$ (600 MHz)

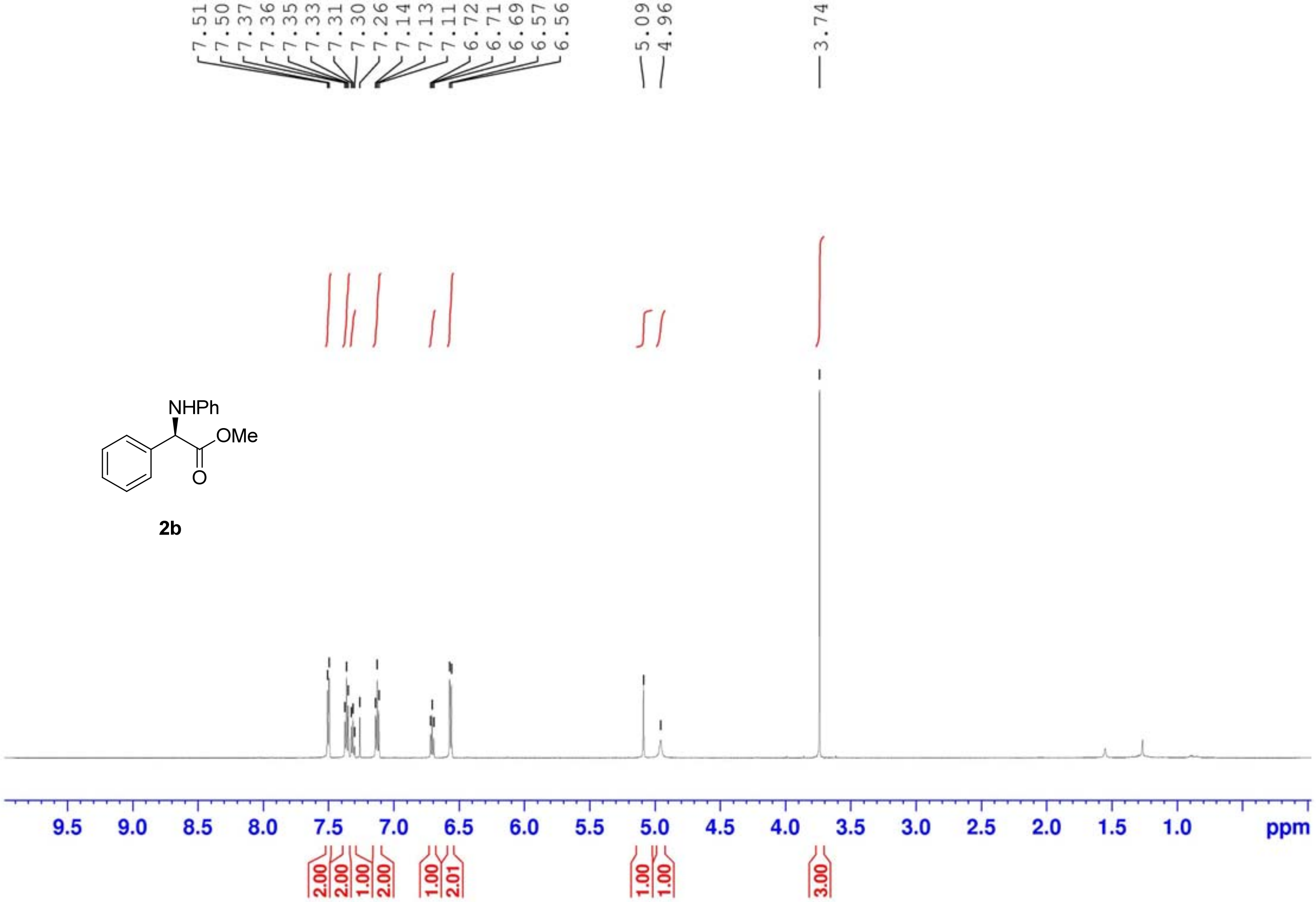

^{13}C NMR $CDCl_3$ (150 MHz)

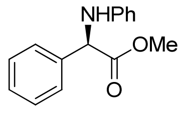

2b

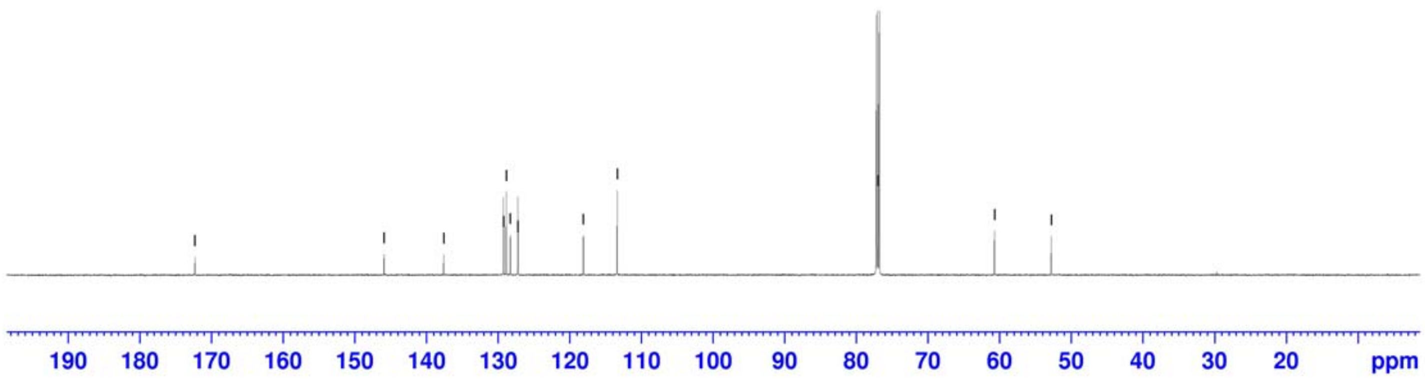

^{1}H NMR $CDCl_3$ (400 MHz)

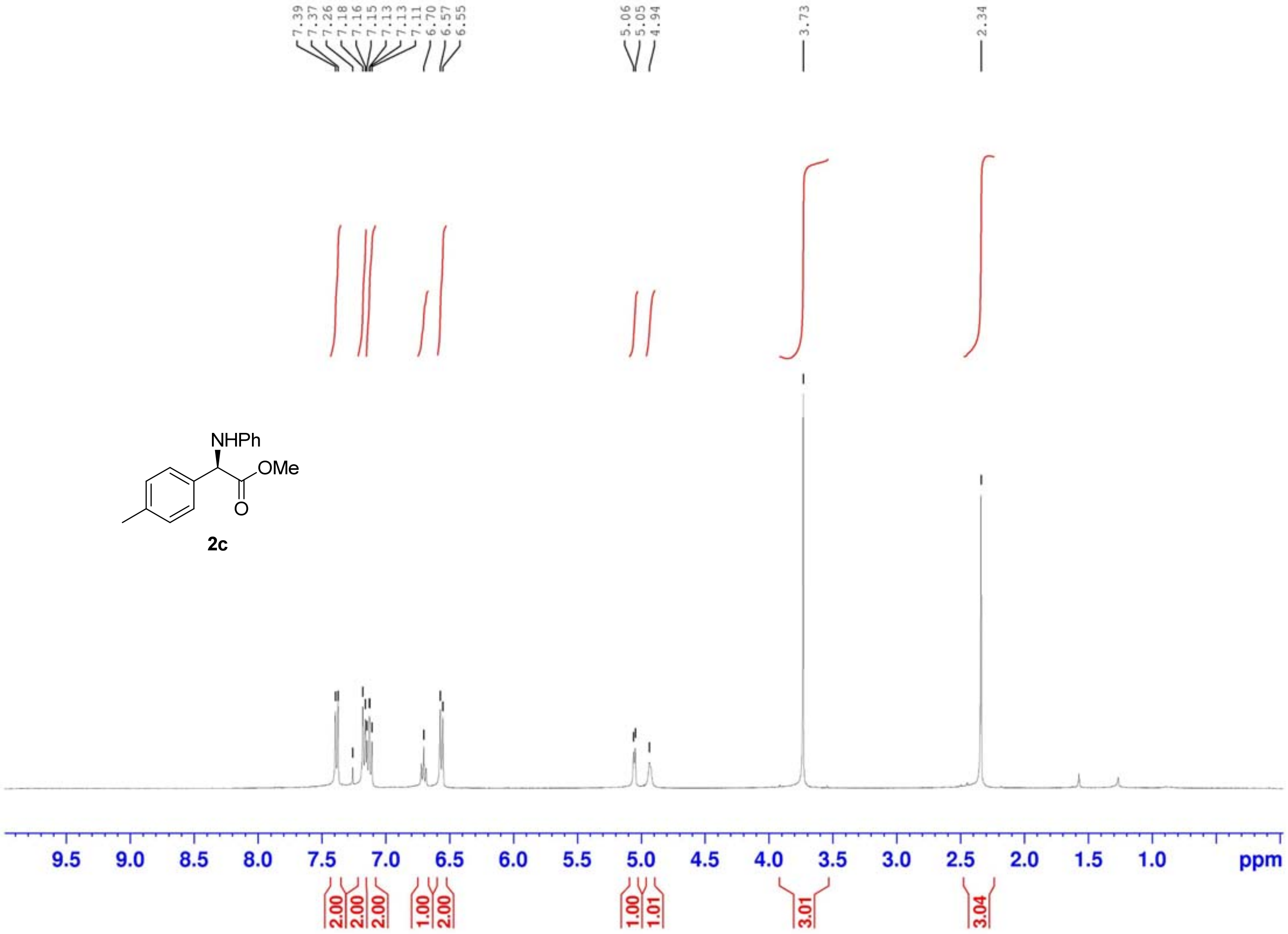

^{13}C NMR $CDCl_3$ (150 MHz)

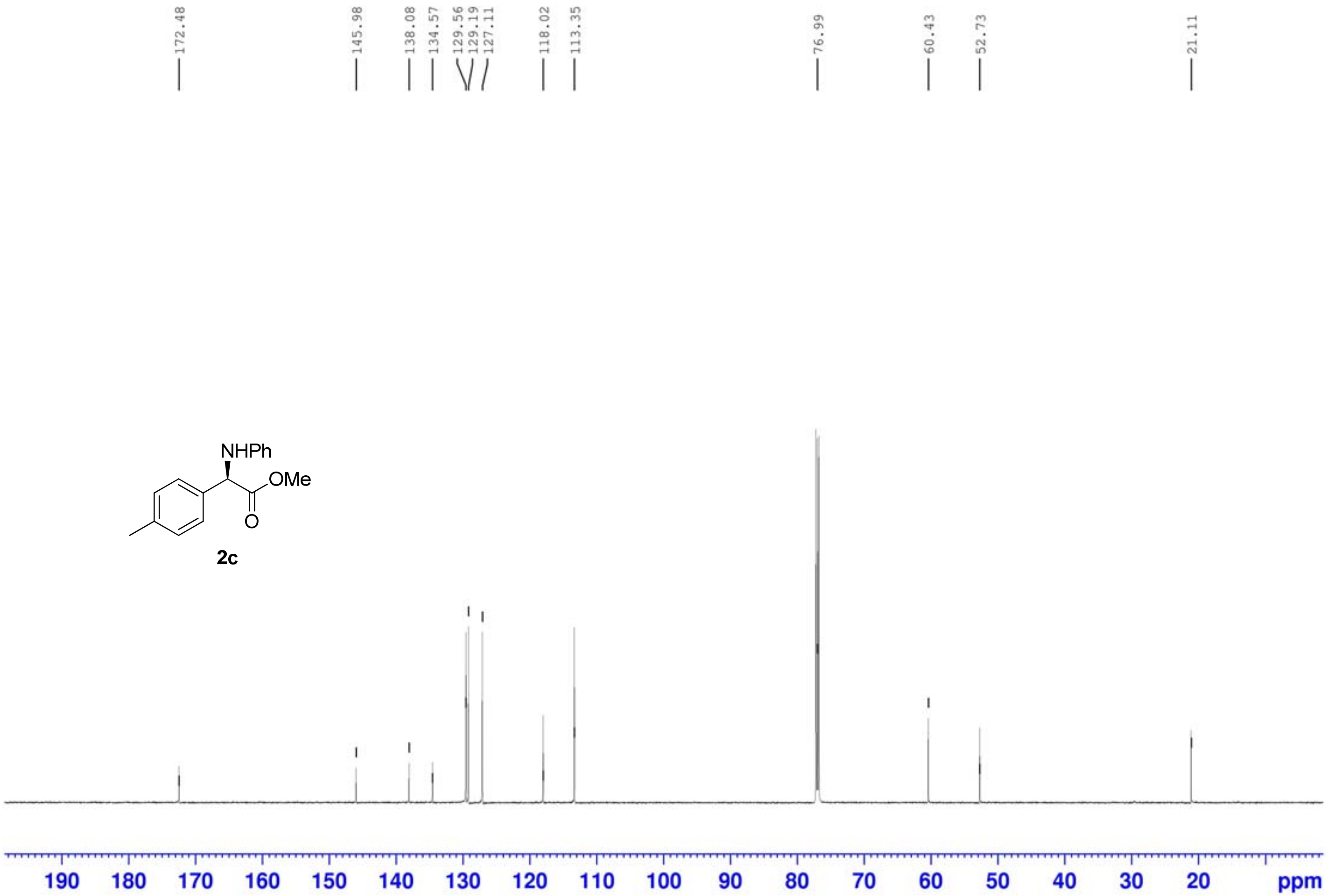

^1H NMR $CDCl_3$ (400 MHz)

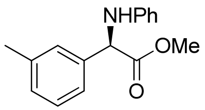

2d

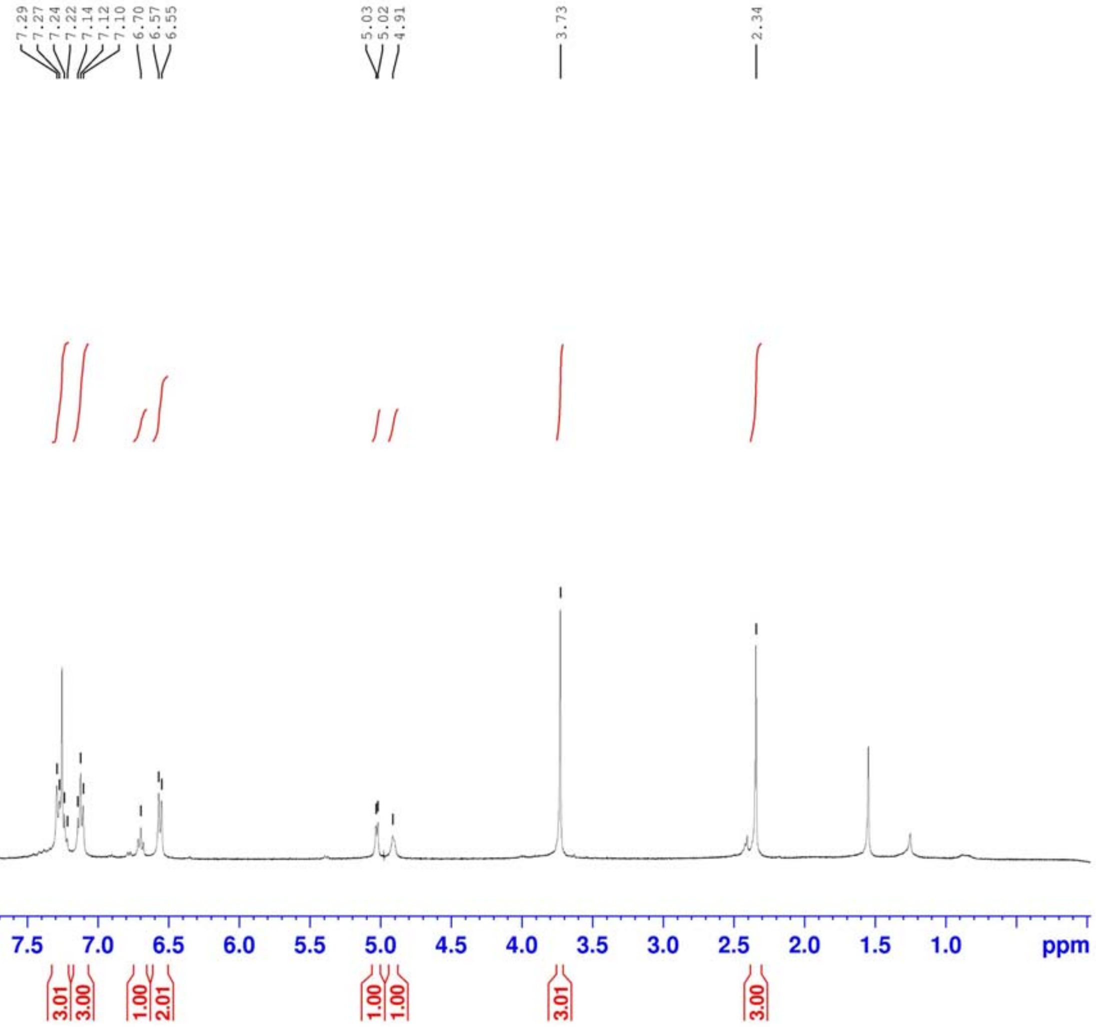

^{13}C NMR $CDCl_3$ (100 MHz)

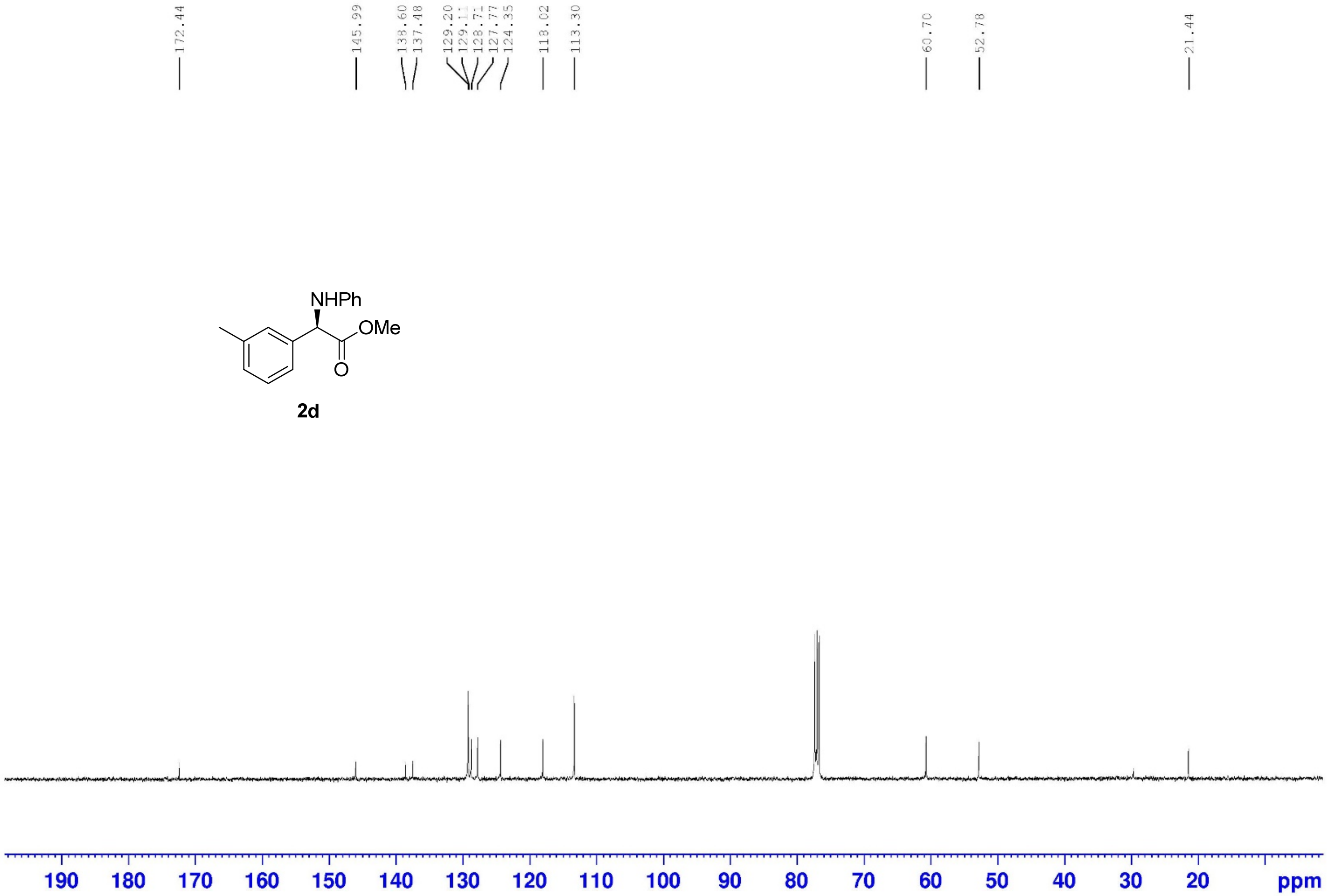

2d

^1H NMR $CDCl_3$ (600 MHz)

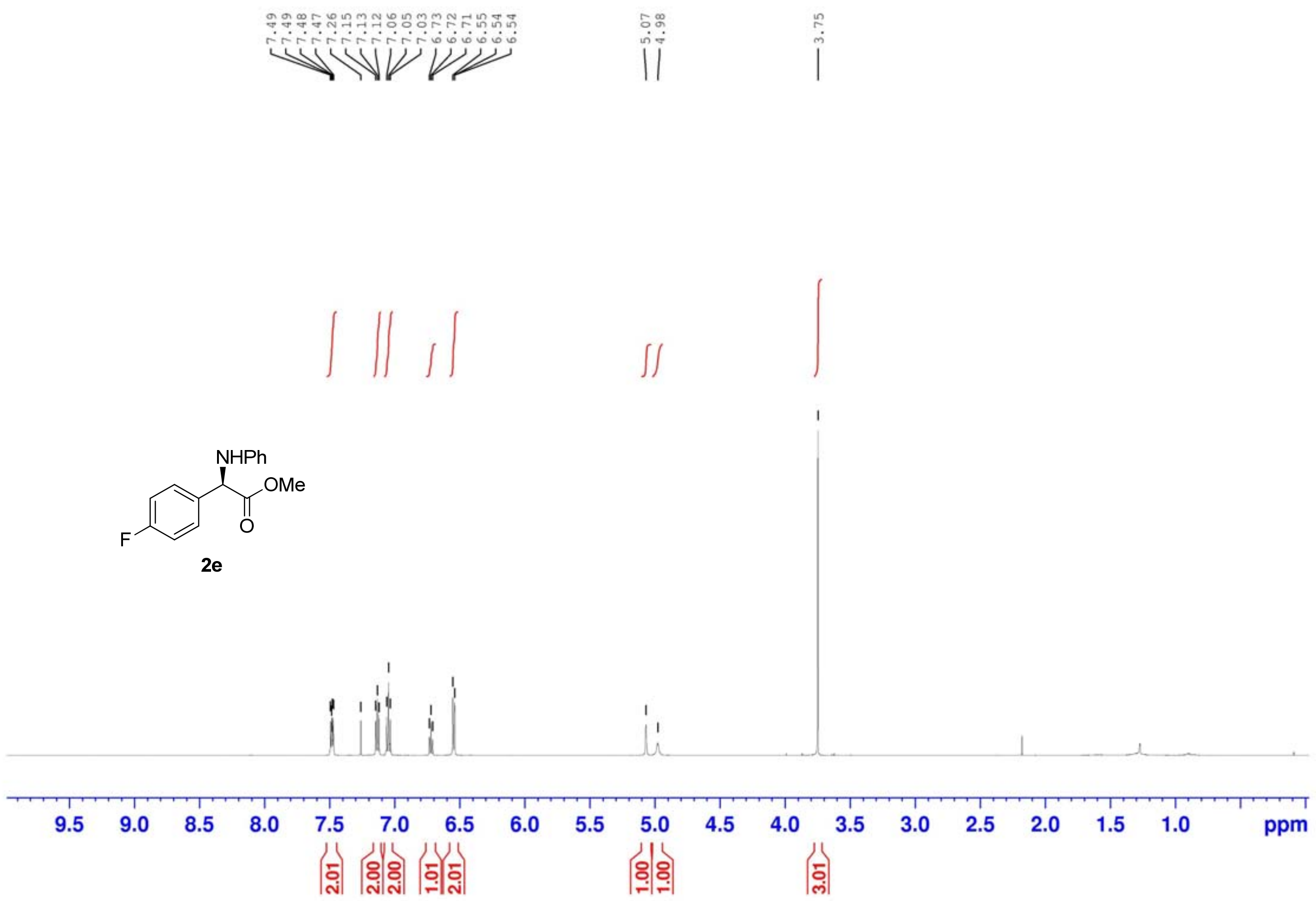

^{13}C NMR $CDCl_3$ (150 MHz)

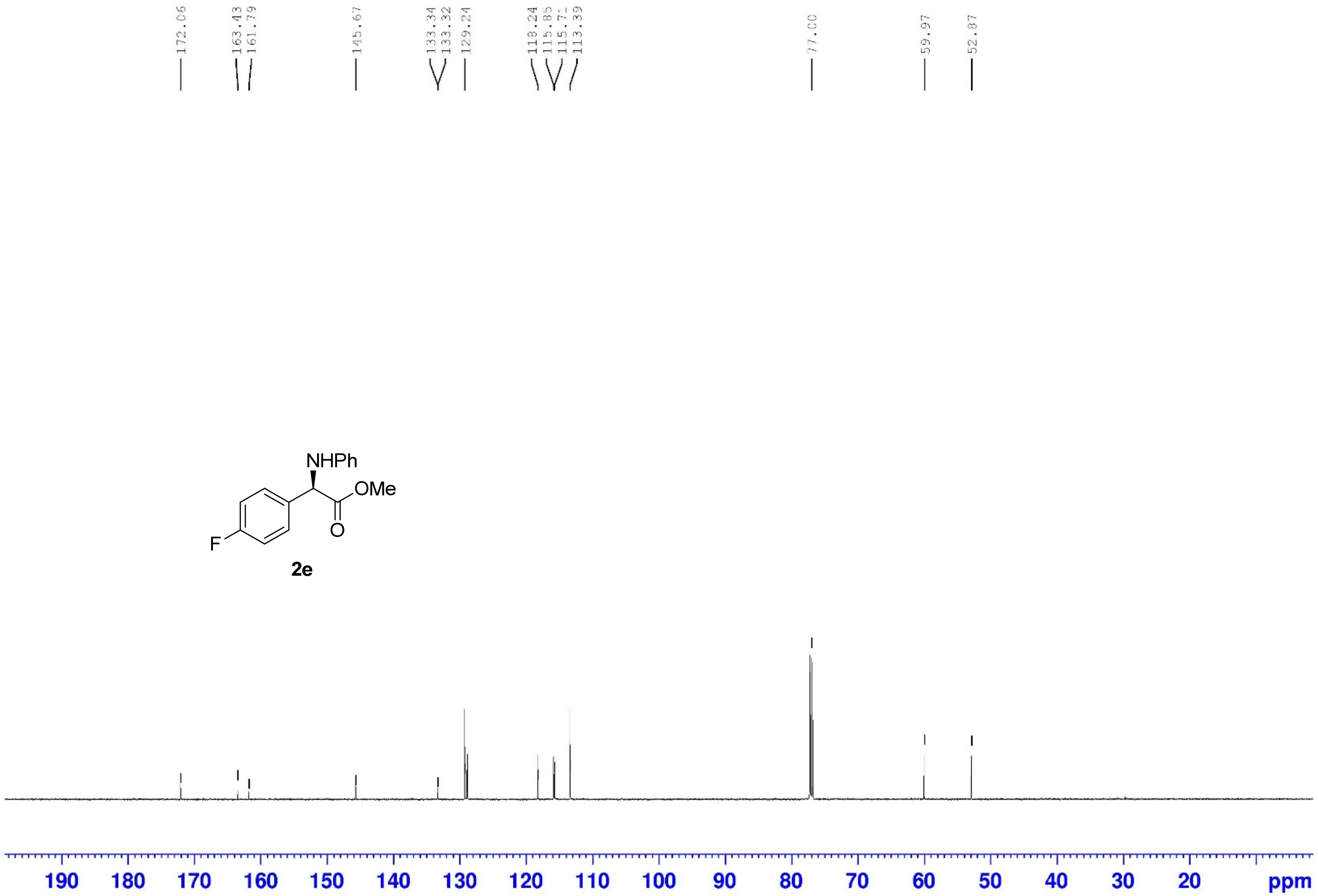

^{1}H NMR $CDCl_3$ (300 MHz)

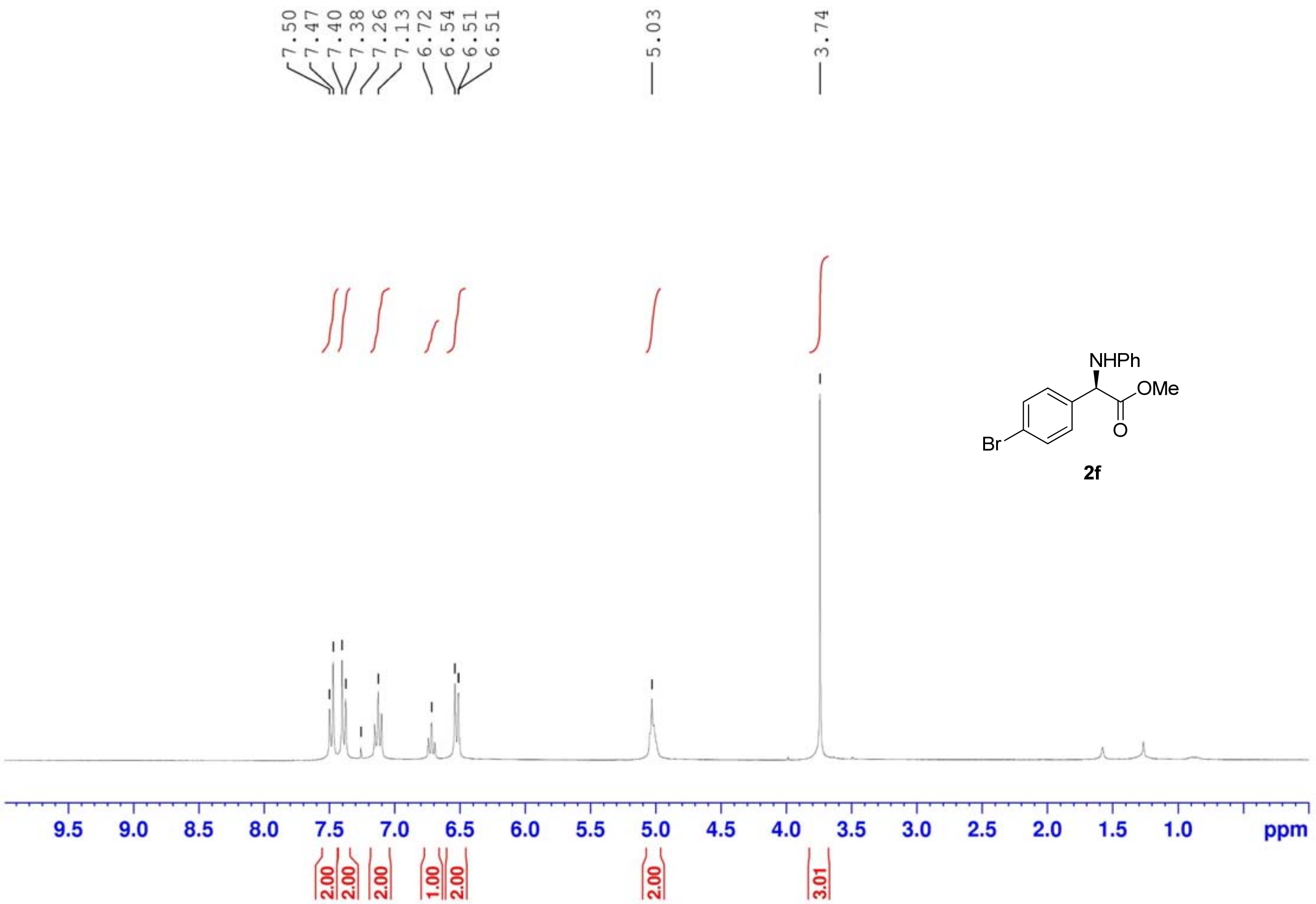

^{13}C NMR $CDCl_3$ (75 MHz)

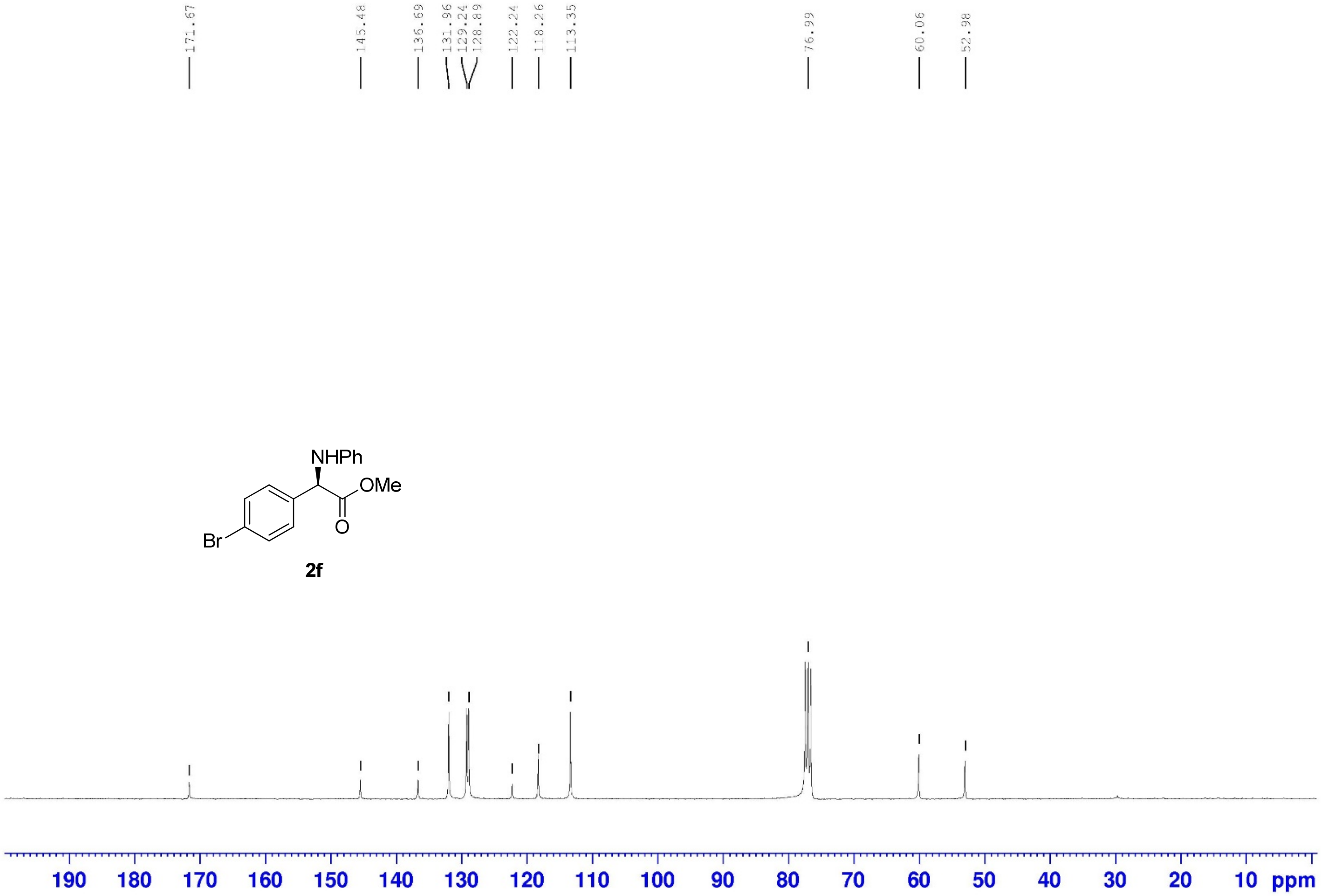

^{1}H NMR $CDCl_3$ (600 MHz)

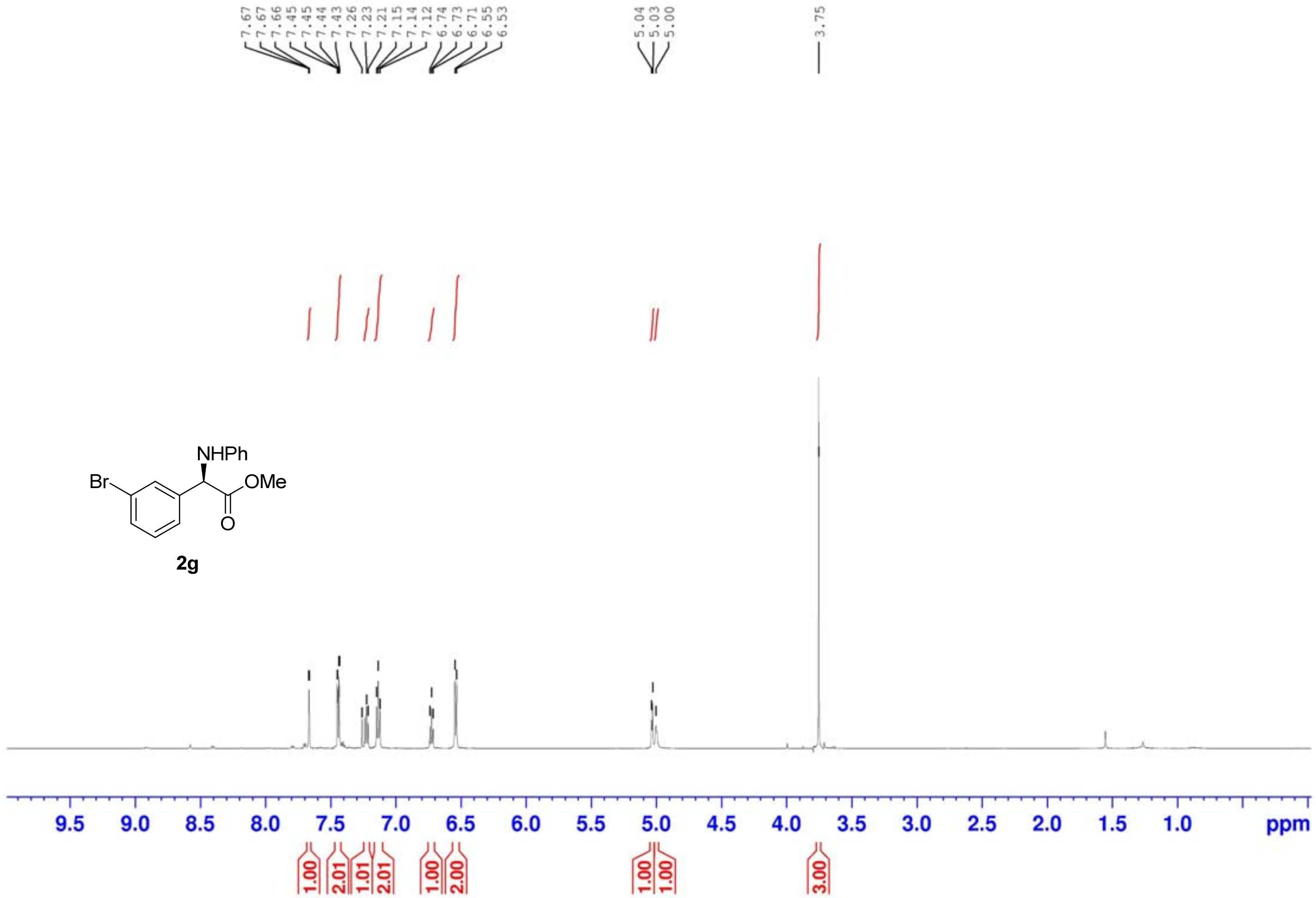

^{13}C NMR $CDCl_3$ (150 MHz)

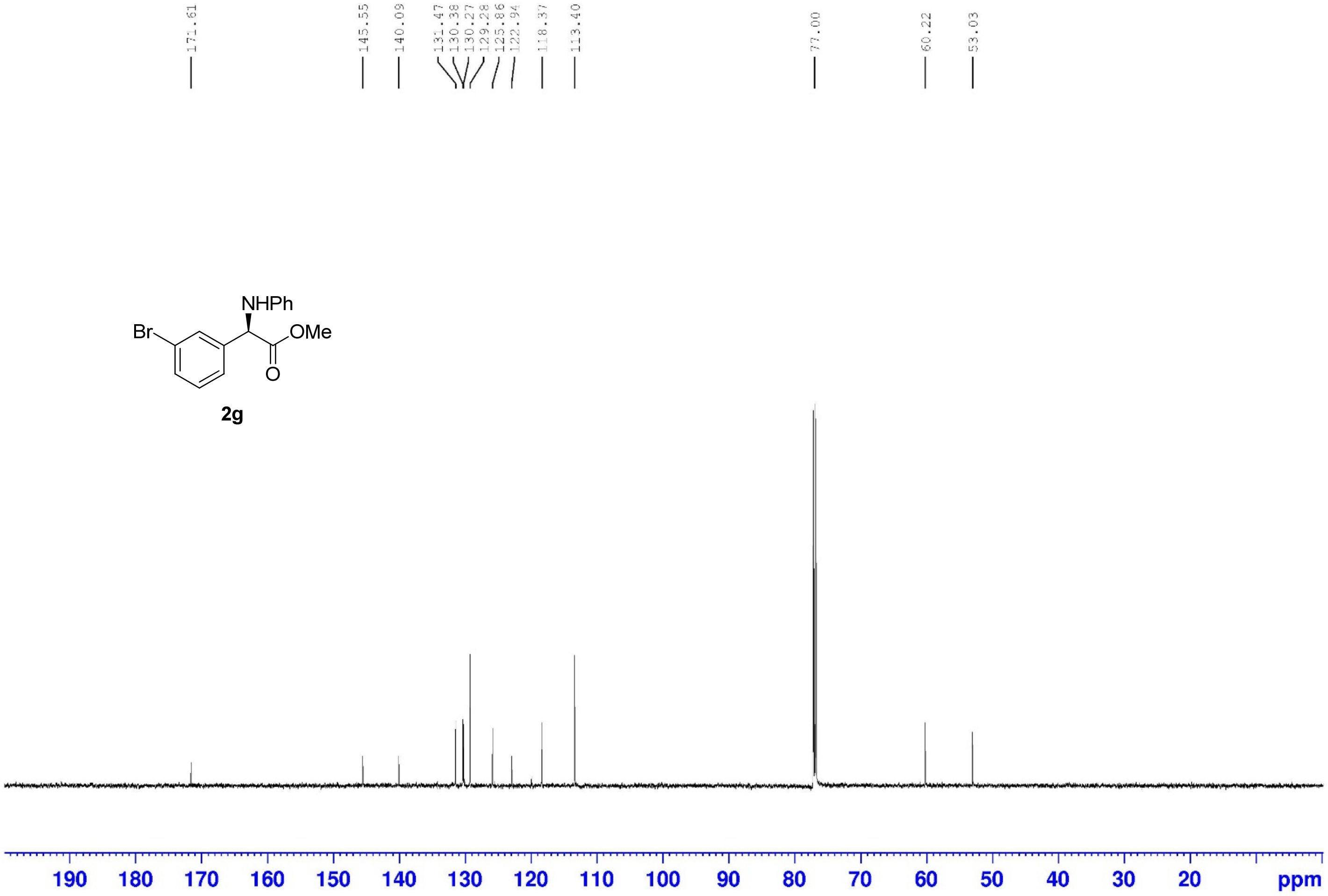

^{1}H NMR $CDCl_3$ (400 MHz)

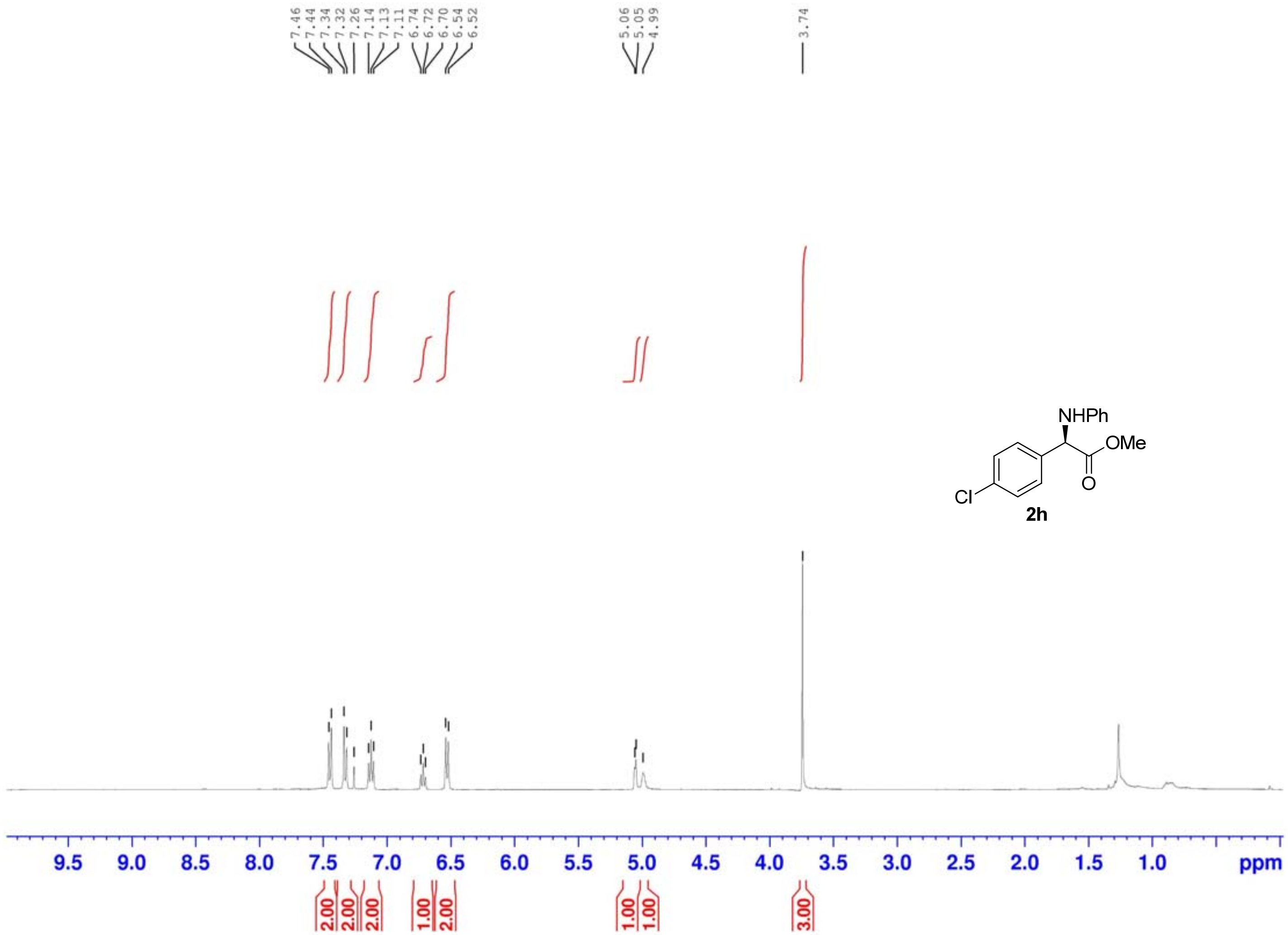

^{13}C NMR $CDCl_3$ (100 MHz)

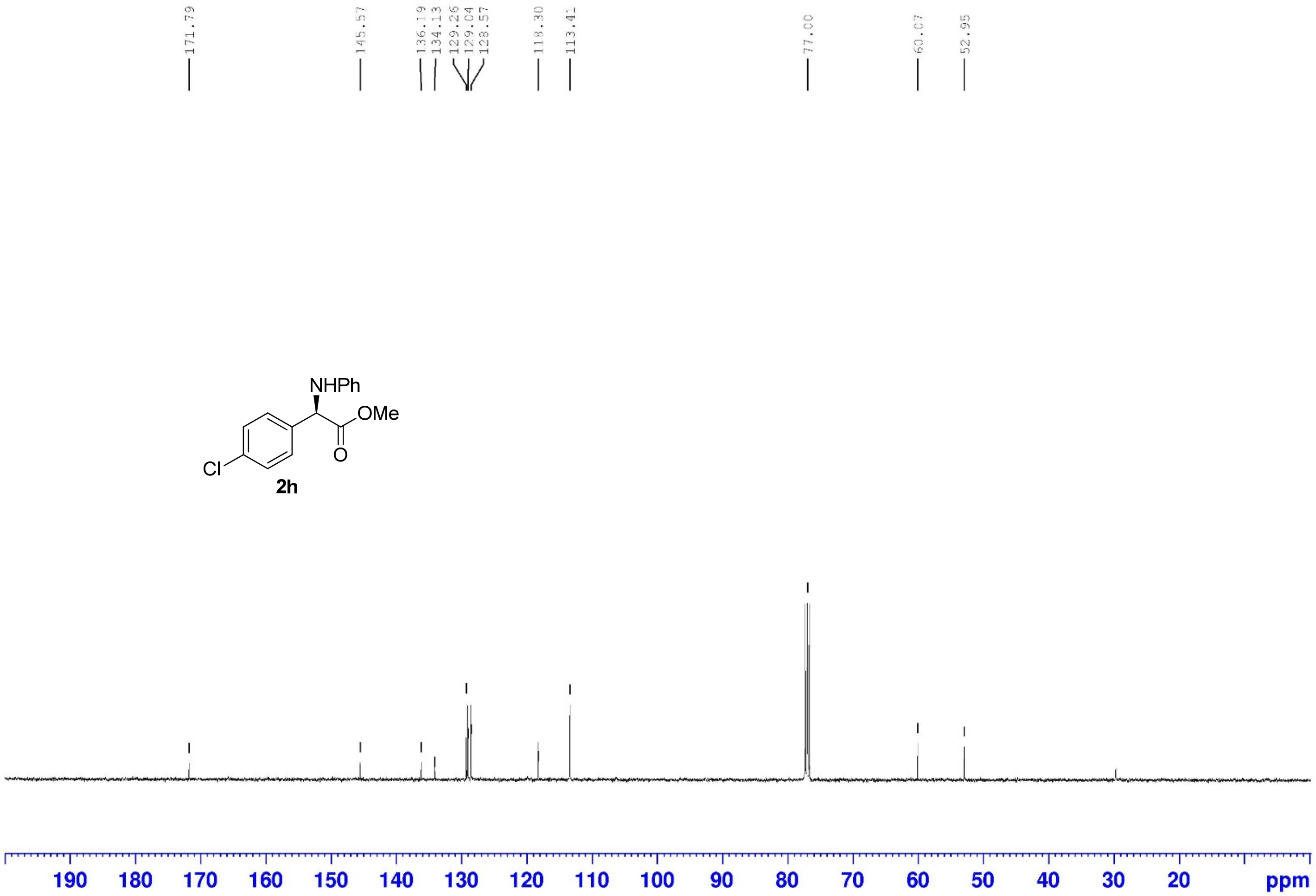

^{1}H NMR $CDCl_3$ (400 MHz)

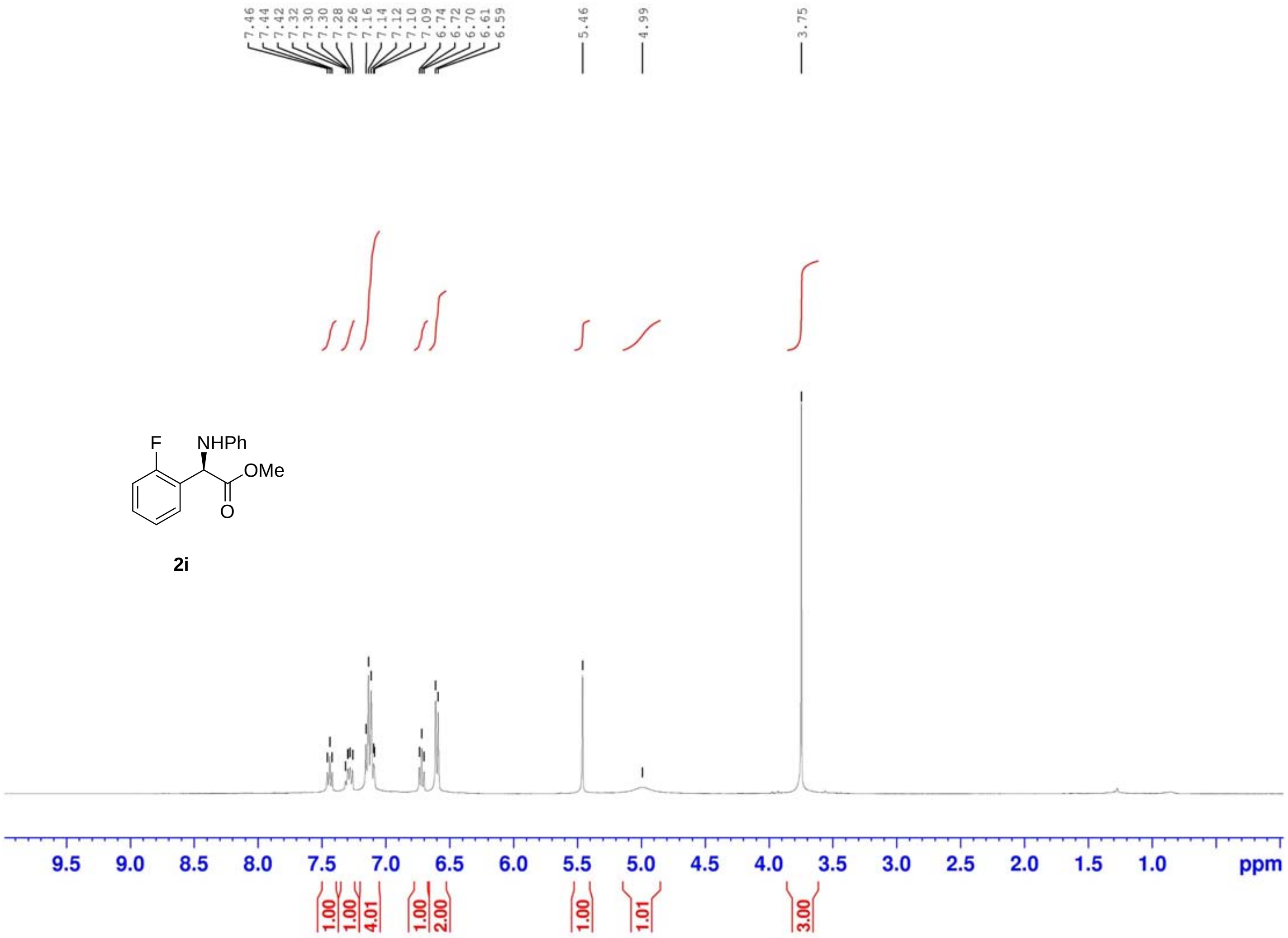

^{13}C NMR $CDCl_3$ (100 MHz)

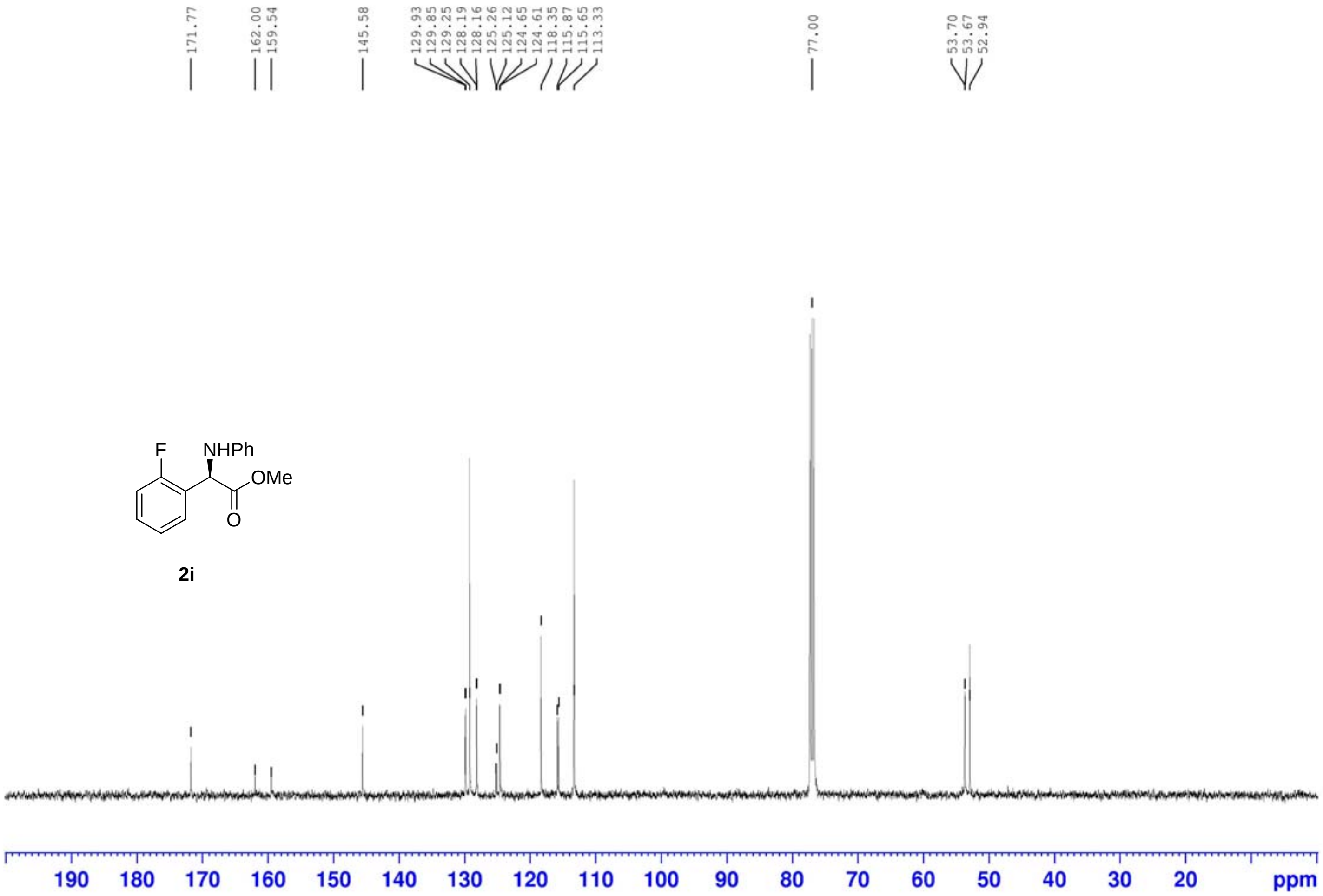

2i

^{1}H NMR $CDCl_3$ (600 MHz)

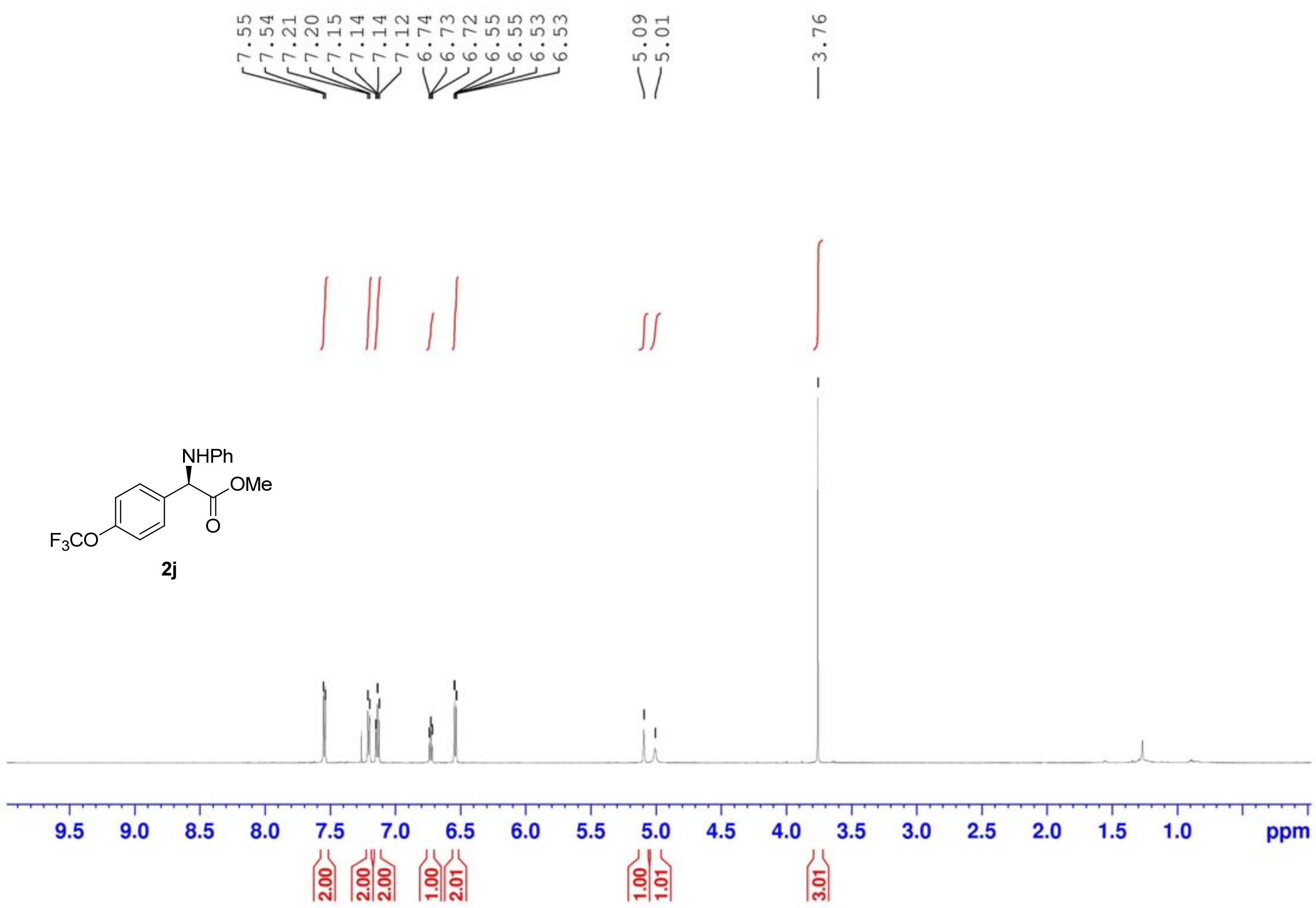

^{13}C NMR $CDCl_3$ (75 MHz)

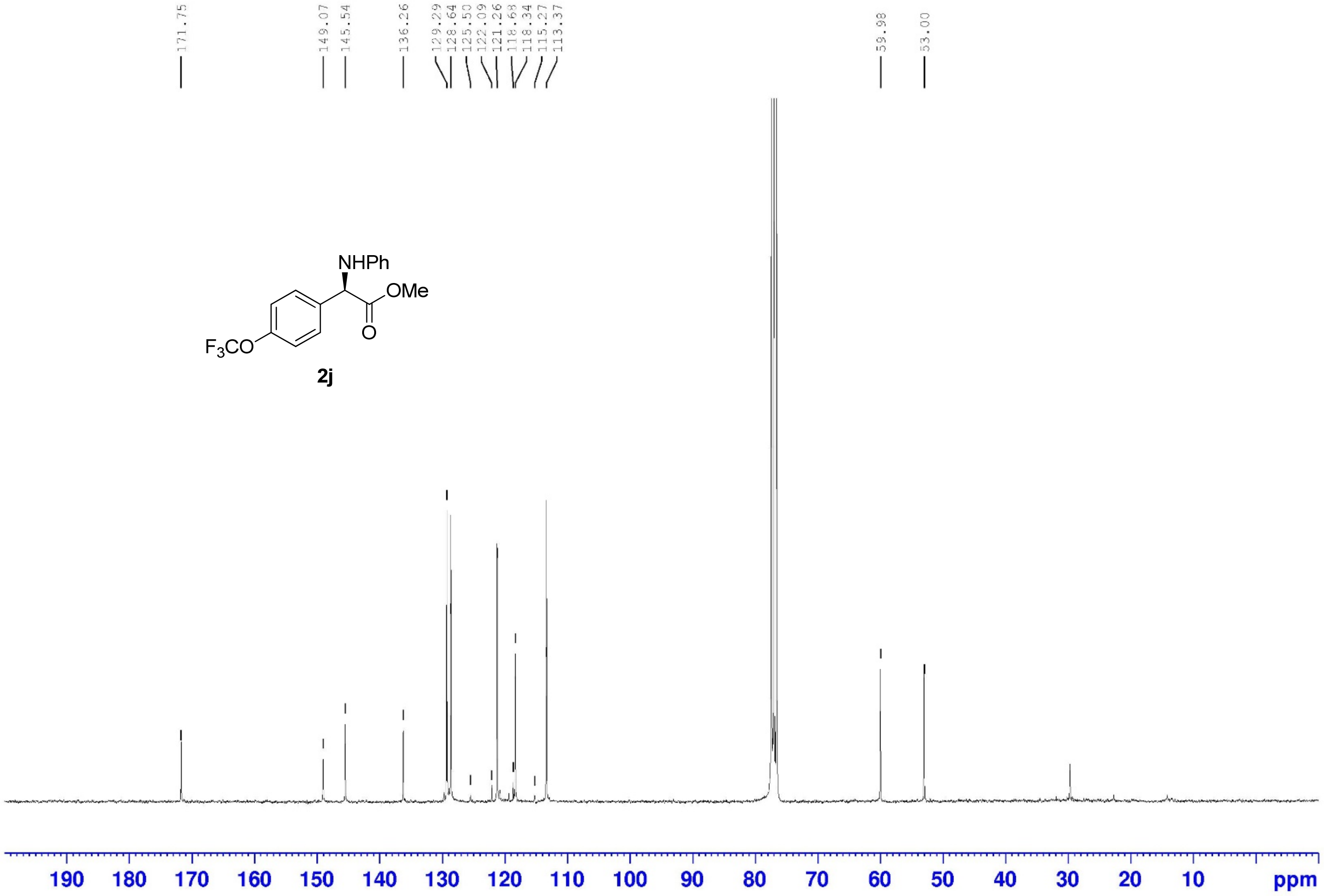

^{1}H NMR $CDCl_3$ (600 MHz)

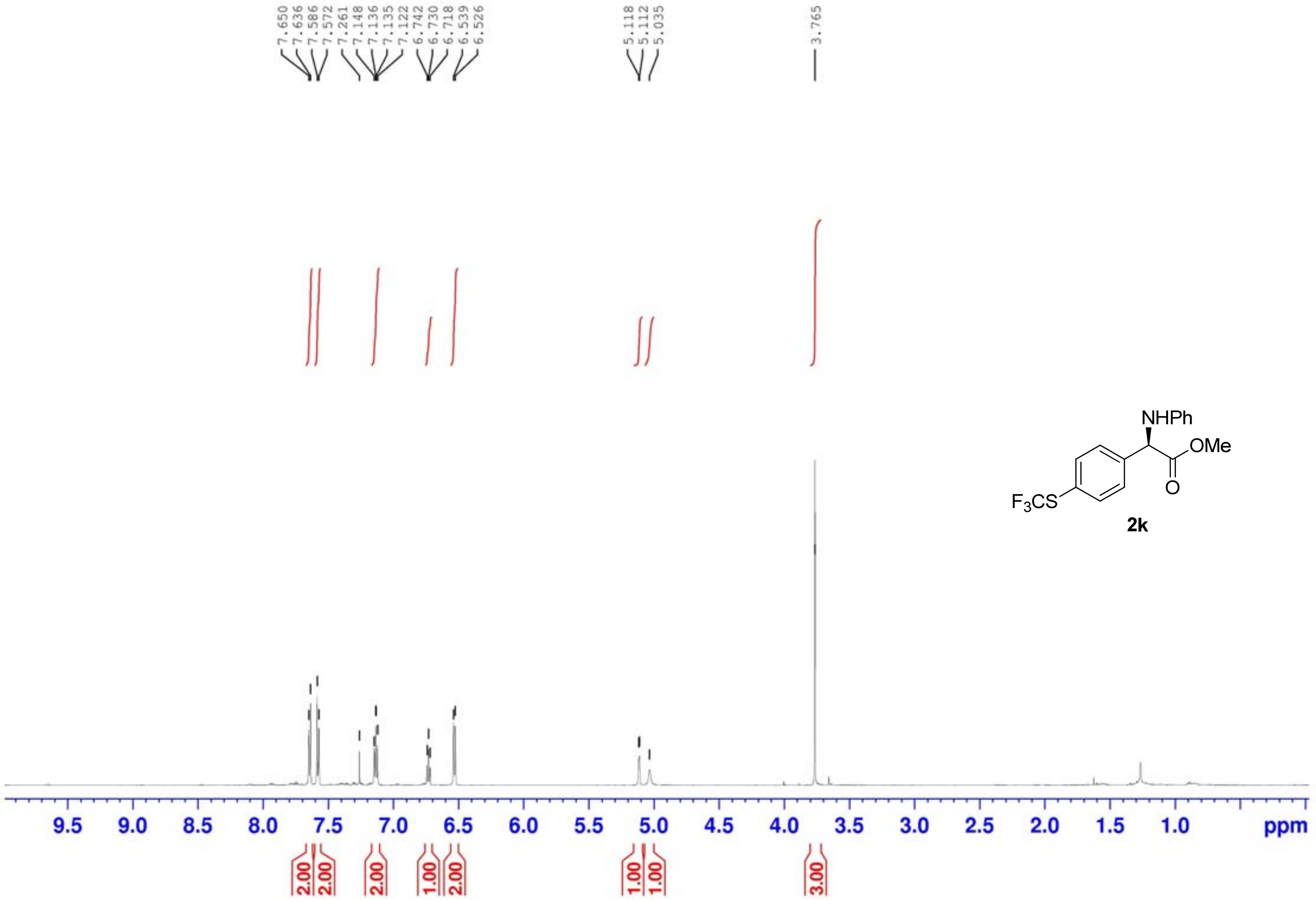

^{13}C NMR $CDCl_3$ (150 MHz)

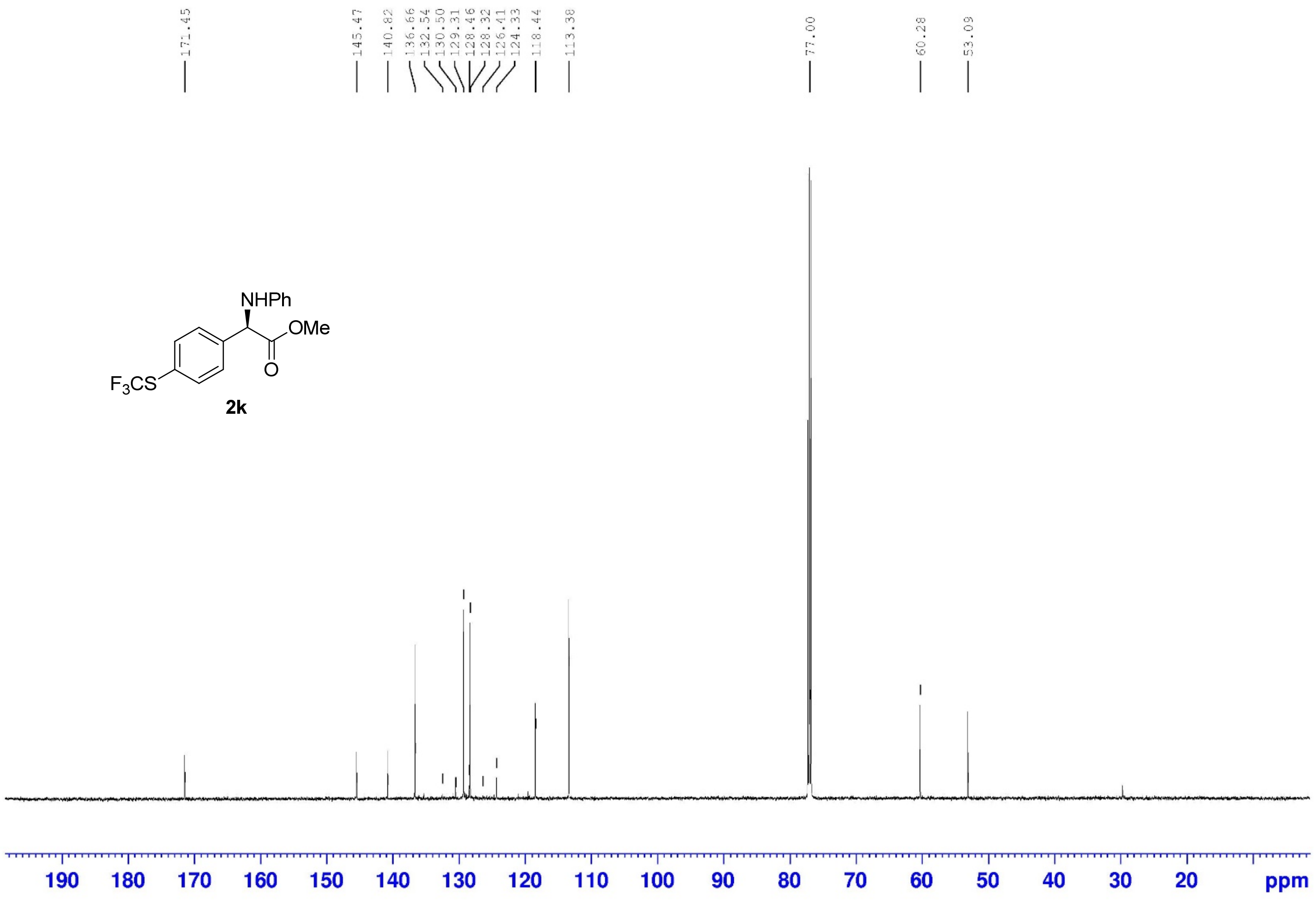

^{1}H NMR $CDCl_3$ (400 MHz)

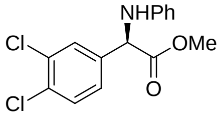

2l

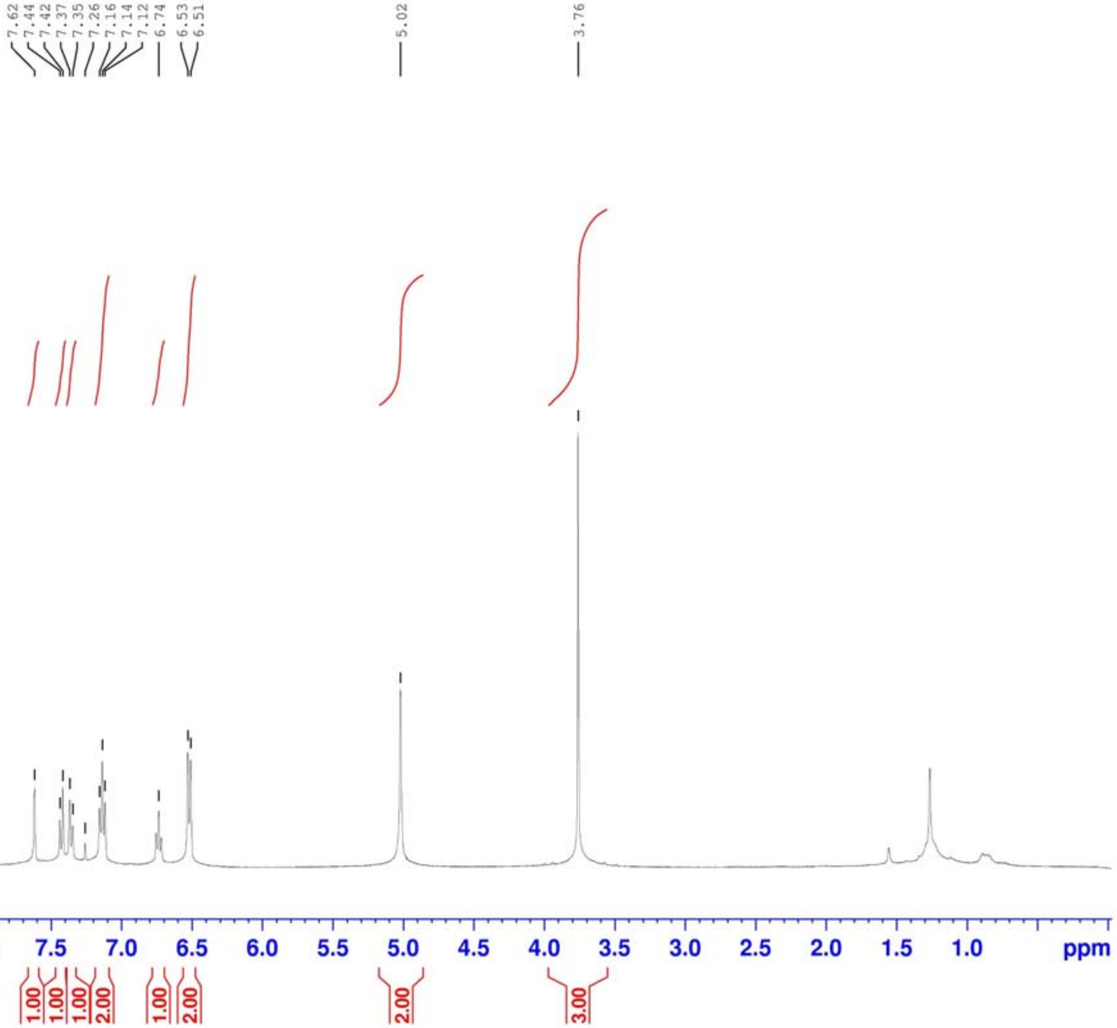

^{13}C NMR $CDCl_3$ (75 MHz)

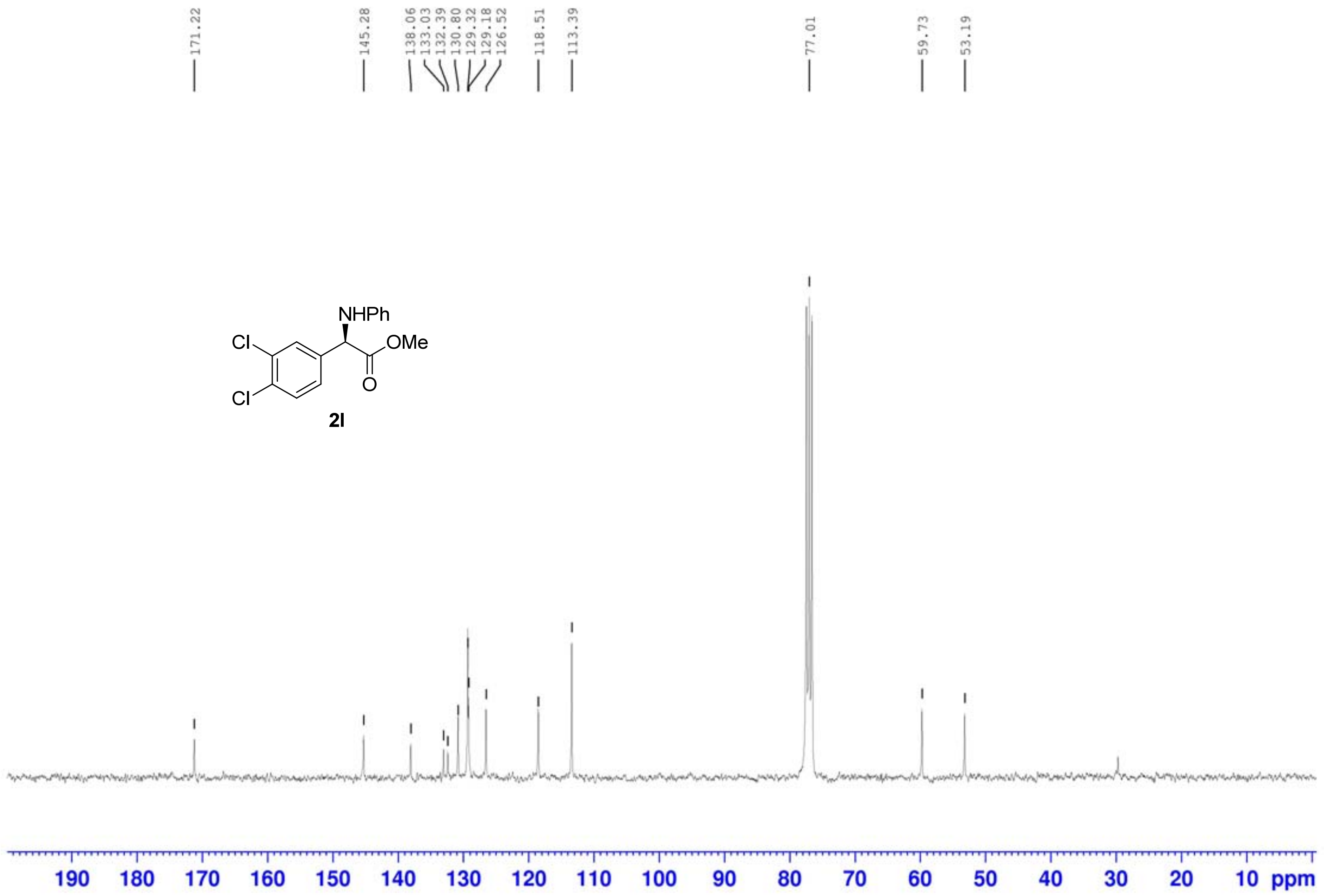

^{1}H NMR $CDCl_3$ (600 MHz)

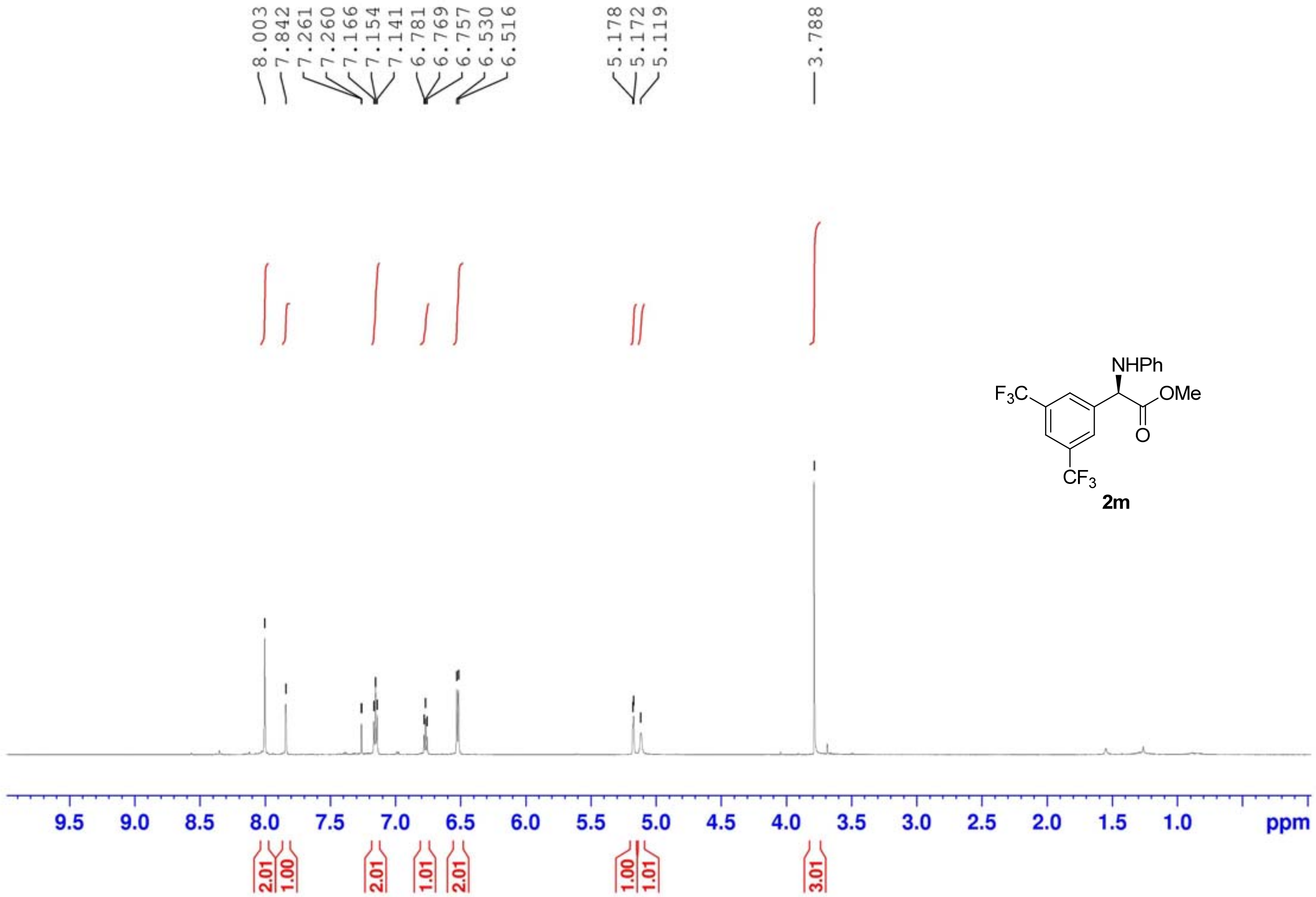

^{13}C NMR $CDCl_3$ (75 MHz)

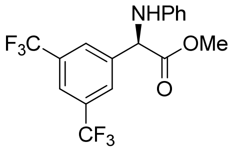

2m

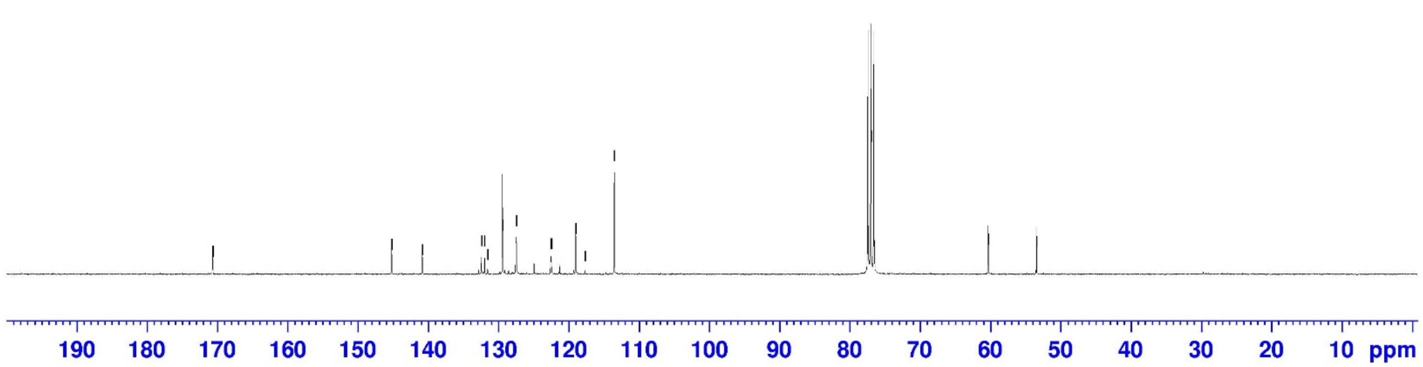

^{1}H NMR $CDCl_3$ (400 MHz)

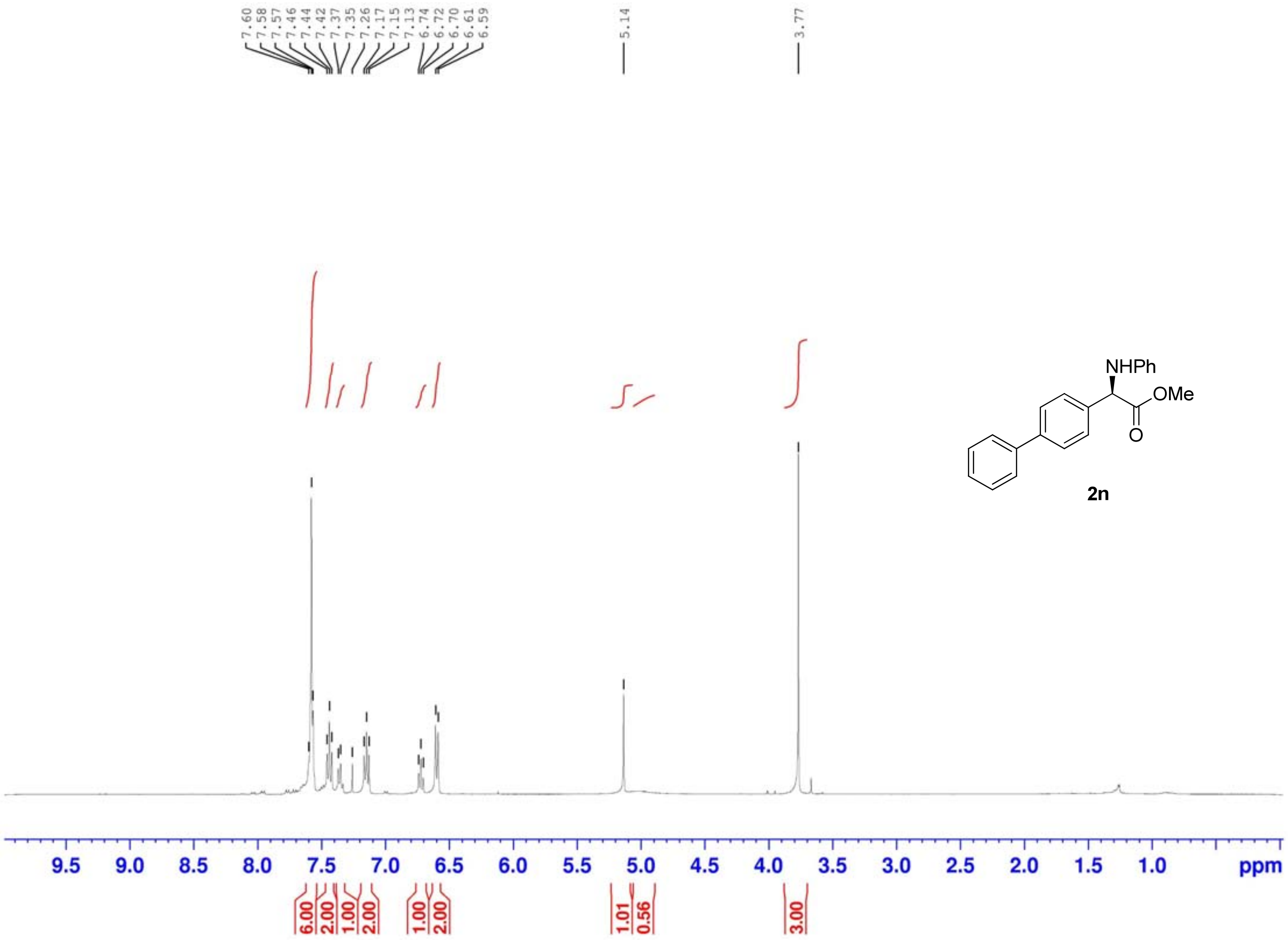

2n

^{13}C NMR $CDCl_3$ (100 MHz)

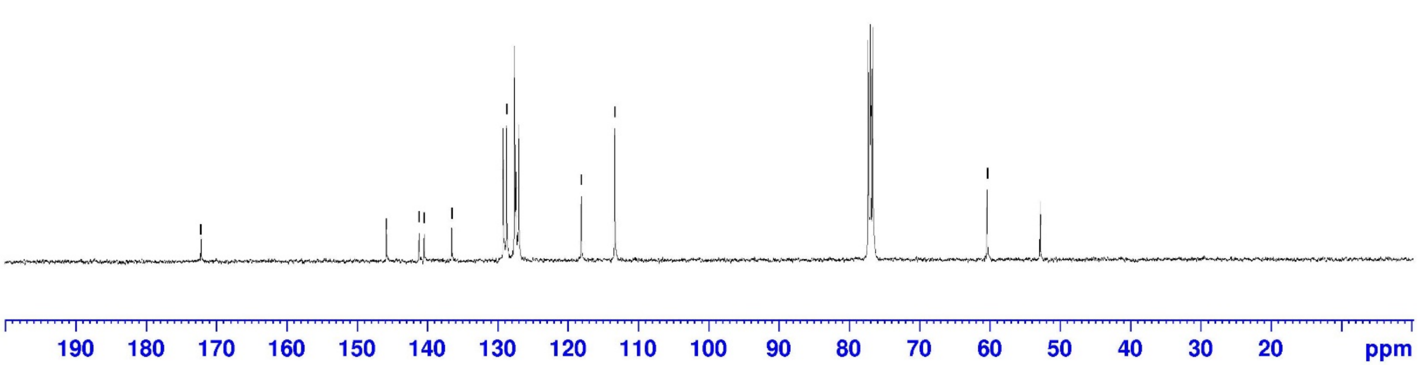

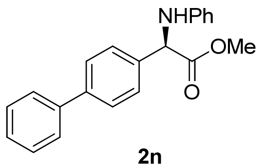

2n

^{1}H NMR $CDCl_3$ (600 MHz)

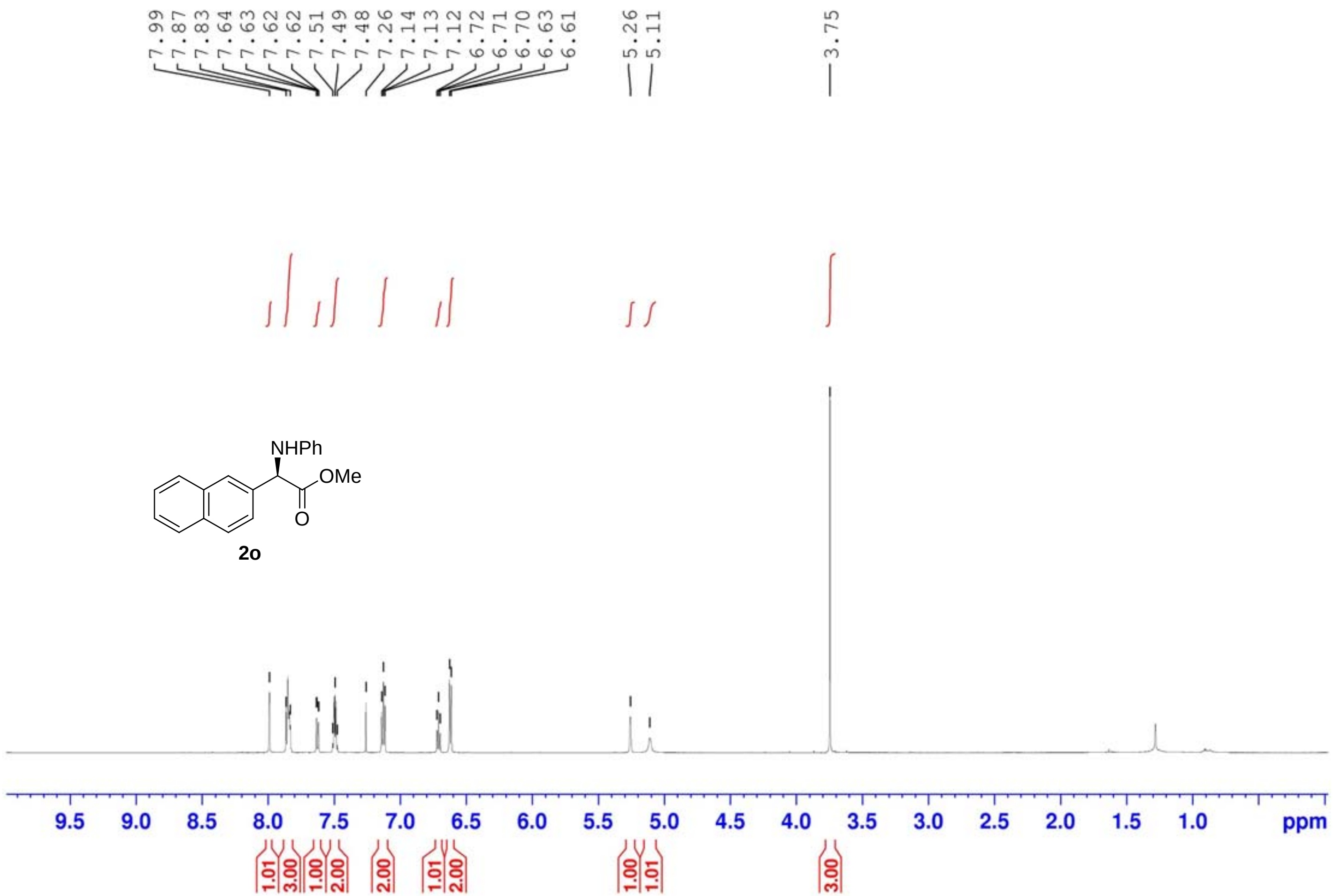

^{13}C NMR $CDCl_3$ (150 MHz)

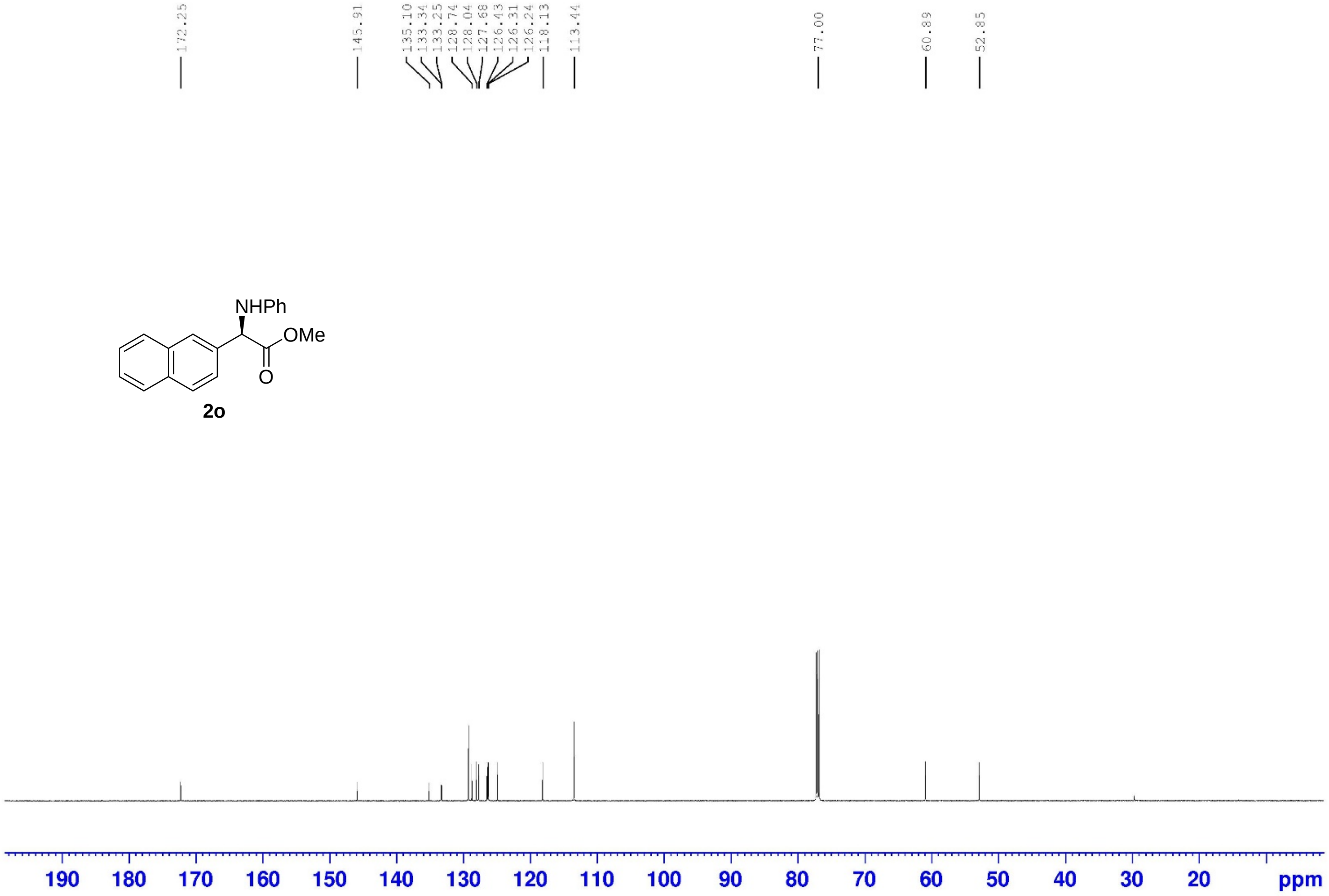

2o

^1H NMR $CDCl_3$ (300 MHz)

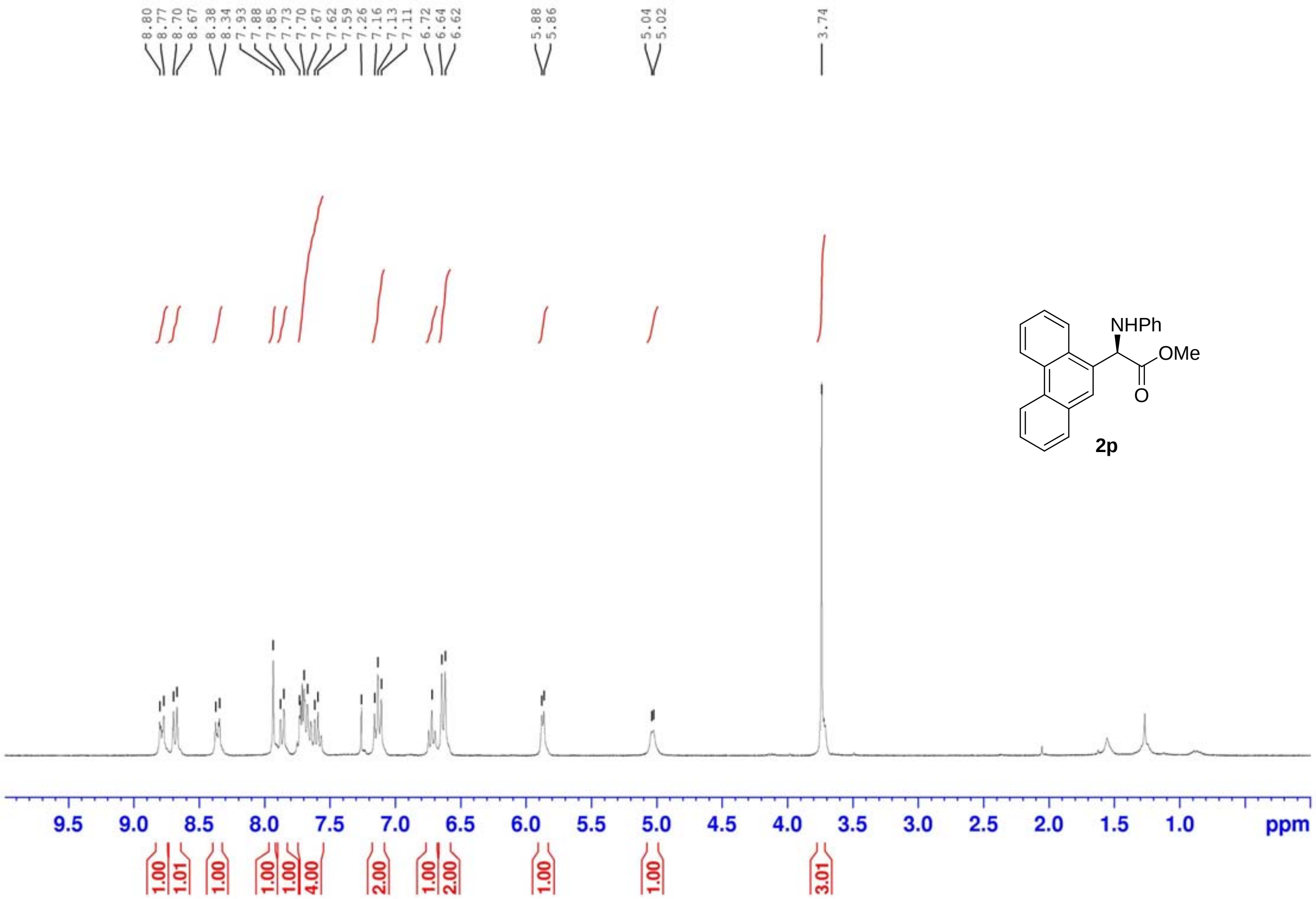

2p

^{13}C NMR $CDCl_3$ (75 MHz)

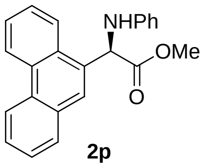

2p

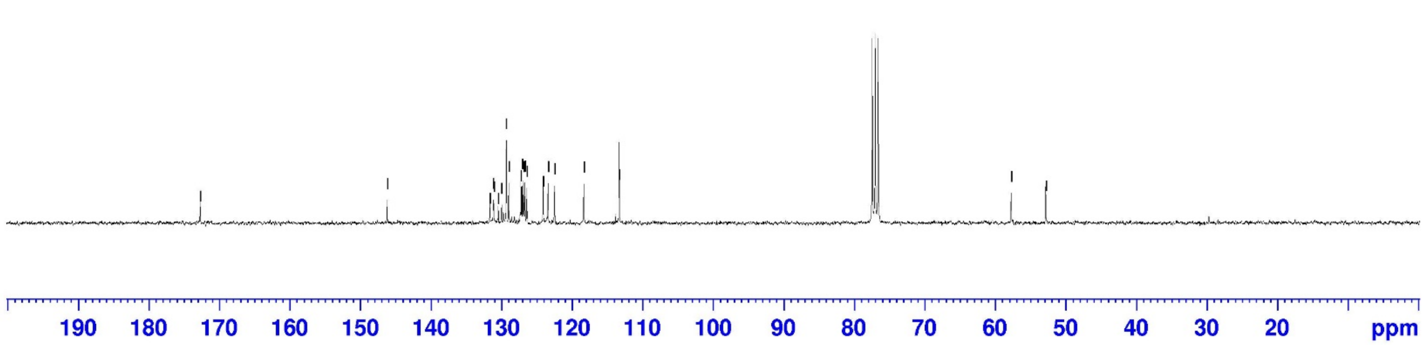

^{1}H NMR $CDCl_3$ (400 MHz)

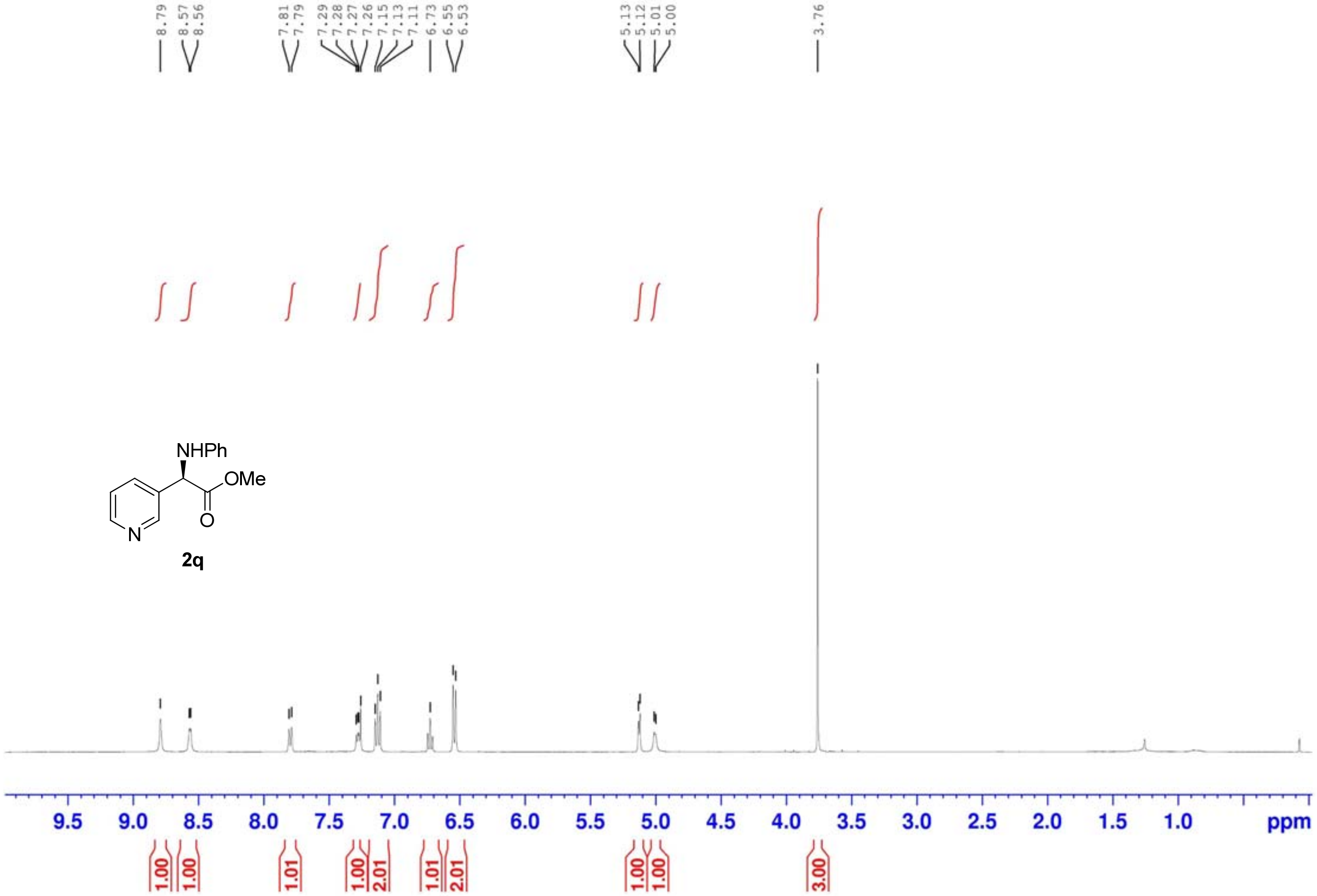

^{13}C NMR $CDCl_3$ (100 MHz)

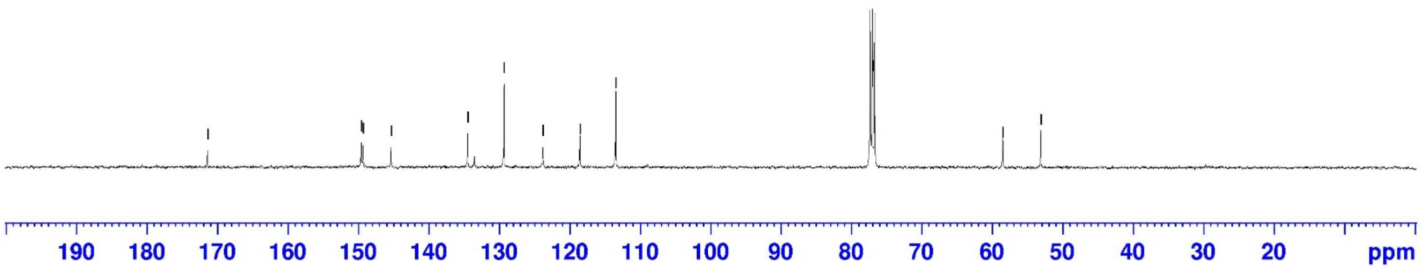

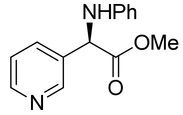

2q

^1H NMR $CDCl_3$ (400 MHz)

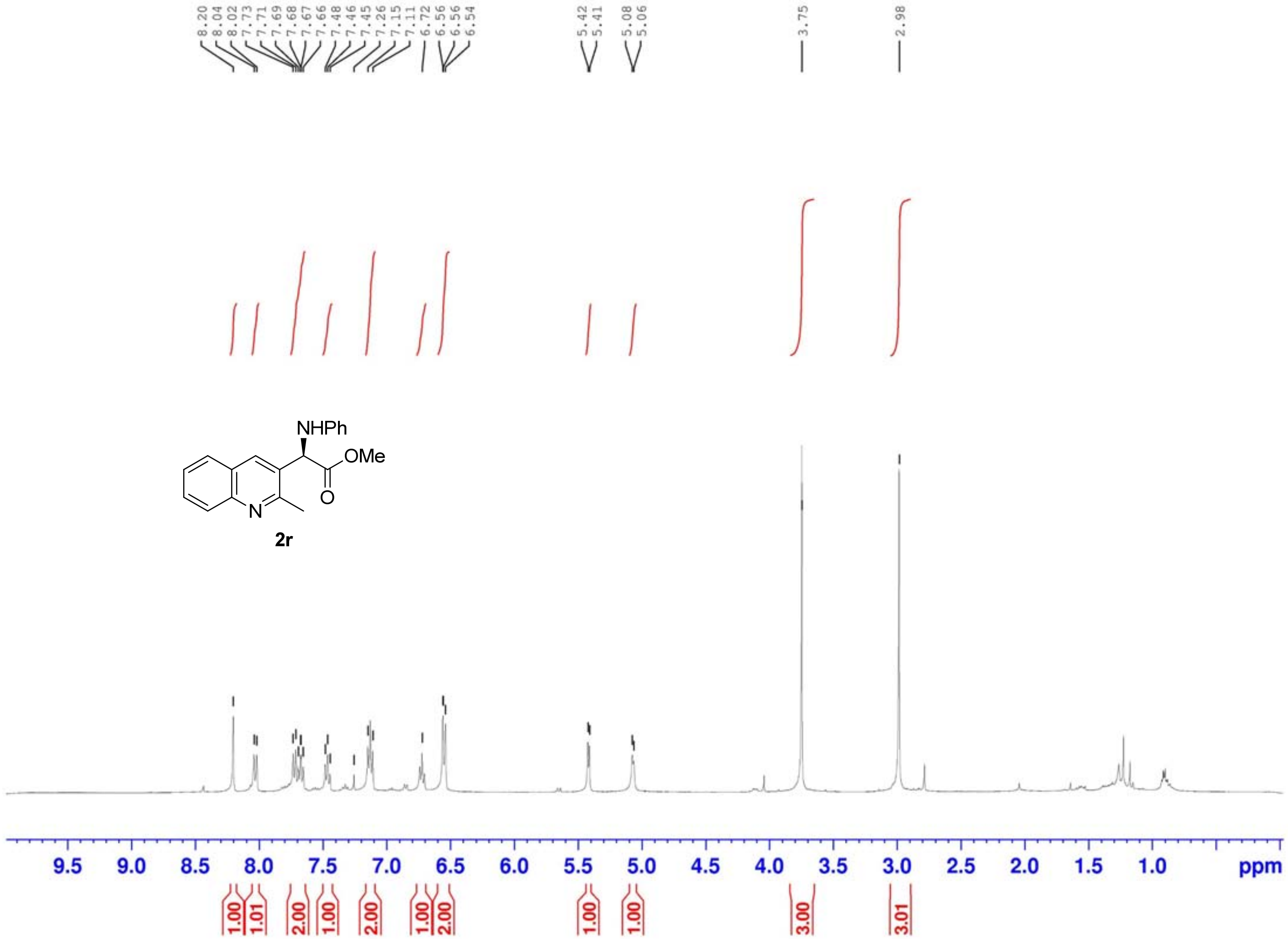

^{13}C NMR $CDCl_3$ (100 MHz)

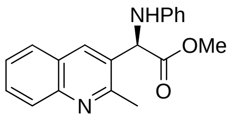

2r

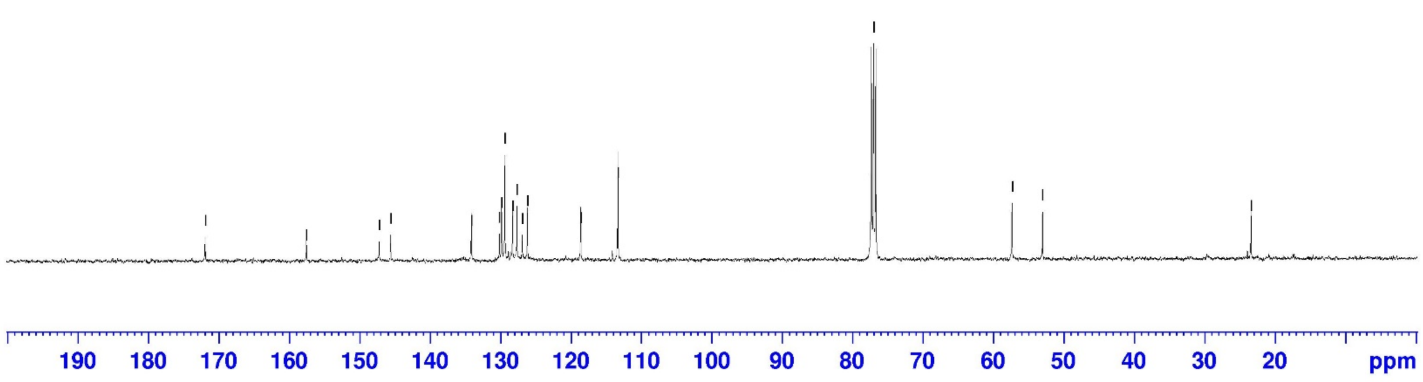

^1H NMR $CDCl_3$ (400 MHz)

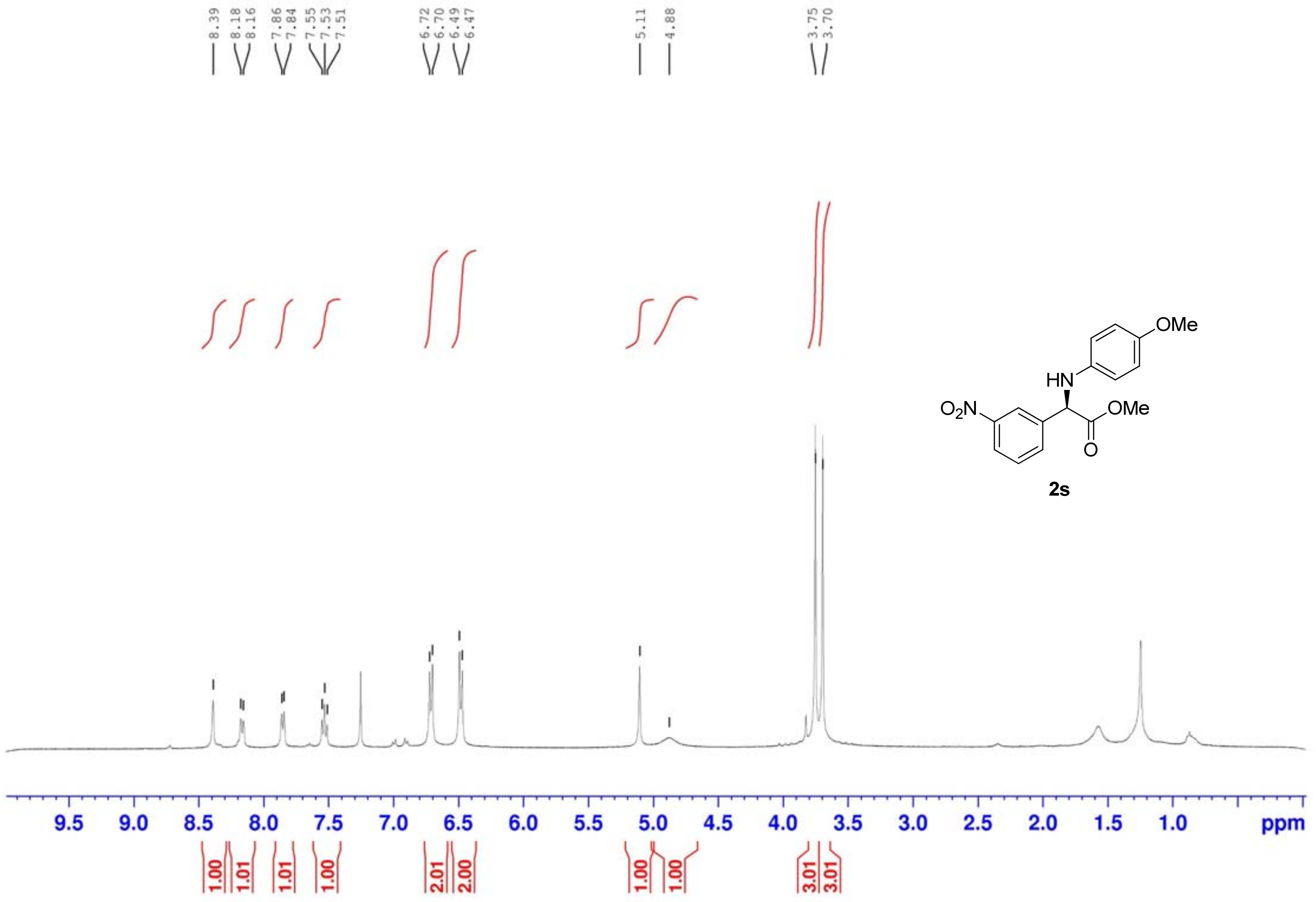

^{13}C NMR $CDCl_3$ (150 MHz)

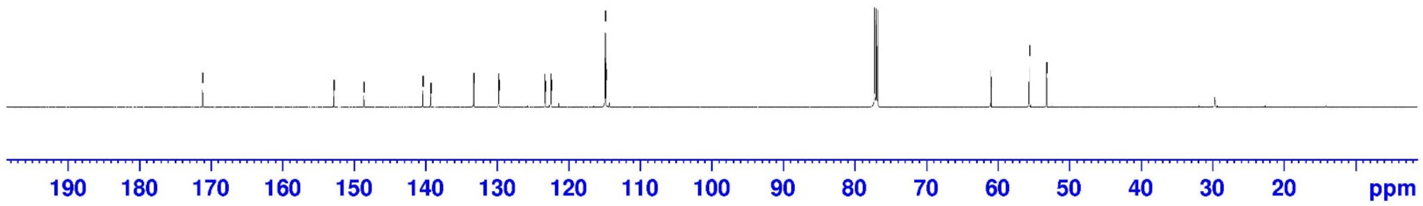

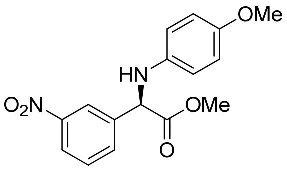

2s

^{1}H NMR $CDCl_3$ (400 MHz)

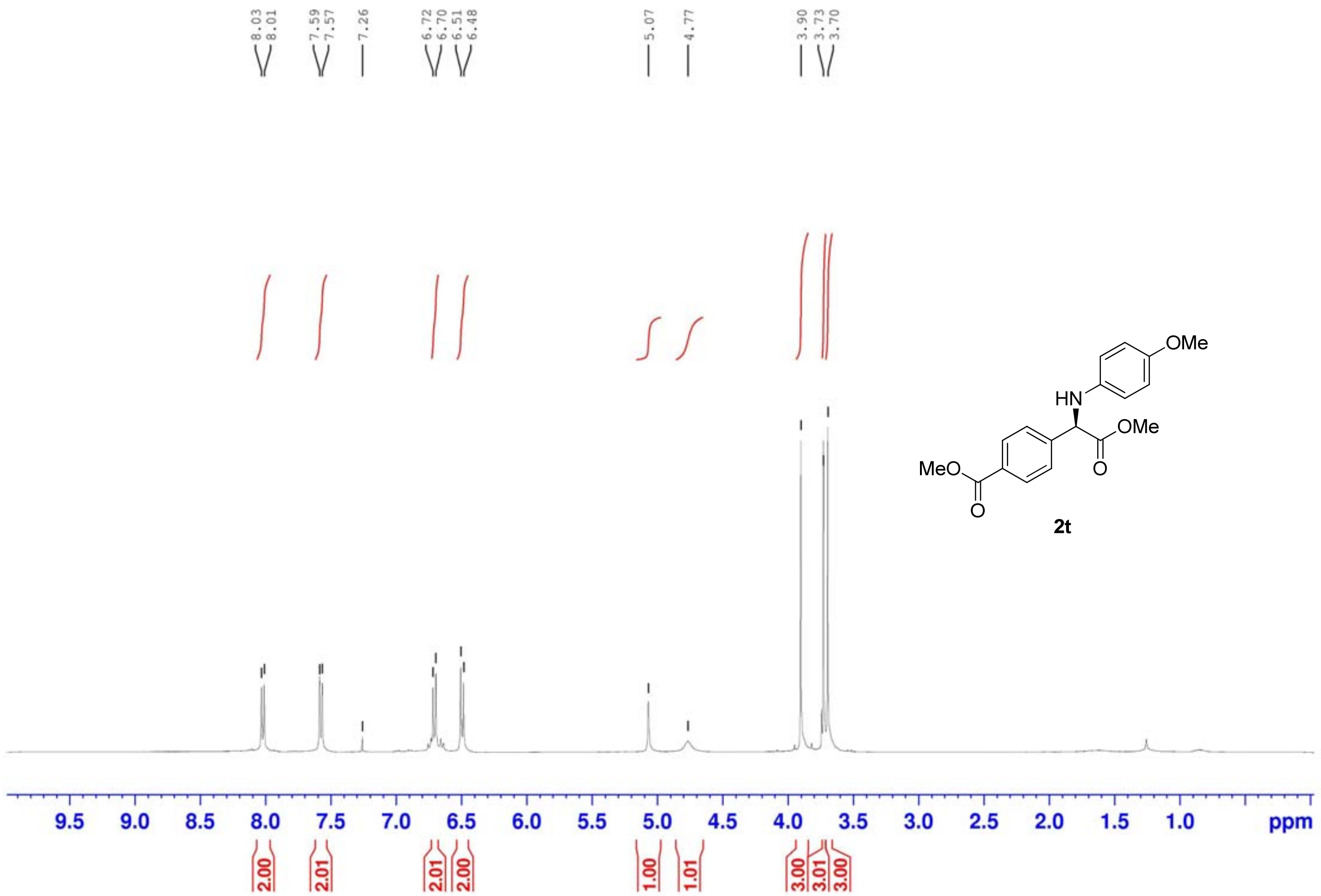

2t

^{13}C NMR $CDCl_3$ (100 MHz)

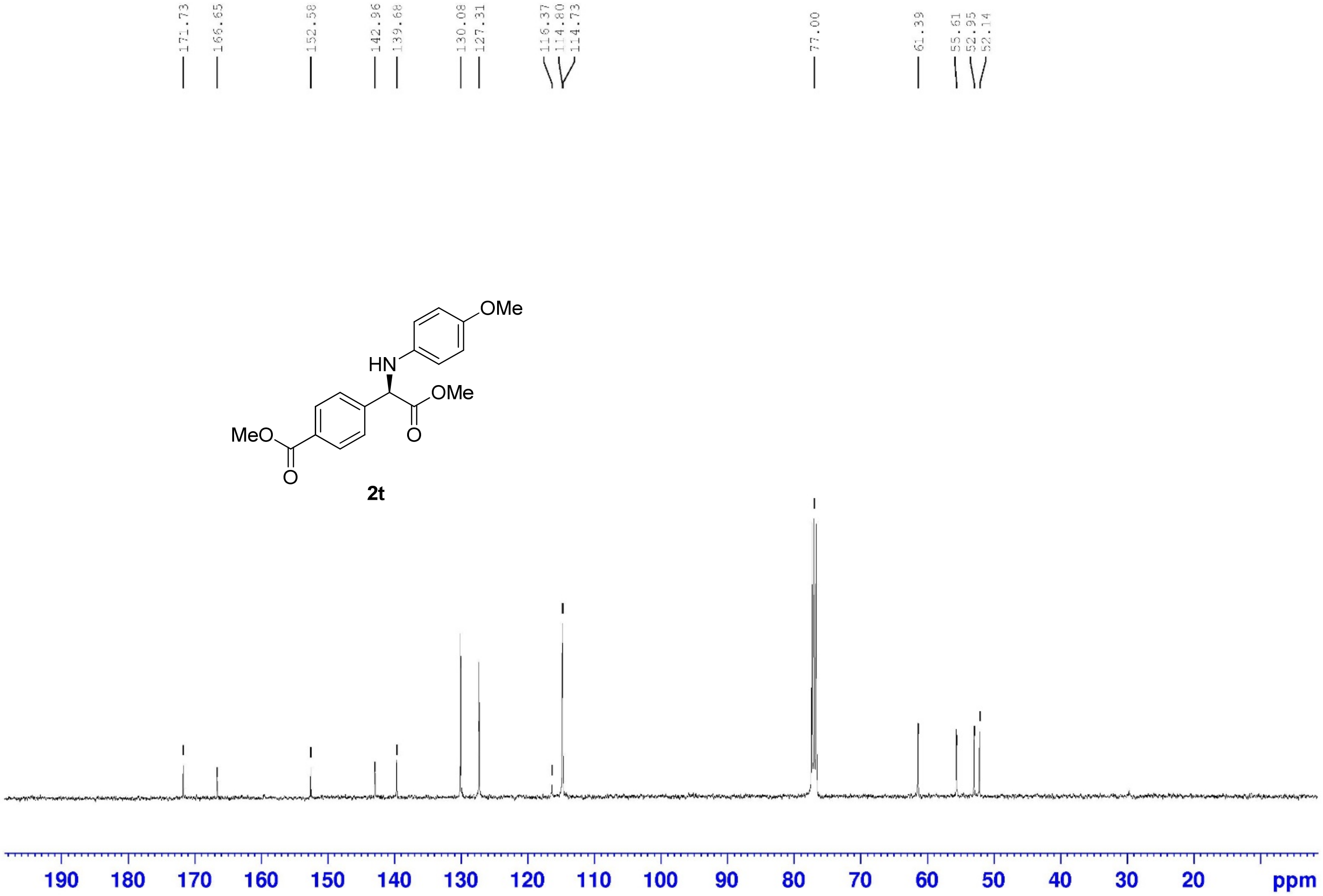

2t

^{1}H NMR $CDCl_3$ (600 MHz)

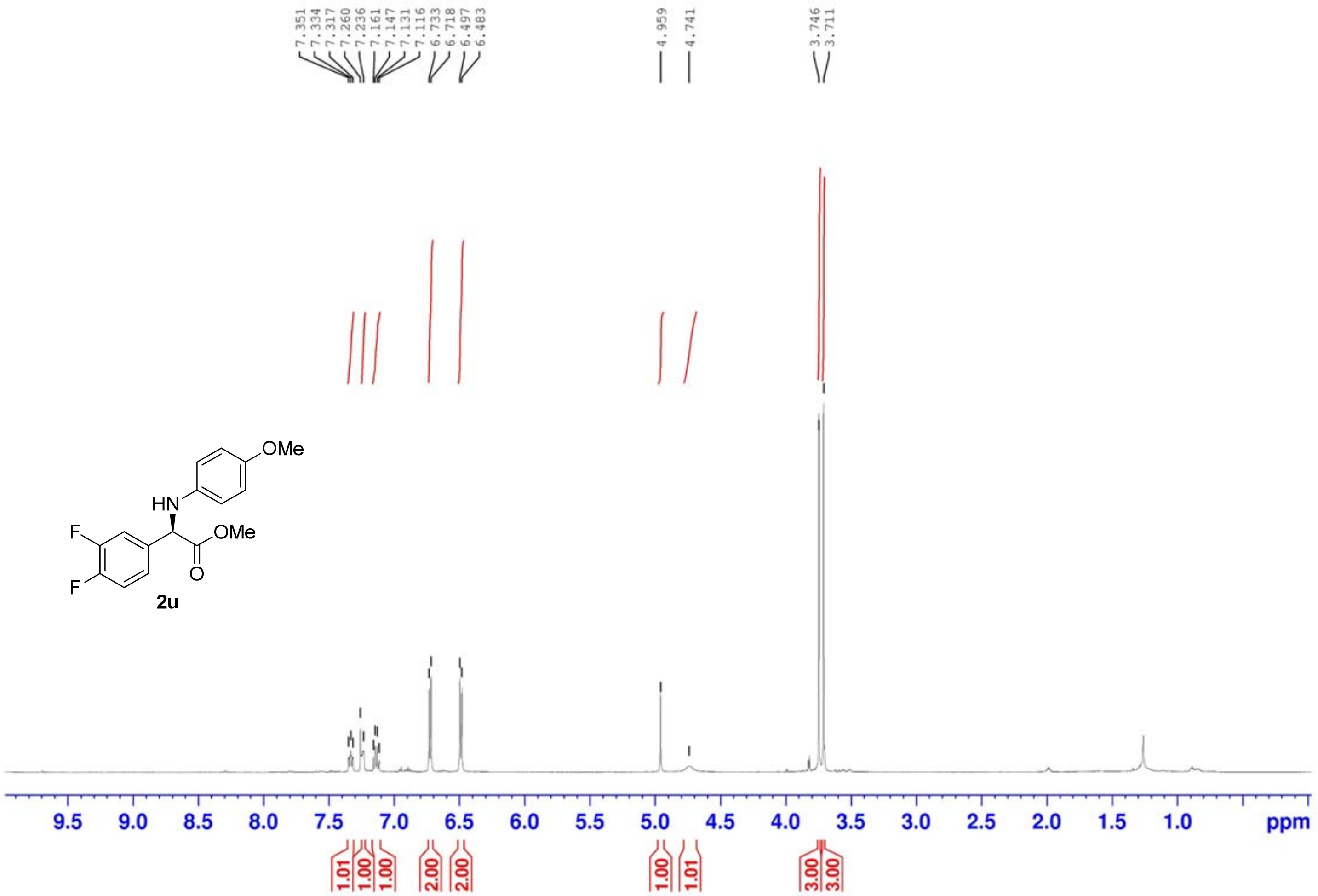

^{13}C NMR $CDCl_3$ (150 MHz)

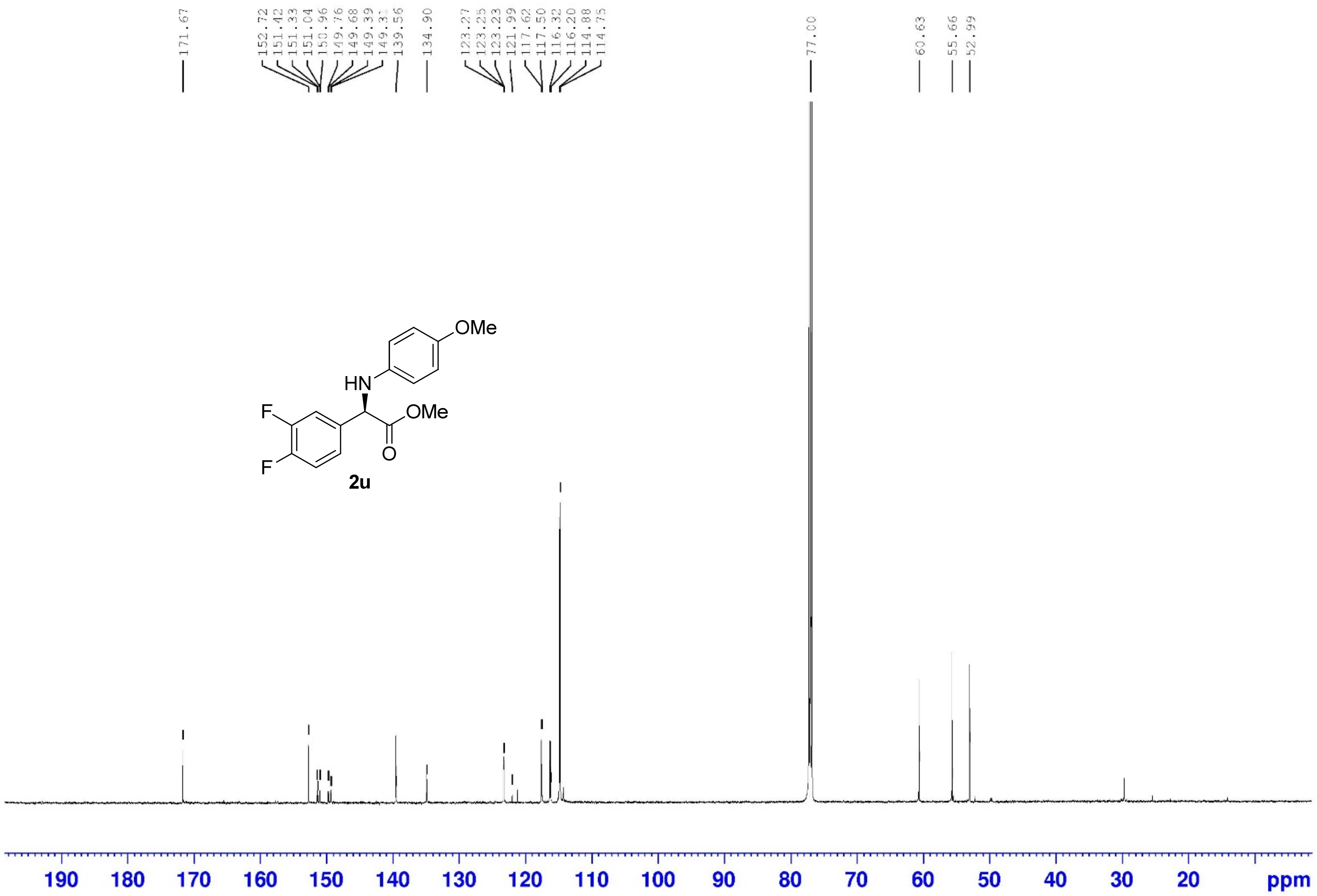

^1H NMR $CDCl_3$ (400 MHz)

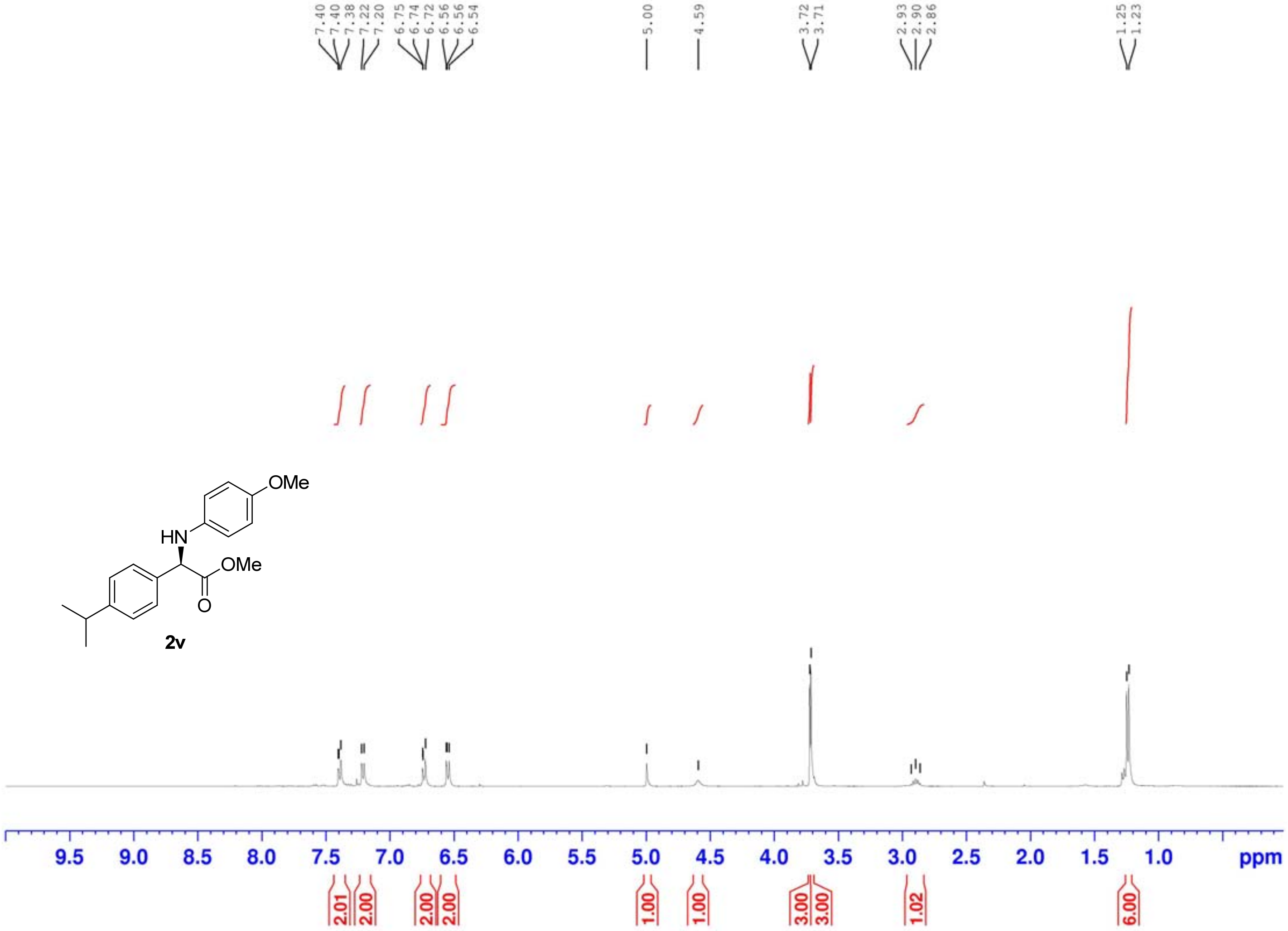

^{13}C NMR $CDCl_3$ (100 MHz)

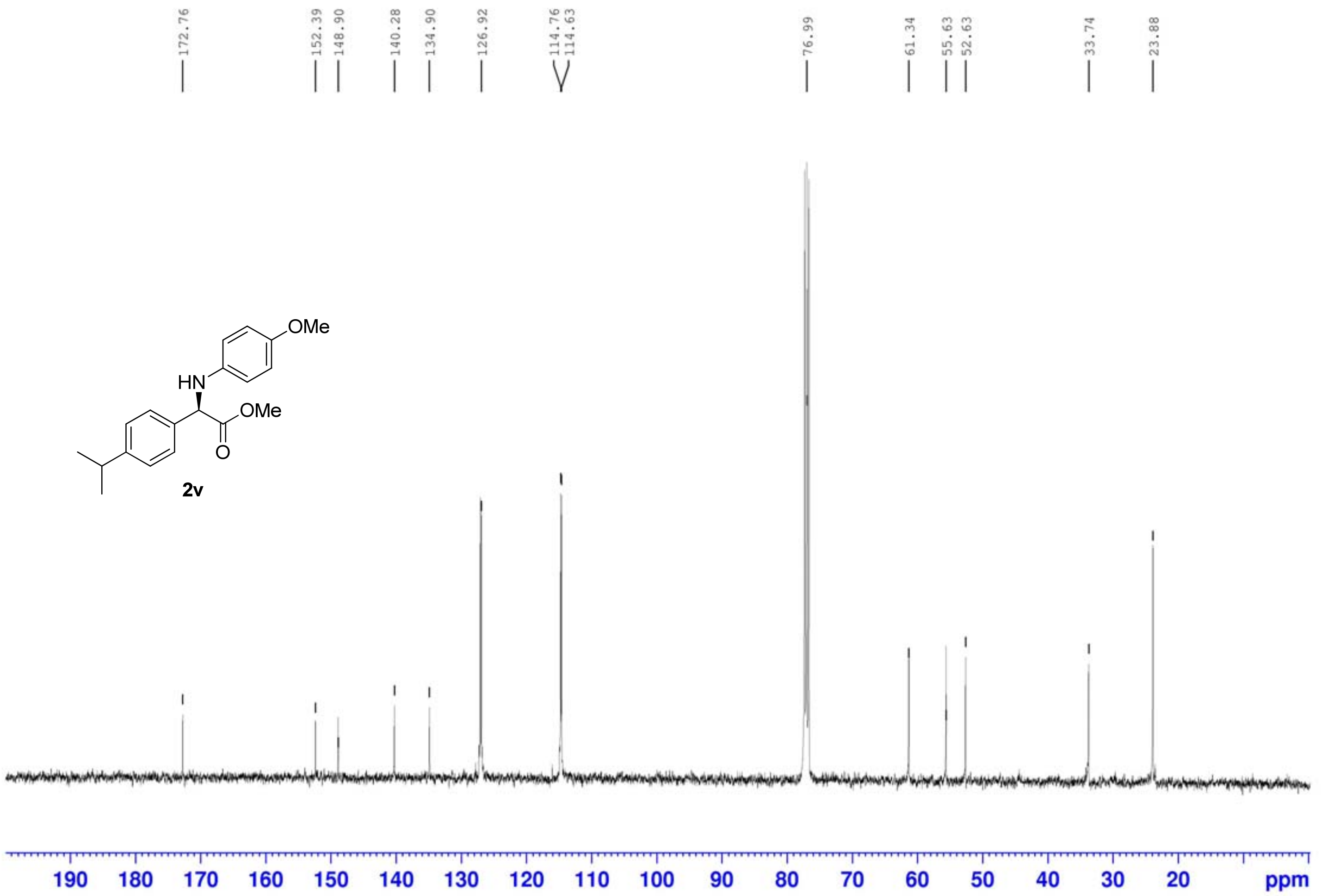

^{1}H NMR $CDCl_3$ (600 MHz)

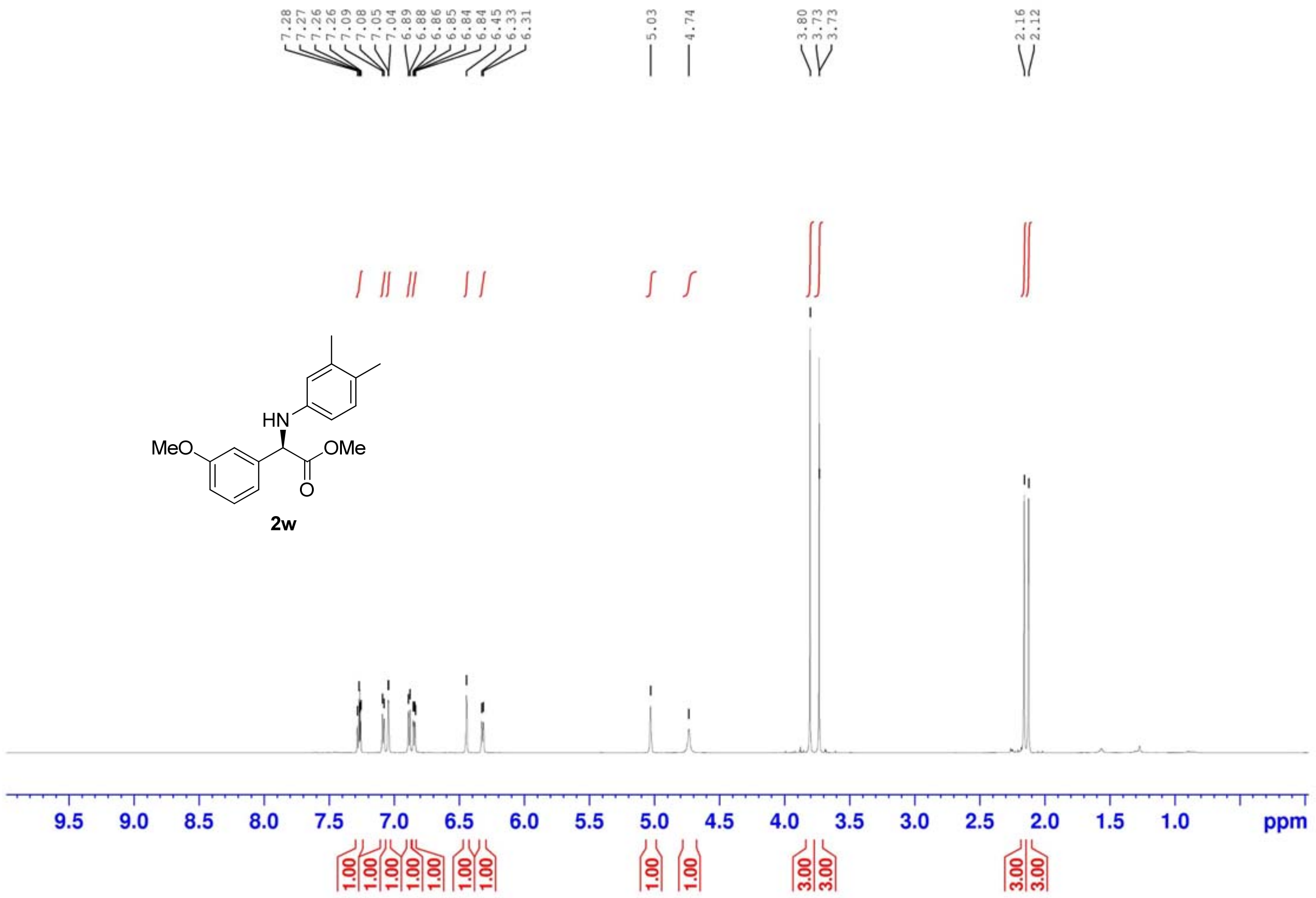

^{13}C NMR $CDCl_3$ (100 MHz)

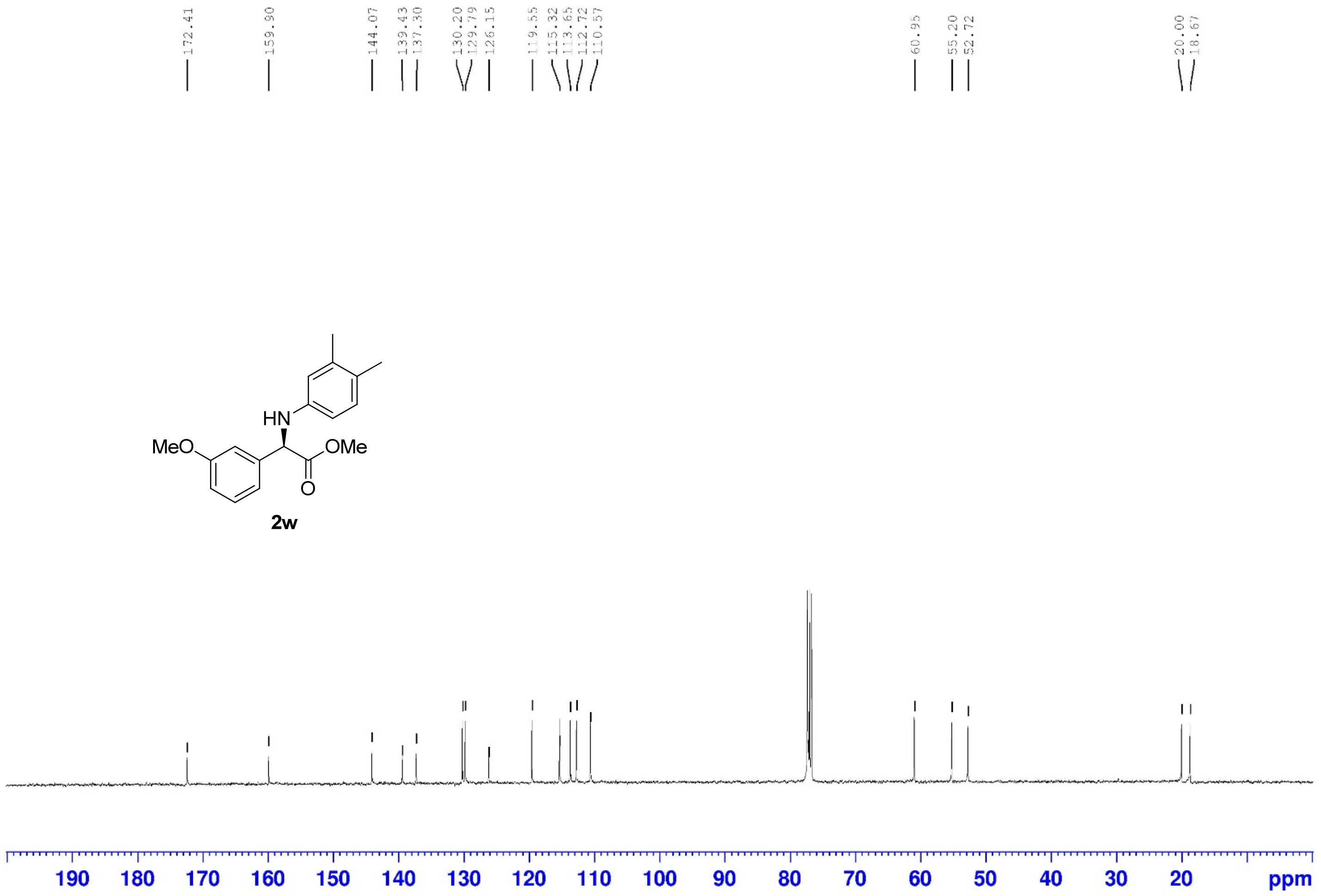

^{1}H NMR $CDCl_3$ (600 MHz)

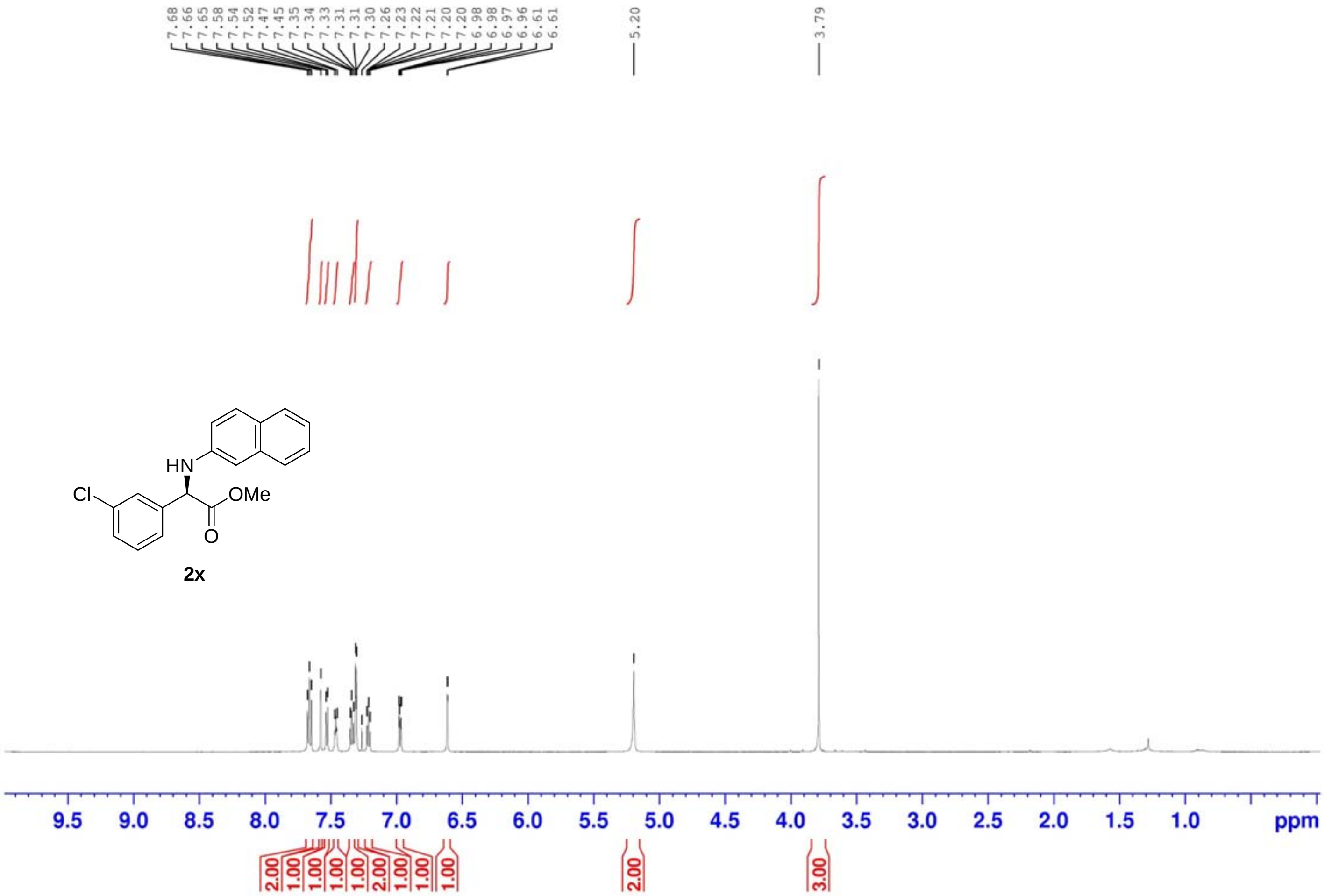

^{13}C NMR $CDCl_3$ (150 MHz)

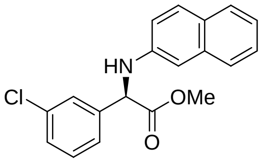

2x

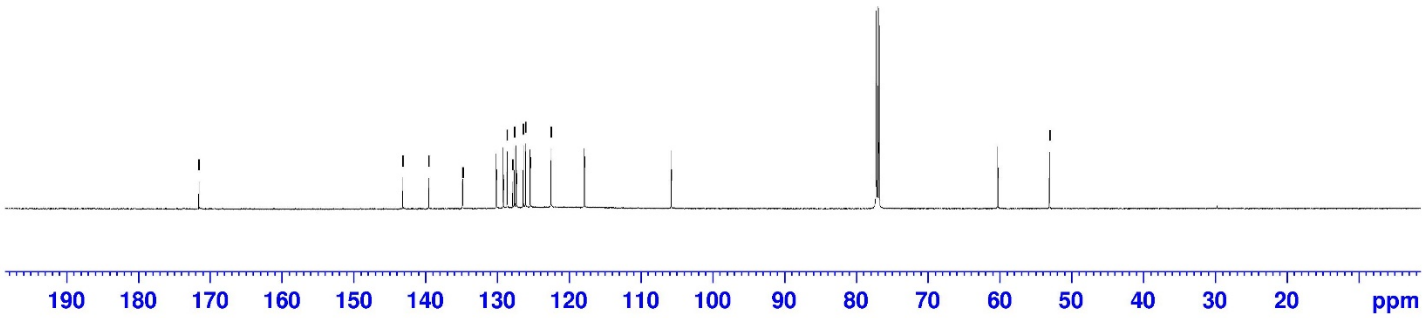

^{1}H NMR $CDCl_3$ (600 MHz)

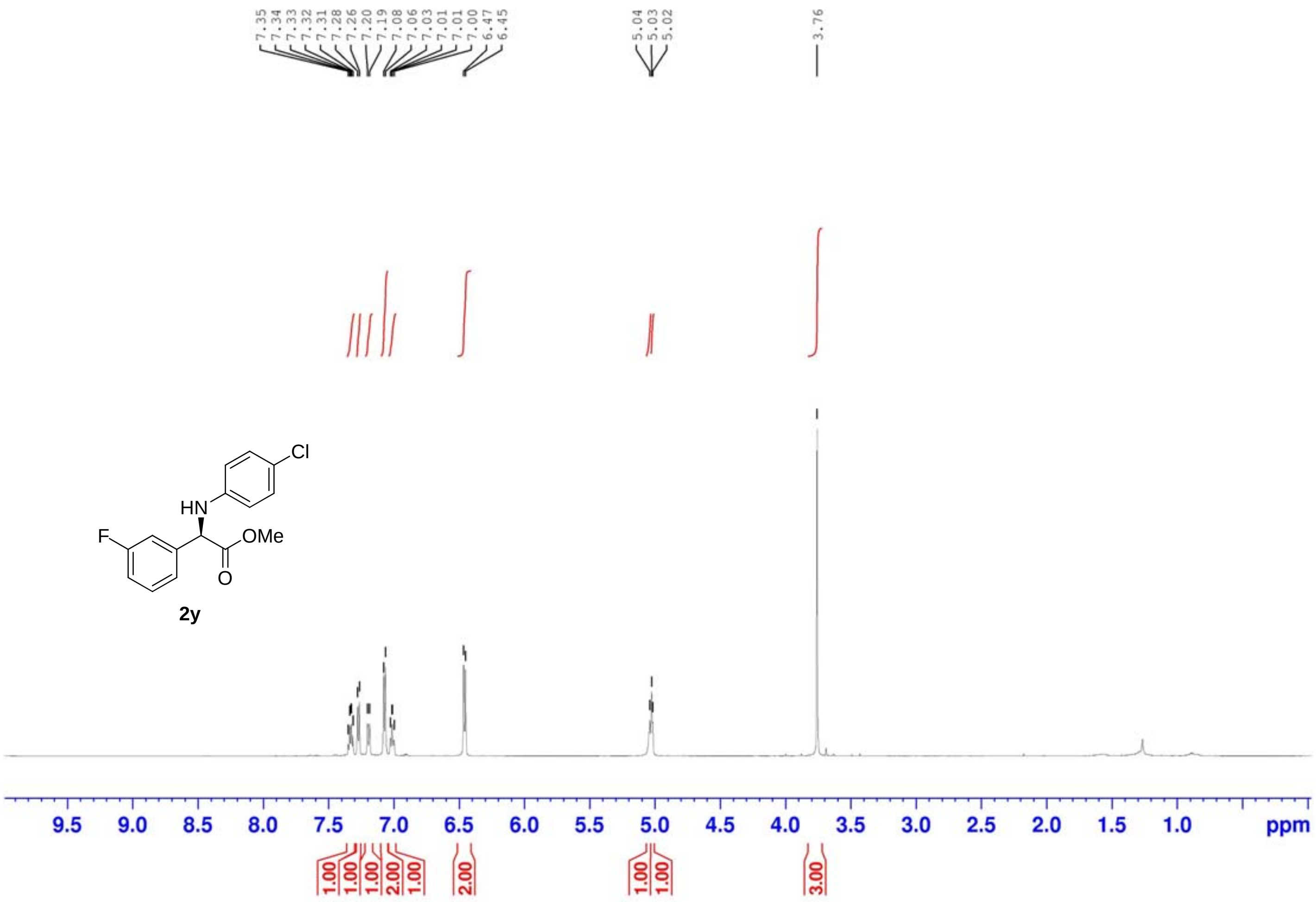

^{13}C NMR $CDCl_3$ (MHz)

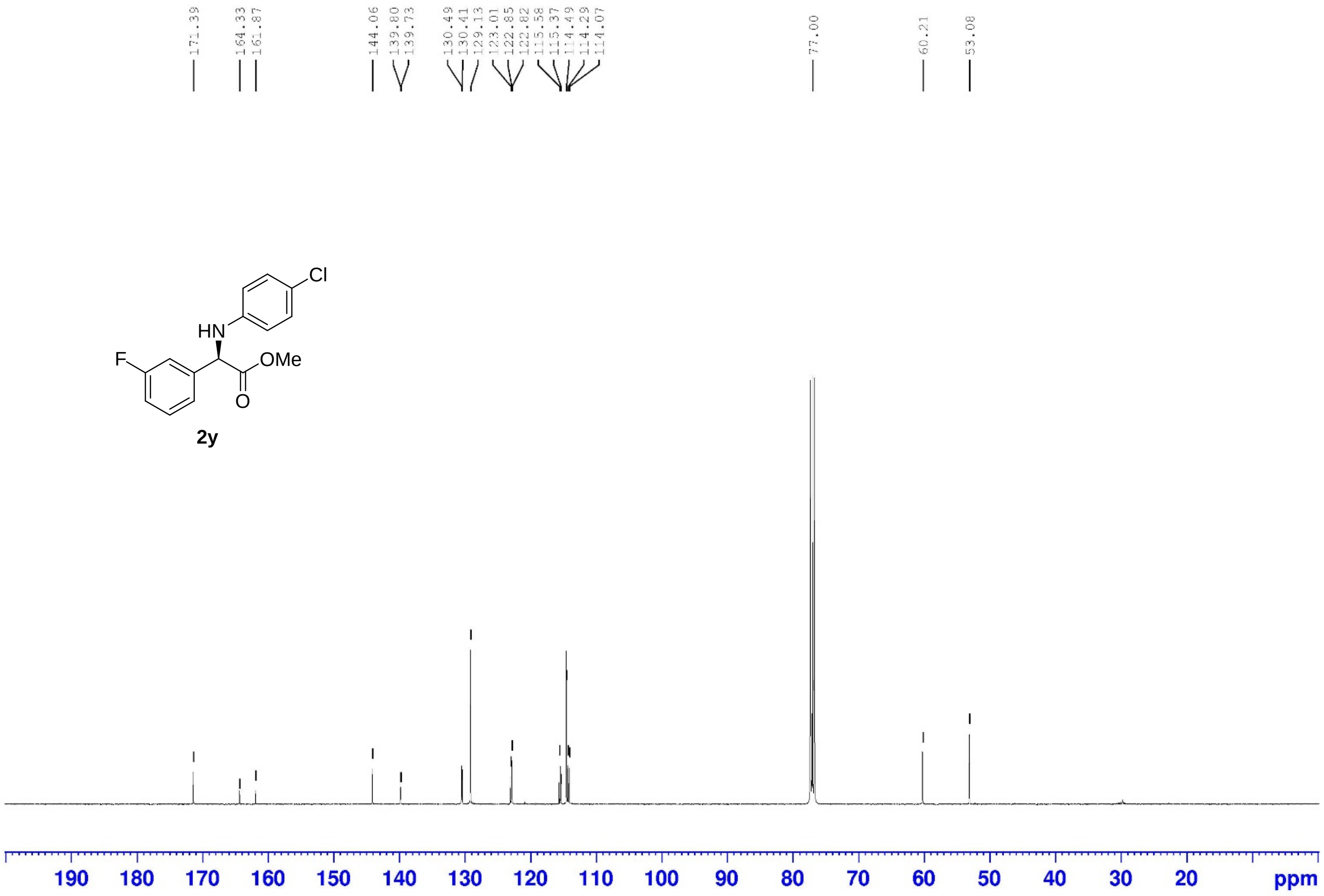

^{1}H NMR $CDCl_3$ (600MHz)

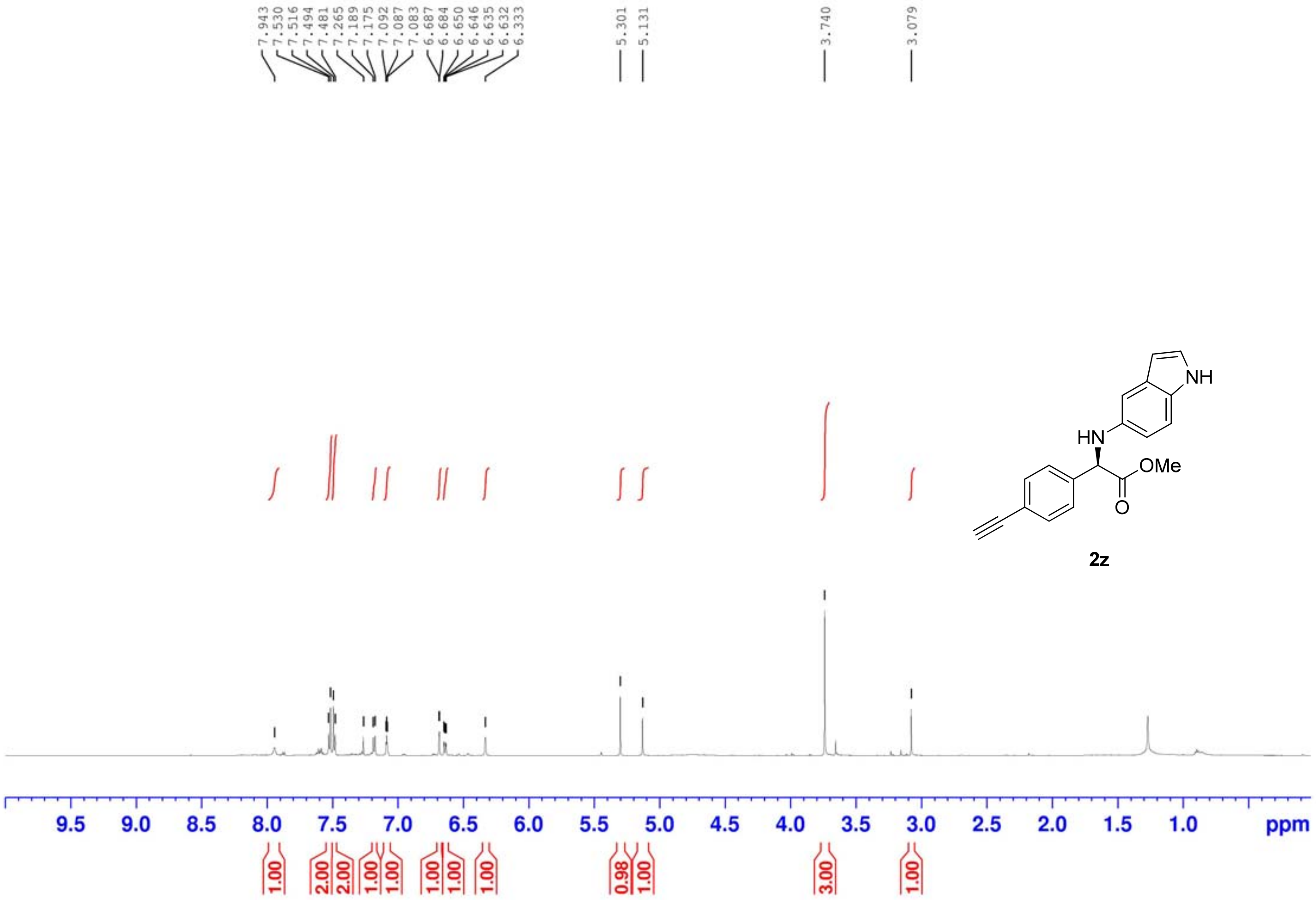

2z

^{13}C NMR $CDCl_3$ (150 MHz)

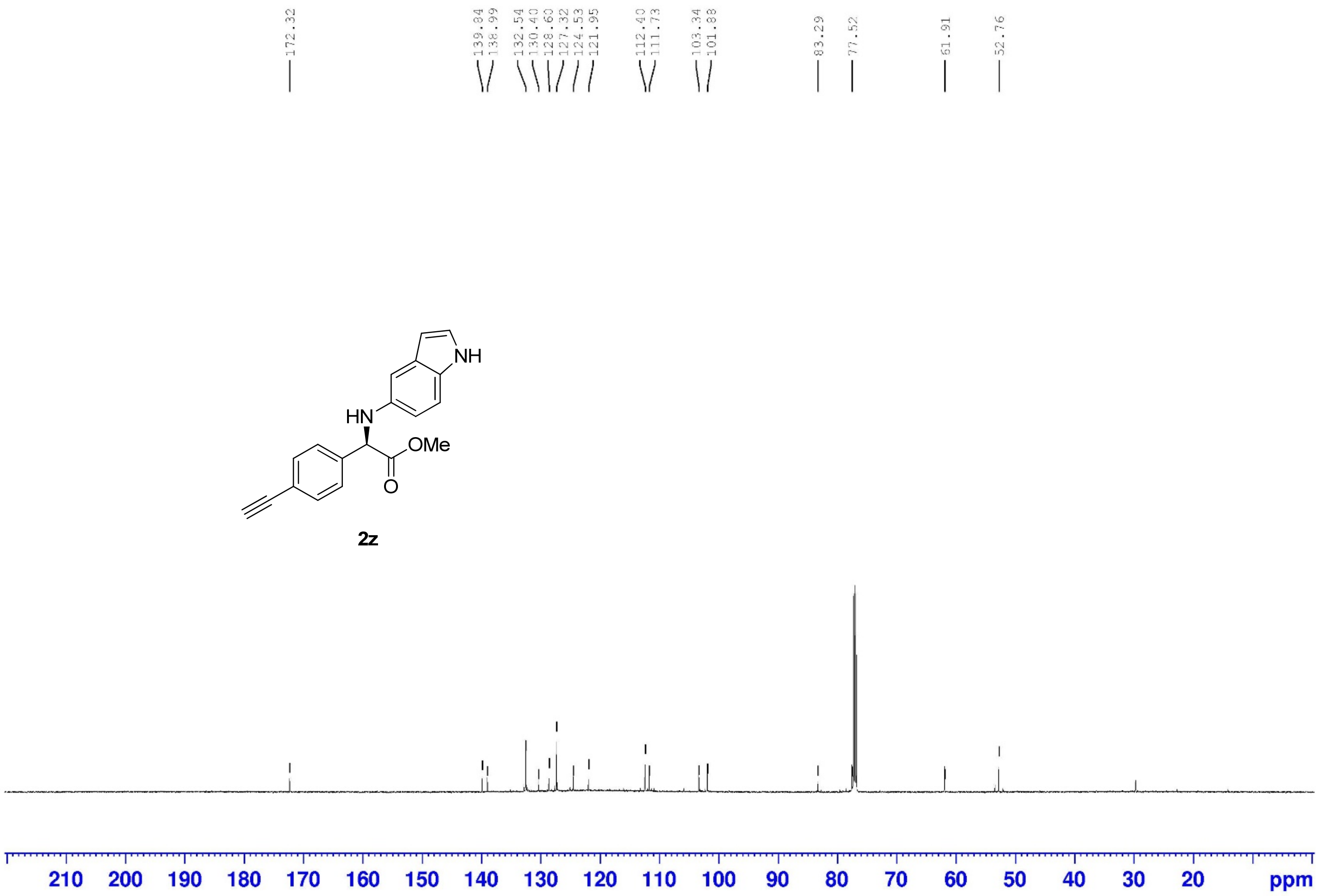

^{1}H NMR $CDCl_3$ (600 MHz)

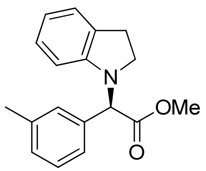

2aa

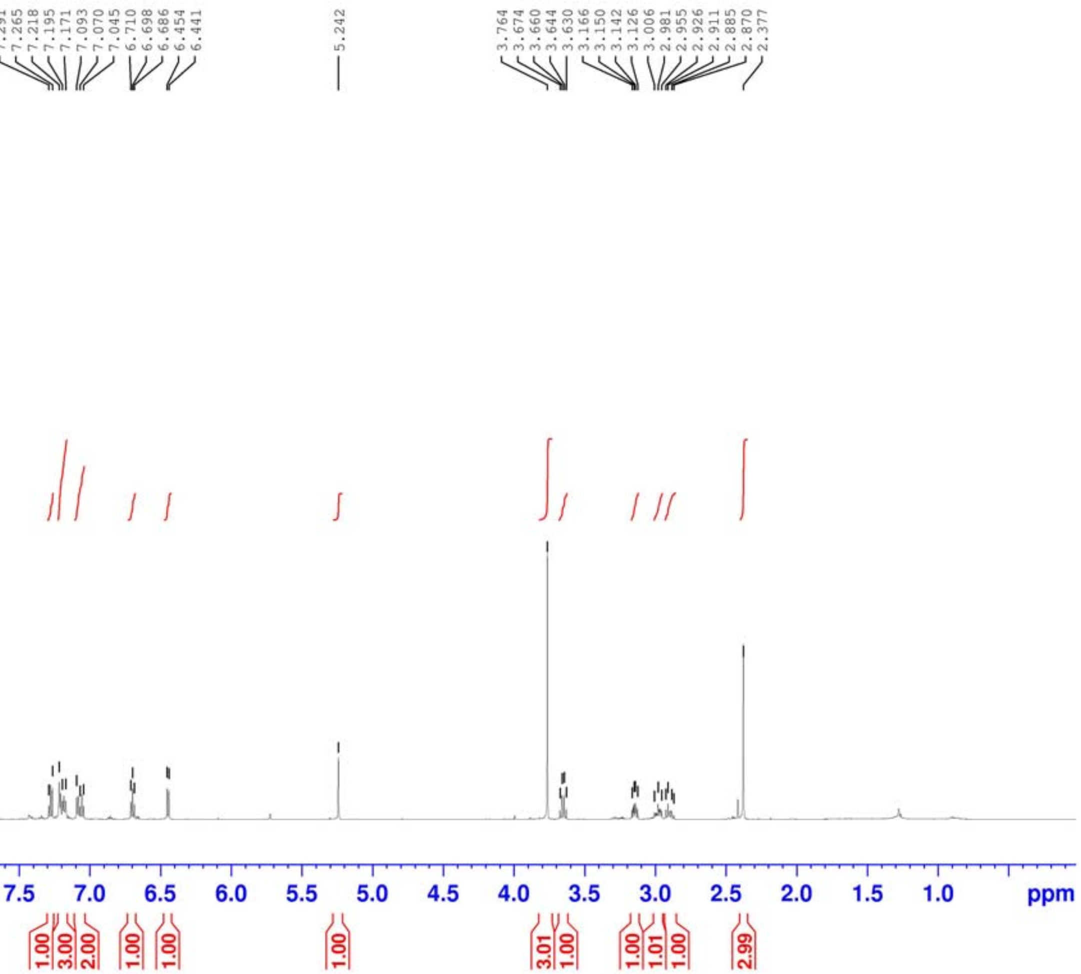

^{13}C NMR $CDCl_3$ (150 MHz)

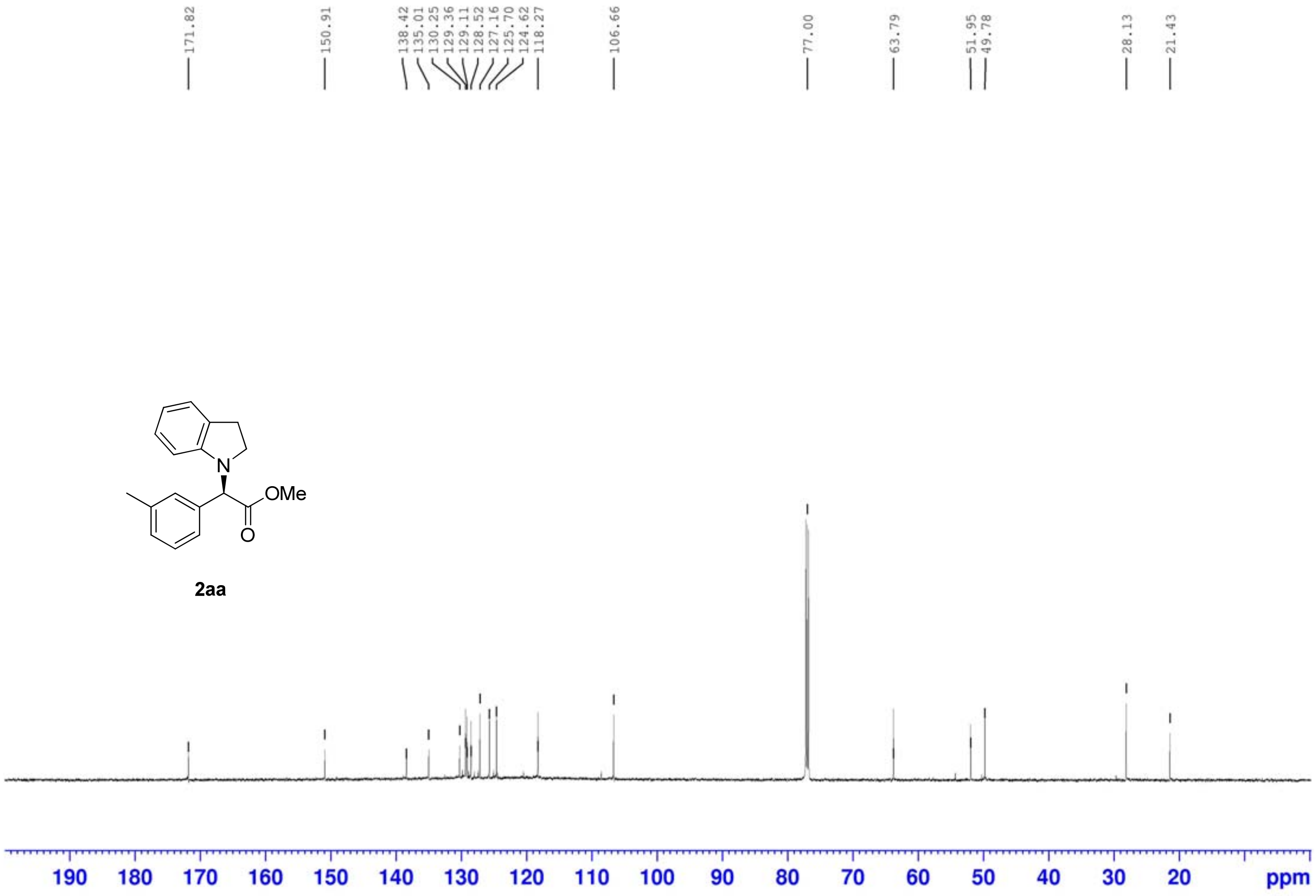

^{1}H NMR $CDCl_3$ (400 MHz)

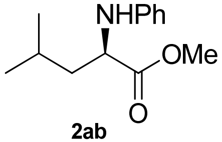

2ab

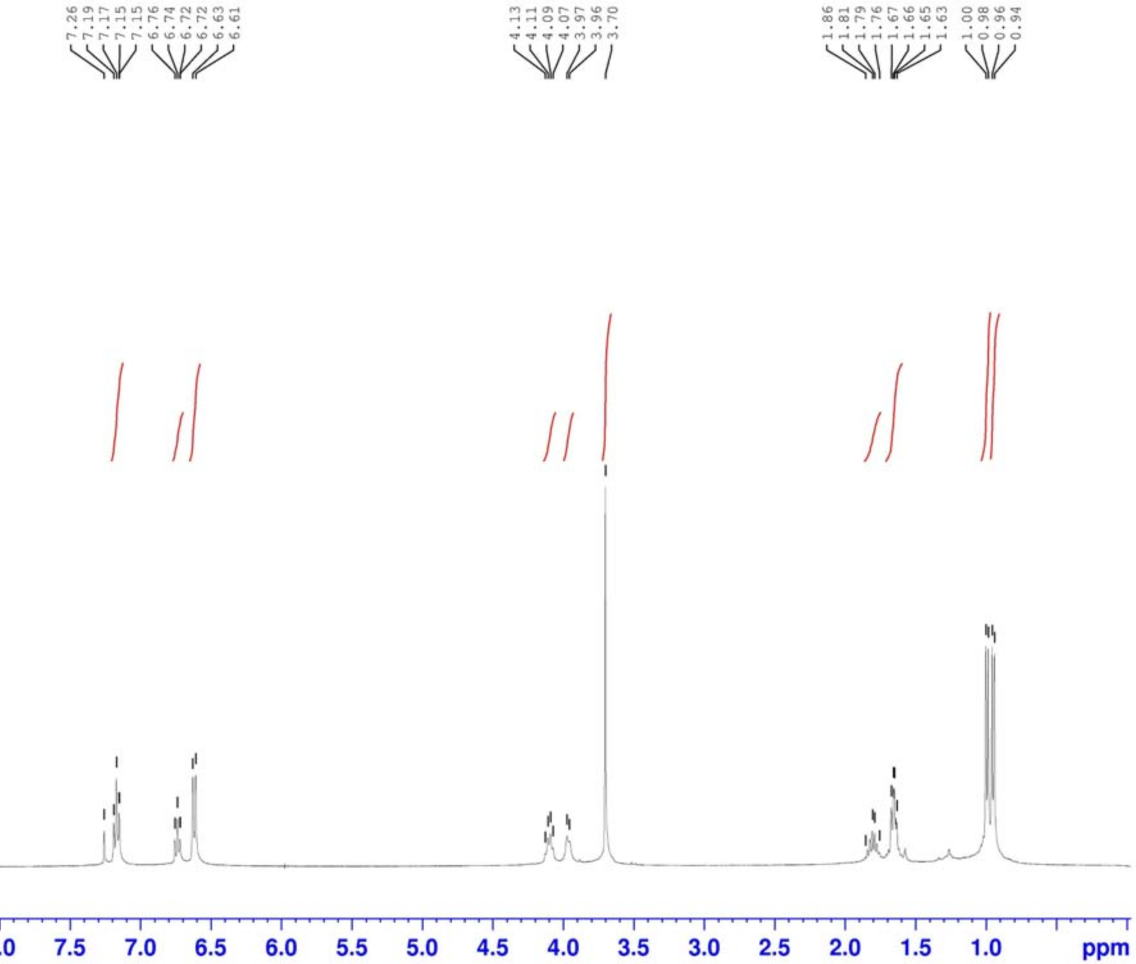

^{13}C NMR $CDCl_3$ (100 MHz)

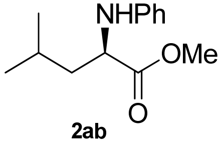

2ab

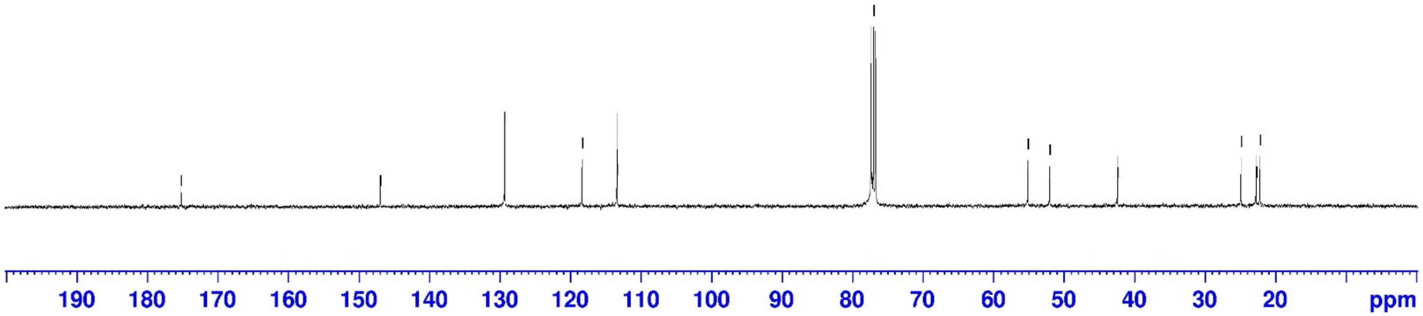

^{1}H NMR $CDCl_3$ (300 MHz)

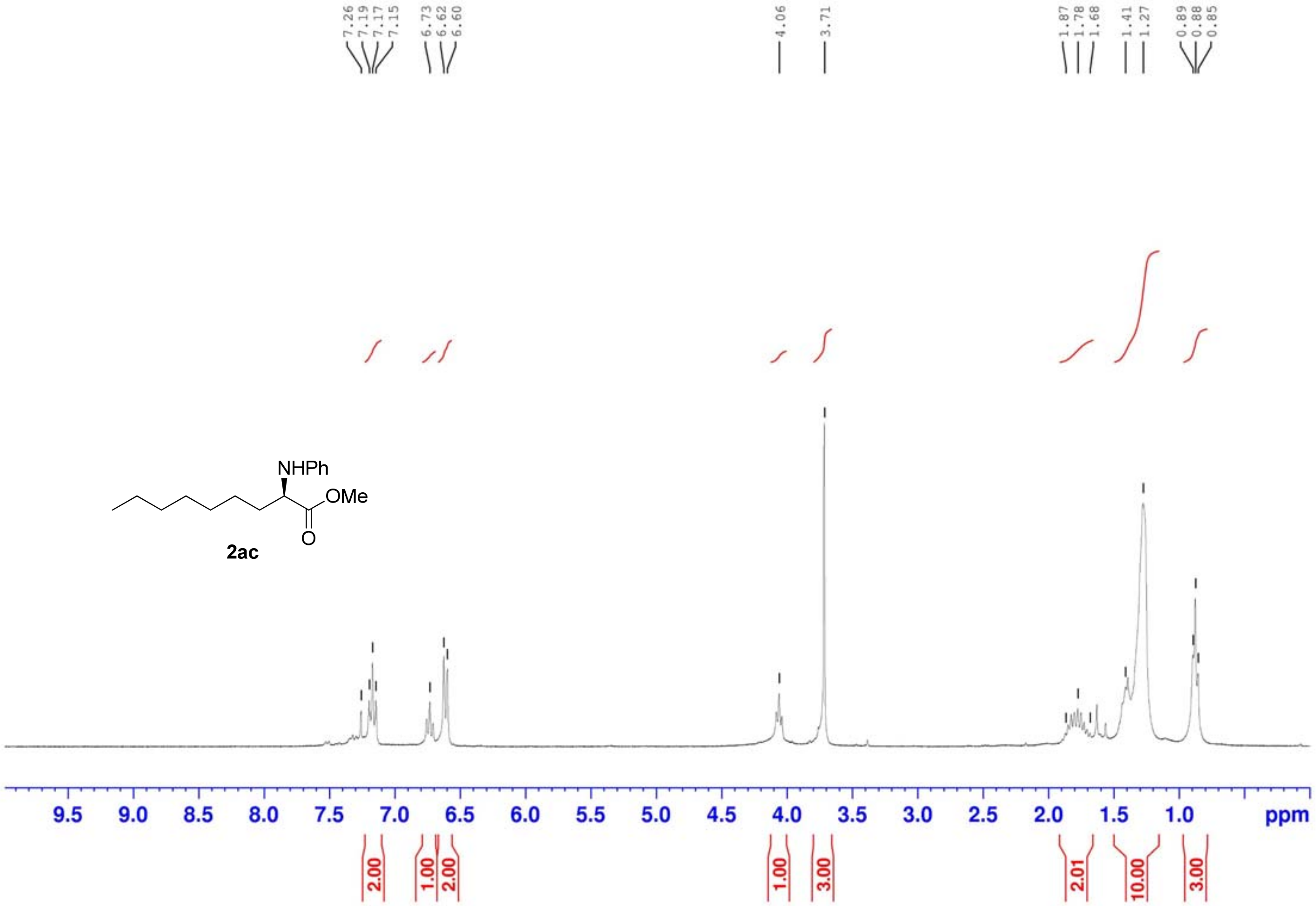

^{13}C NMR $CDCl_3$ (75 MHz)

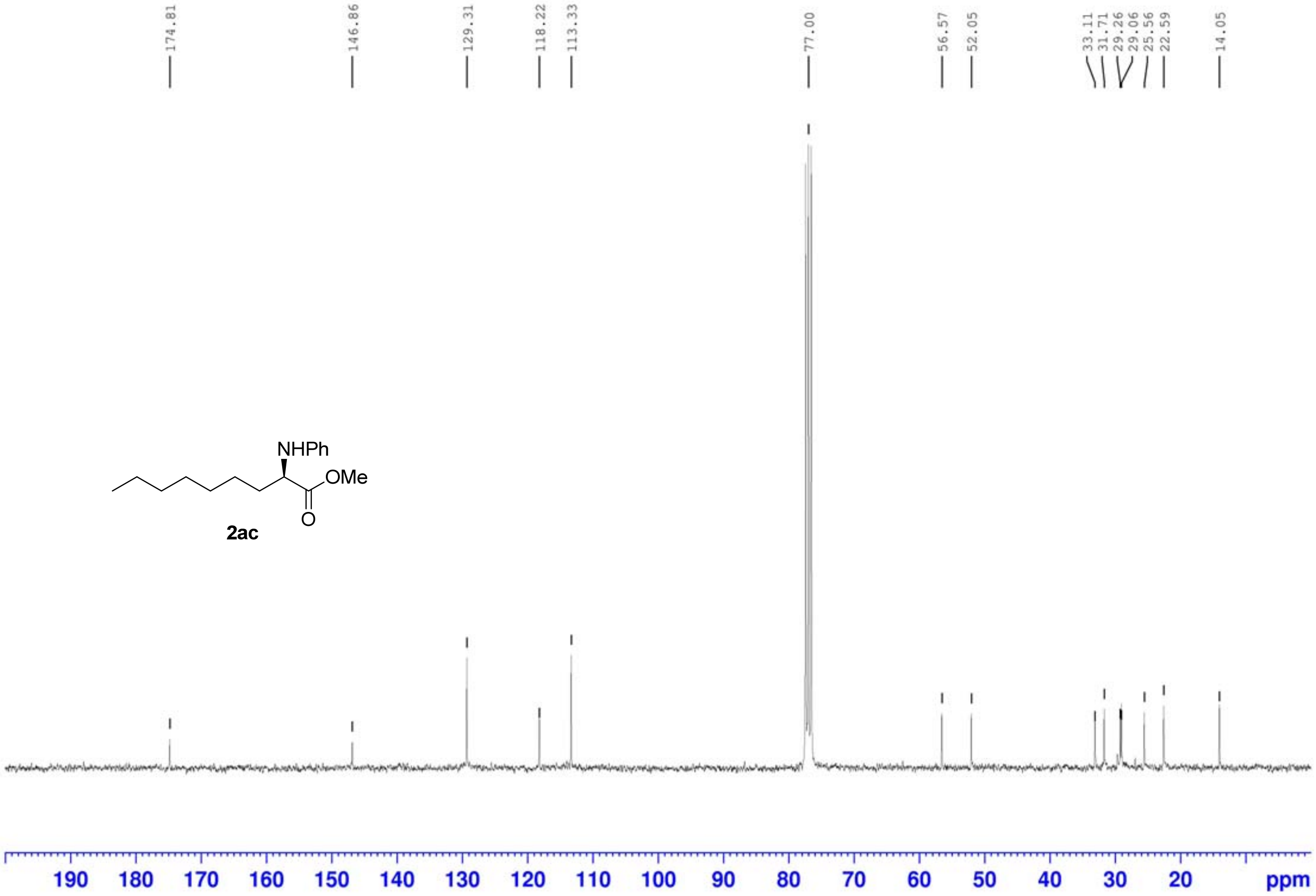

^{1}H NMR $CDCl_3$ (600 MHz)

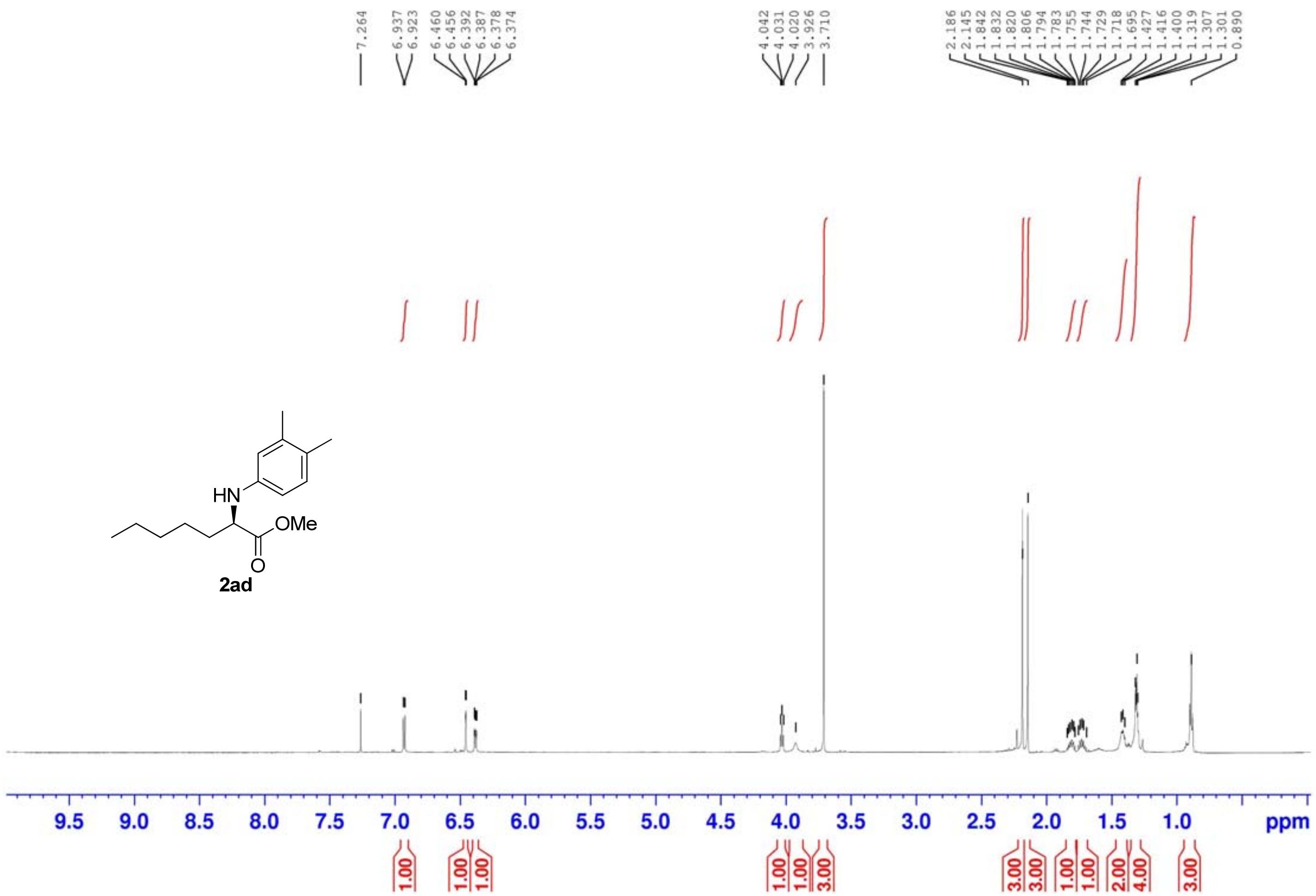

^{13}C NMR $CDCl_3$ (150 MHz)

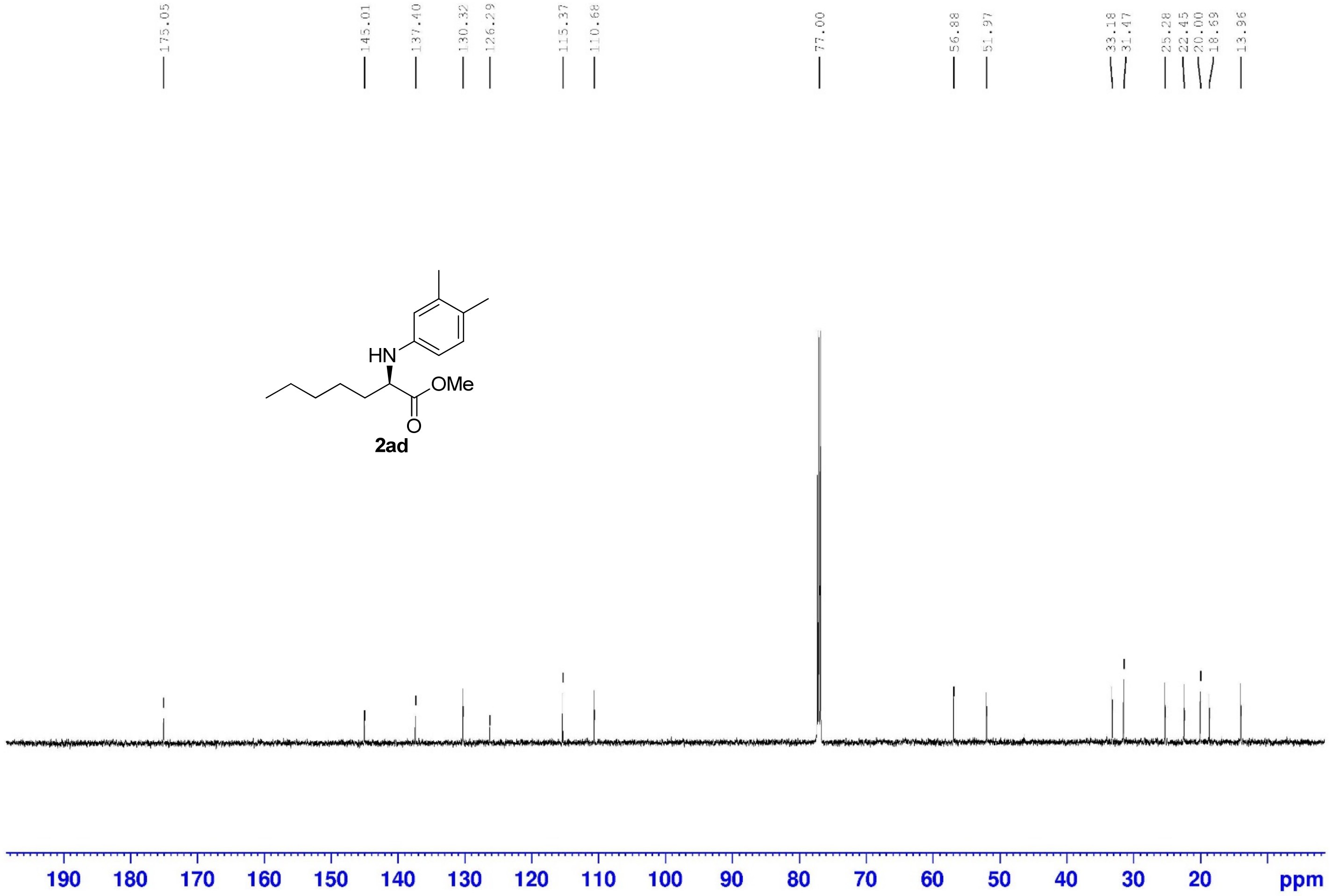

^{1}H NMR $CDCl_3$ (300 MHz)

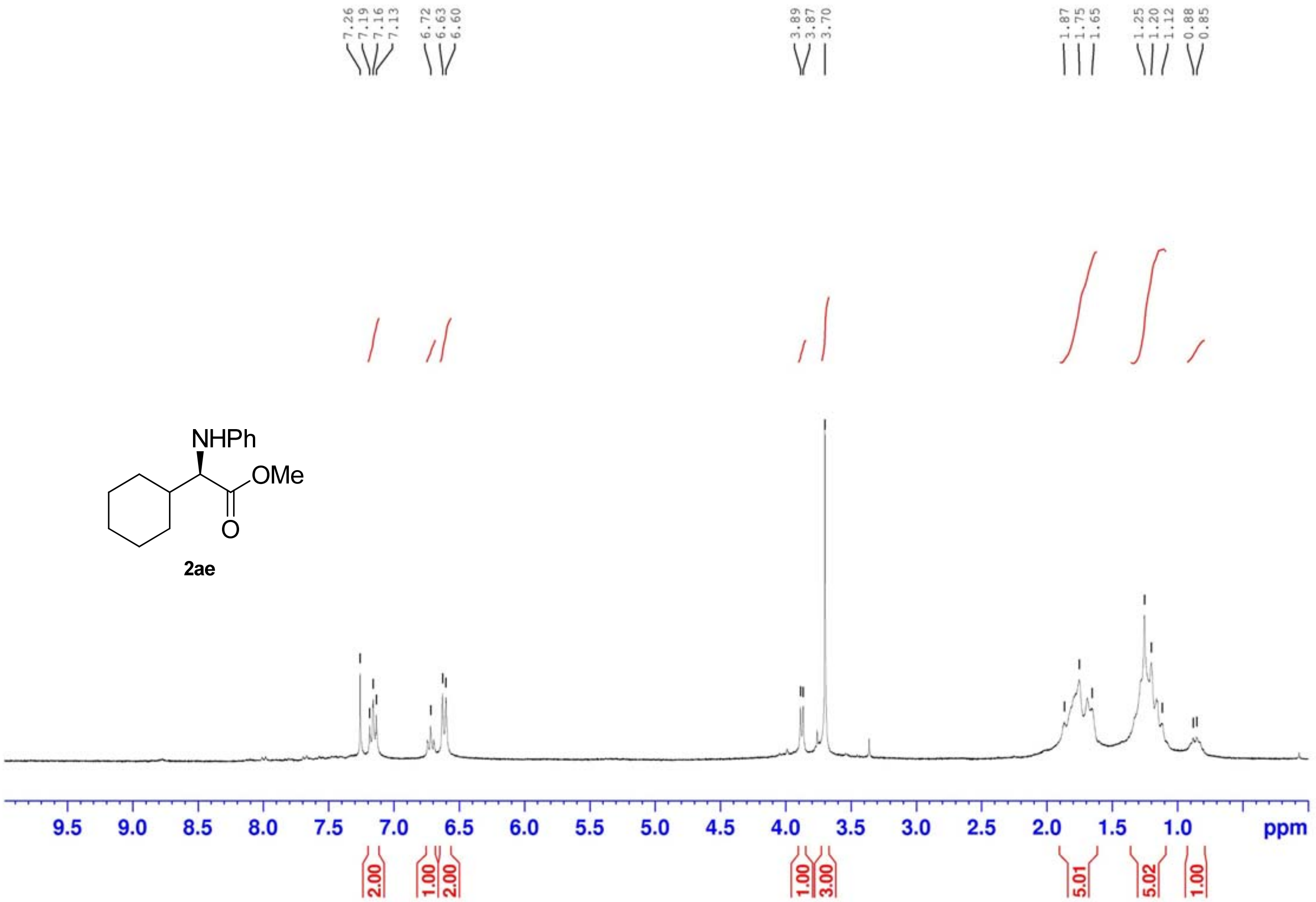

^{13}C NMR $CDCl_3$ (75 MHz)

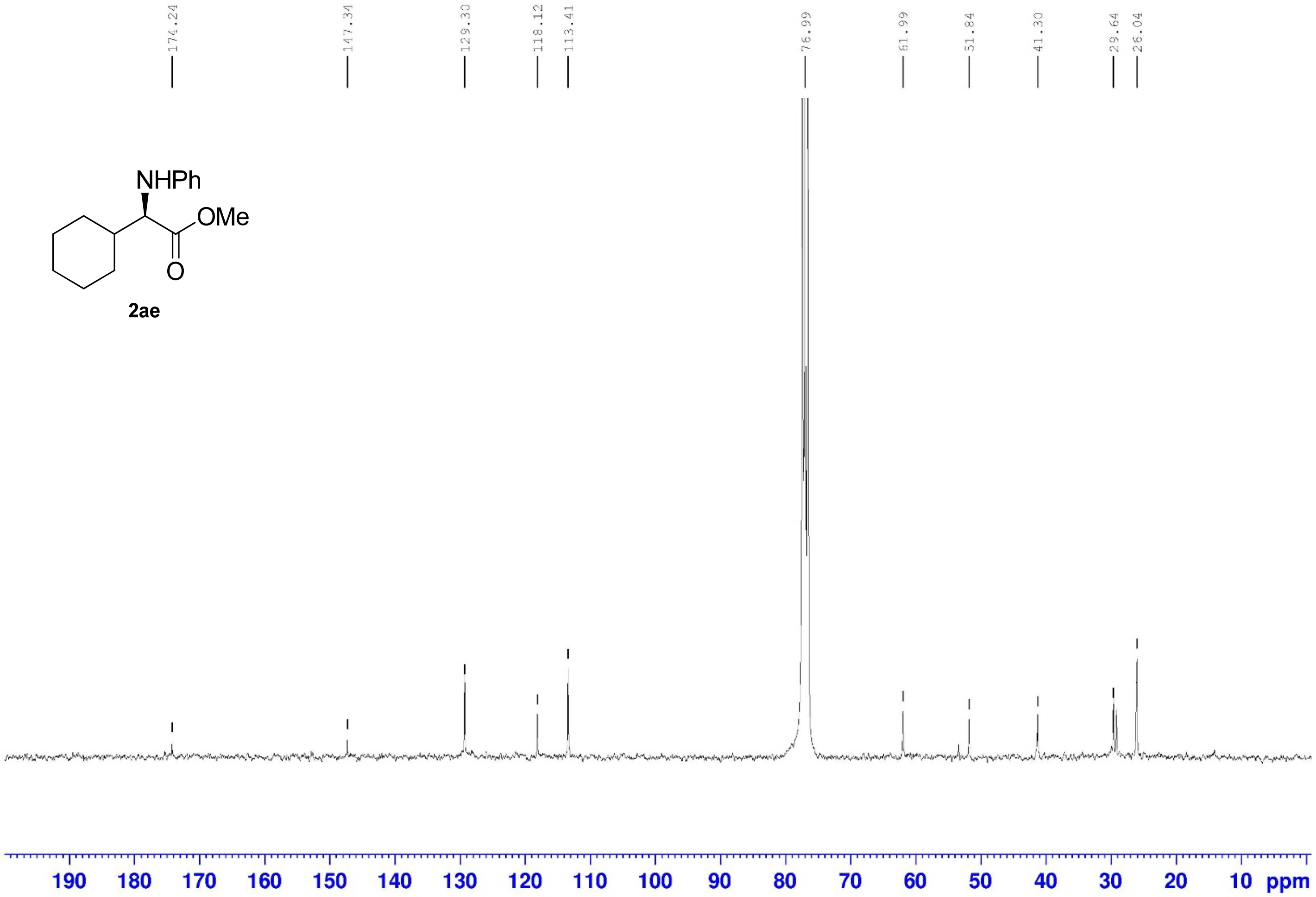

^{1}H NMR $CDCl_3$ (400 MHz)

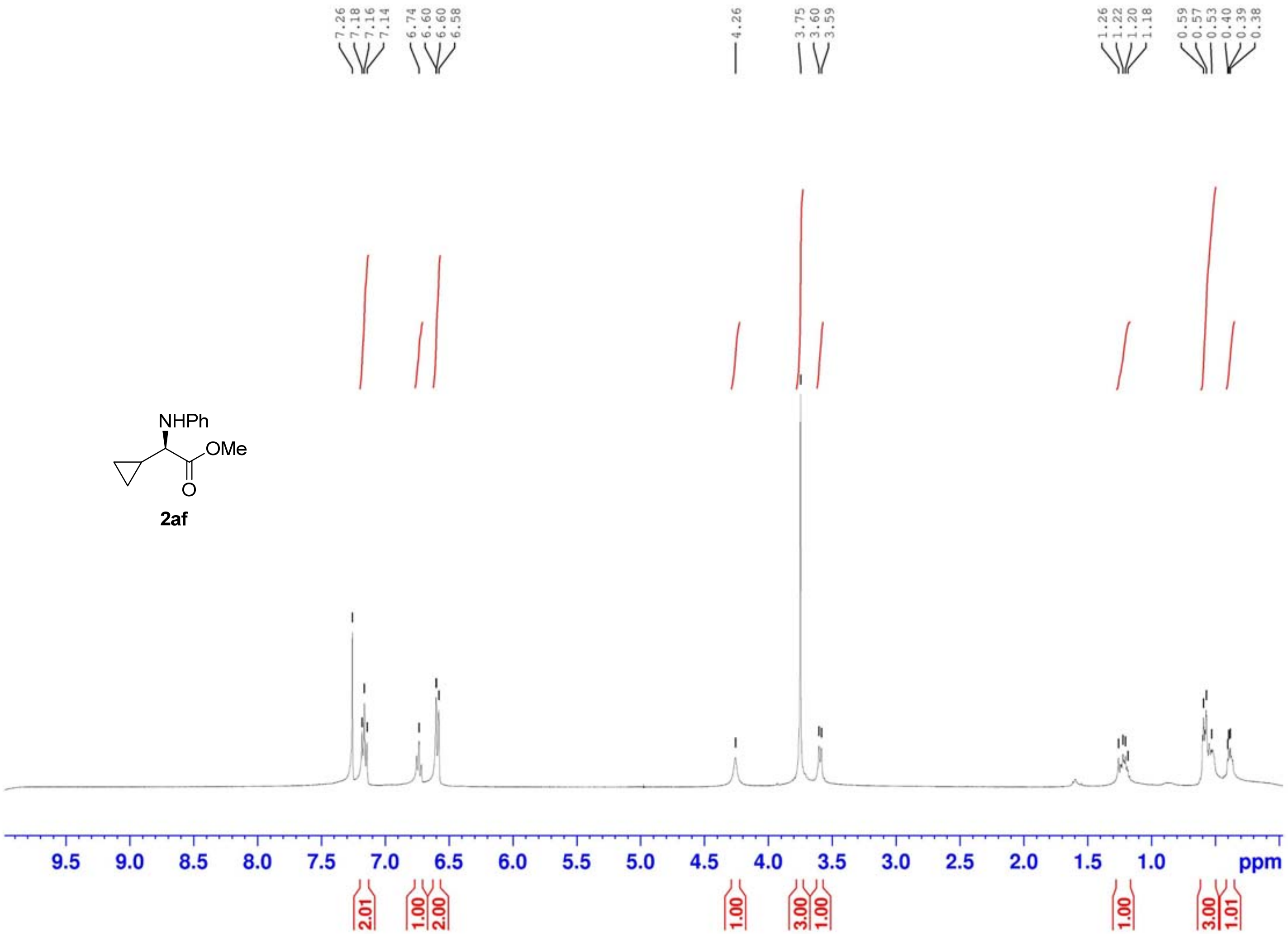

2af

^{13}C NMR $CDCl_3$ (100 MHz)

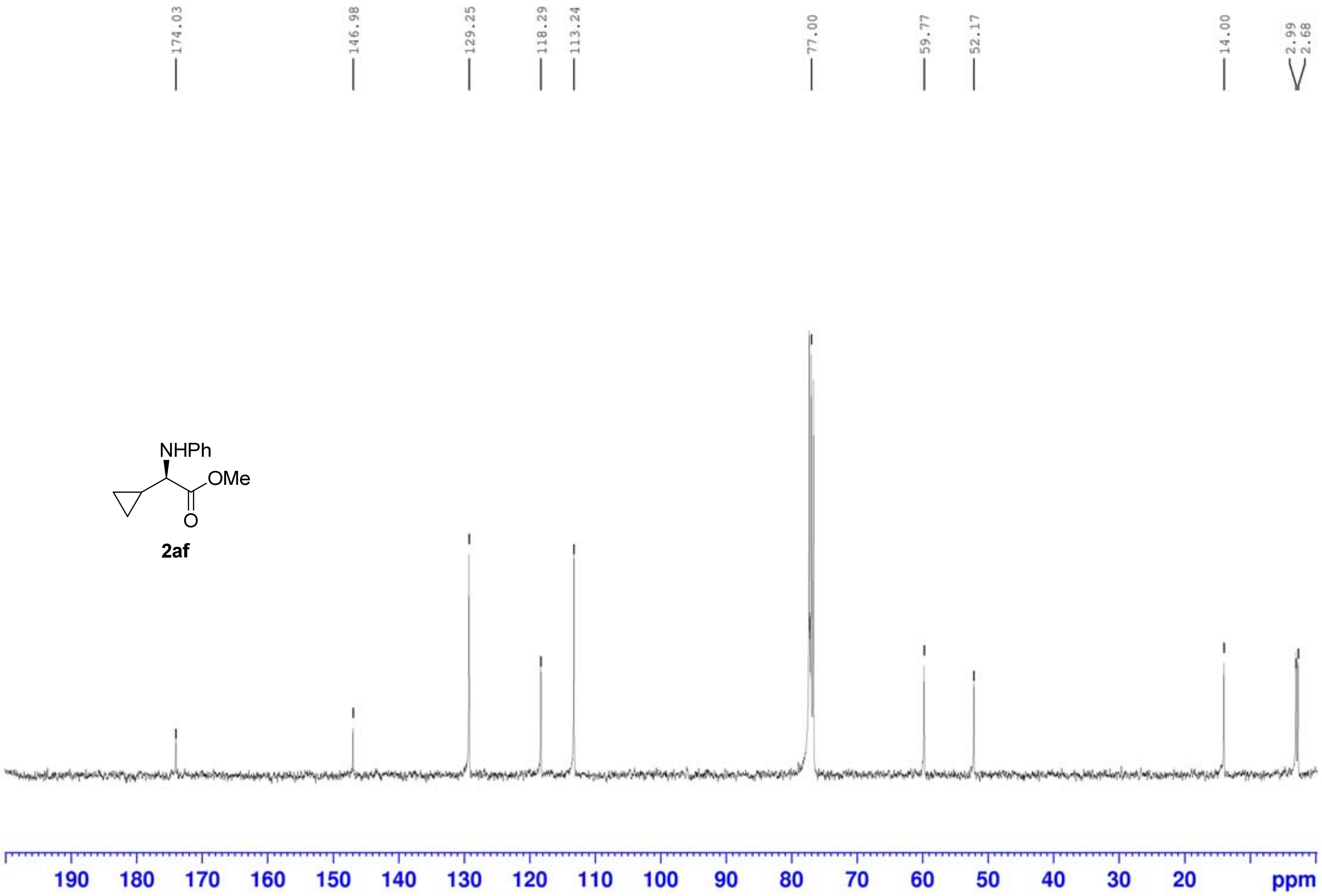

2af

^{1}H NMR $CDCl_3$ (400 MHz)

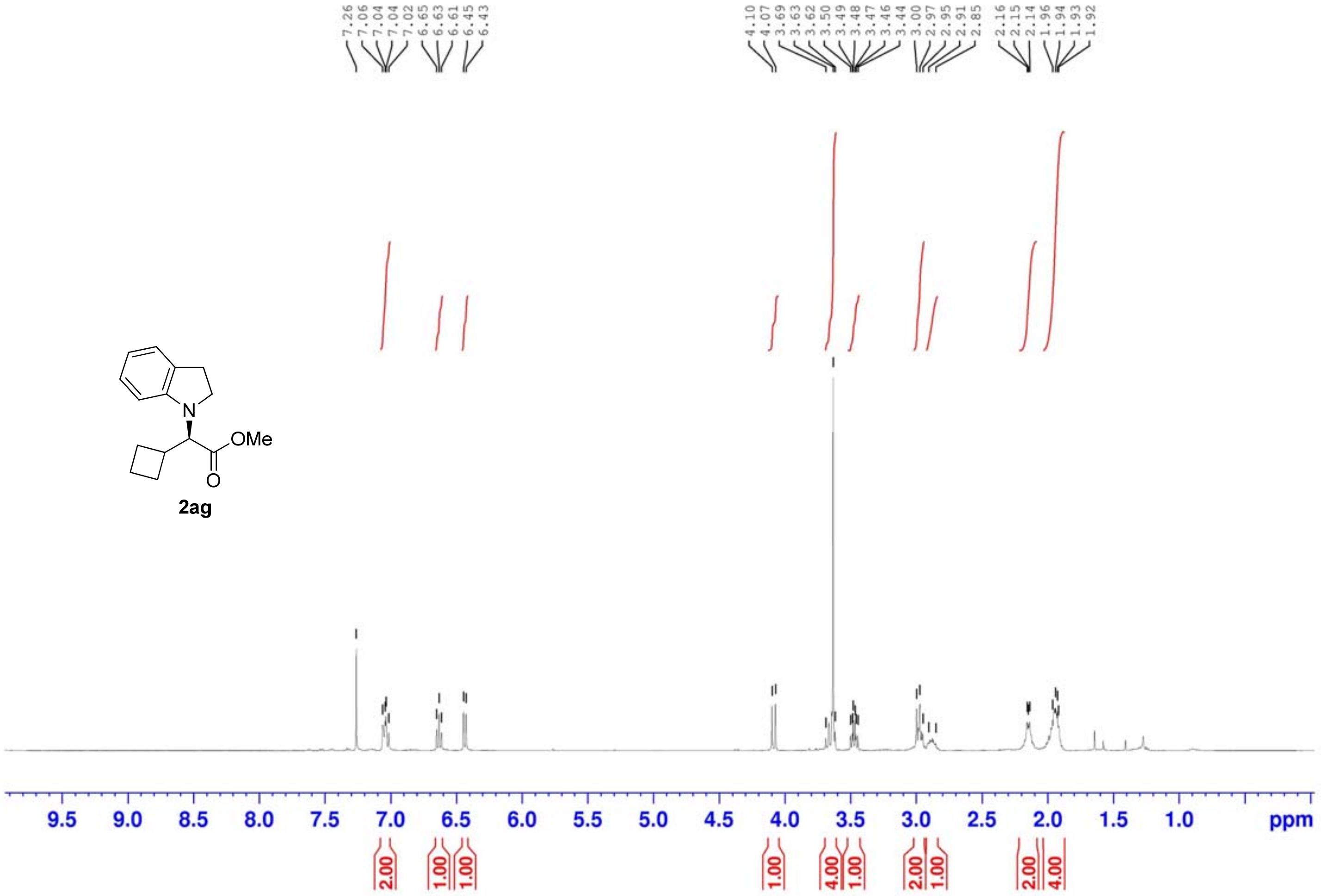

^{13}C NMR $CDCl_3$ (100 MHz)

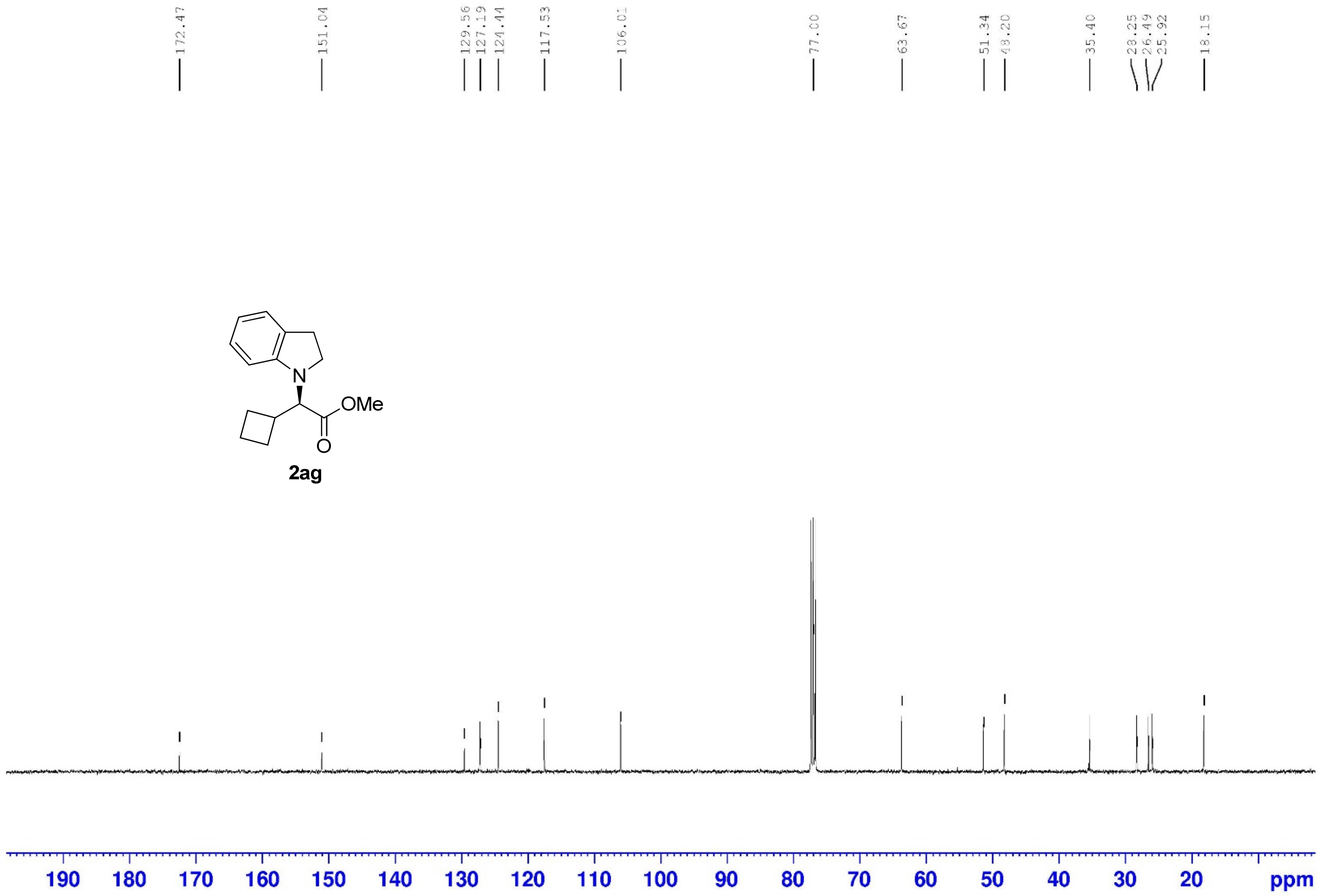

2ag

^{1}H NMR $CDCl_3$ (600 MHz)

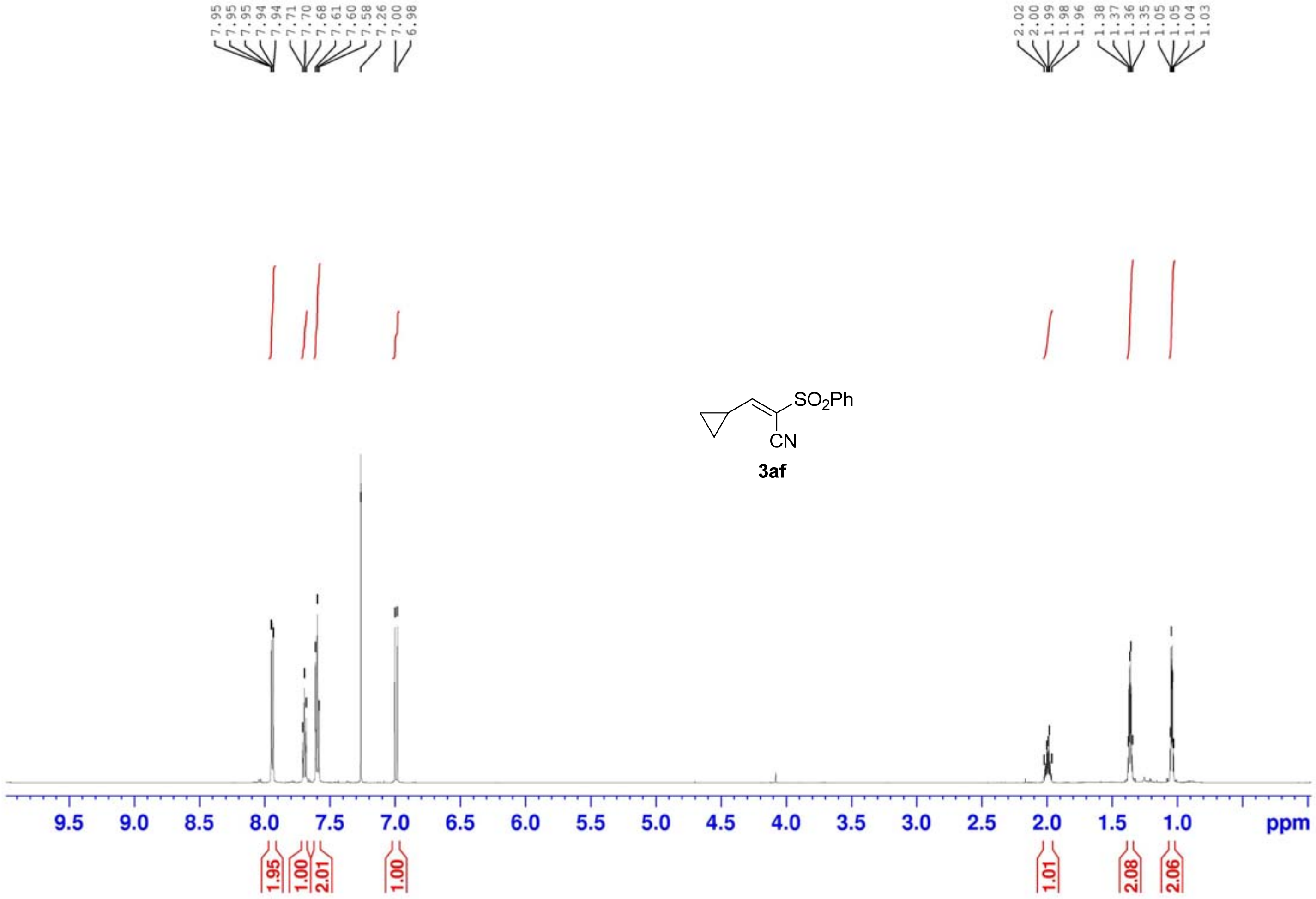

^{13}C NMR $CDCl_3$ (150 MHz)

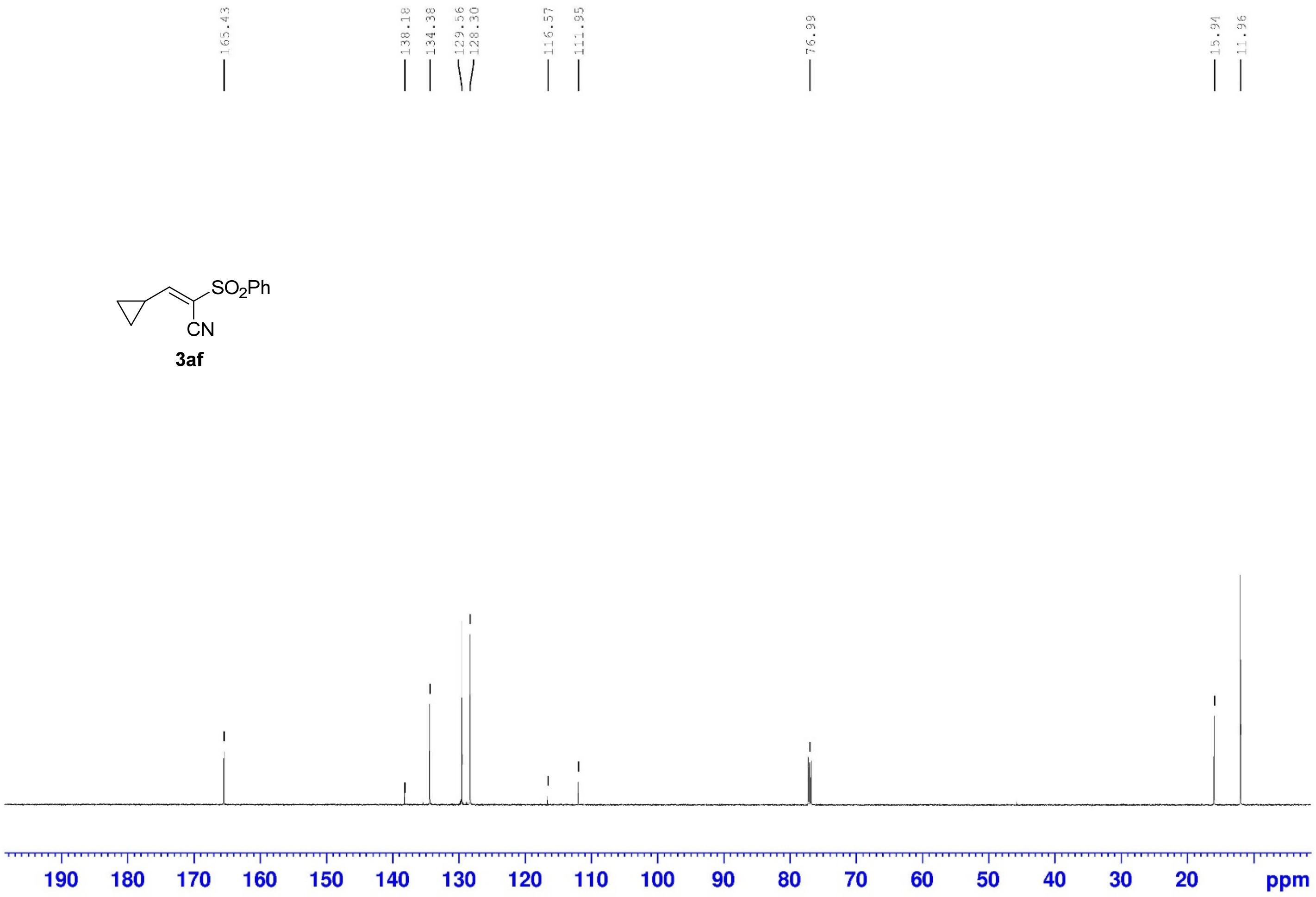

3af

^{1}H NMR $CDCl_3$ (400 MHz)

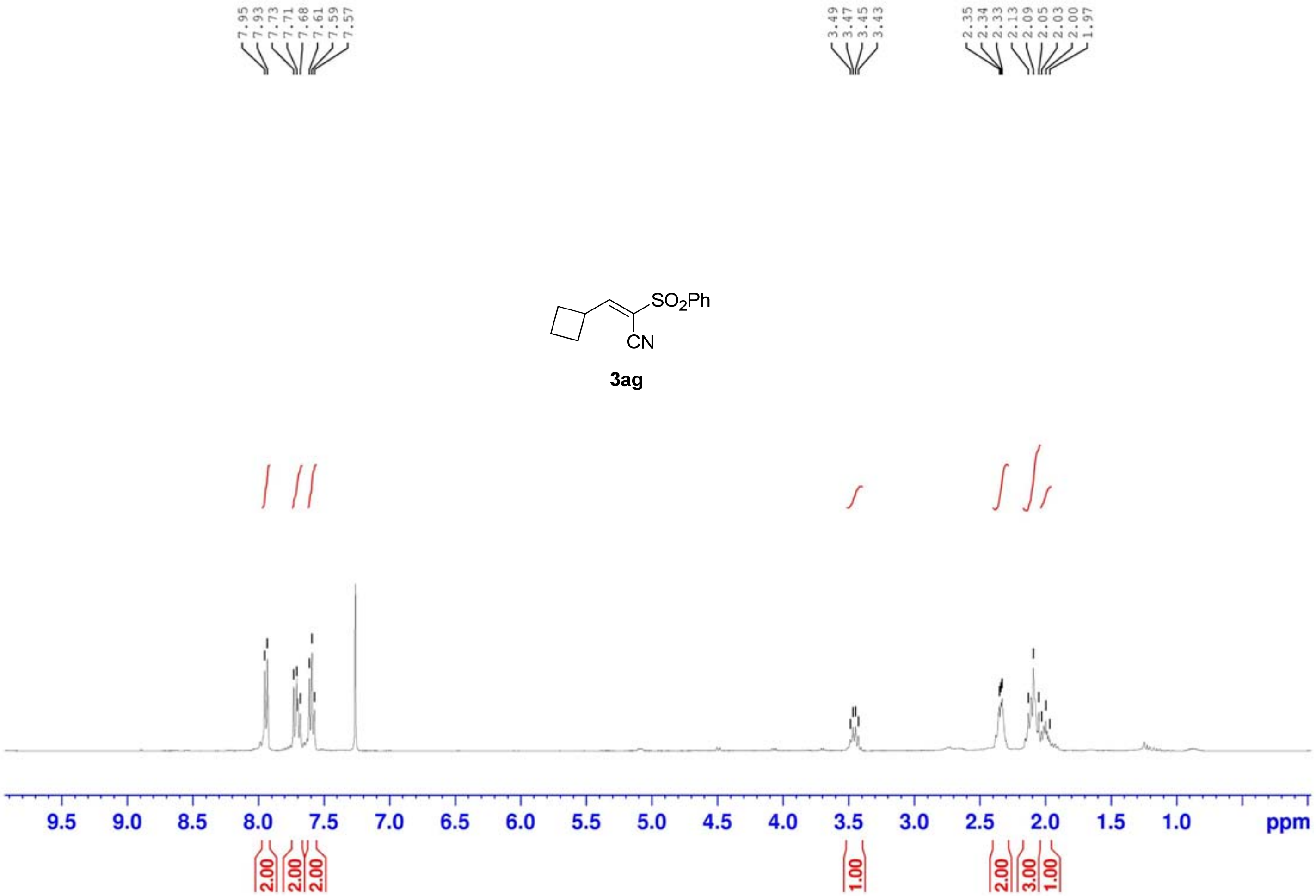

^{13}C NMR $CDCl_3$ (100 MHz)

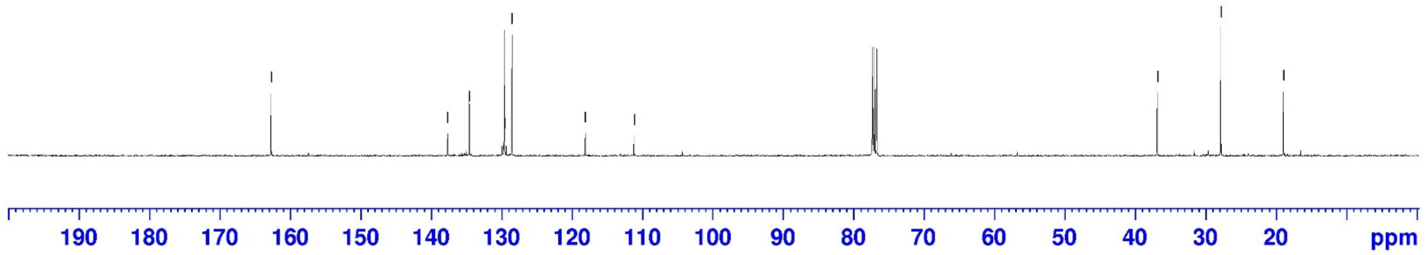

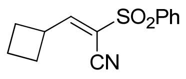

3ag

^1H NMR $CDCl_3$ (600 MHz)

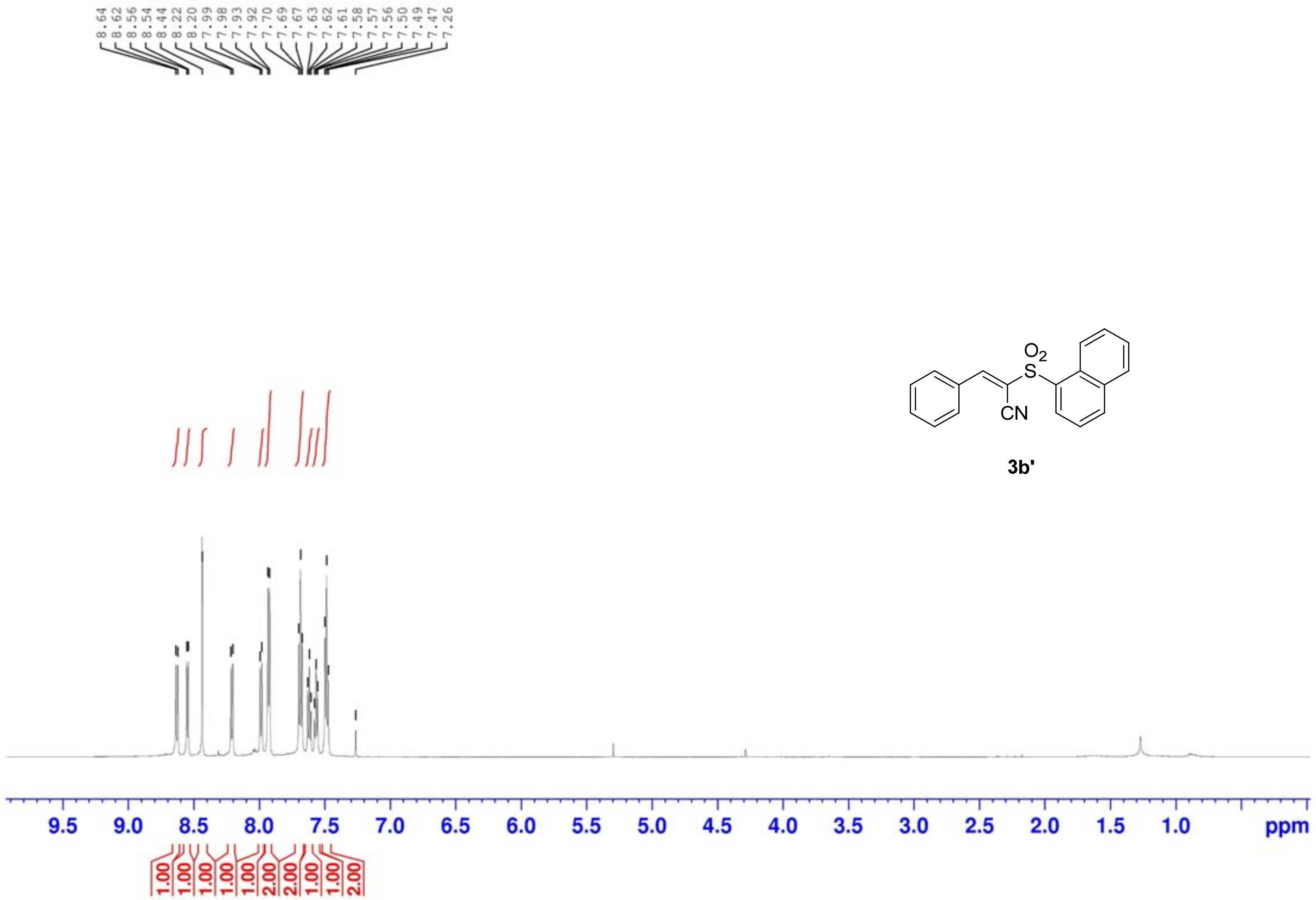

3b'

^{13}C NMR $CDCl_3$ (150 MHz)

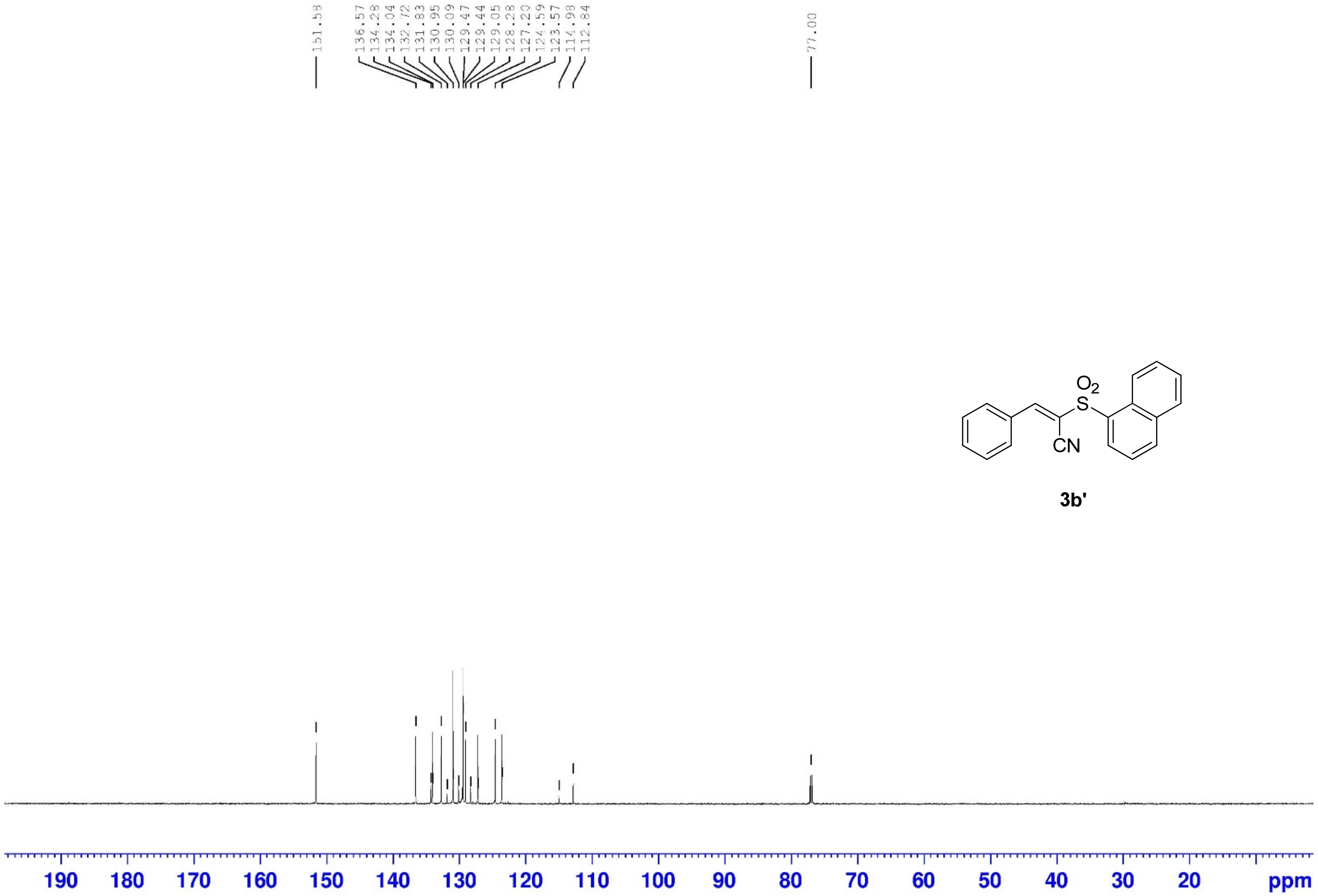

3b'

^{1}H NMR $CDCl_3$ (400 MHz)

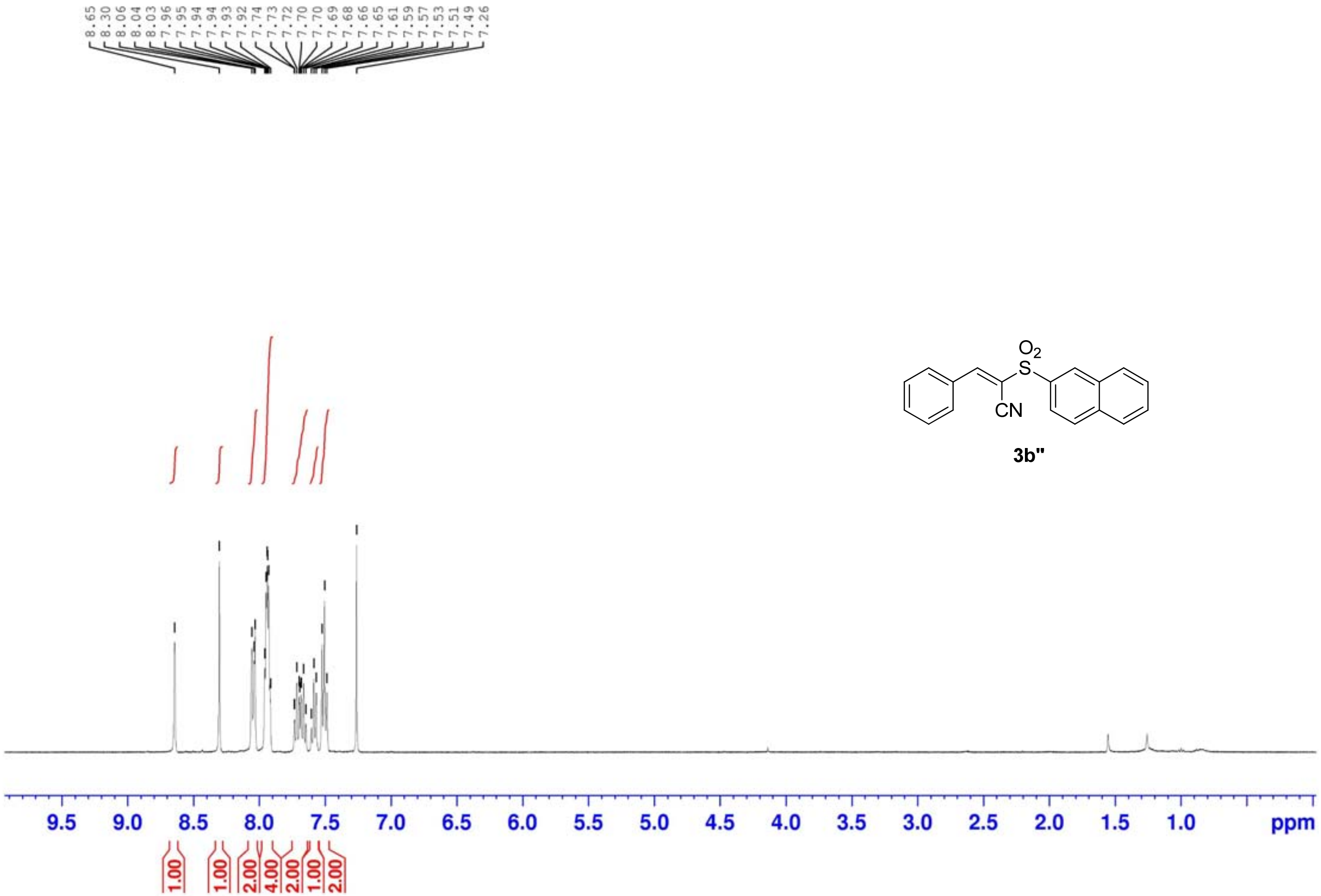

3b''

^{13}C NMR $CDCl_3$ (100 MHz)

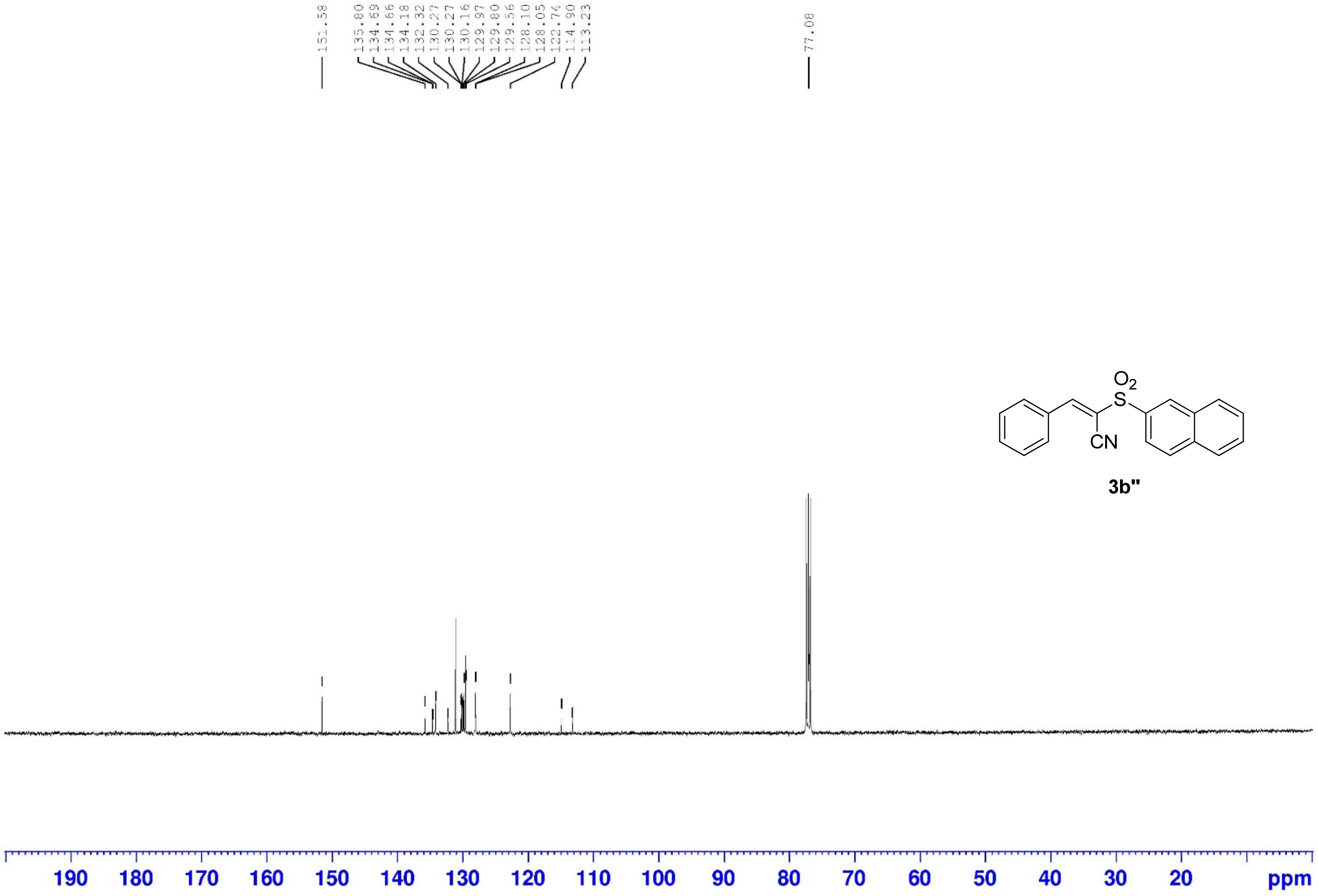

3b''

^{1}H NMR $CDCl_3$ (300 MHz)

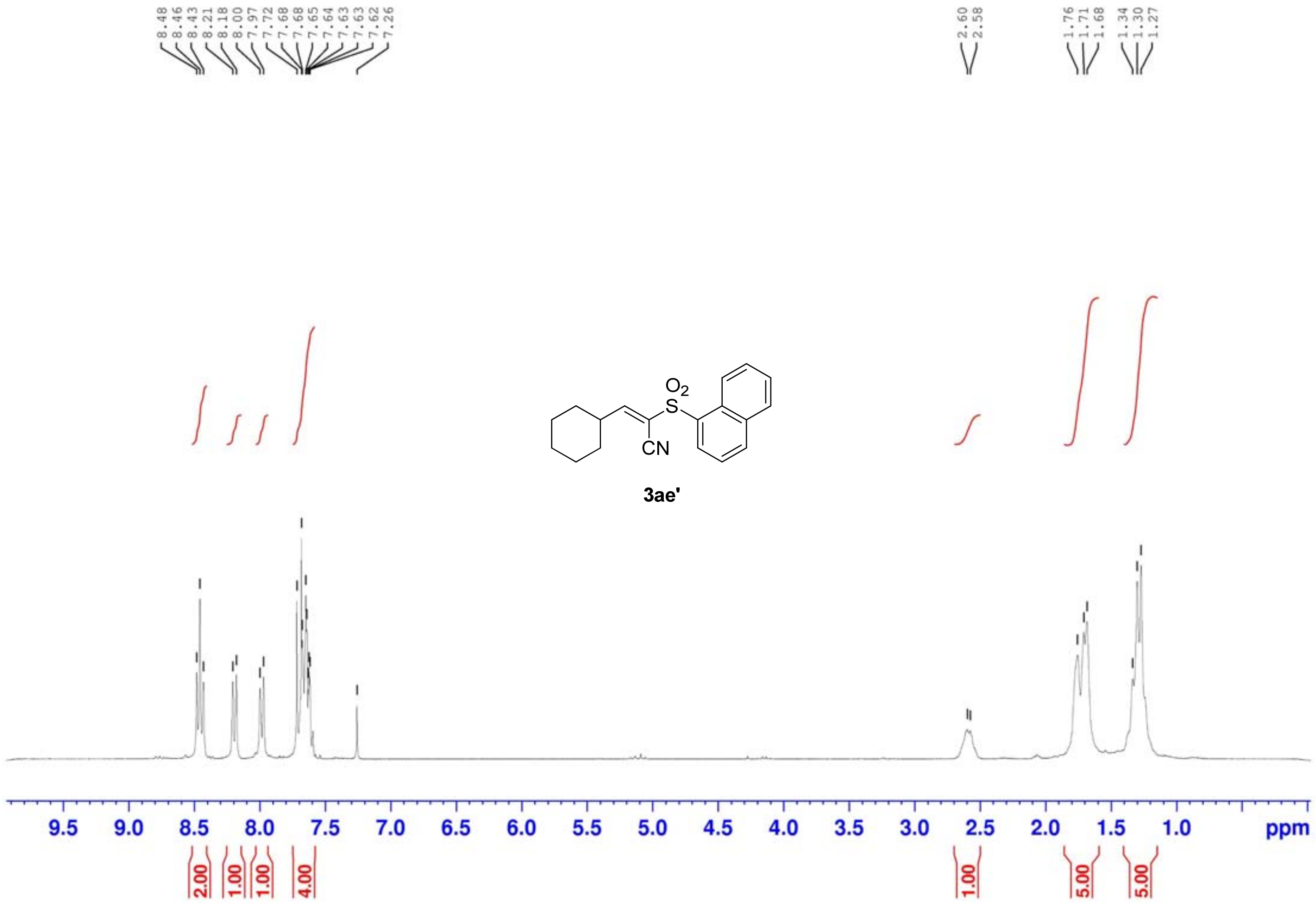

^{13}C NMR $CDCl_3$ (75 MHz)

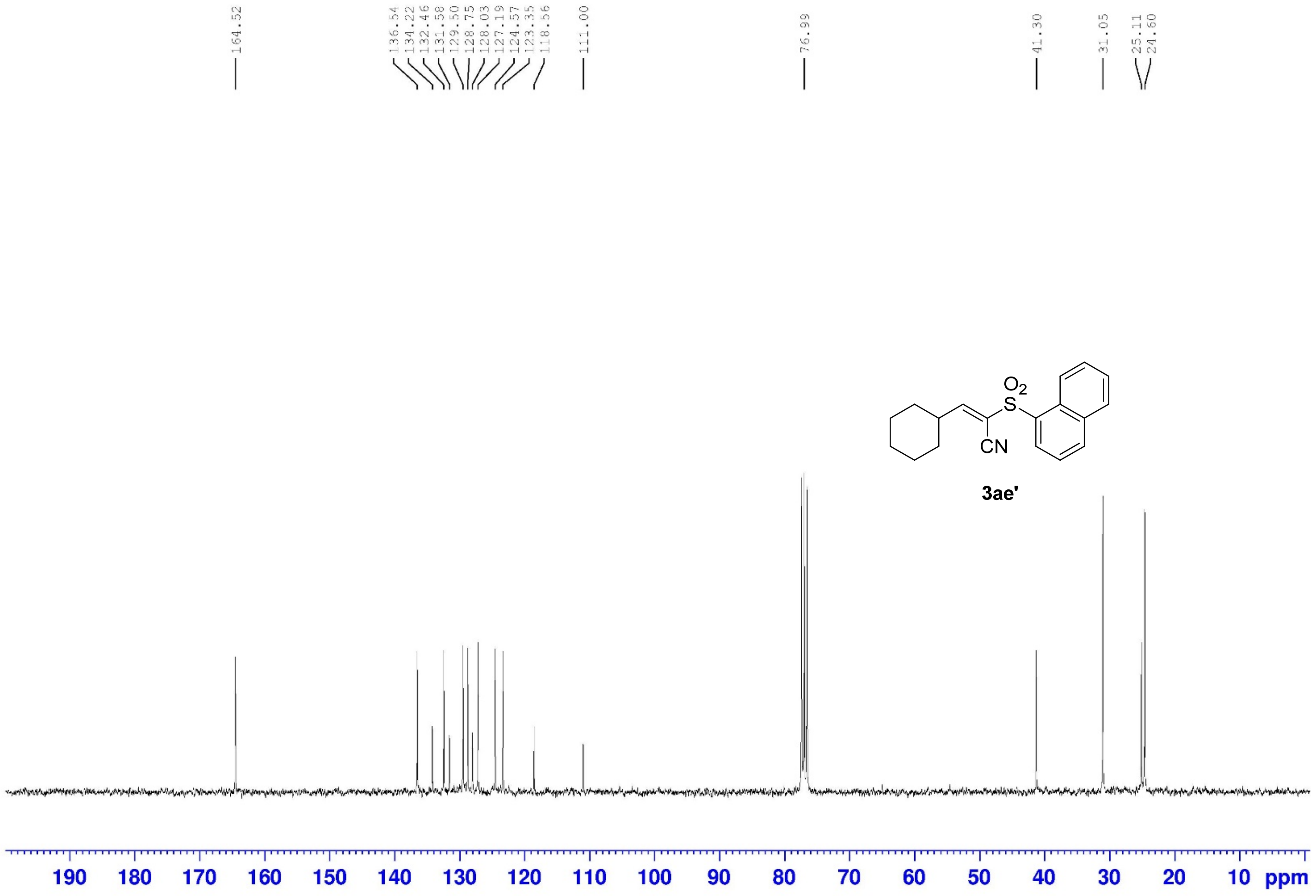

^{1}H NMR $CDCl_3$ (400 MHz)

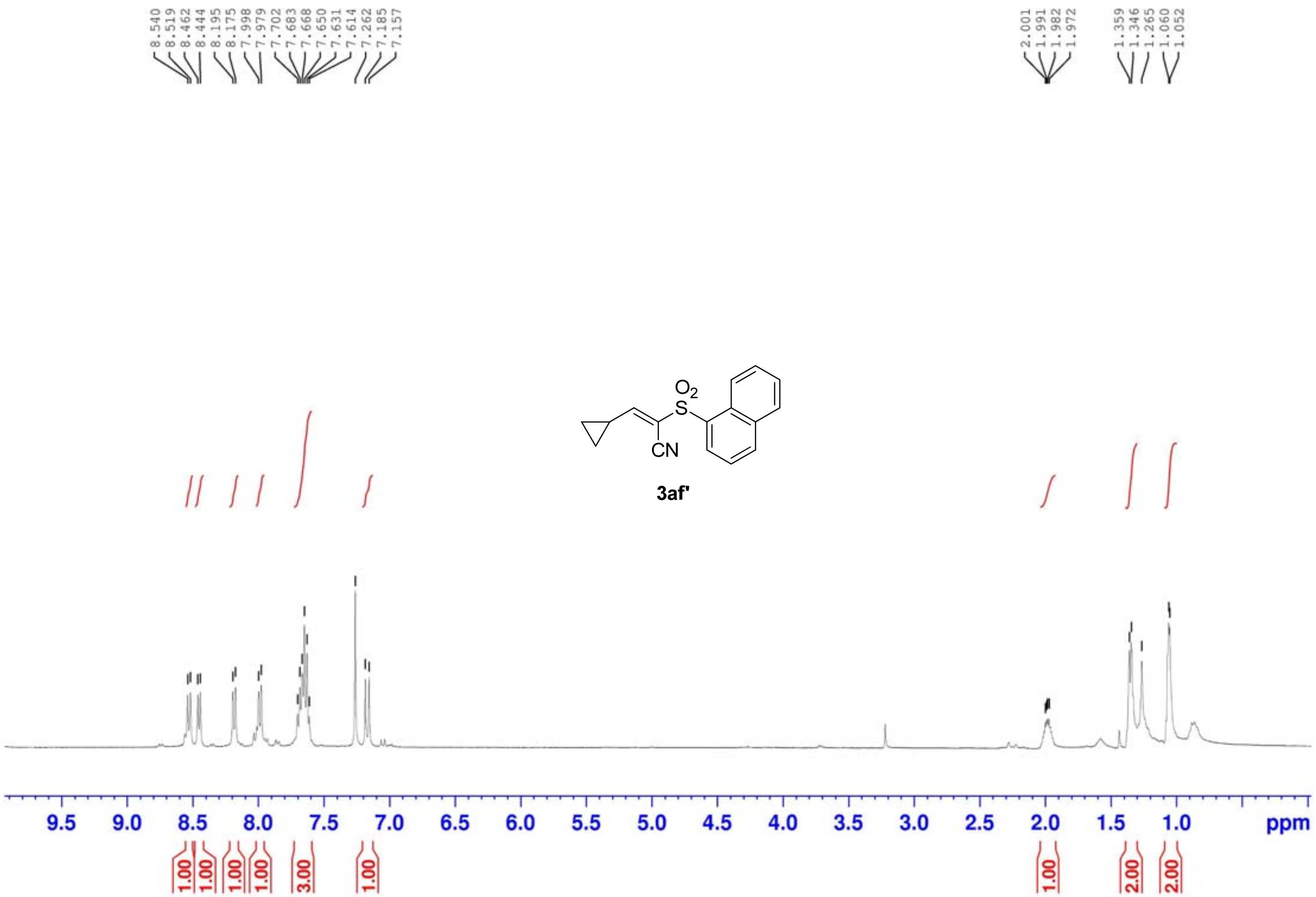

3af'

^{13}C NMR $CDCl_3$ (100 MHz)

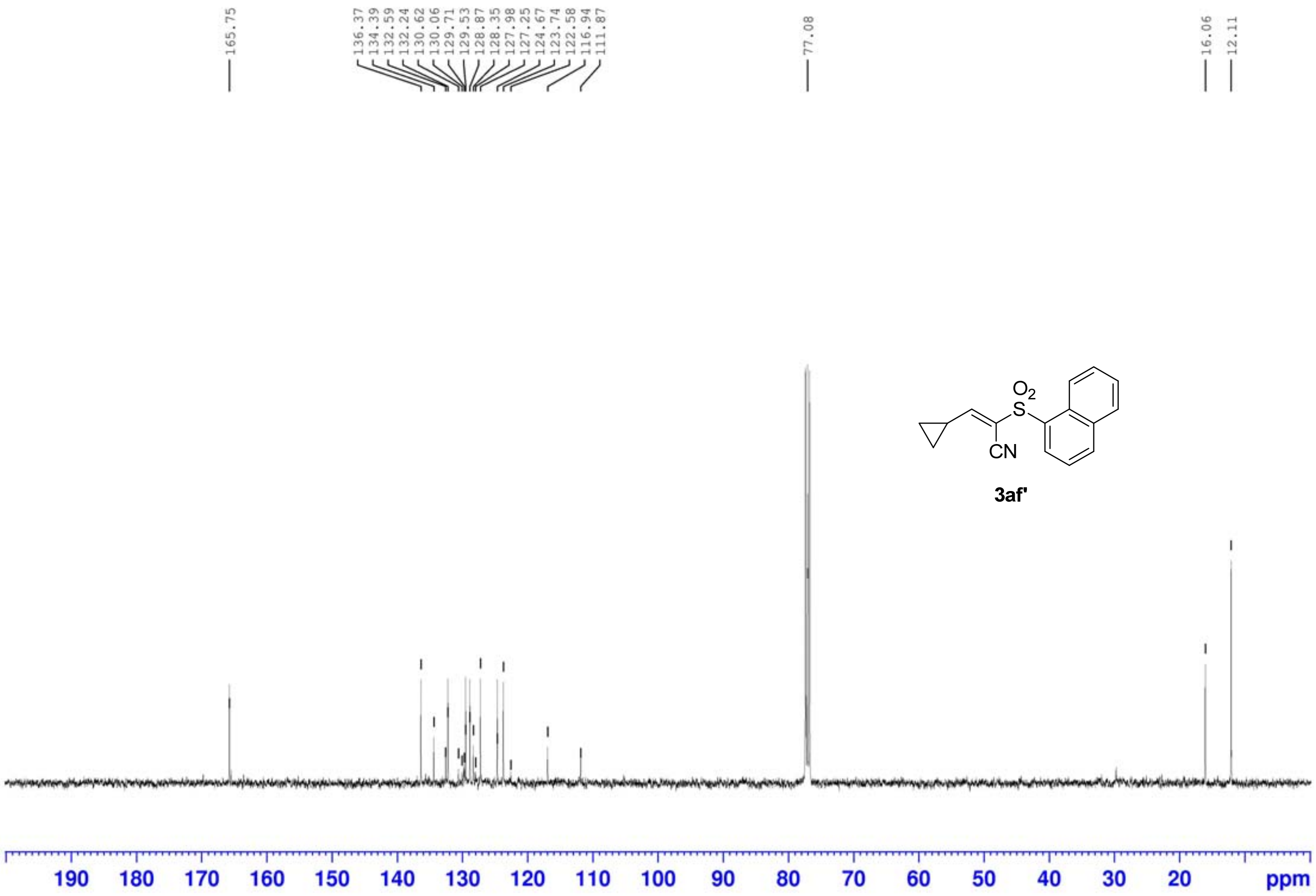

^{1}H NMR $CDCl_3$ (600 MHz)

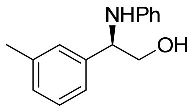

5a

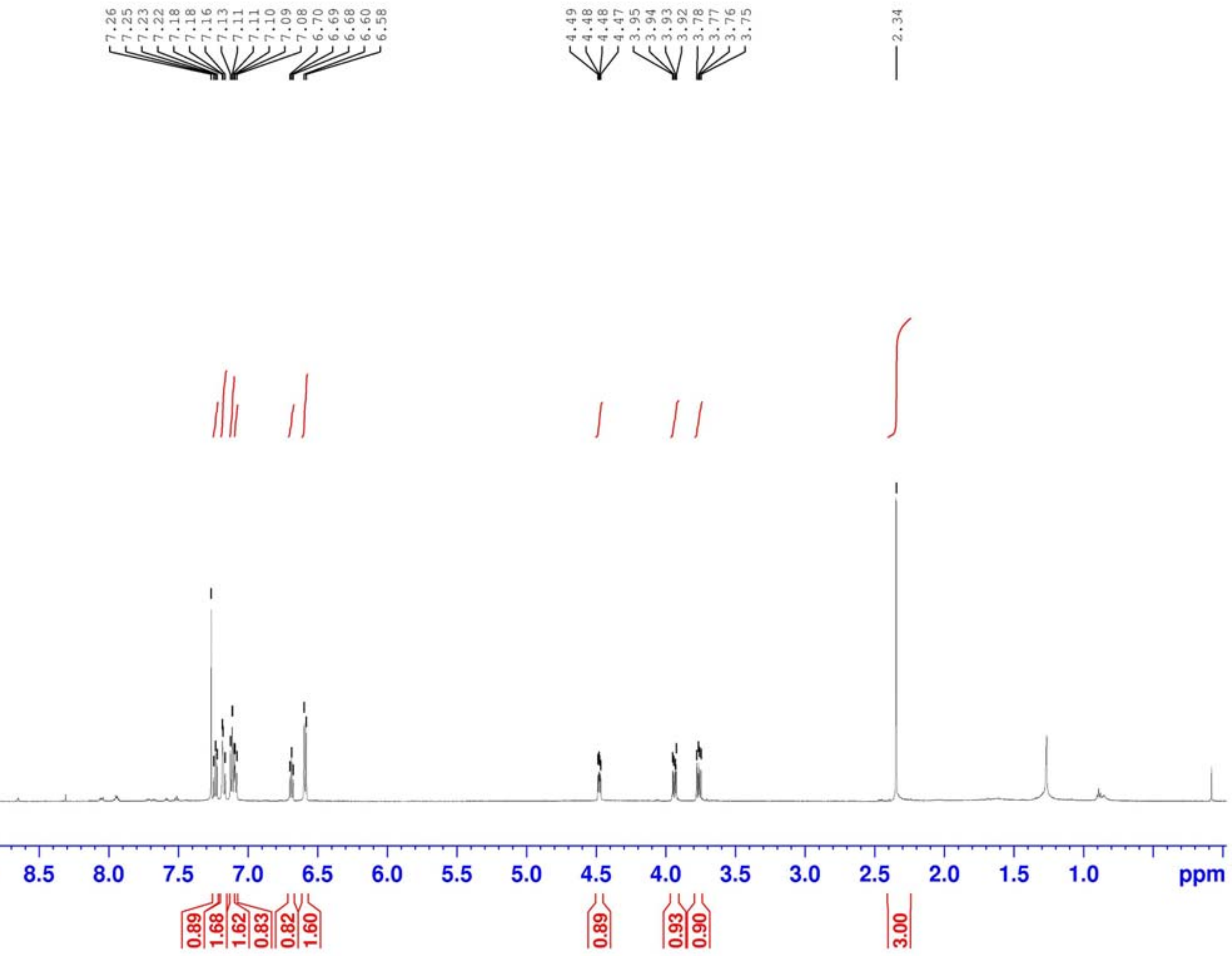

^{13}C NMR $CDCl_3$ (150 MHz)

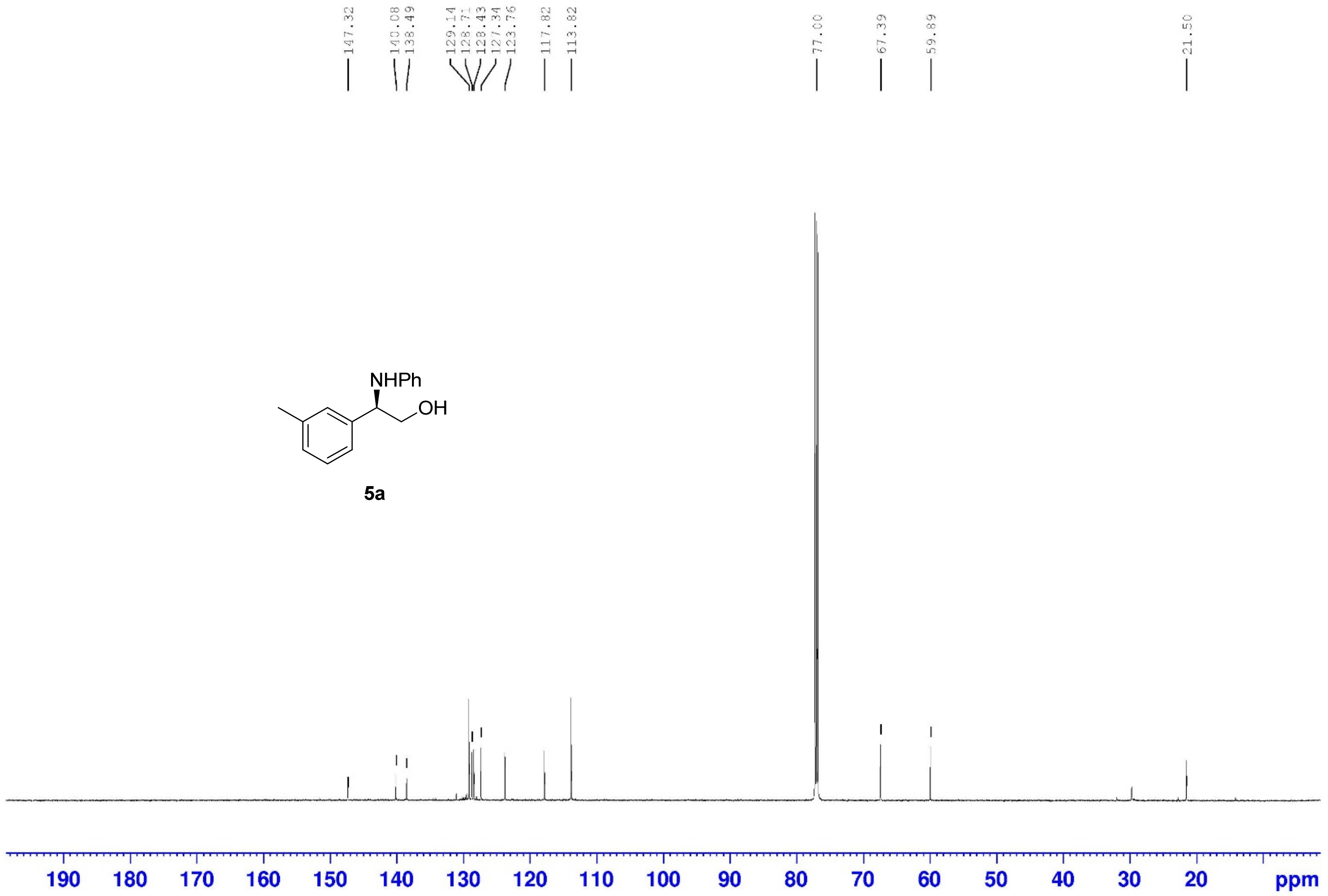

^{1}H NMR $CDCl_3$ (600 MHz)

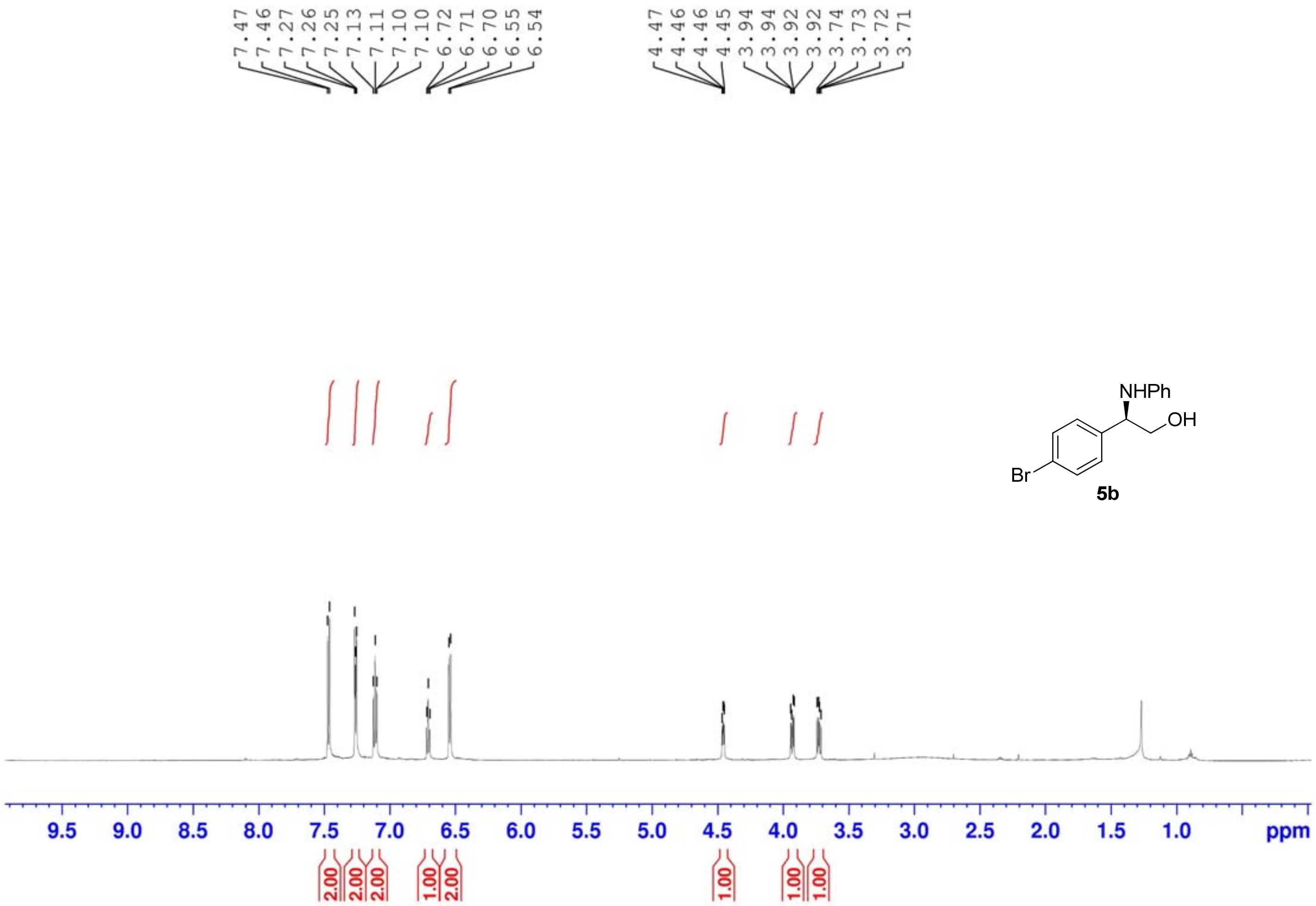

^{13}C NMR $CDCl_3$ (150 MHz)

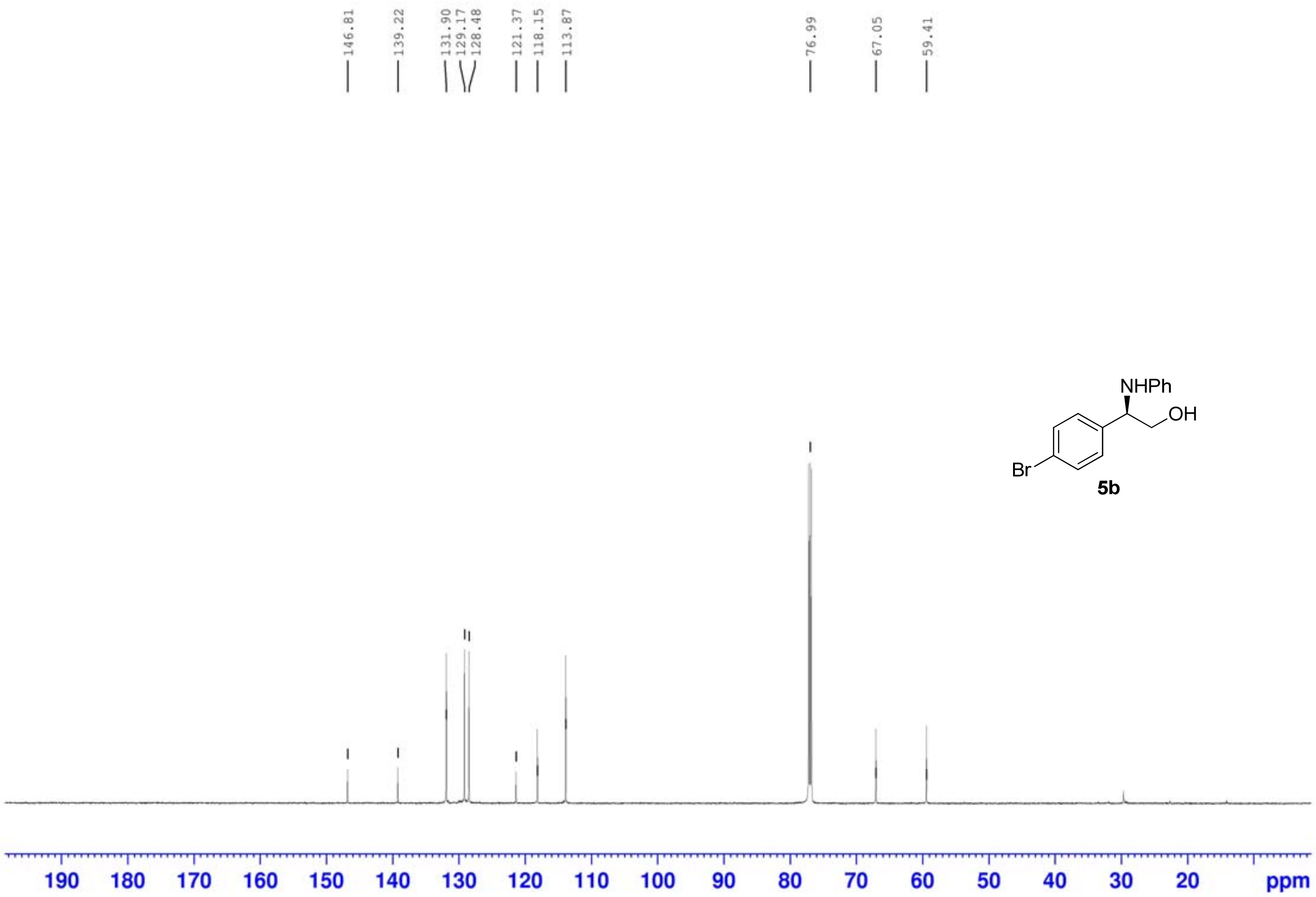

^{1}H NMR $CDCl_3$ (600 MHz)

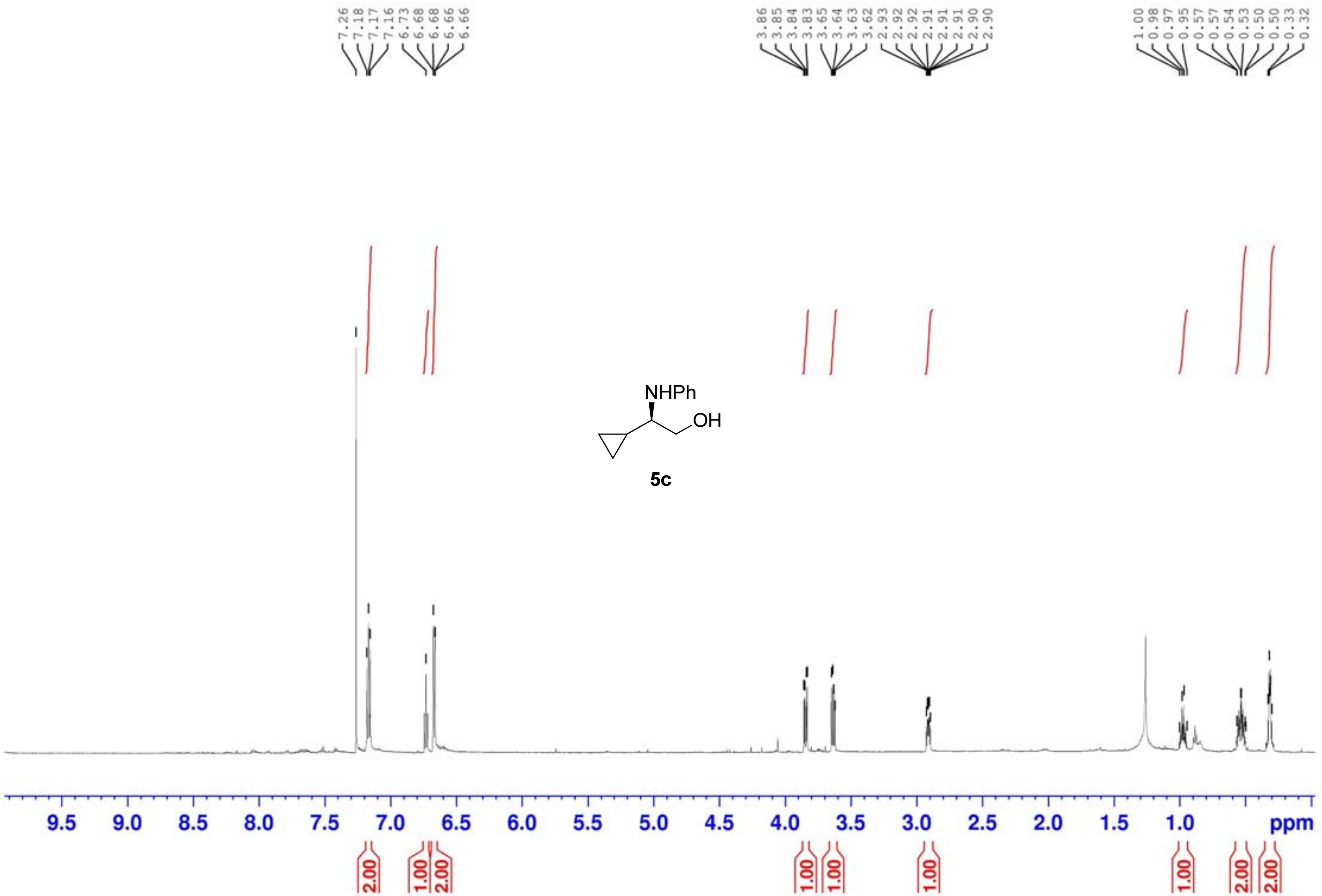

^{13}C NMR $CDCl_3$ (150 MHz)

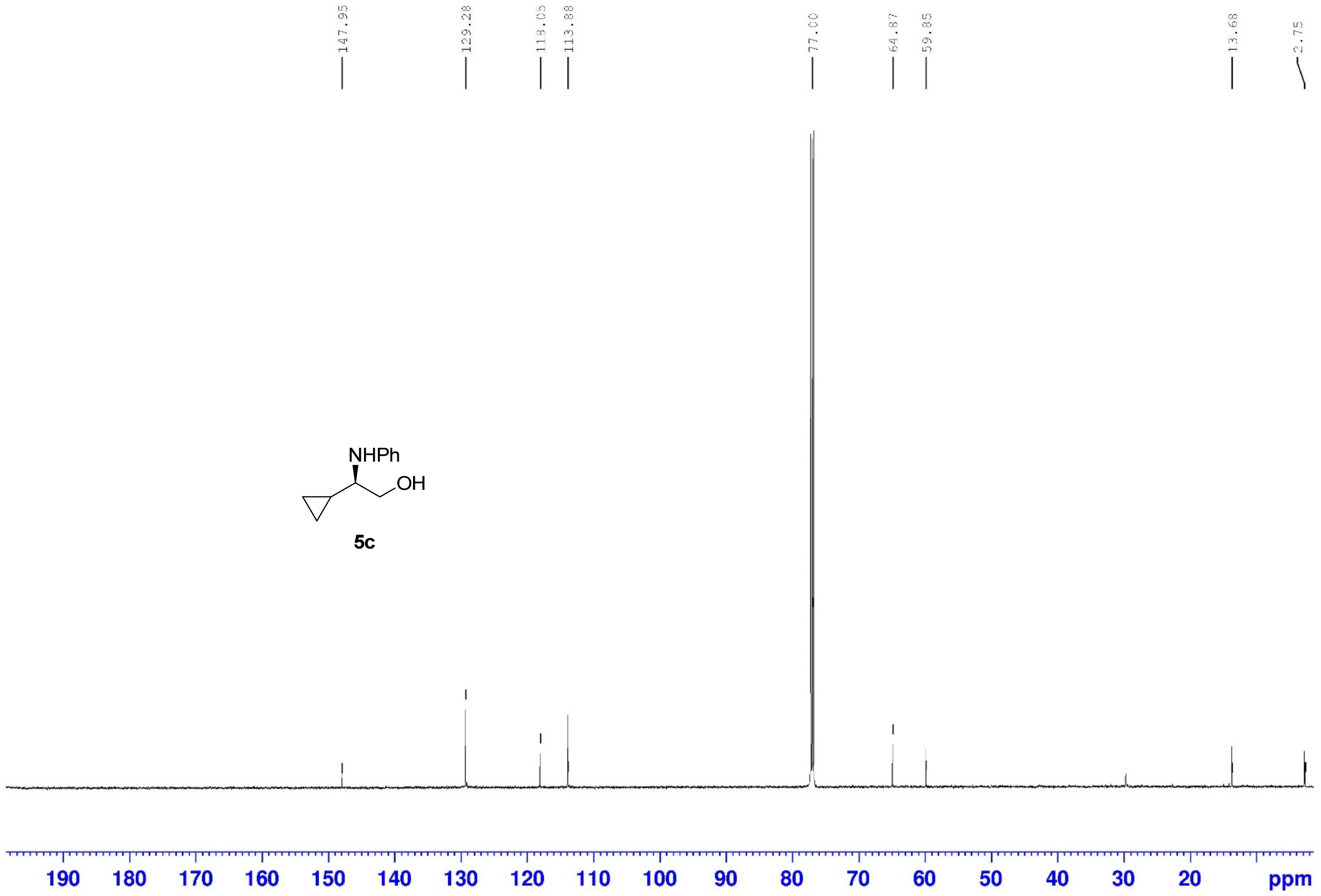

^{1}H NMR $CDCl_3$ (600 MHz)

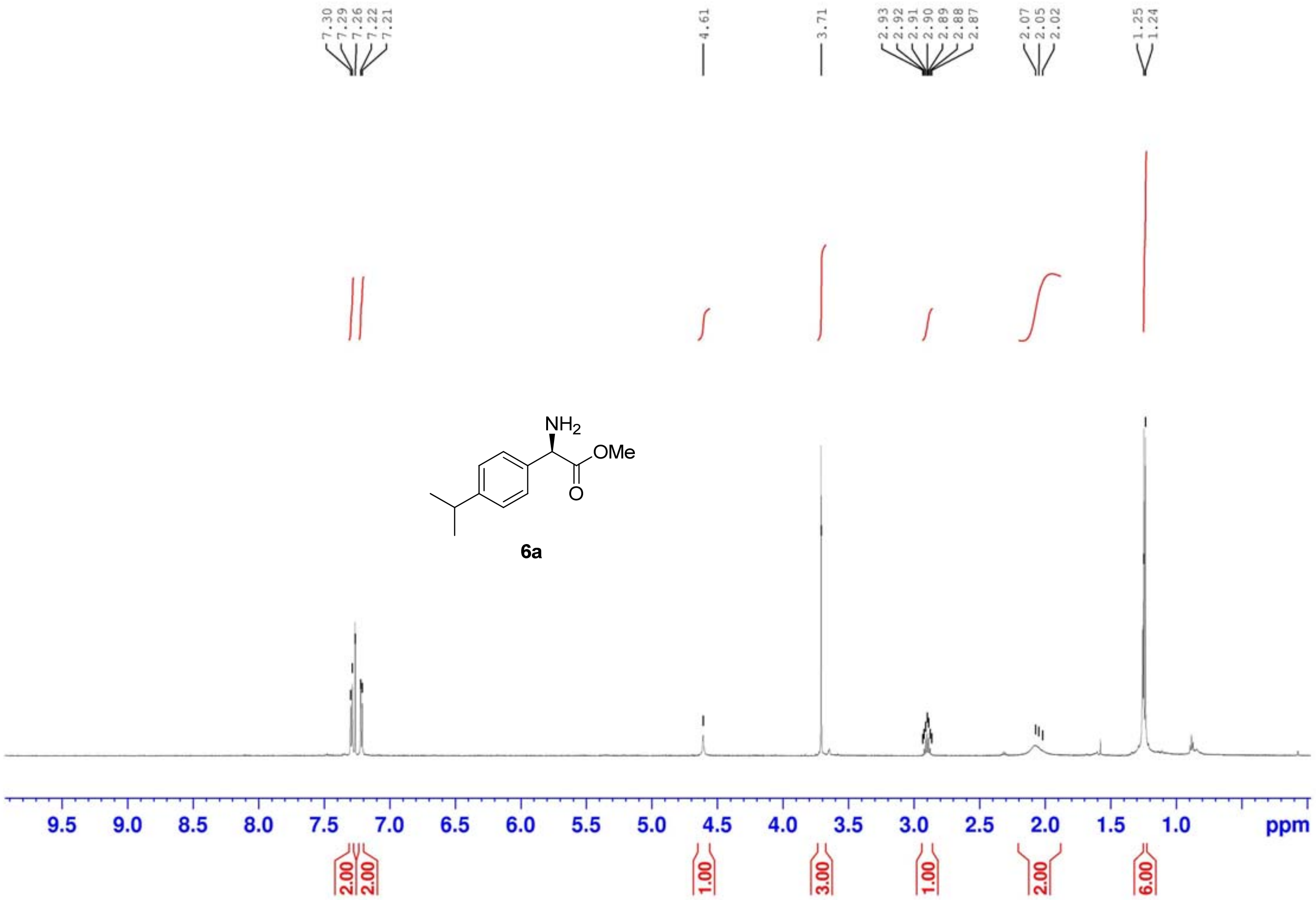

^{13}C NMR $CDCl_3$ (150 MHz)

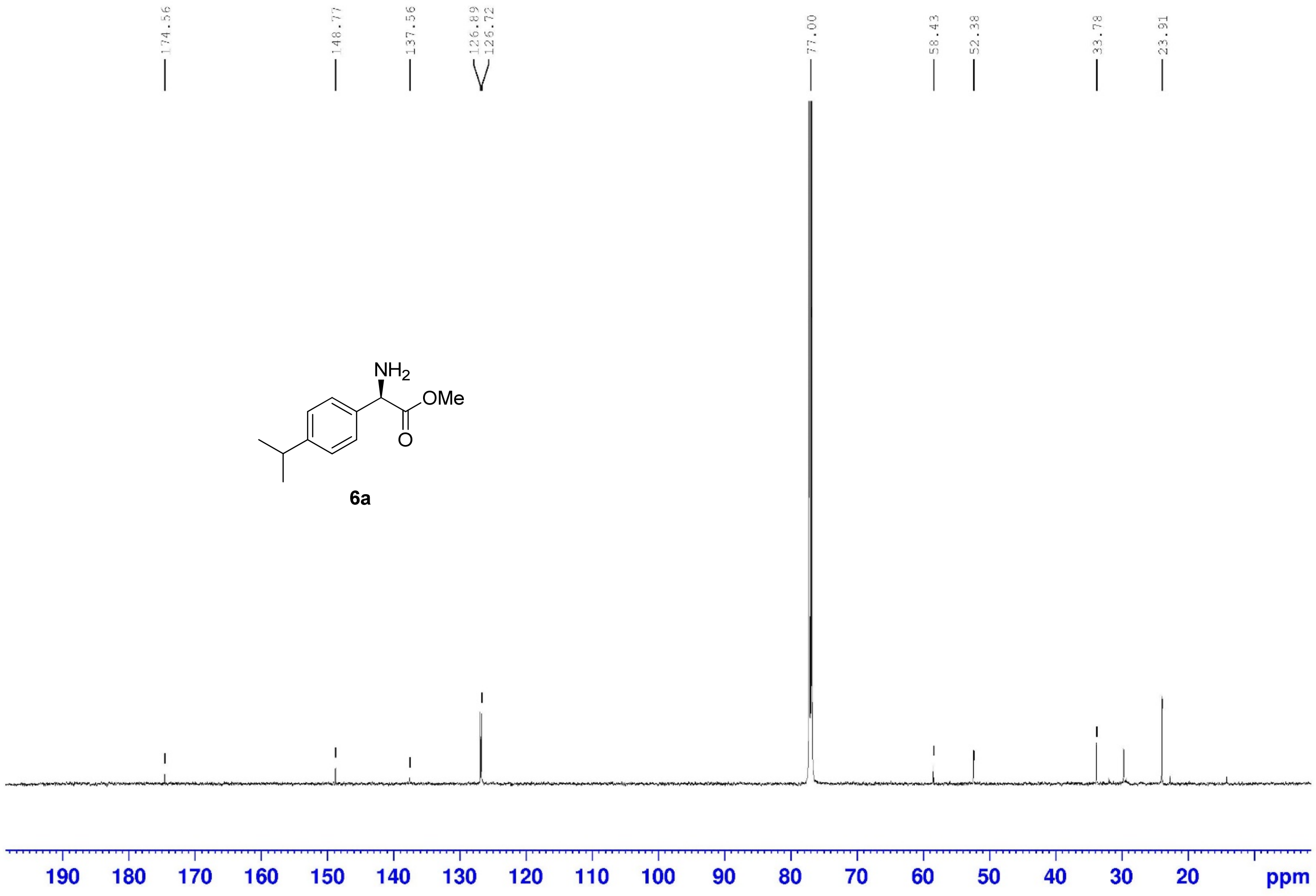

HPLC Chromatograms

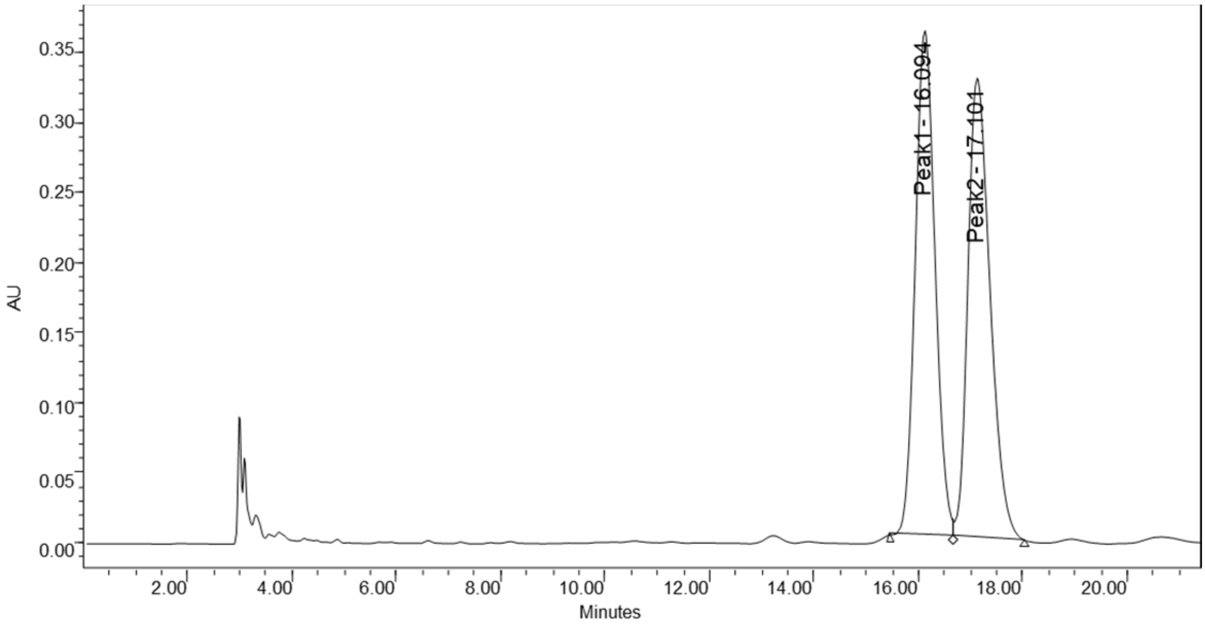

	Peak Name	RT (min)	Area (V*sec)	% Area	Height (V)	% Height
1	Peak1	16.094	9540226	49.57	360743	52.35
2	Peak2	17.101	9707587	50.43	328386	47.65

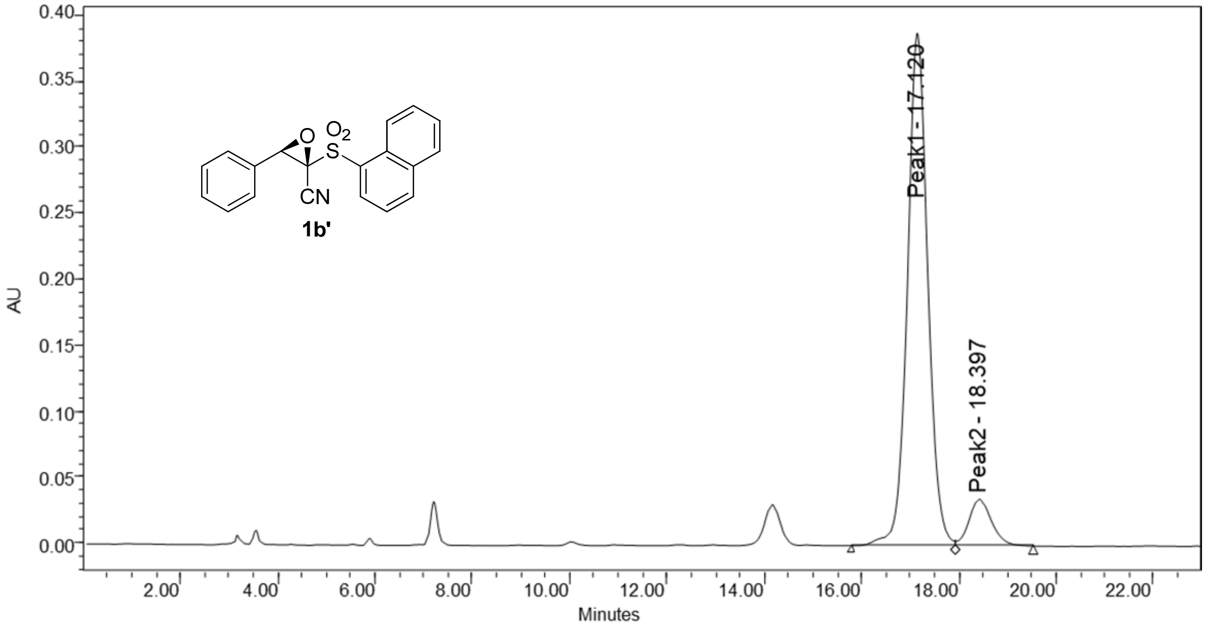

	Peak Name	RT (min)	Area (V*sec)	% Area	Height (V)	% Height
1	Peak1	17.120	11336800	90.73	388628	91.62
2	Peak2	18.397	1158707	9.27	35545	8.38

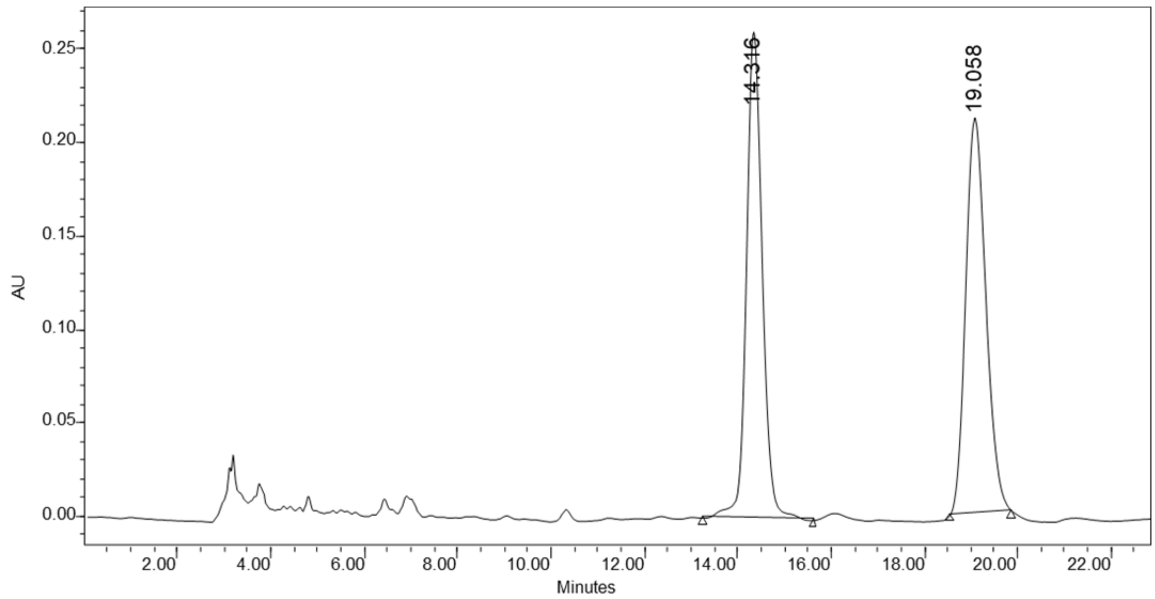

	RT (min)	Area (V*sec)	% Area	Height (V)	% Height
1	14.316	6190368	49.30	260470	55.17
2	19.058	6366710	50.70	211685	44.83

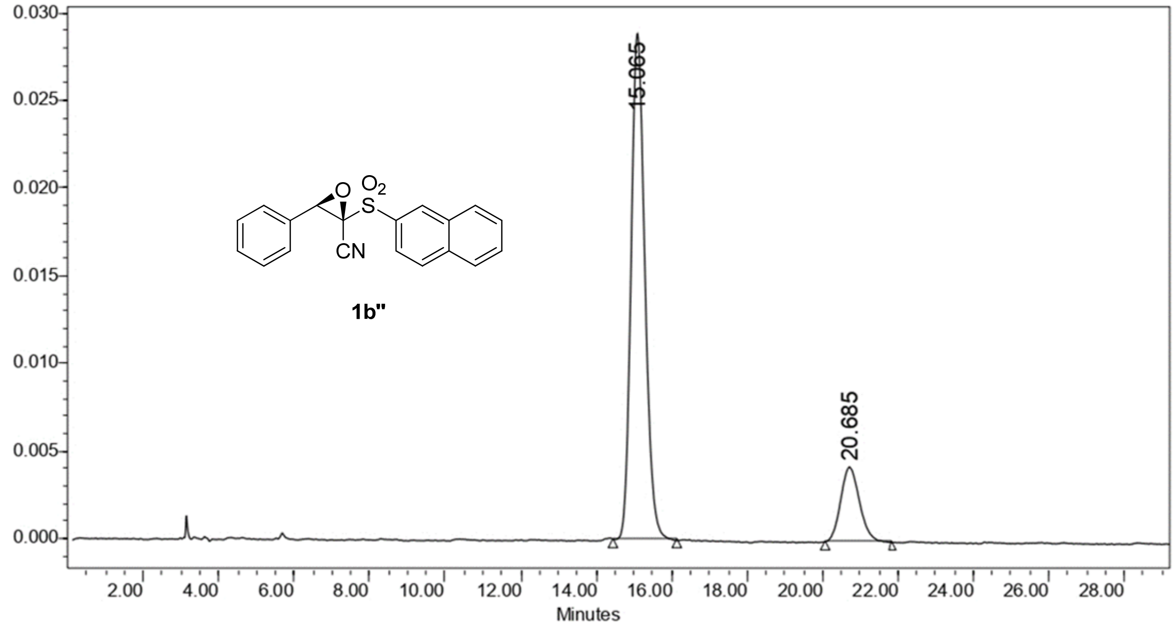

	RT (min)	Area (□V*sec)	% Area	Height (□V)	% Height
1	15.065	735014	83.15	28886	87.10
2	20.685	148908	16.85	4279	12.90

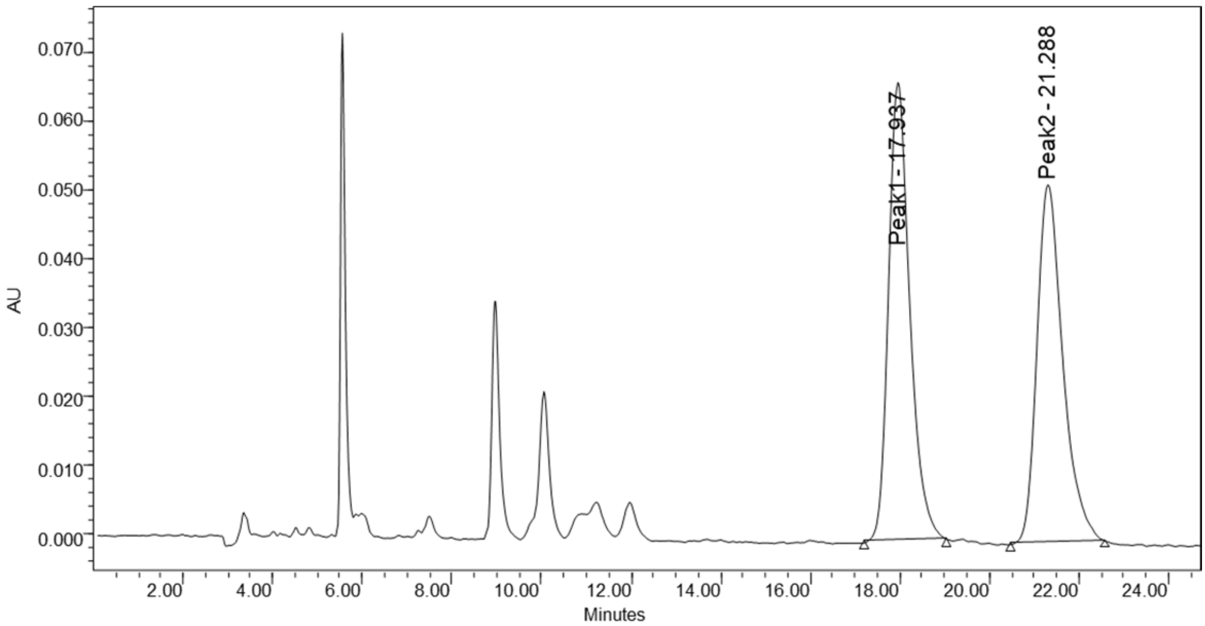

	Peak Name	RT (min)	Area (V*sec)	% Area	Height (V)	% Height
1	Peak1	17.937	2200510	51.21	66437	56.15
2	Peak2	21.288	2096134	48.79	51882	43.85

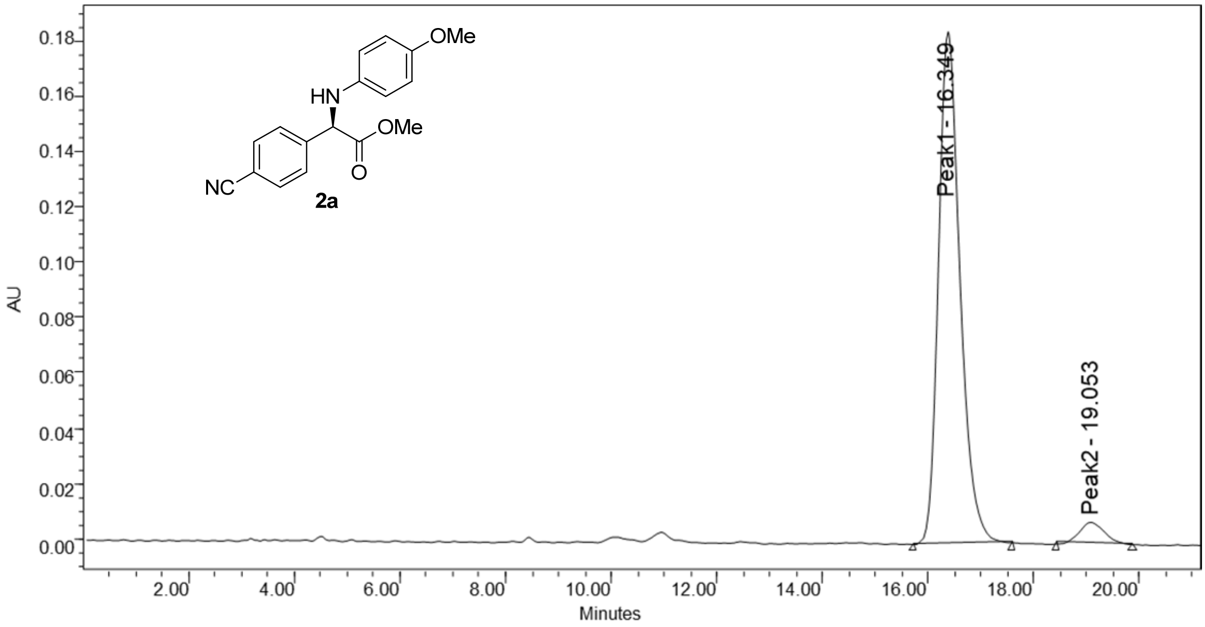

	Peak Name	RT (min)	Area (V*sec)	% Area	Height (V)	% Height
1	Peak1	16.349	5258724	95.19	184878	95.91
2	Peak2	19.053	265565	4.81	7877	4.09

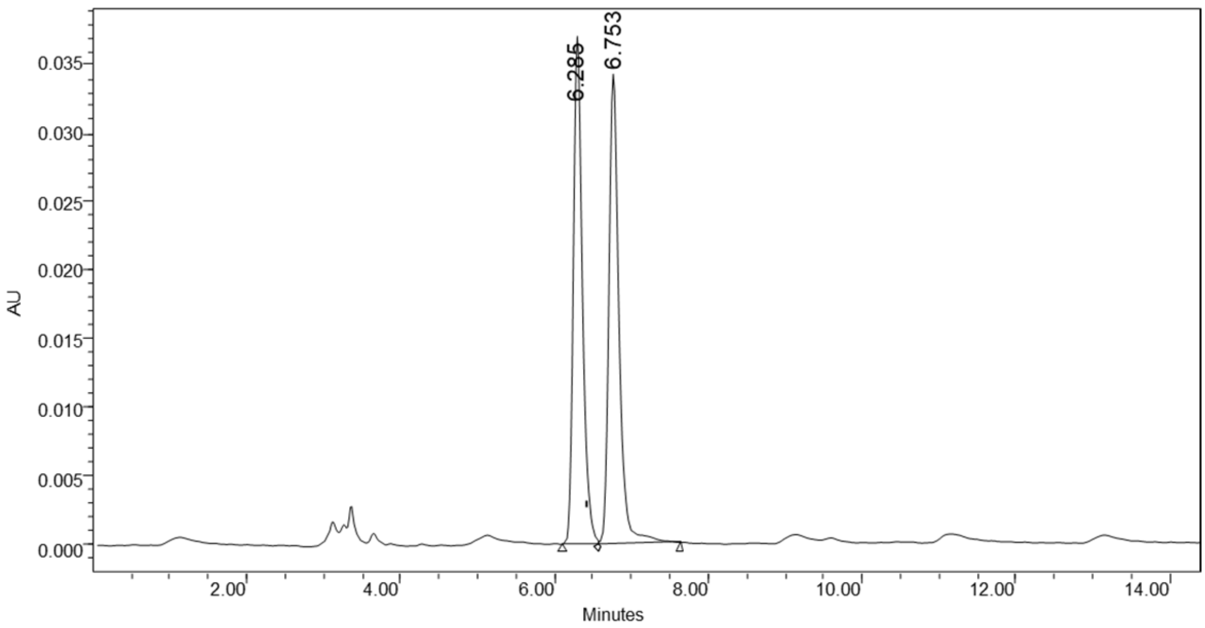

	RT (min)	Area (V*sec)	% Area	Height (V)	% Height
1	6.285	302074	49.18	37014	51.94
2	6.753	312201	50.82	34243	48.06

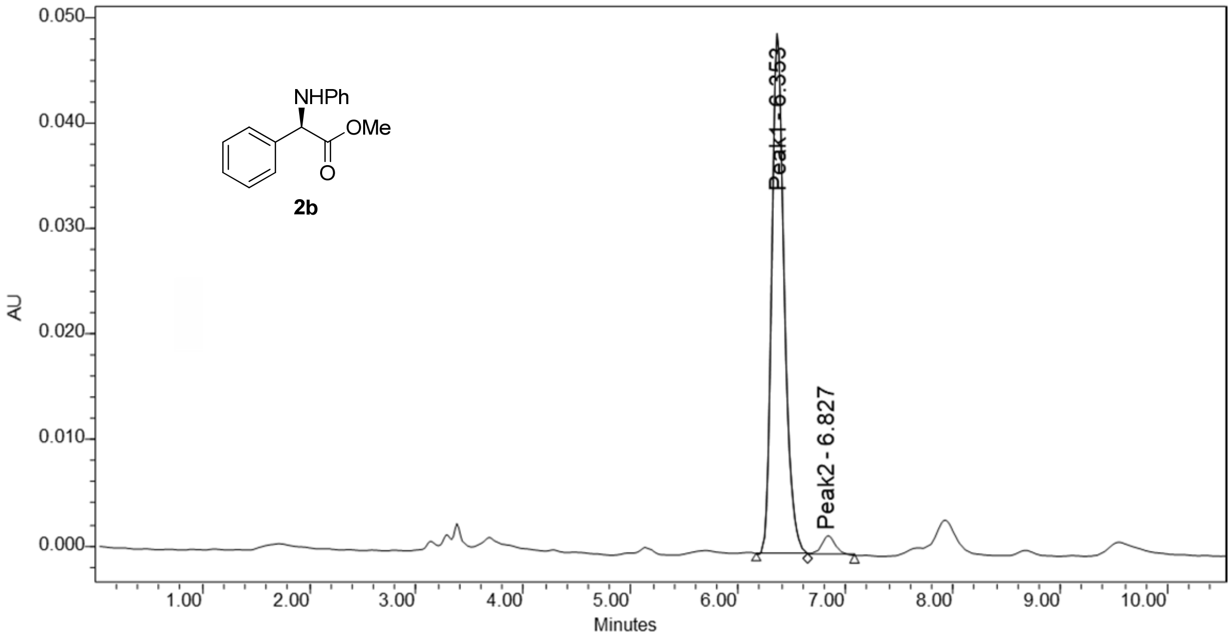

	Peak Name	RT (min)	Area (V*sec)	% Area	Height (V)	% Height
1	Peak1	6.353	400950	96.03	49245	96.42
2	Peak2	6.827	16563	3.97	1830	3.58

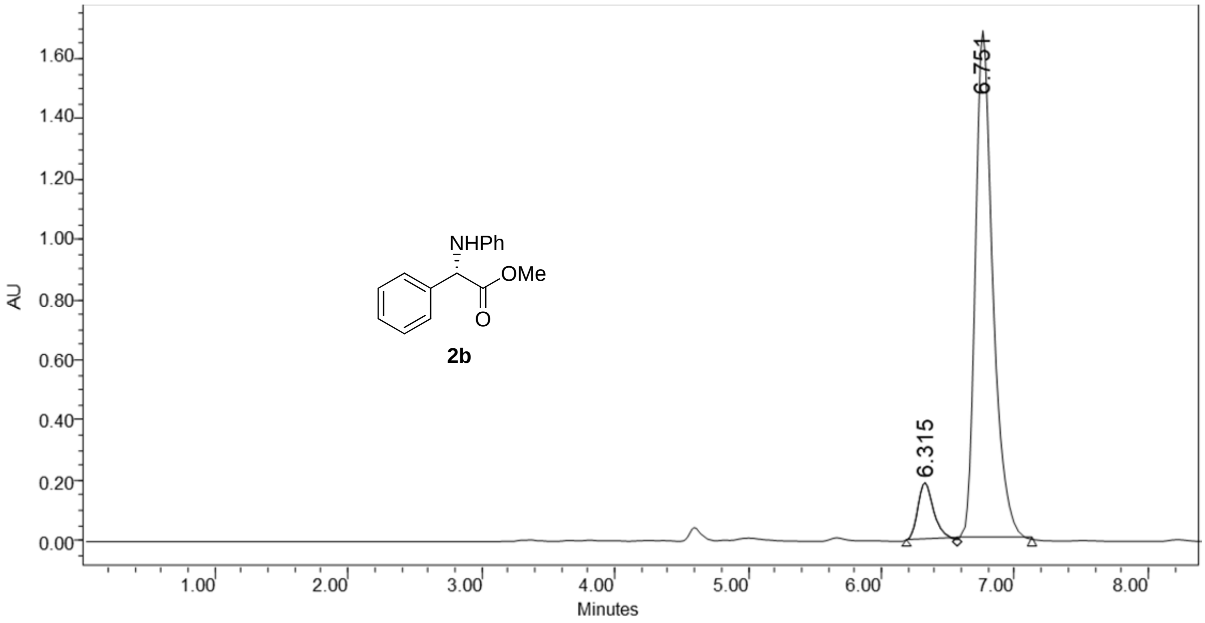

	RT (min)	Area (V*sec)	% Area	Height (V)	% Height
1	6.315	1572090	9.18	190485	10.15
2	6.751	15561262	90.82	1686993	89.85

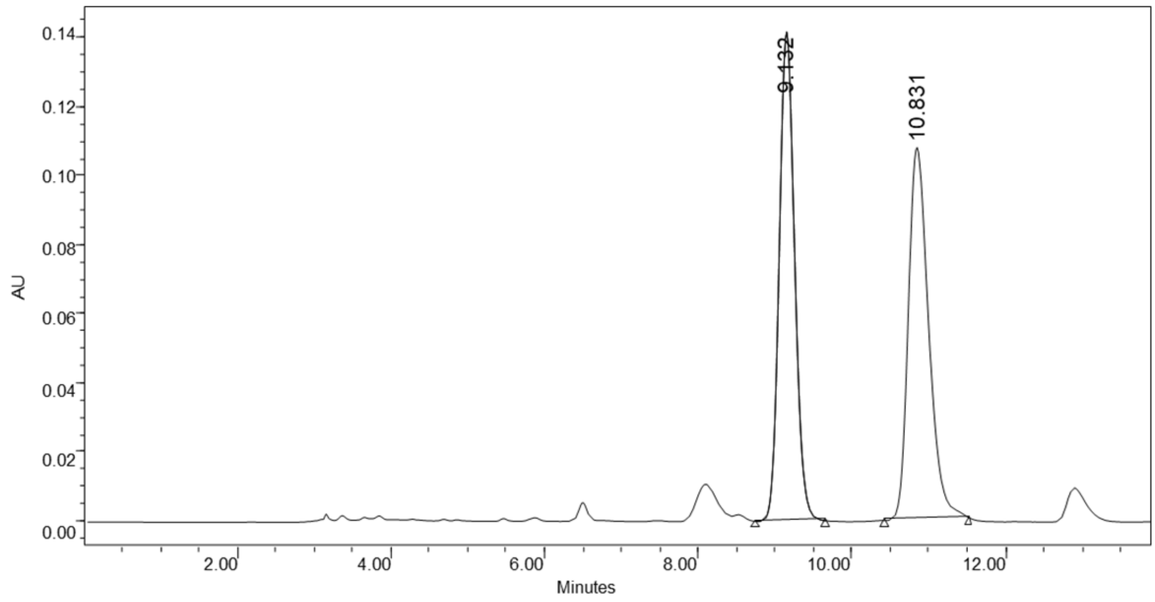

	RT (min)	Area (V*sec)	% Area	Height (V)	% Height
1	9.132	1976409	49.97	141627	56.79
2	10.831	1979105	50.03	107761	43.21

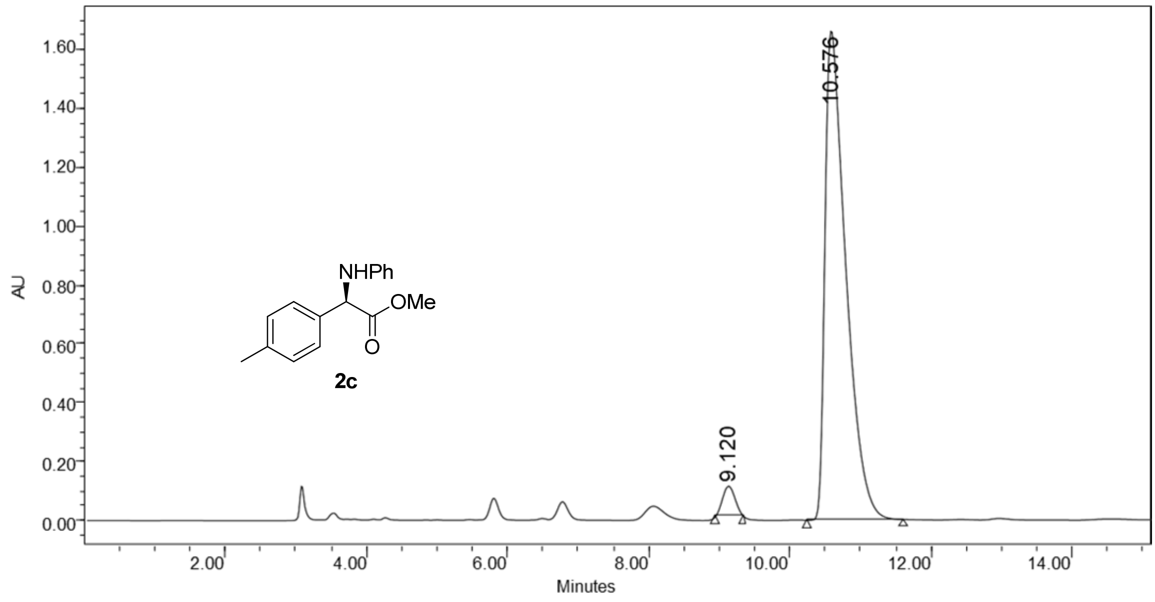

	RT (min)	Area (V*sec)	% Area	Height (V)	% Height
1	9.120	1278831	3.60	103442	5.84
2	10.576	34201052	96.40	1668463	94.16

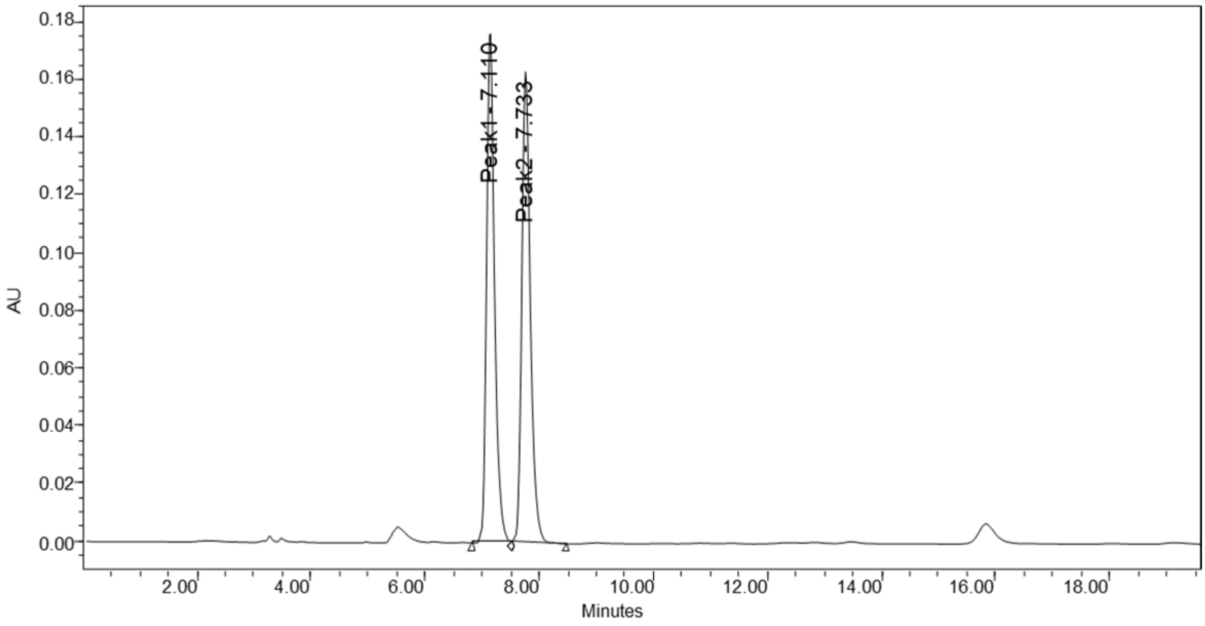

	Peak Name	RT (min)	Area (V*sec)	% Area	Height (V)	% Height
1	Peak1	7.110	1720818	50.12	177662	52.05
2	Peak2	7.733	1712783	49.88	163637	47.95

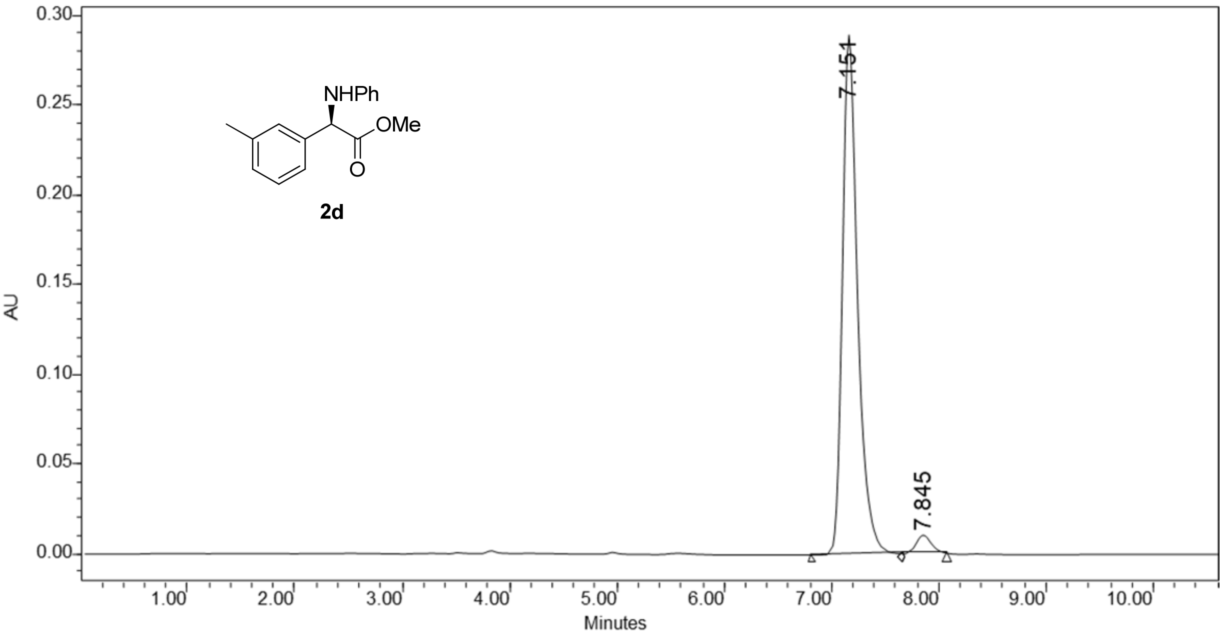

	RT (min)	Area (V*sec)	% Area	Height (V)	% Height
1	7.151	2844745	96.59	289664	96.58
2	7.845	100401	3.41	10250	3.42

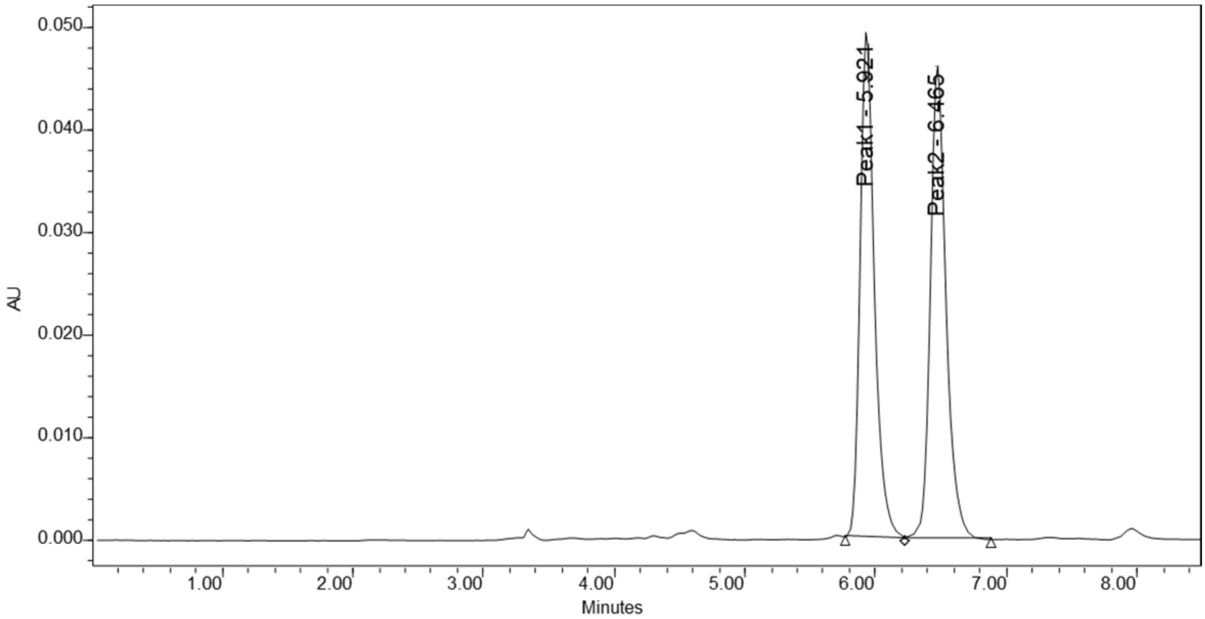

	Peak Name	RT (min)	Area (V*sec)	% Area	Height (V)	% Height
1	Peak1	5.921	375820	49.30	49418	51.71
2	Peak2	6.465	386507	50.70	46150	48.29

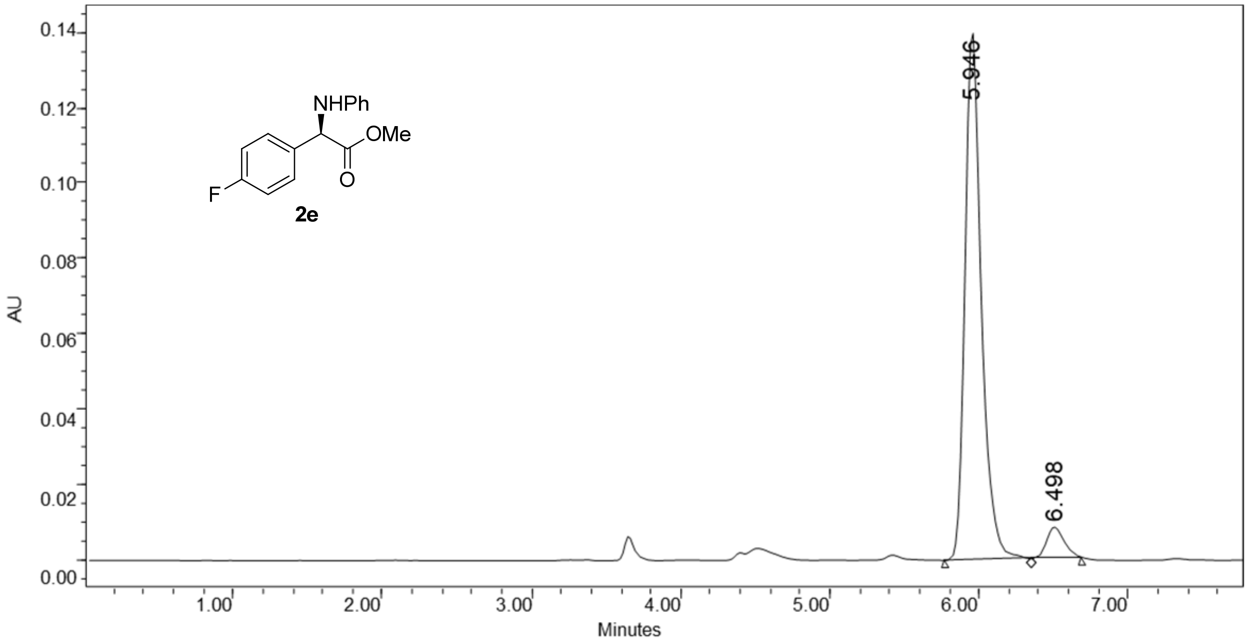

	RT (min)	Area (V*sec)	% Area	Height (V)	% Height
1	5.946	1075237	94.11	140073	94.44
2	6.498	67295	5.89	8248	5.56

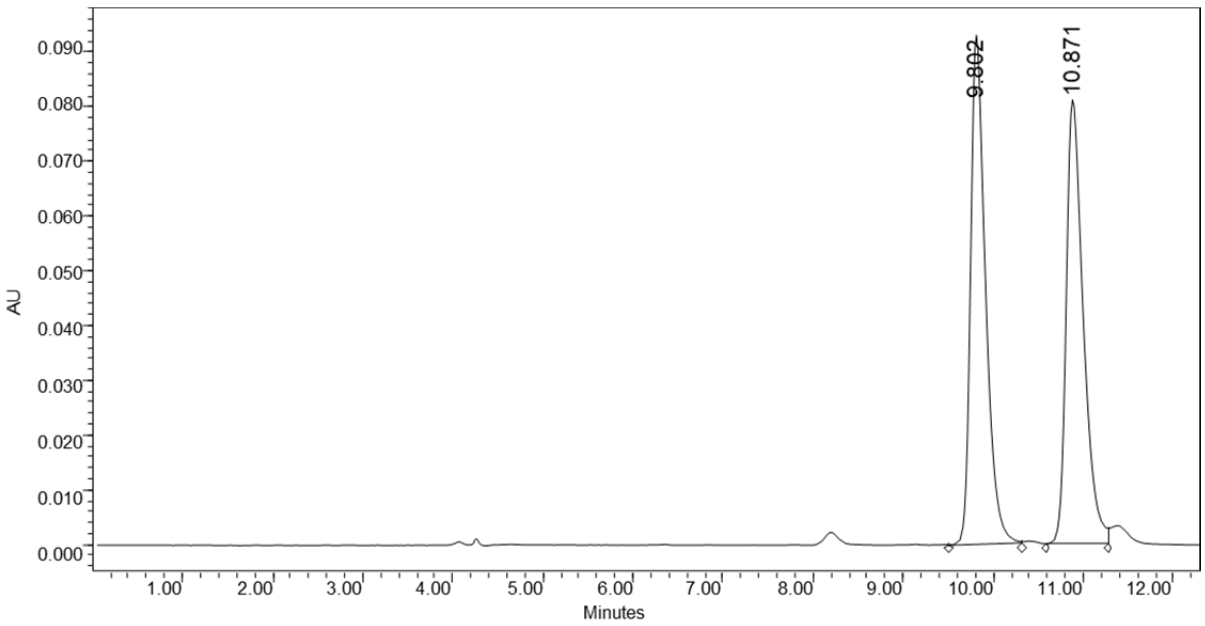

	RT (min)	Area (V*sec)	% Area	Height (V)	% Height
1	9.802	1083728	50.25	93336	53.36
2	10.871	1072941	49.75	81583	46.64

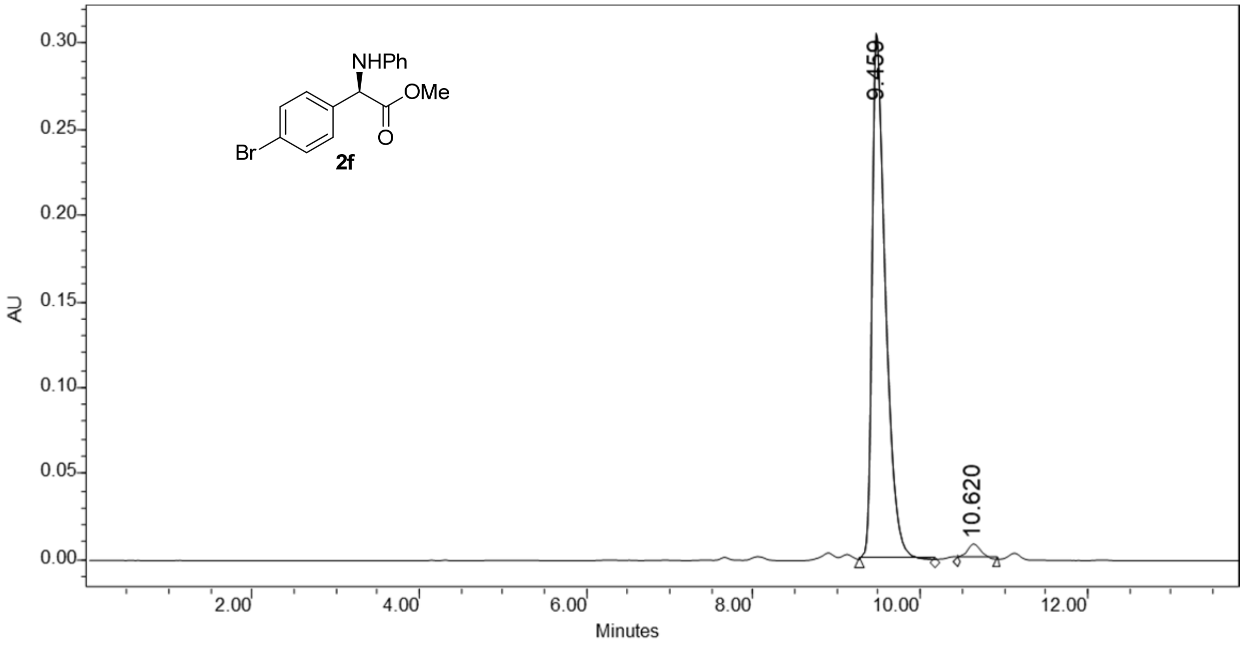

	RT (min)	Area (V*sec)	% Area	Height (V)	% Height
1	9.459	3409387	96.97	309370	97.20
2	10.620	106554	3.03	8915	2.80

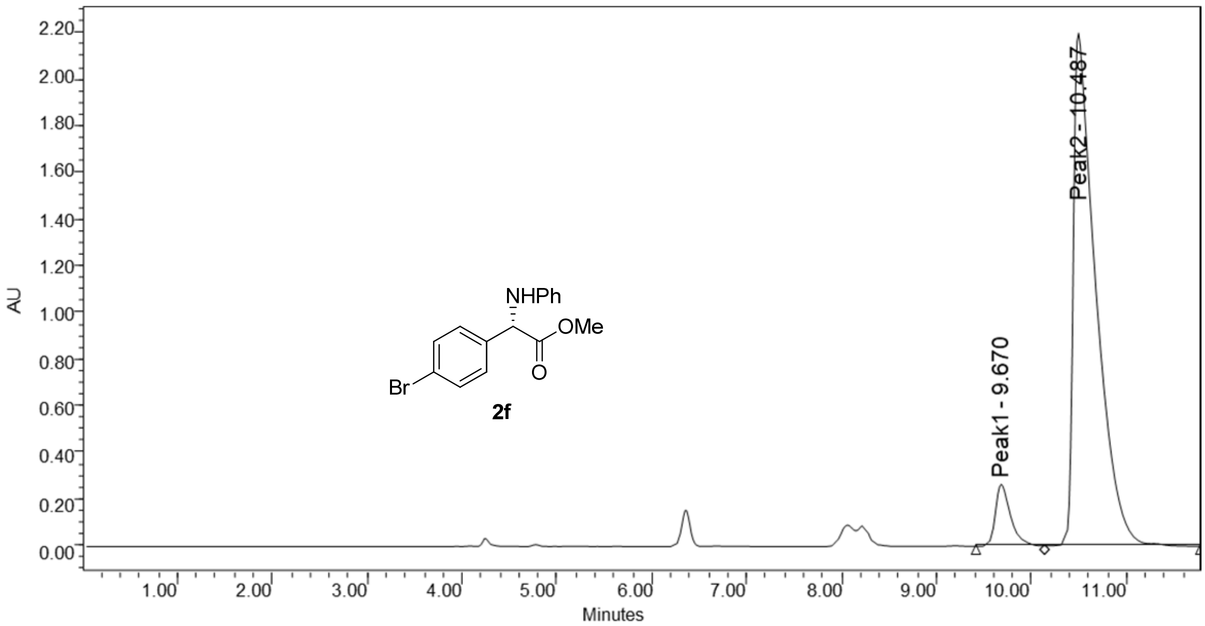

	Peak Name	RT (min)	Area (V*sec)	% Area	Height (V)	% Height
1	Peak1	9.670	2967760	7.58	266415	10.78
2	Peak2	10.487	36185311	92.42	2205346	89.22

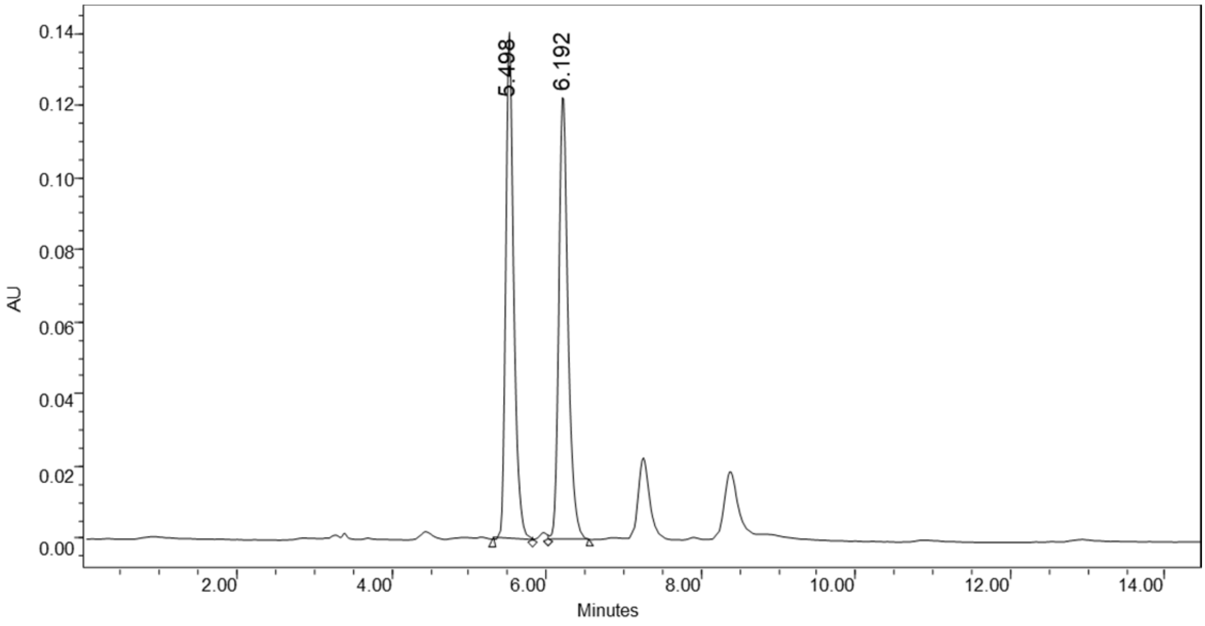

	RT (min)	Area (V*sec)	% Area	Height (V)	% Height
1	5.498	1010984	49.64	140333	53.11
2	6.192	1025505	50.36	123898	46.89

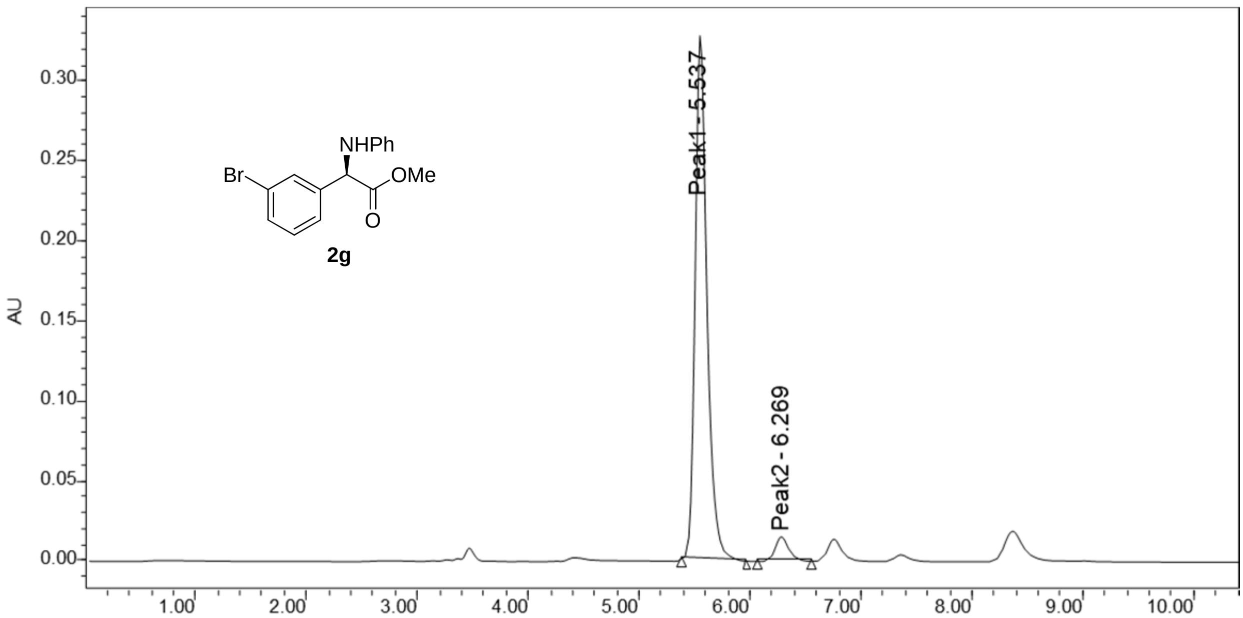

	Peak Name	RT (min)	Area (V*sec)	% Area	Height (V)	% Height
1	Peak1	5.537	2431987	94.95	327772	95.50
2	Peak2	6.269	129445	5.05	15452	4.50

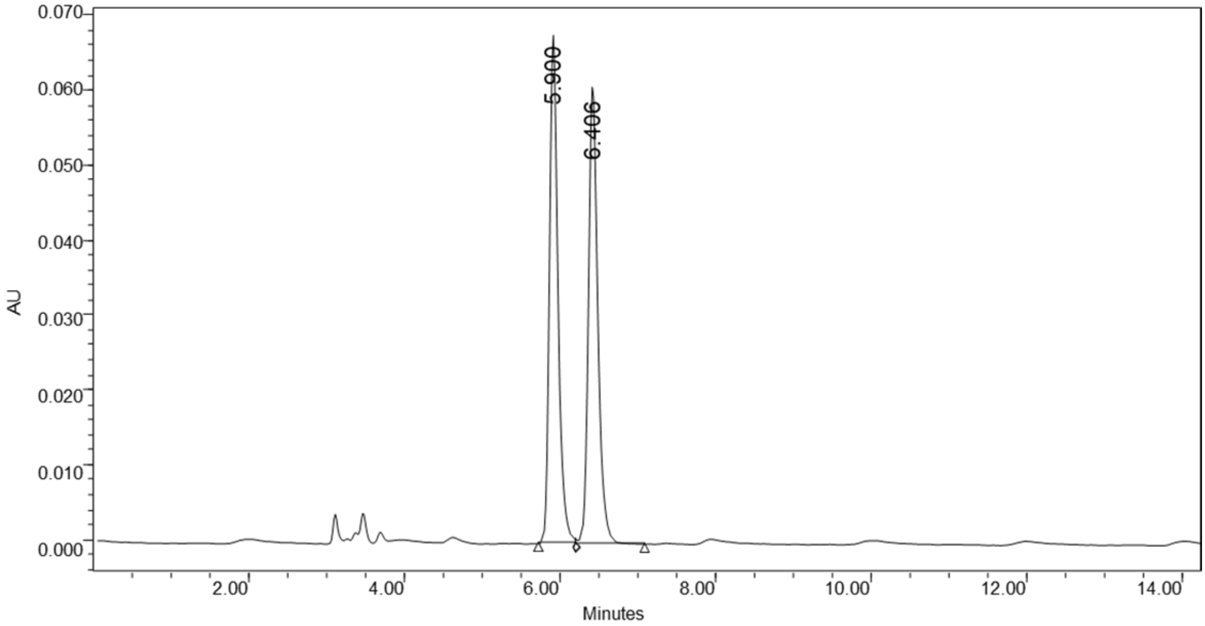

	Peak Name	RT (min)	Area (V*sec)	% Area	Height (V)	% Height
1	Peak1	5.900	517795	50.20	67675	52.51
2	Peak2	6.406	513719	49.80	61206	47.49

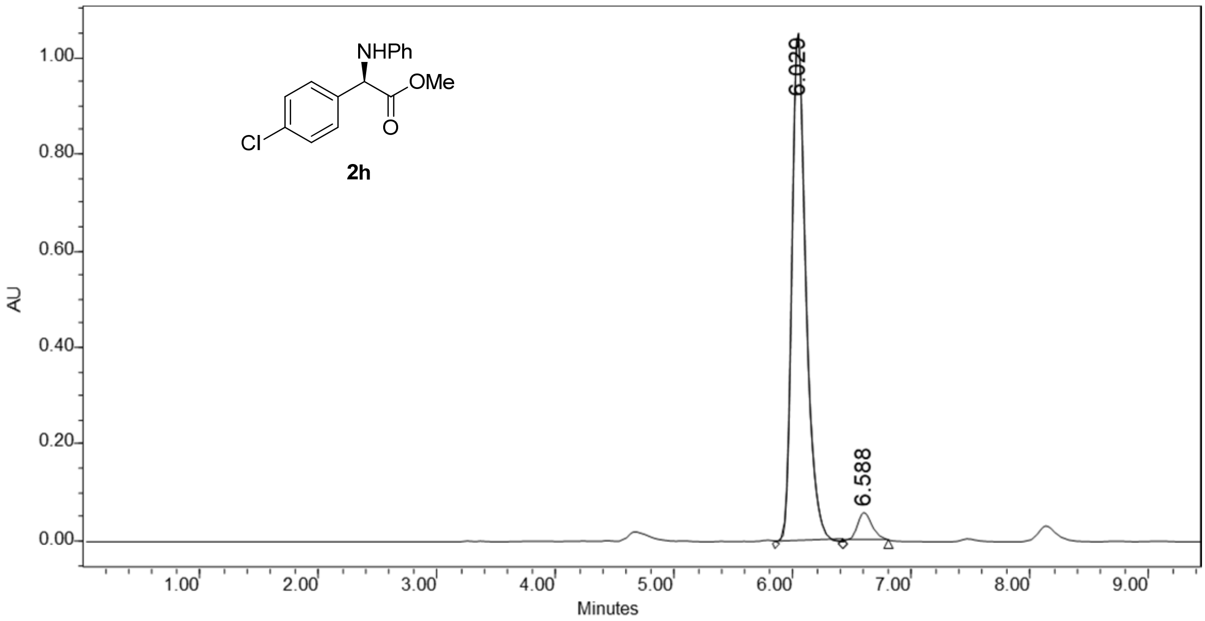

	RT (min)	Area (V*sec)	% Area	Height (V)	% Height
1	6.029	8685087	94.64	1051470	94.79
2	6.588	491895	5.36	57821	5.21

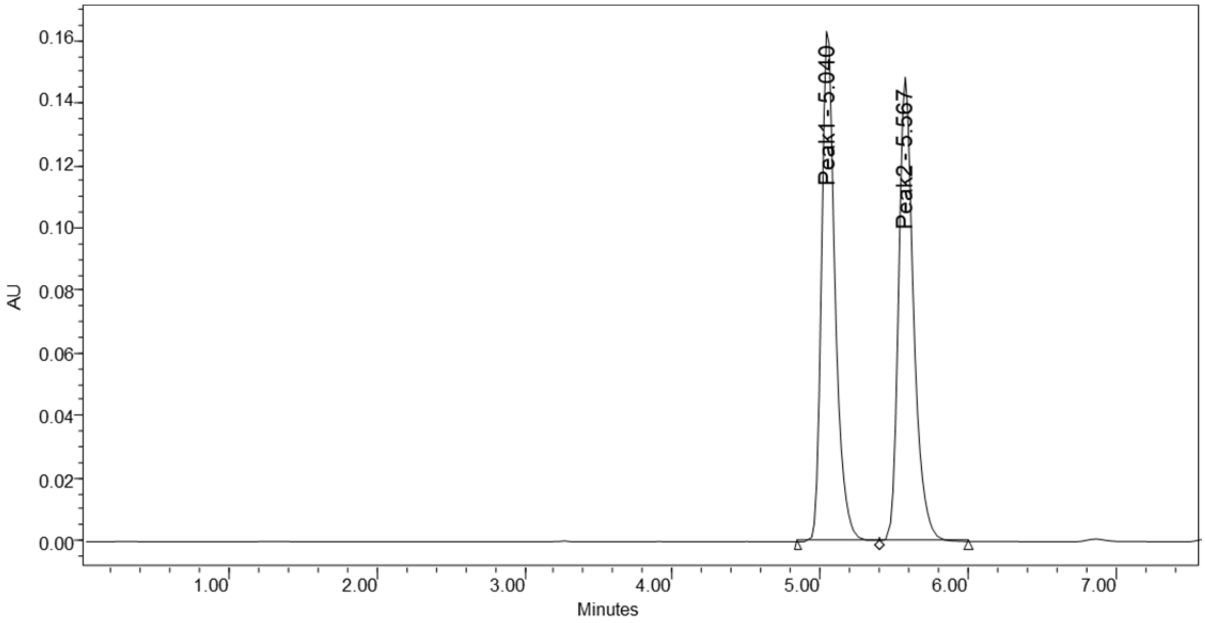

	Peak Name	RT (min)	Area (V*sec)	% Area	Height (V)	% Height
1	Peak1	5.040	1046556	49.94	163758	52.58
2	Peak2	5.567	1048882	50.06	147672	47.42

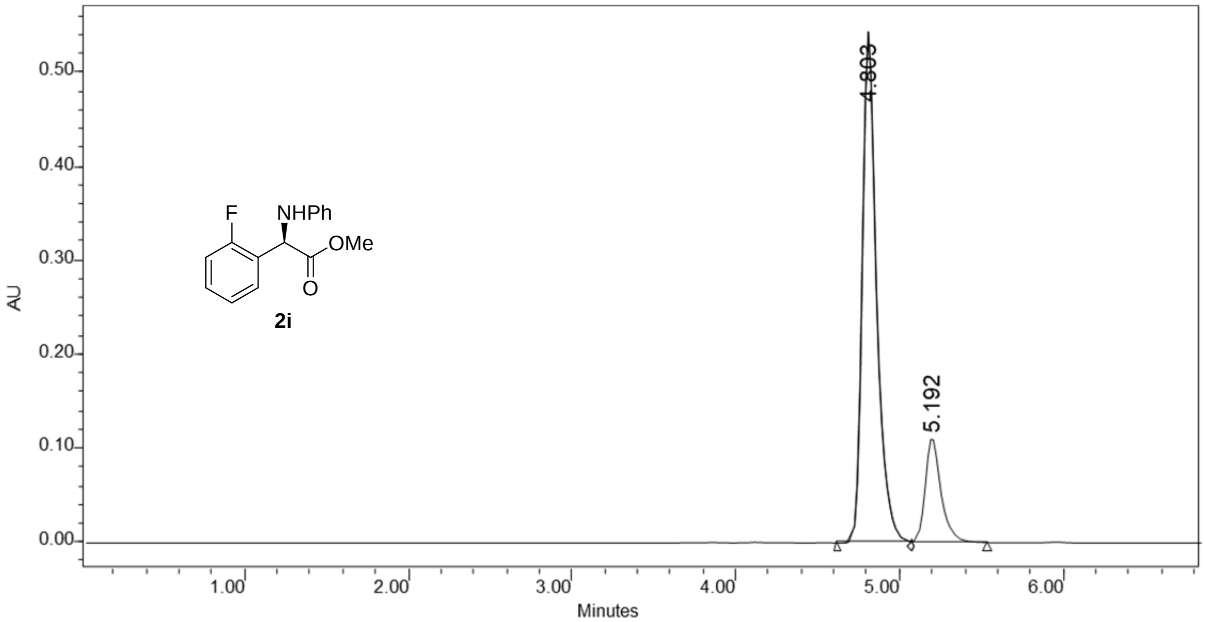

	RT (min)	Area (V*sec)	% Area	Height (V)	% Height
1	4.803	3308738	81.52	538800	82.91
2	5.192	750005	18.48	111091	17.09

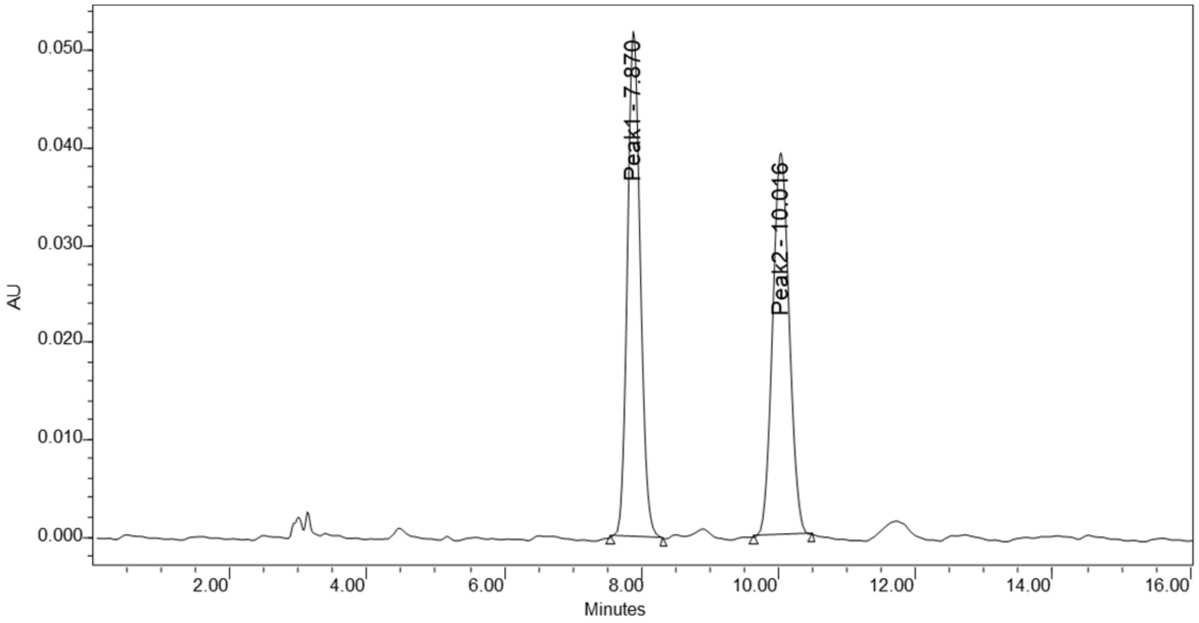

	Peak Name	RT (min)	Area (V*sec)	% Area	Height (V)	% Height
1	Peak1	7.870	678932	50.61	52114	56.95
2	Peak2	10.016	662647	49.39	39391	43.05

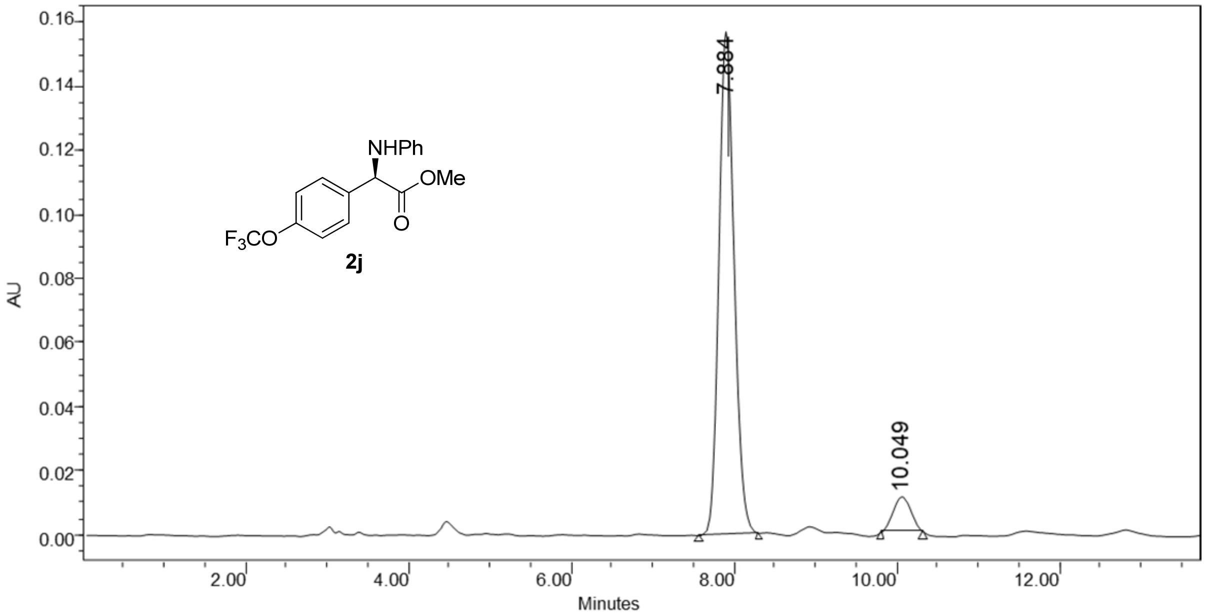

	RT (min)	Area (V*sec)	% Area	Height (V)	% Height
1	7.884	2035553	92.15	156888	93.34
2	10.049	173284	7.85	11189	6.66

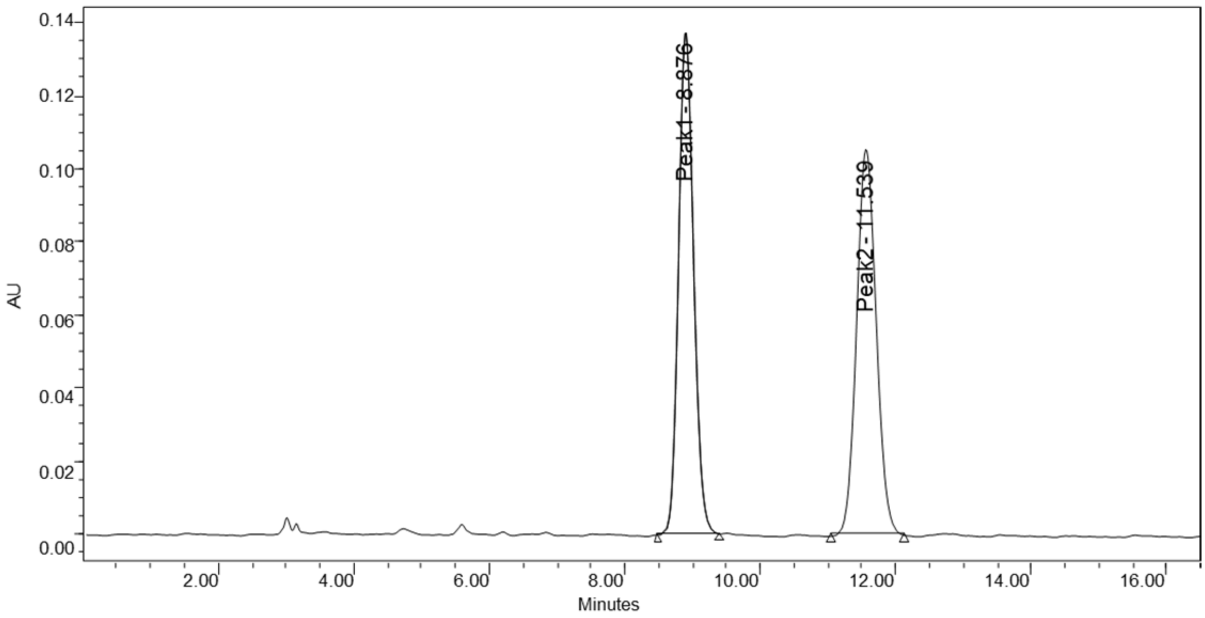

	Peak Name	RT (min)	Area (V*sec)	% Area	Height (V)	% Height
1	Peak1	8.876	2123935	49.85	137526	56.51
2	Peak2	11.539	2136568	50.15	105838	43.49

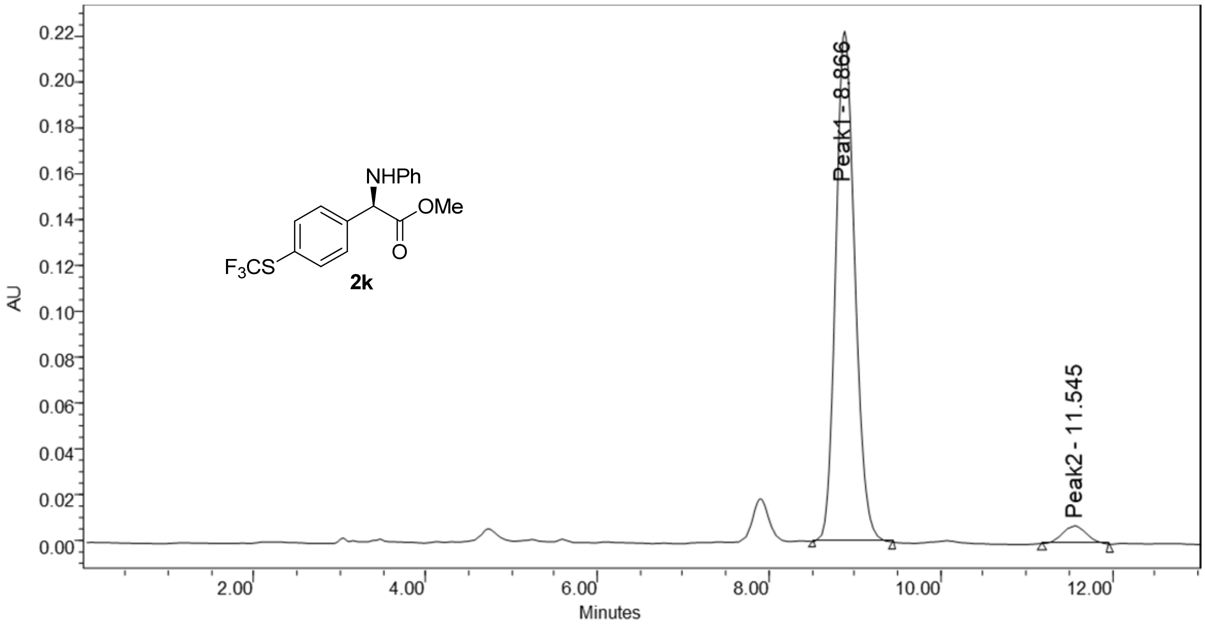

	Peak Name	RT (min)	Area (V*sec)	% Area	Height (V)	% Height
1	Peak1	8.866	3439505	95.71	222007	96.61
2	Peak2	11.545	154255	4.29	7799	3.39

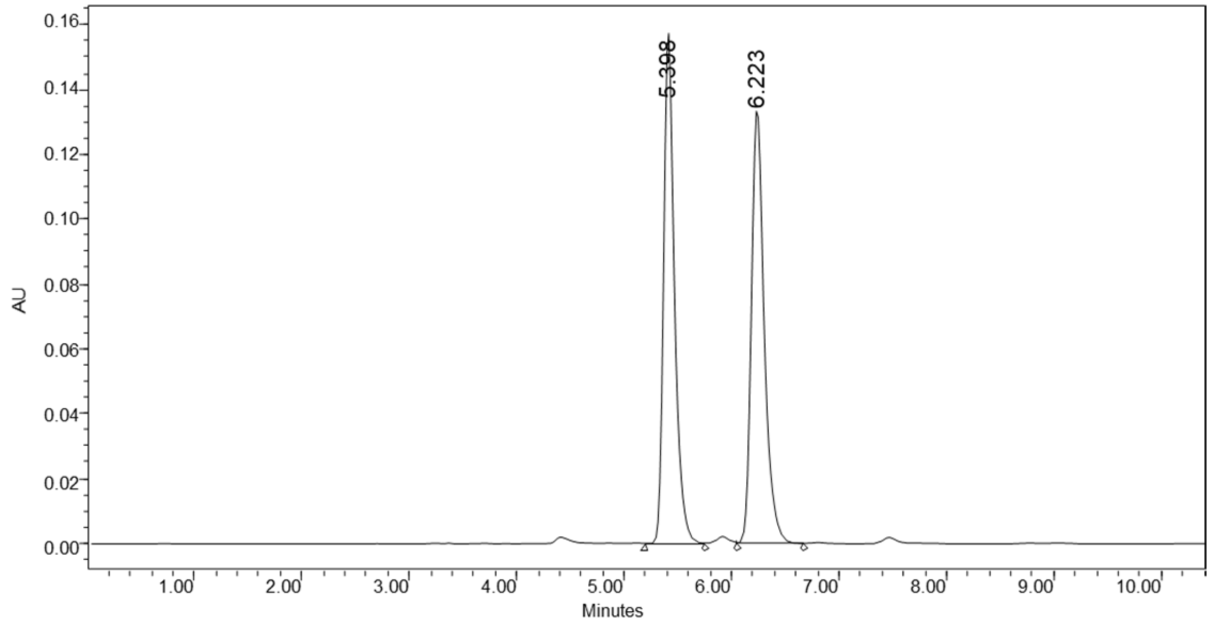

	RT (min)	Area (V*sec)	% Area	Height (V)	% Height
1	5.398	1112041	49.86	157199	53.91
2	6.223	1118284	50.14	134393	46.09

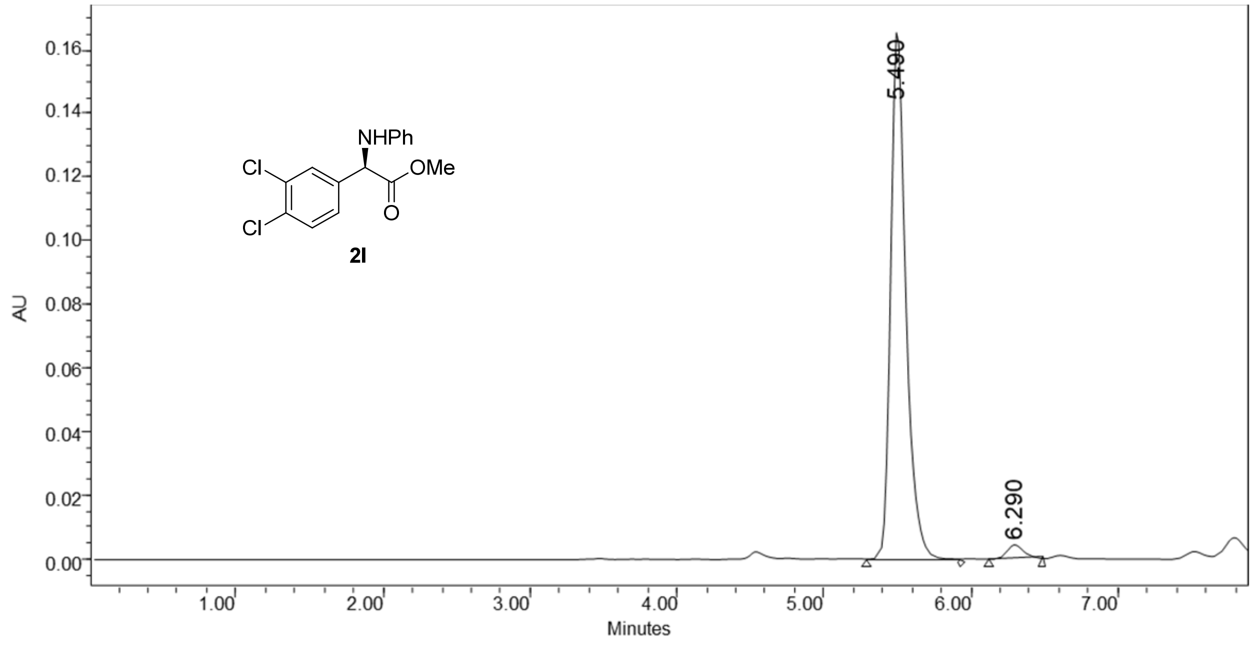

	RT (min)	Area (V*sec)	% Area	Height (V)	% Height
1	5.490	1190513	97.15	166652	97.40
2	6.290	34900	2.85	4441	2.60

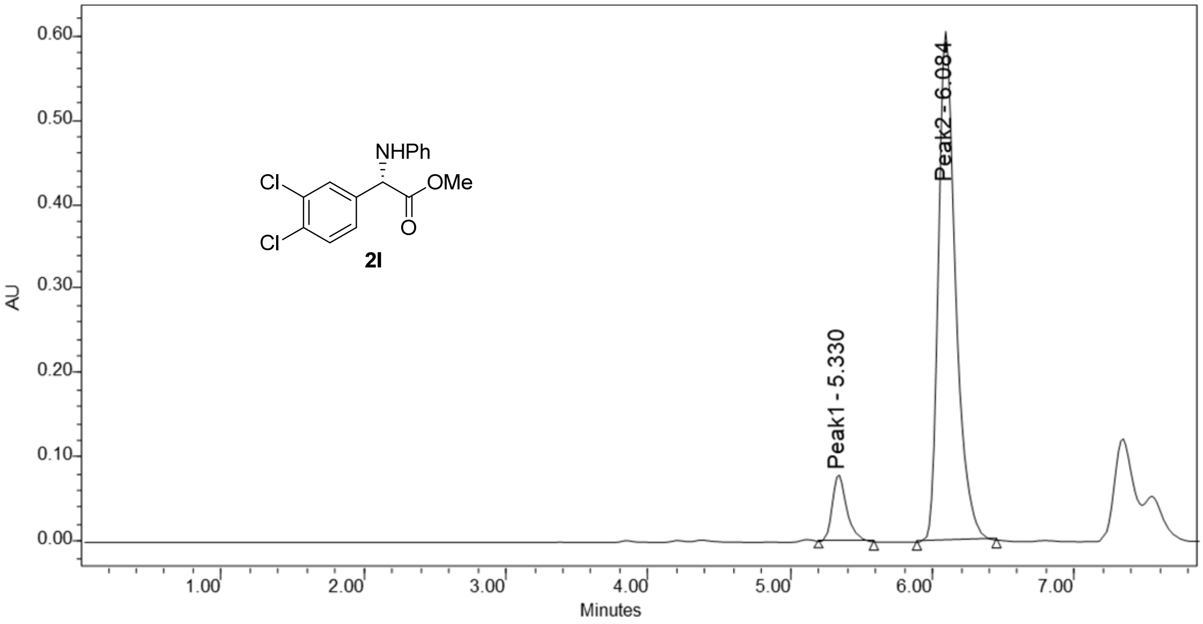

	Peak Name	RT (min)	Area (V*sec)	% Area	Height (V)	% Height
1	Peak1	5.330	559447	9.87	78665	11.53
2	Peak2	6.084	5109863	90.13	603534	88.47

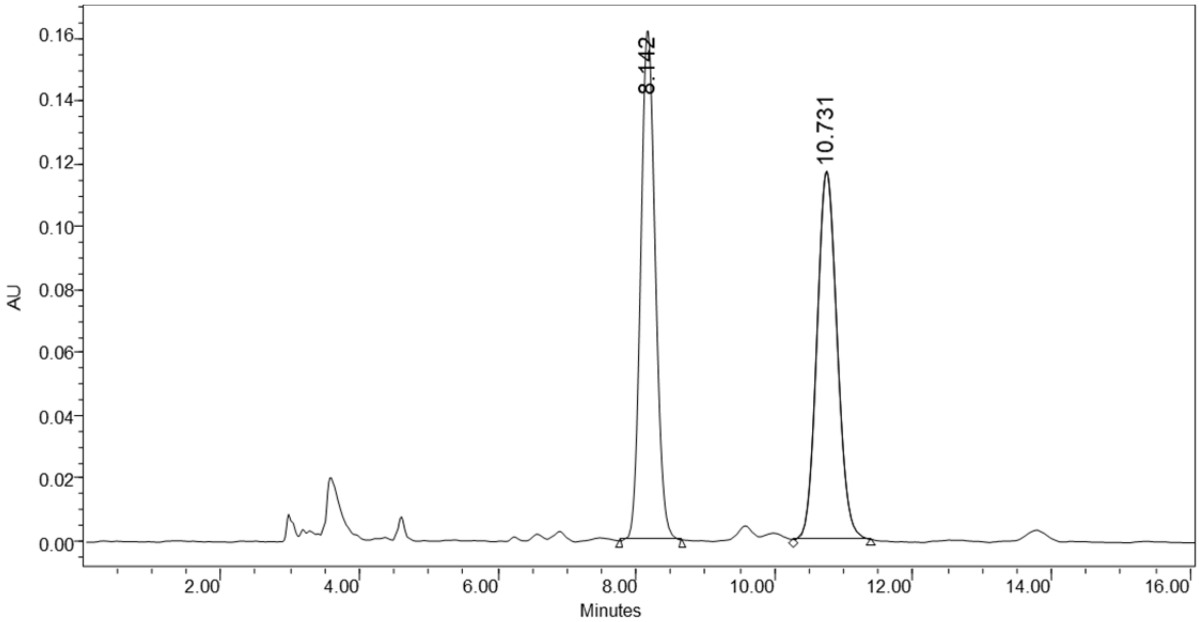

	RT (min)	Area (V*sec)	% Area	Height (V)	% Height
1	8.142	2398961	50.09	162558	58.06
2	10.731	2390170	49.91	117419	41.94

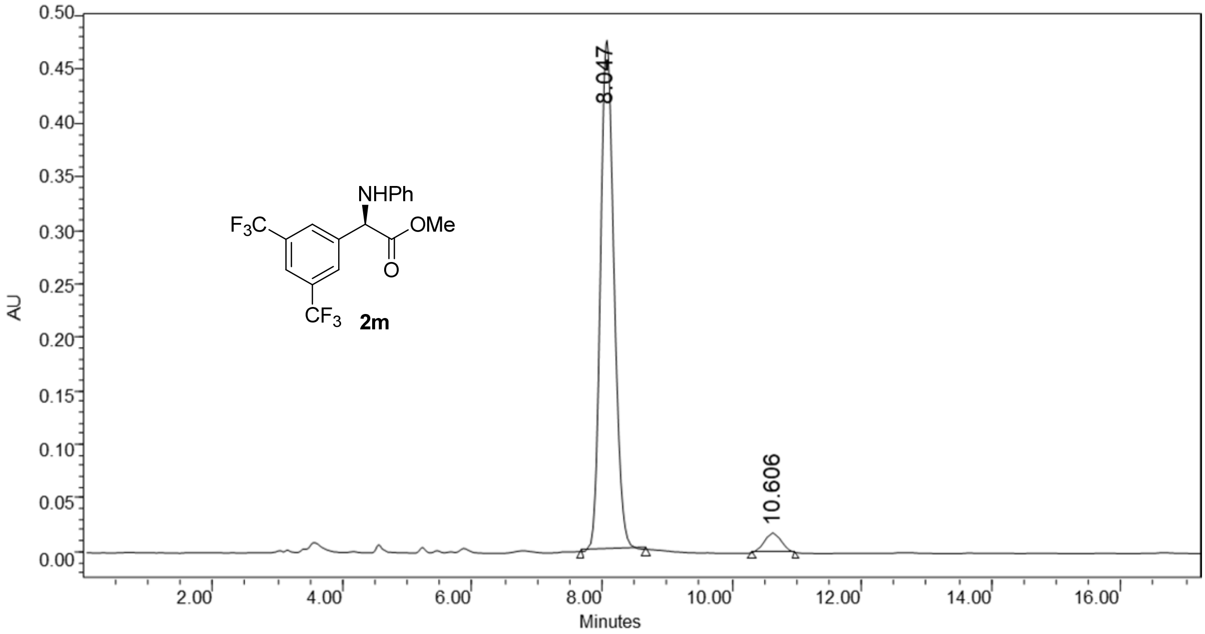

	RT (min)	Area (V*sec)	% Area	Height (V)	% Height
1	8.047	6961056	95.39	476211	96.32
2	10.606	336522	4.61	18192	3.68

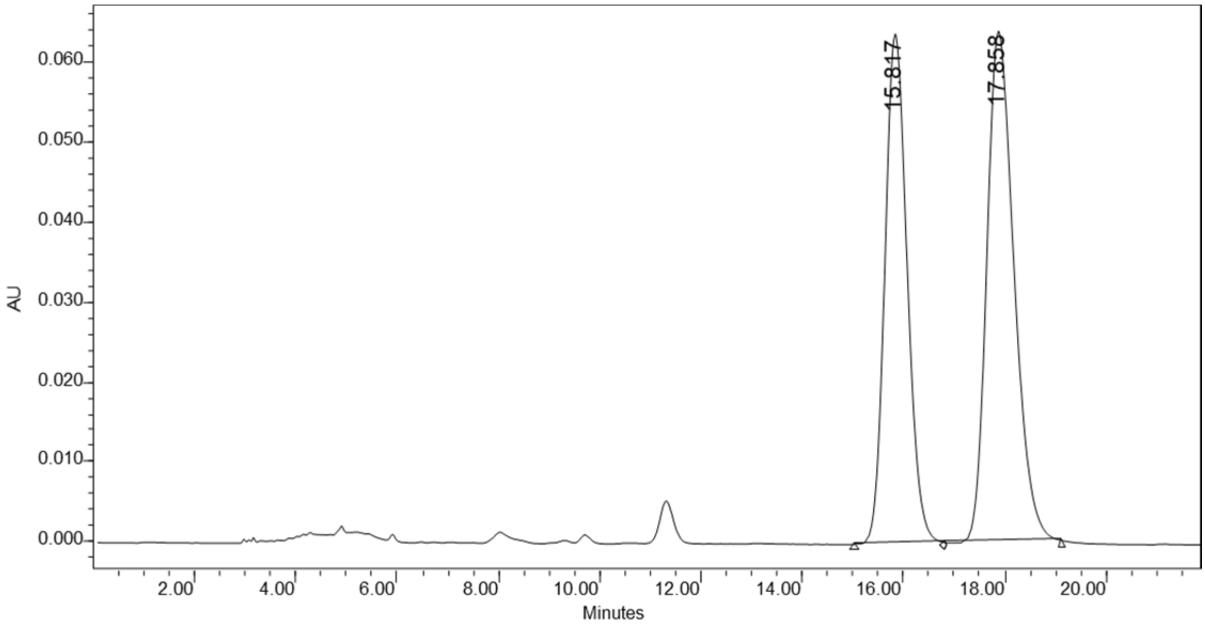

	RT (min)	Area (V*sec)	% Area	Height (V)	% Height
1	15.817	1950274	44.93	63808	49.95
2	17.858	2390112	55.07	63942	50.05

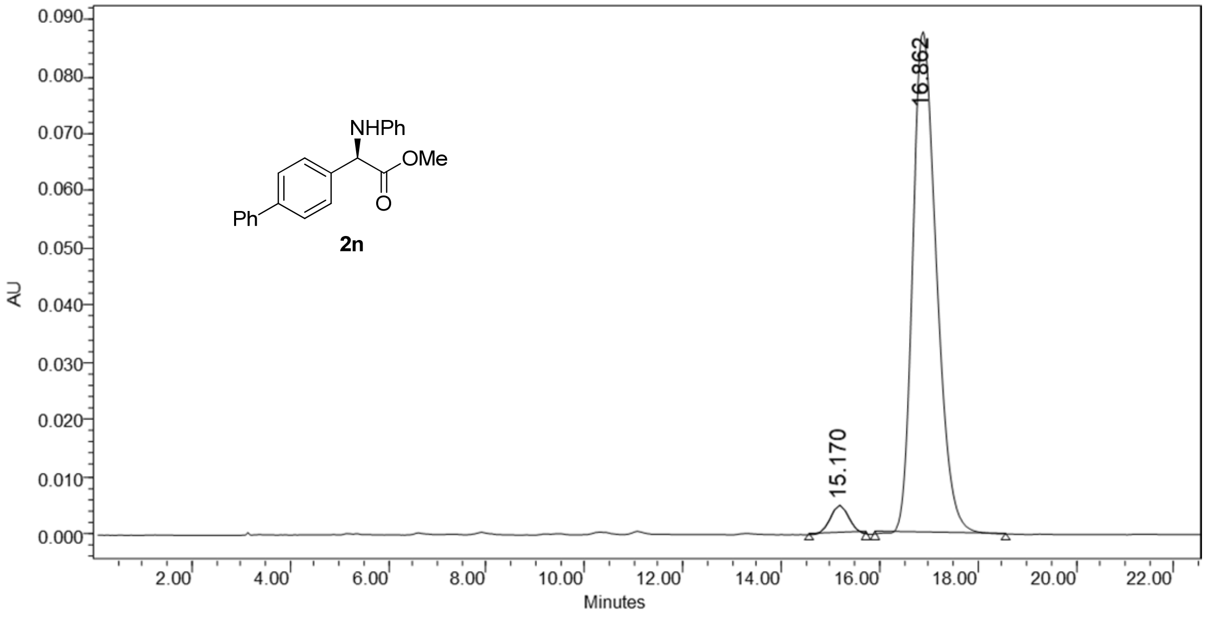

	RT (min)	Area (V*sec)	% Area	Height (V)	% Height
1	15.170	134511	4.35	5047	5.44
2	16.862	2957713	95.65	87766	94.56

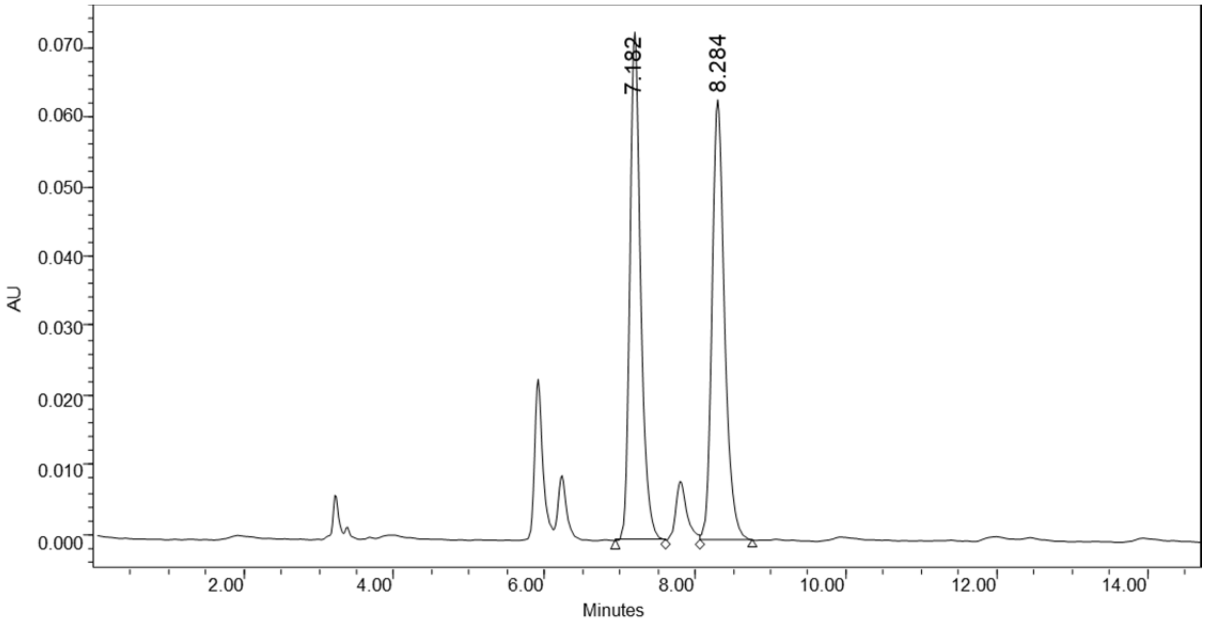

	RT (min)	Area (V*sec)	% Area	Height (V)	% Height
1	7.182	727917	49.85	73024	53.56
2	8.284	732319	50.15	63308	46.44

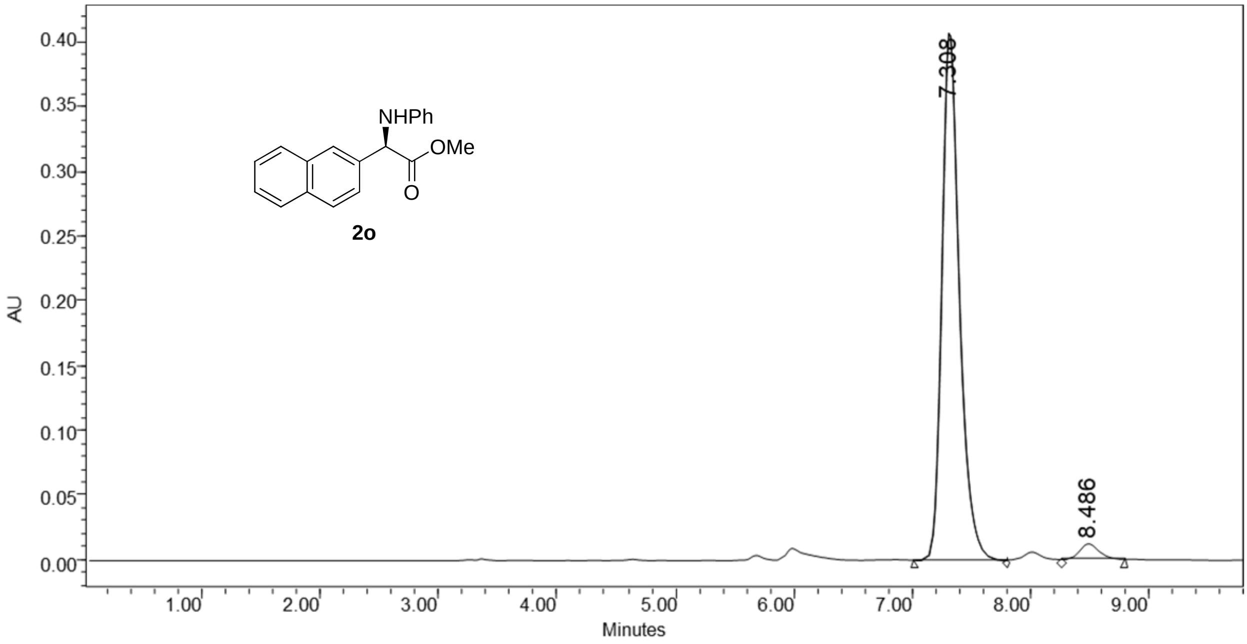

	RT (min)	Area (V*sec)	% Area	Height (V)	% Height
1	7.308	4214876	96.80	409787	97.10
2	8.486	139293	3.20	12257	2.90

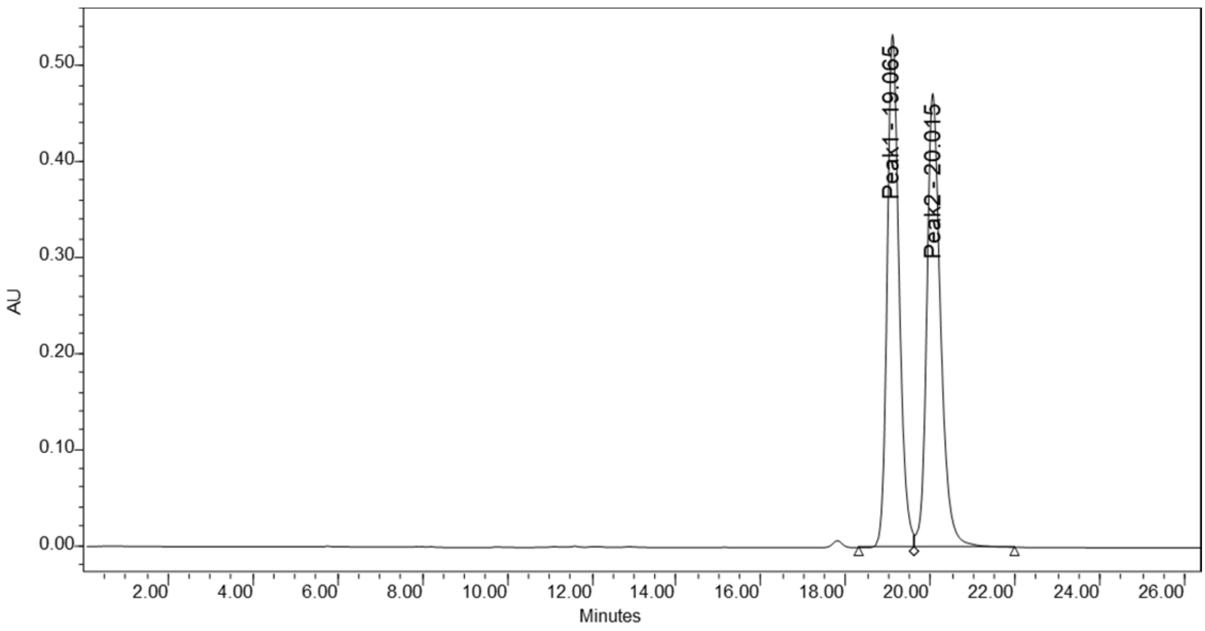

	Peak Name	RT (min)	Area (V*sec)	% Area	Height (V)	% Height
1	Peak1	19.065	10474093	49.46	534294	53.06
2	Peak2	20.015	10704582	50.54	472762	46.94

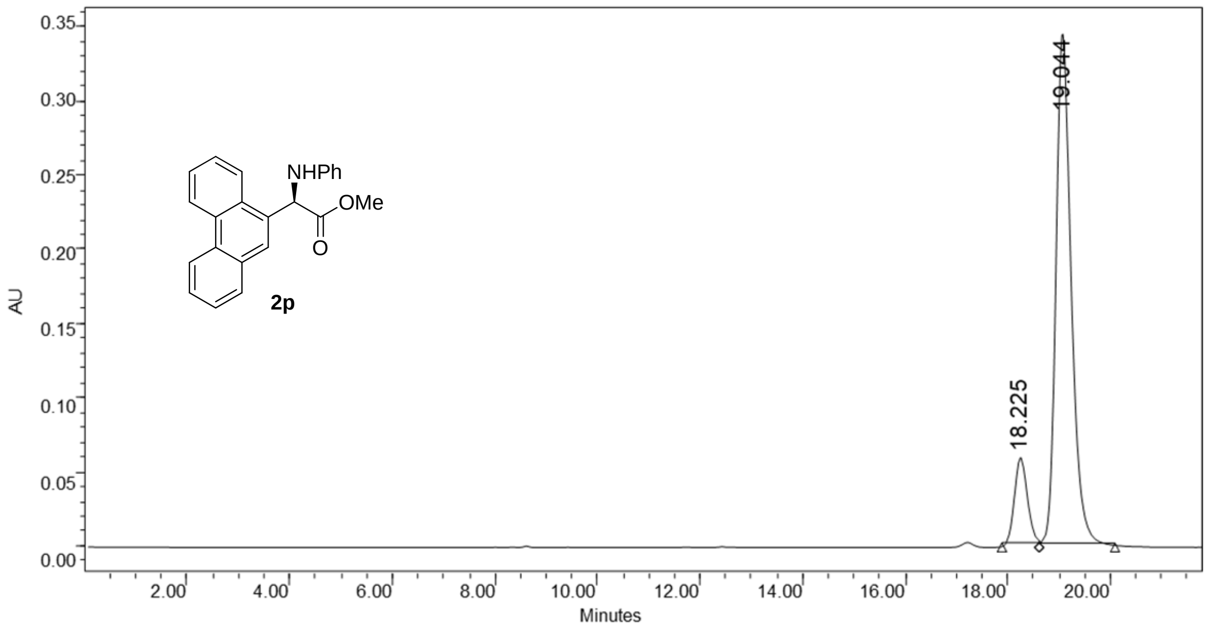

	RT (min)	Area (V*sec)	% Area	Height (V)	% Height
1	18.225	1061286	12.93	59258	14.67
2	19.044	7149096	87.07	344657	85.33

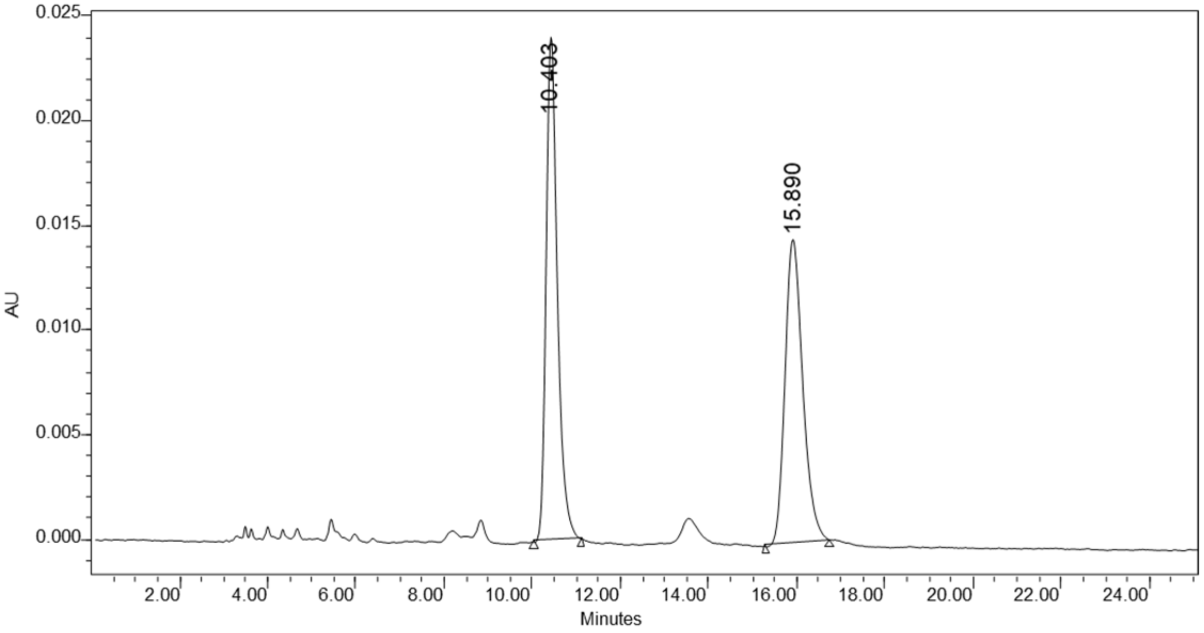

	RT (min)	Area (V*sec)	% Area	Height (V)	% Height
1	10.403	428410	51.12	24080	62.33
2	15.890	409654	48.88	14555	37.67

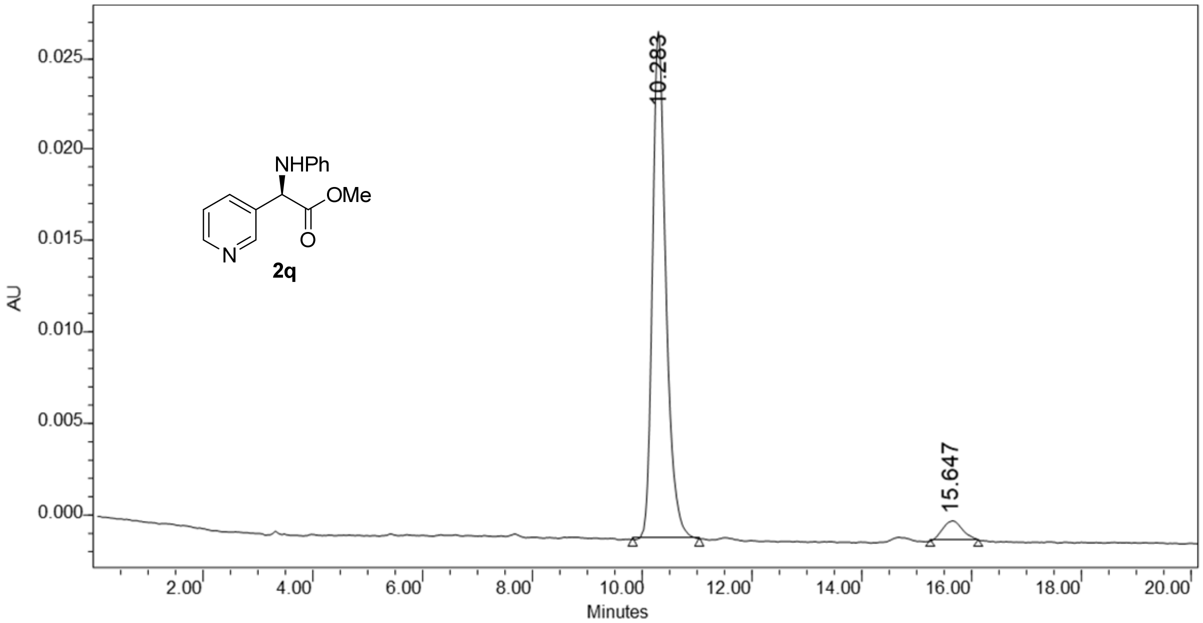

	RT (min)	Area (V*sec)	% Area	Height (V)	% Height
1	10.283	474165	94.48	27775	96.26
2	15.647	27715	5.52	1079	3.74

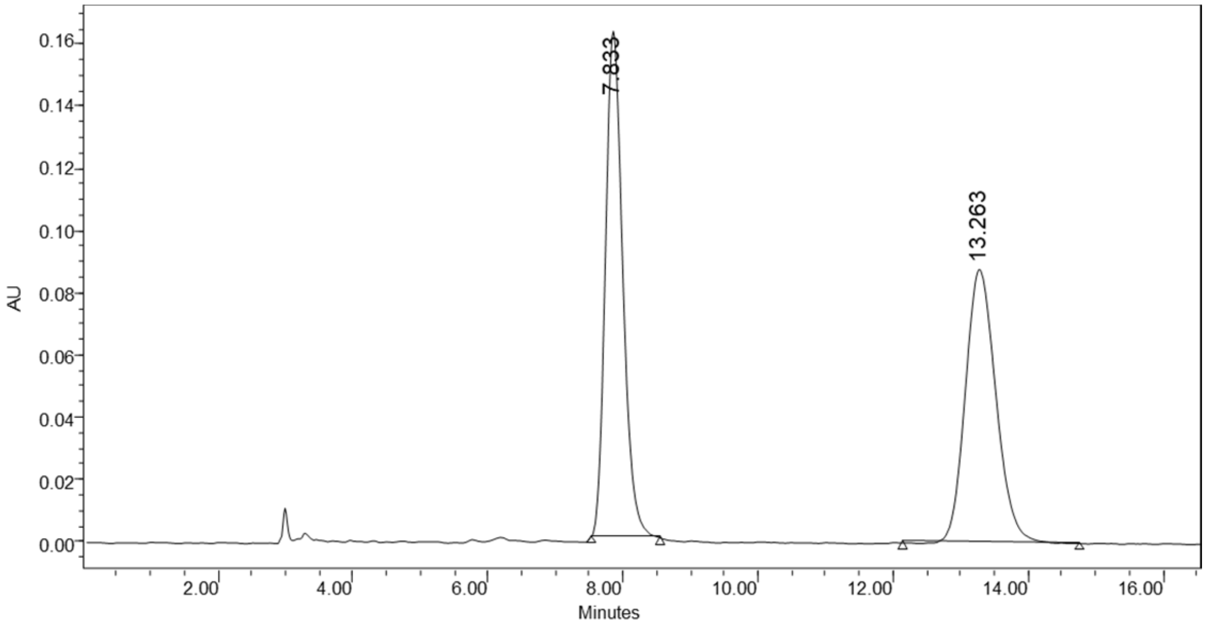

	RT (min)	Area (V*sec)	% Area	Height (V)	% Height
1	7.833	2915384	51.08	162710	64.89
2	13.263	2792057	48.92	88049	35.11

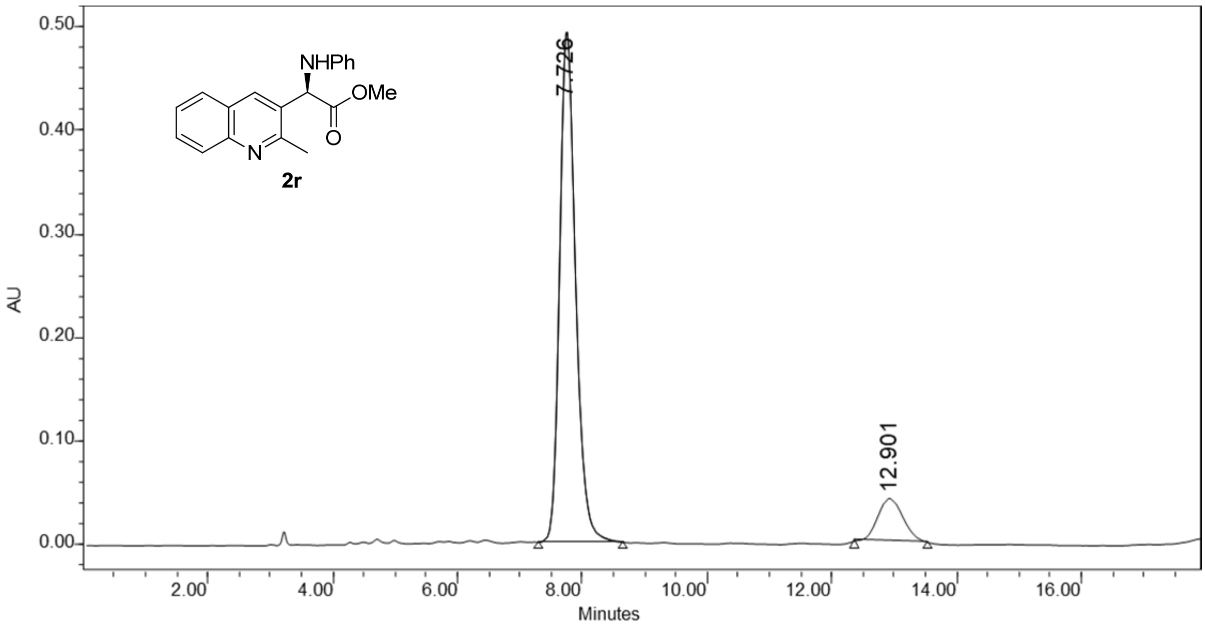

	RT (min)	Area (V*sec)	% Area	Height (V)	% Height
1	7.726	8771786	87.95	493257	92.16
2	12.901	1201776	12.05	41945	7.84

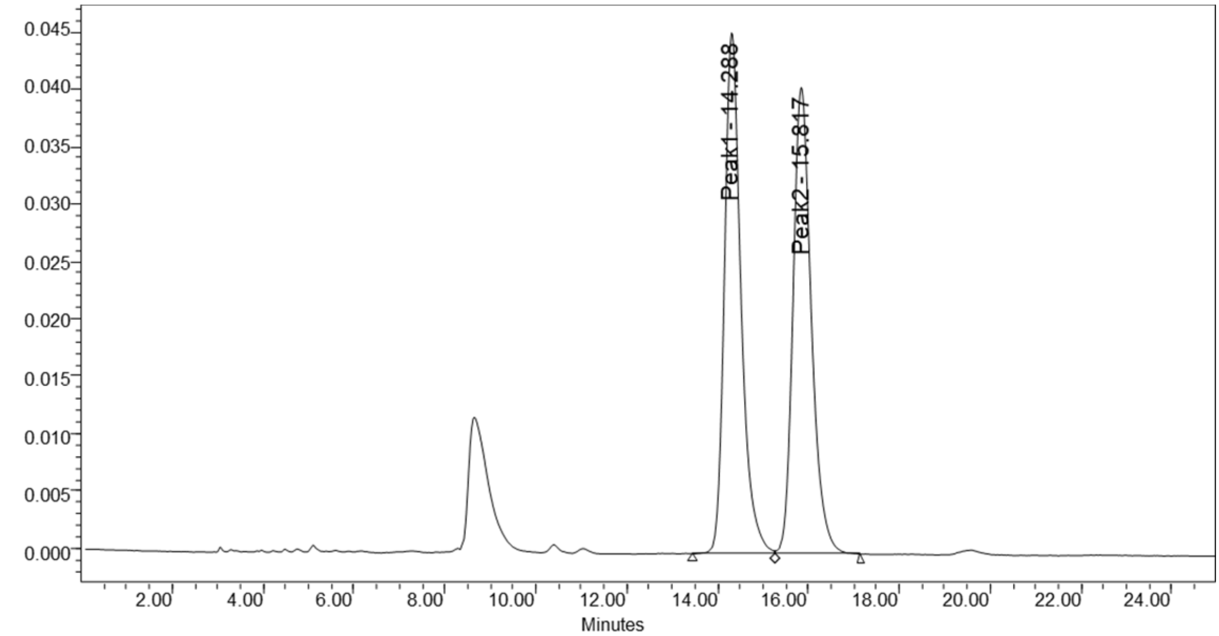

	Peak Name	RT (min)	Area (V*sec)	% Area	Height (V)	% Height
1	Peak1	14.288	1157905	50.37	45486	52.76
2	Peak2	15.817	1140772	49.63	40724	47.24

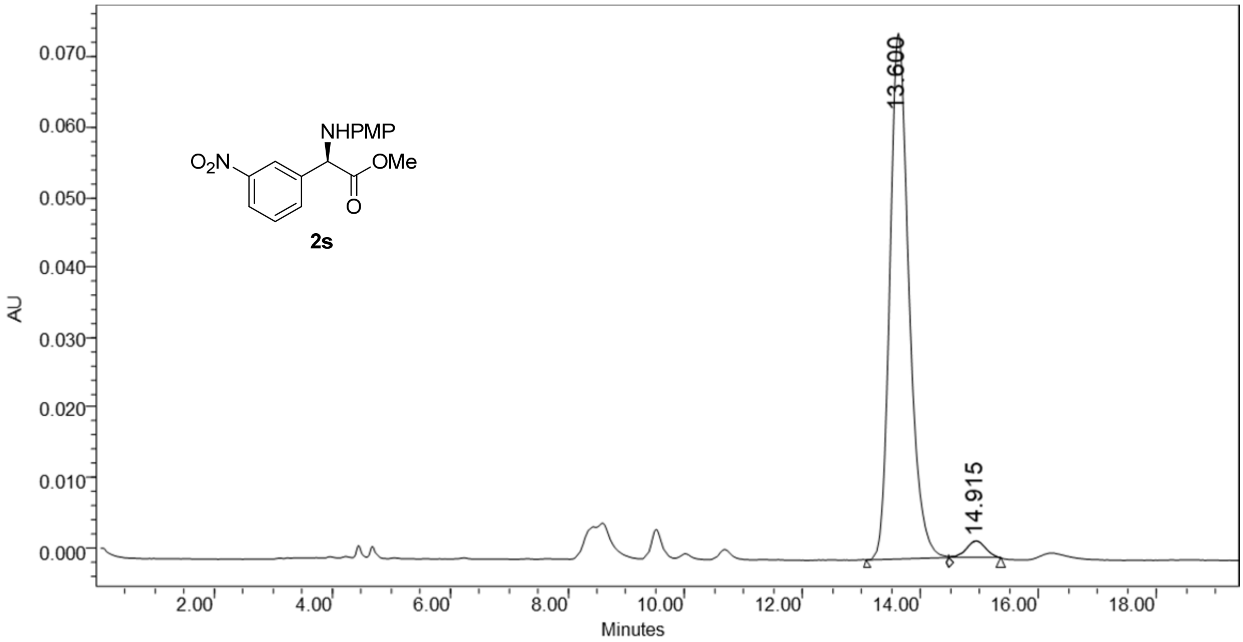

2s

	RT (min)	Area (V*sec)	% Area	Height (V)	% Height
1	13.600	1701379	96.49	75293	96.73
2	14.915	61822	3.51	2543	3.27

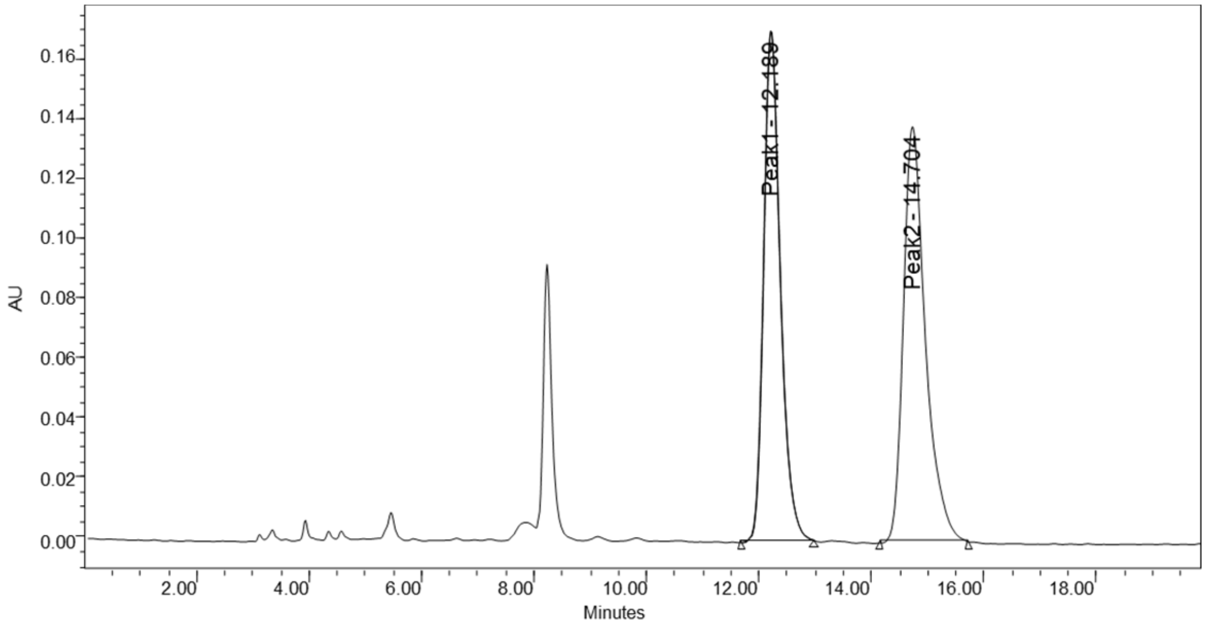

	Peak Name	RT (min)	Area (V*sec)	% Area	Height (V)	% Height
1	Peak1	12.189	3539321	48.20	171316	55.13
2	Peak2	14.704	3803650	51.80	139423	44.87

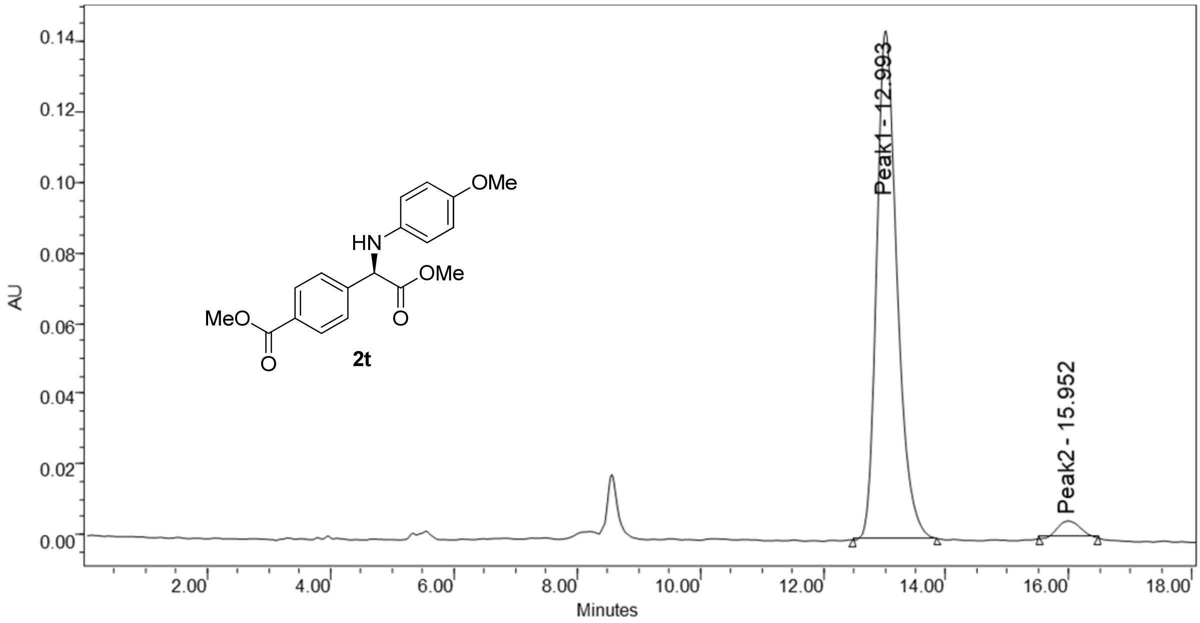

	Peak Name	RT (min)	Area (V*sec)	% Area	Height (V)	% Height
1	Peak1	12.993	3318708	96.21	144254	96.67
2	Peak2	15.952	130733	3.79	4967	3.33

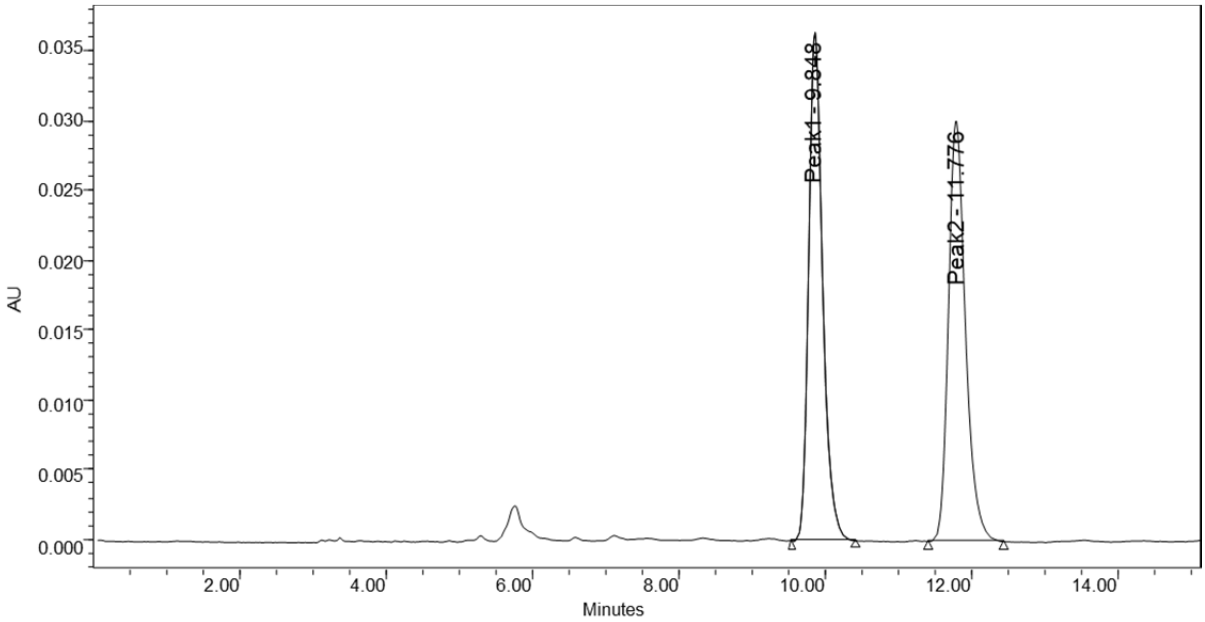

	Peak Name	RT (min)	Area (V*sec)	% Area	Height (V)	% Height
1	Peak1	9.848	484600	49.84	36462	54.68
2	Peak2	11.776	487664	50.16	30220	45.32

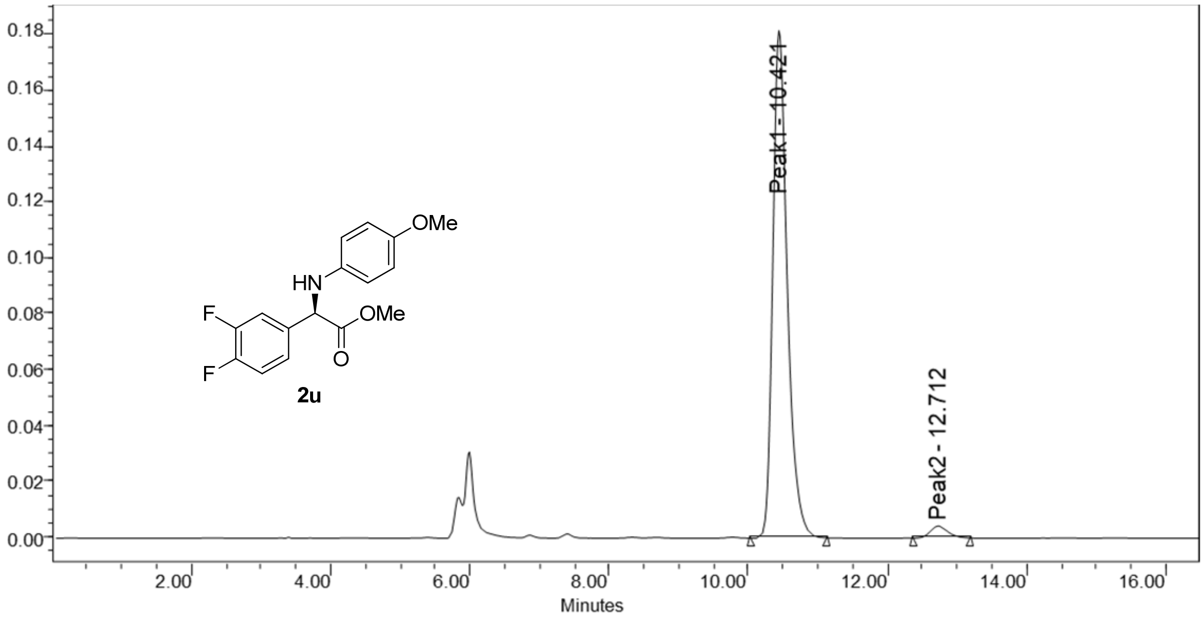

	Peak Name	RT (min)	Area (V*sec)	% Area	Height (V)	% Height
1	Peak1	10.421	2617022	97.15	181597	97.68
2	Peak2	12.712	76759	2.85	4317	2.32

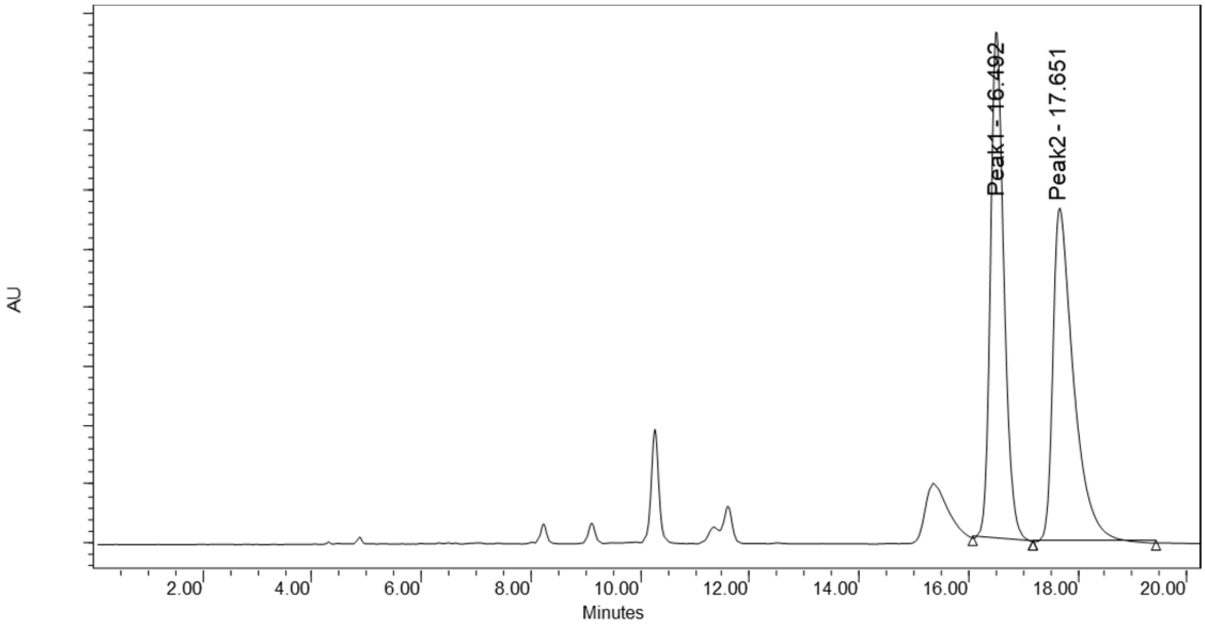

	Peak Name	RT (min)	Area (V*sec)	% Area	Height (V)	% Height
1	Peak1	16.492	1478483	49.53	86599	60.38
2	Peak2	17.651	1506706	50.47	56814	39.62

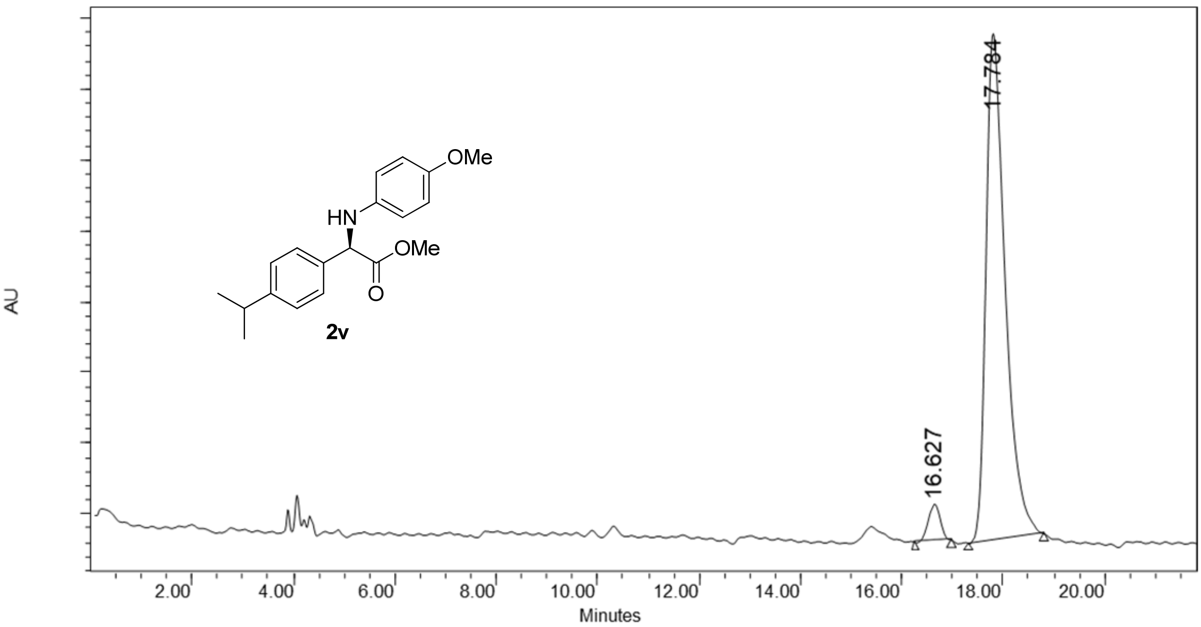

	RT (min)	Area (V*sec)	% Area	Height (V)	% Height
1	16.627	44973	4.54	2666	6.93
2	17.784	945031	95.46	35781	93.07

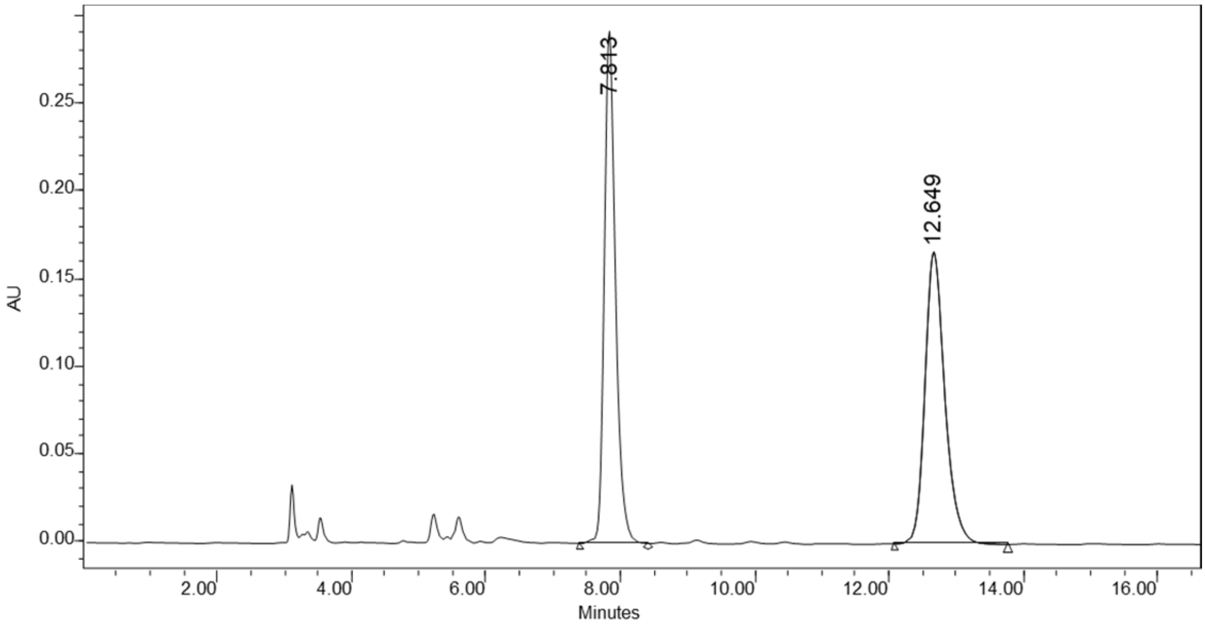

	RT (min)	Area (V*sec)	% Area	Height (V)	% Height
1	7.813	3391941	49.66	291985	63.67
2	12.649	3438310	50.34	166584	36.33

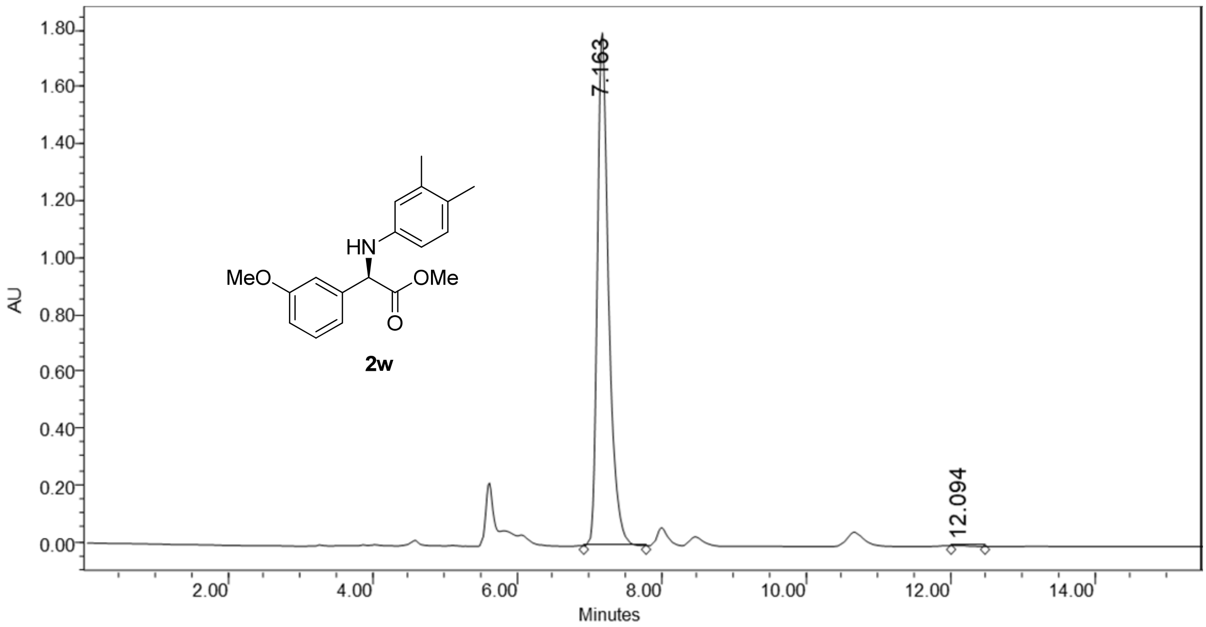

	RT (min)	Area (V*sec)	% Area	Height (V)	% Height
1	7.163	19629478	99.76	1806984	99.82
2	12.094	47379	0.24	3170	0.18

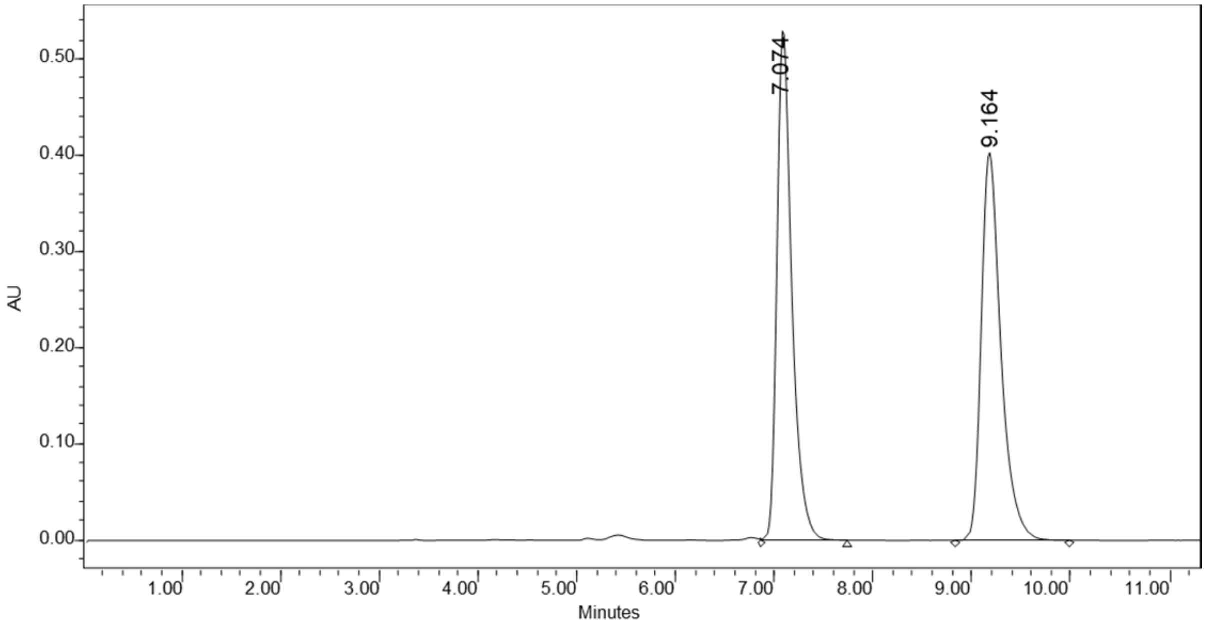

	RT (min)	Area (V*sec)	% Area	Height (V)	% Height
1	7.074	5545718	49.78	533046	56.91
2	9.164	5594382	50.22	403599	43.09

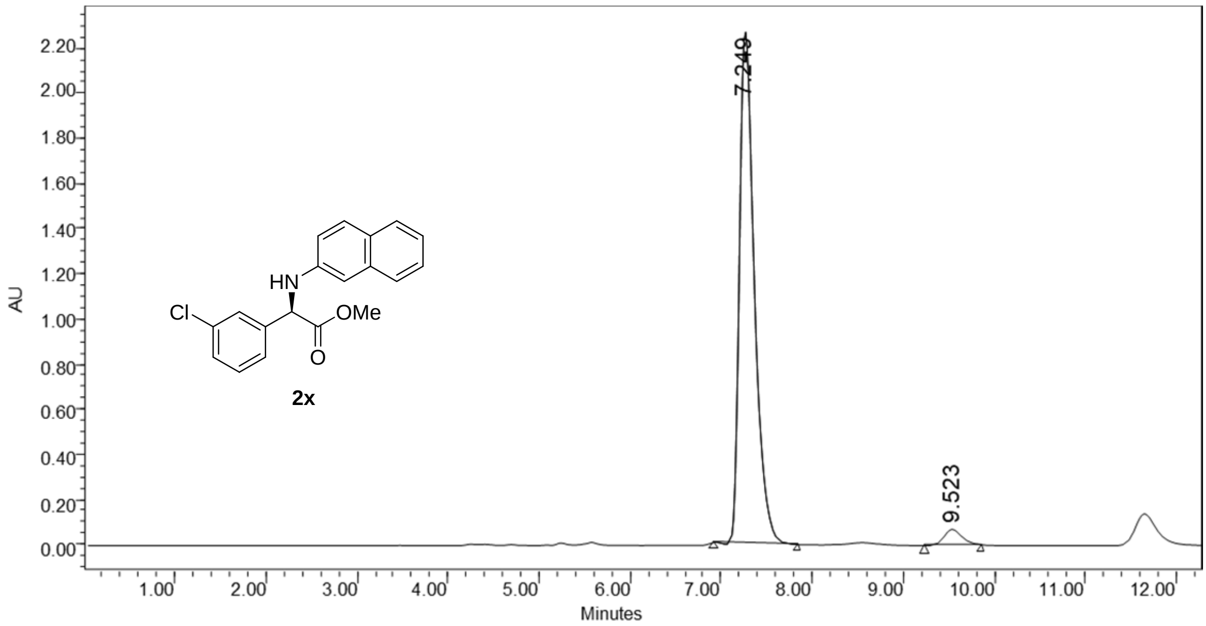

	RT (min)	Area (V*sec)	% Area	Height (V)	% Height
1	7.249	25817375	96.46	2264024	97.00
2	9.523	946232	3.54	70033	3.00

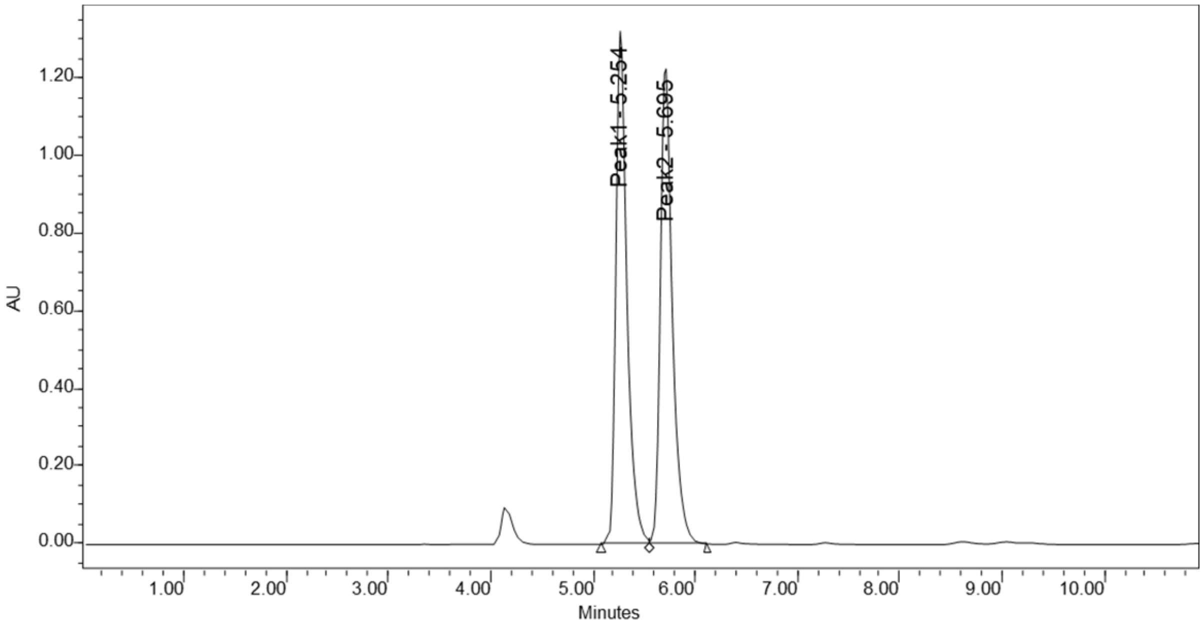

	Peak Name	RT (min)	Area (V*sec)	% Area	Height (V)	% Height
1	Peak1	5.254	9980608	49.86	1320927	51.83
2	Peak2	5.695	10038144	50.14	1227620	48.17

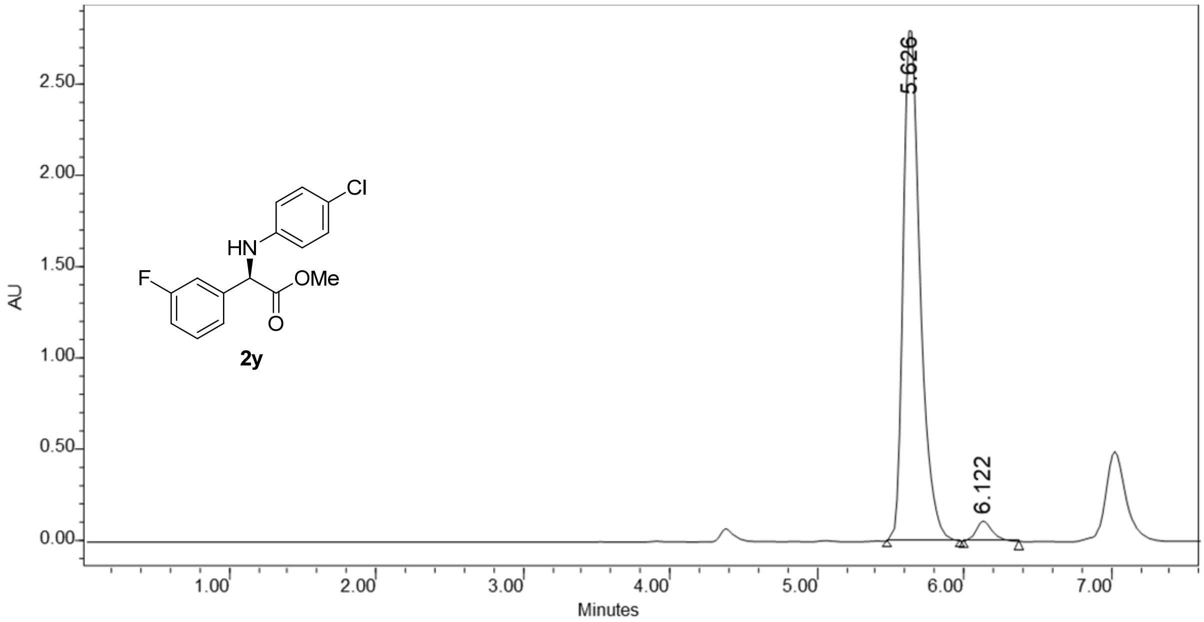

	RT (min)	Area (V*sec)	% Area	Height (V)	% Height
1	5.626	22591814	96.45	2827597	96.29
2	6.122	830361	3.55	109094	3.71

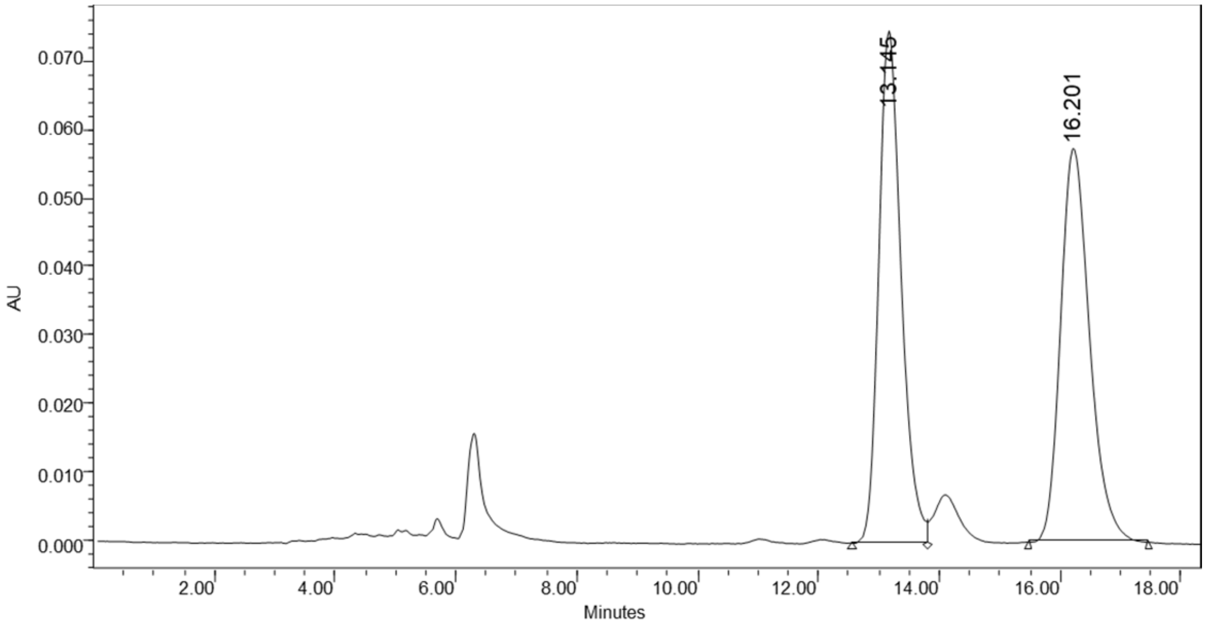

	RT (min)	Area (V*sec)	% Area	Height (V)	% Height
1	13.145	1936333	50.03	74868	56.53
2	16.201	1933646	49.97	57580	43.47

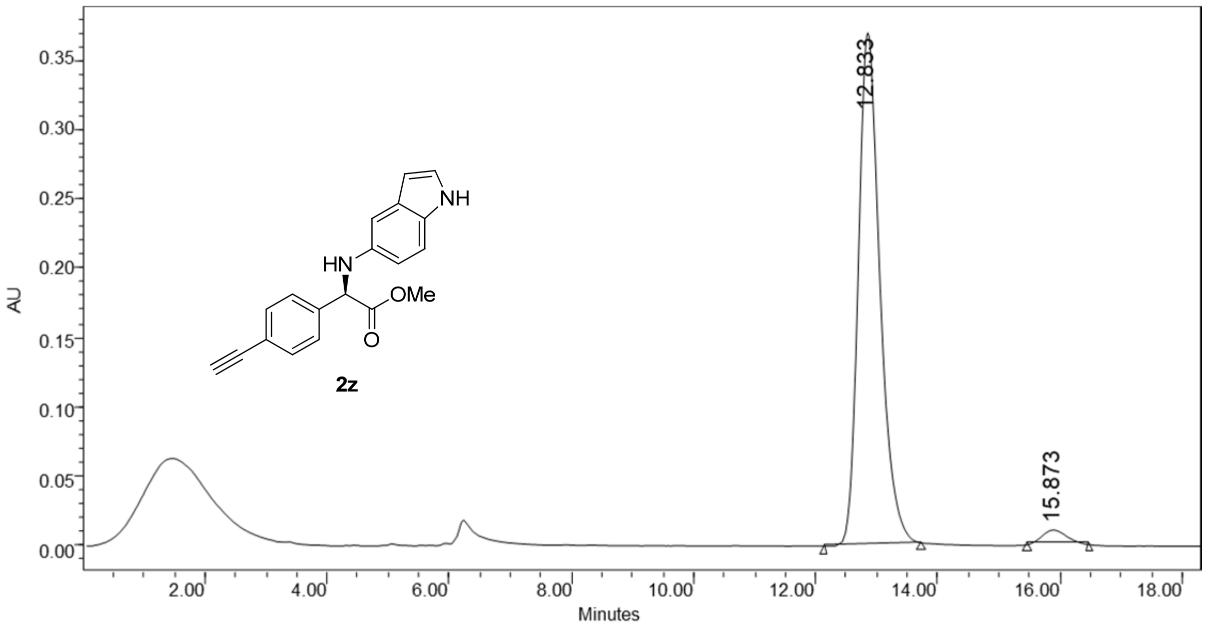

	RT (min)	Area (V*sec)	% Area	Height (V)	% Height
1	12.833	9088867	96.75	369910	97.22
2	15.873	305685	3.25	10588	2.78

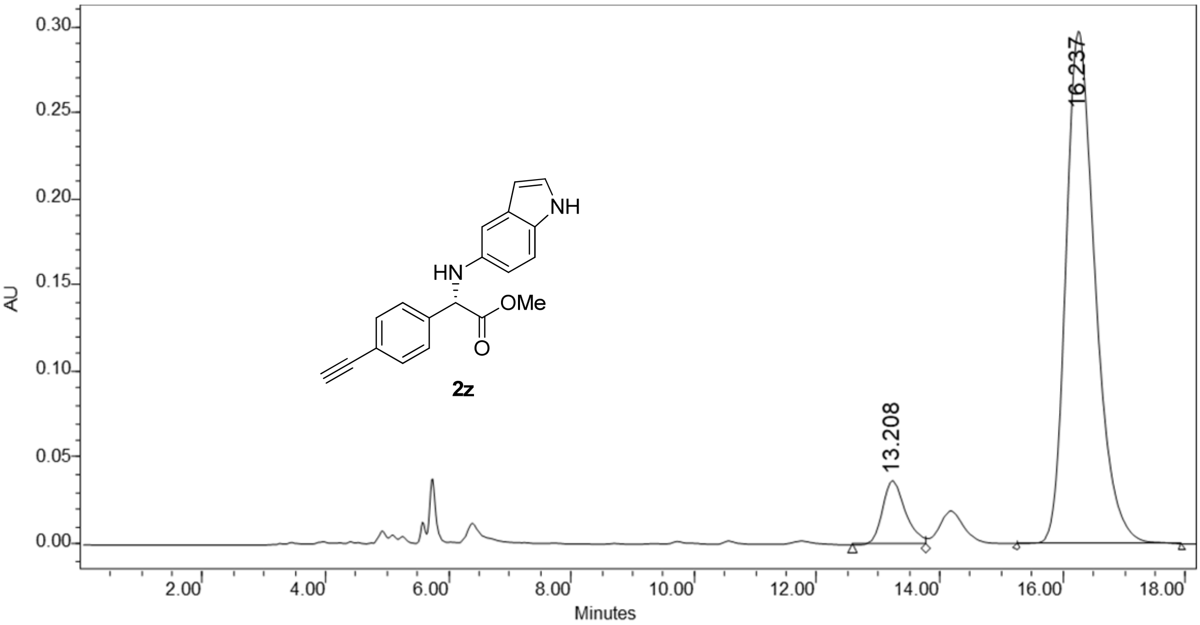

	RT (min)	Area (V*sec)	% Area	Height (V)	% Height
1	13.208	975940	9.00	37098	11.10
2	16.237	9870006	91.00	297233	88.90

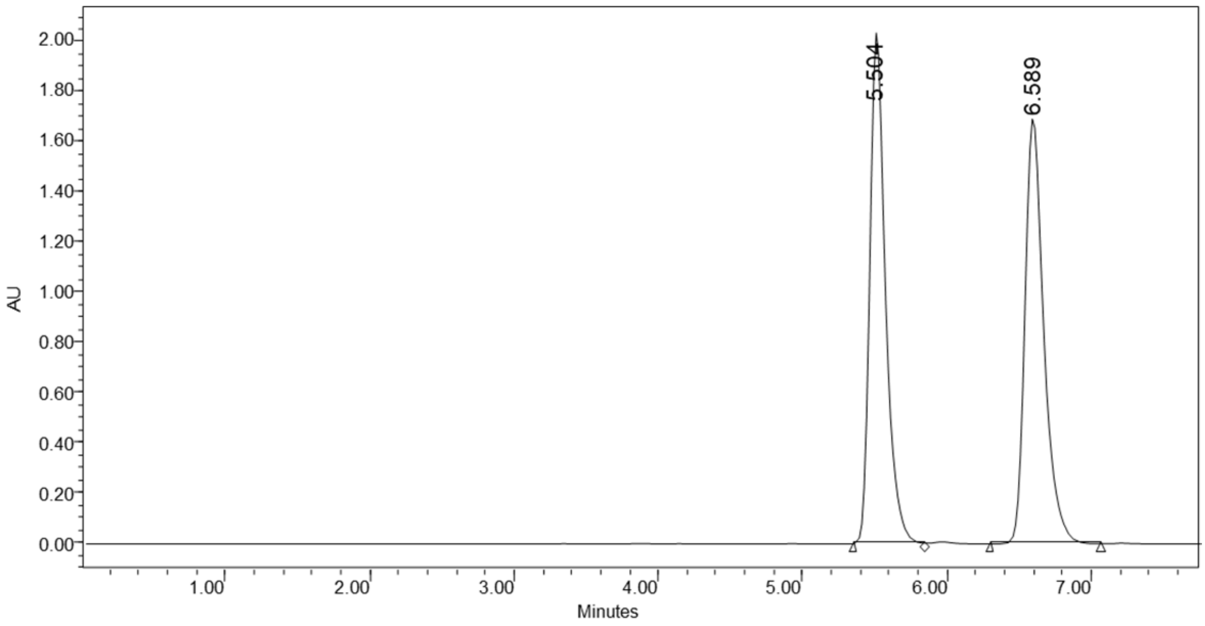

	RT (min)	Area (V*sec)	% Area	Height (V)	% Height
1	5.504	15353769	49.85	2032832	54.49
2	6.589	15446582	50.15	1697888	45.51

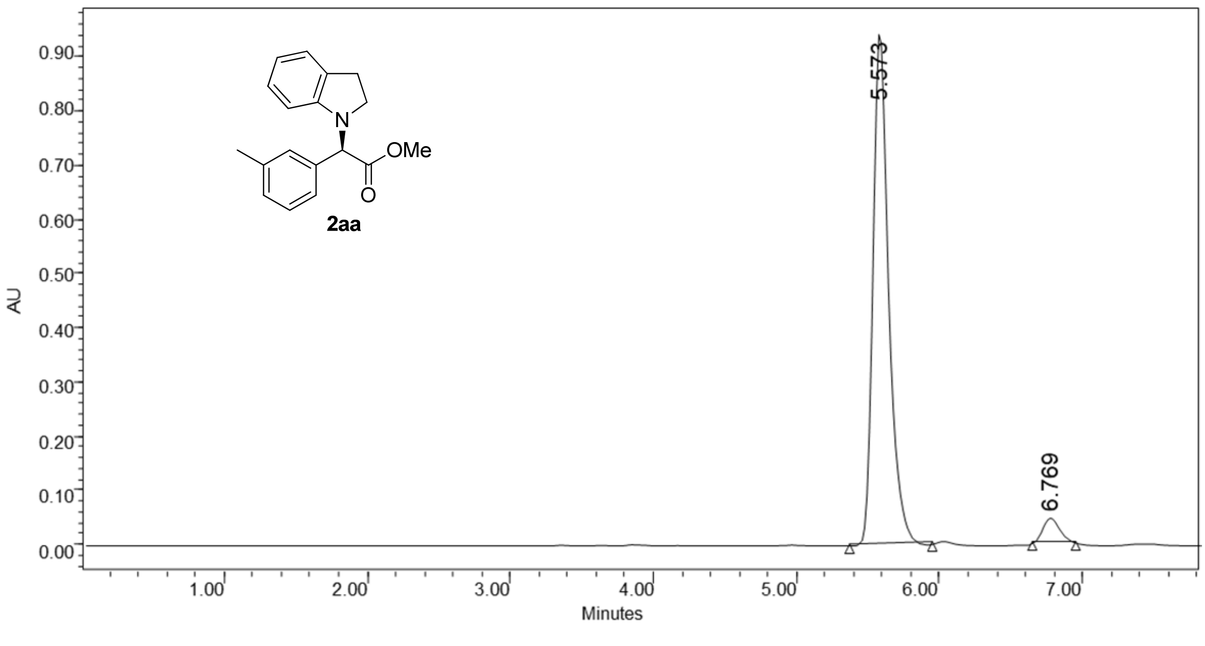

	RT (min)	Area (V*sec)	% Area	Height (V)	% Height
1	5.573	7169303	94.99	944799	95.33
2	6.769	378016	5.01	46303	4.67

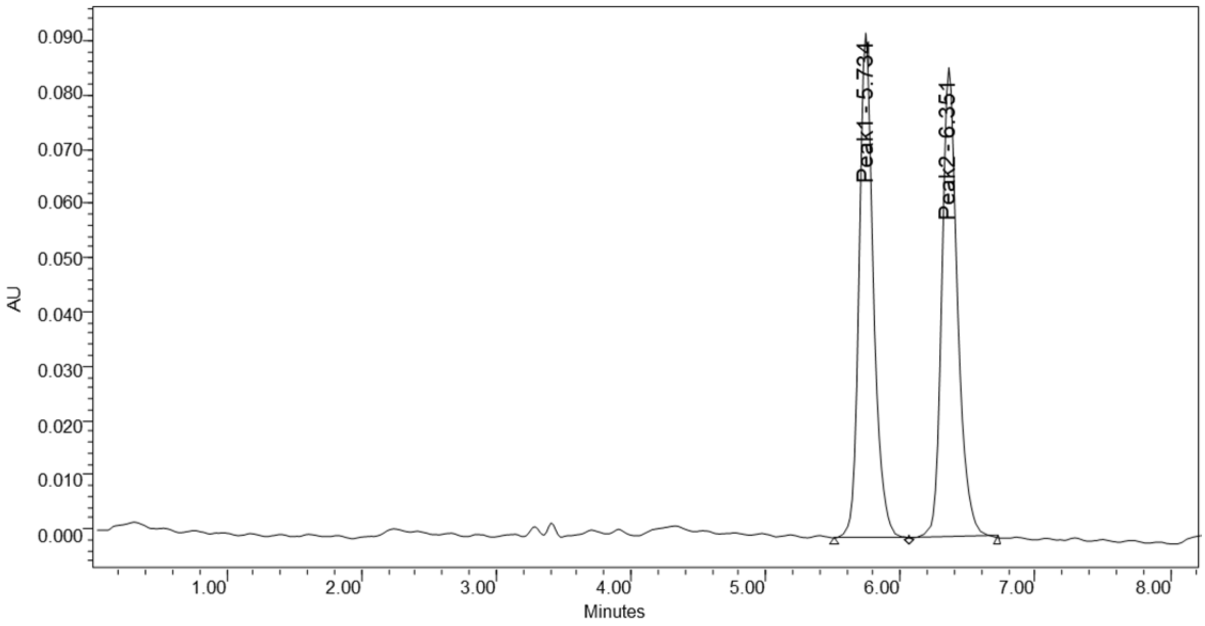

	Peak Name	RT (min)	Area (V*sec)	% Area	Height (V)	% Height
1	Peak1	5.734	686777	49.32	92468	51.73
2	Peak2	6.351	705630	50.68	86300	48.27

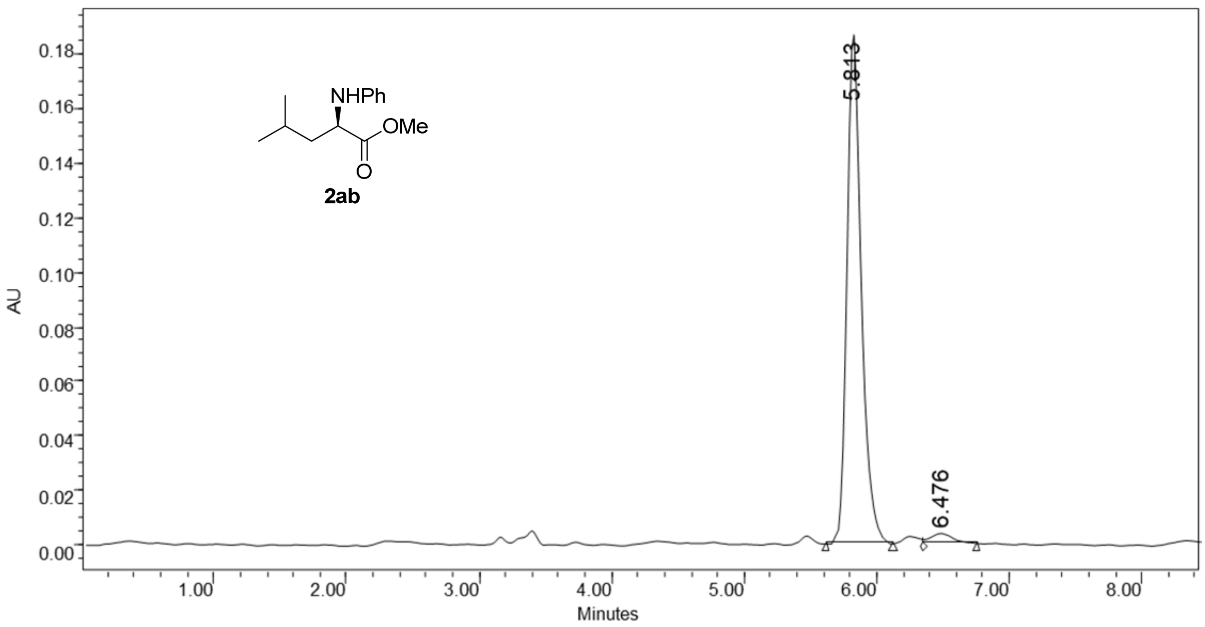

	RT (min)	Area (V*sec)	% Area	Height (V)	% Height
1	5.813	1419492	96.96	186701	98.00
2	6.476	44523	3.04	3801	2.00

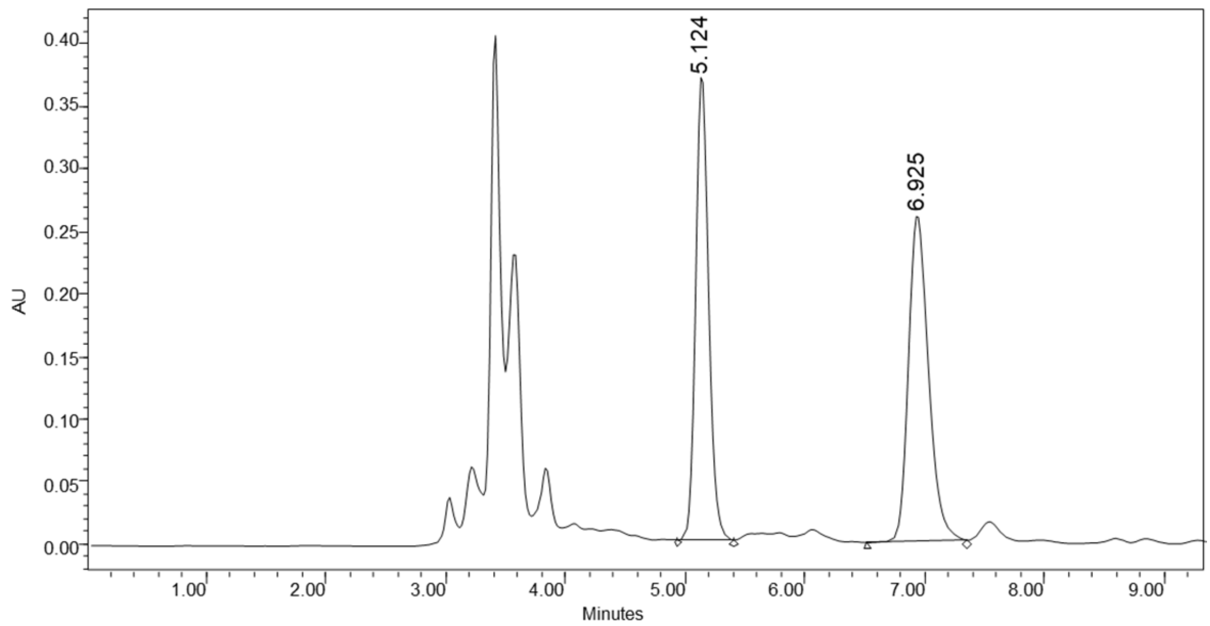

	RT (min)	Area (V*sec)	% Area	Height (V)	% Height
1	5.124	2797900	47.77	371908	58.72
2	6.925	3058724	52.23	261405	41.28

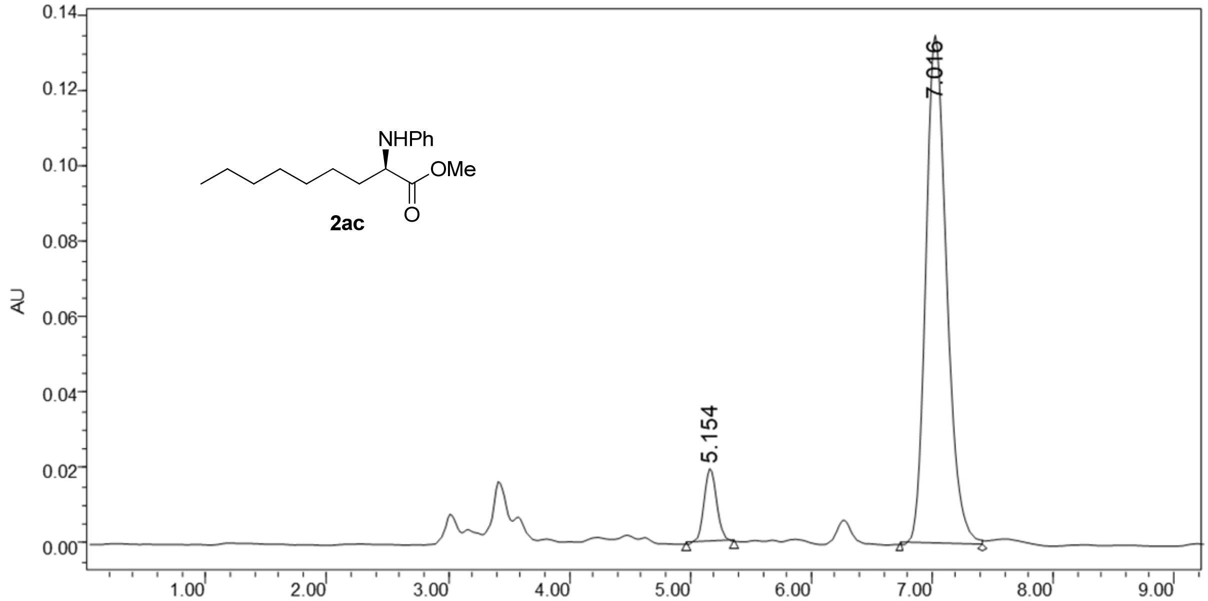

	RT (min)	Area (V*sec)	% Area	Height (V)	% Height
1	5.154	145128	8.19	19719	12.73
2	7.016	1627178	91.81	135157	87.27

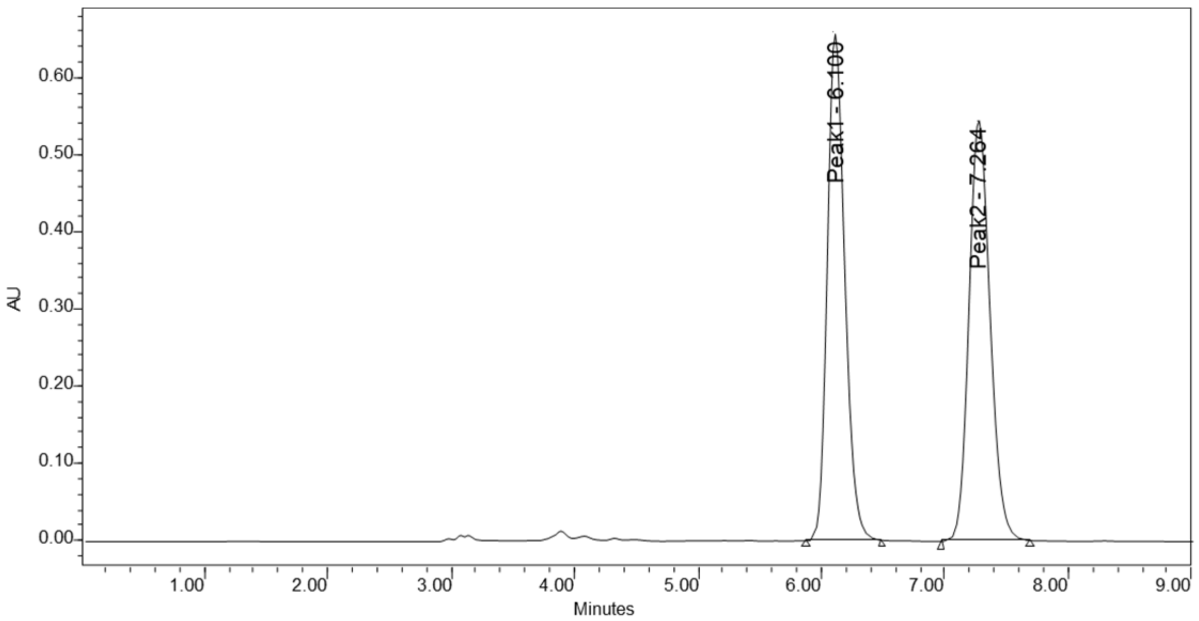

	Peak Name	RT (min)	Area (V*sec)	% Area	Height (V)	% Height
1	Peak1	6.100	6451829	49.88	655240	54.62
2	Peak2	7.264	6482275	50.12	544446	45.38

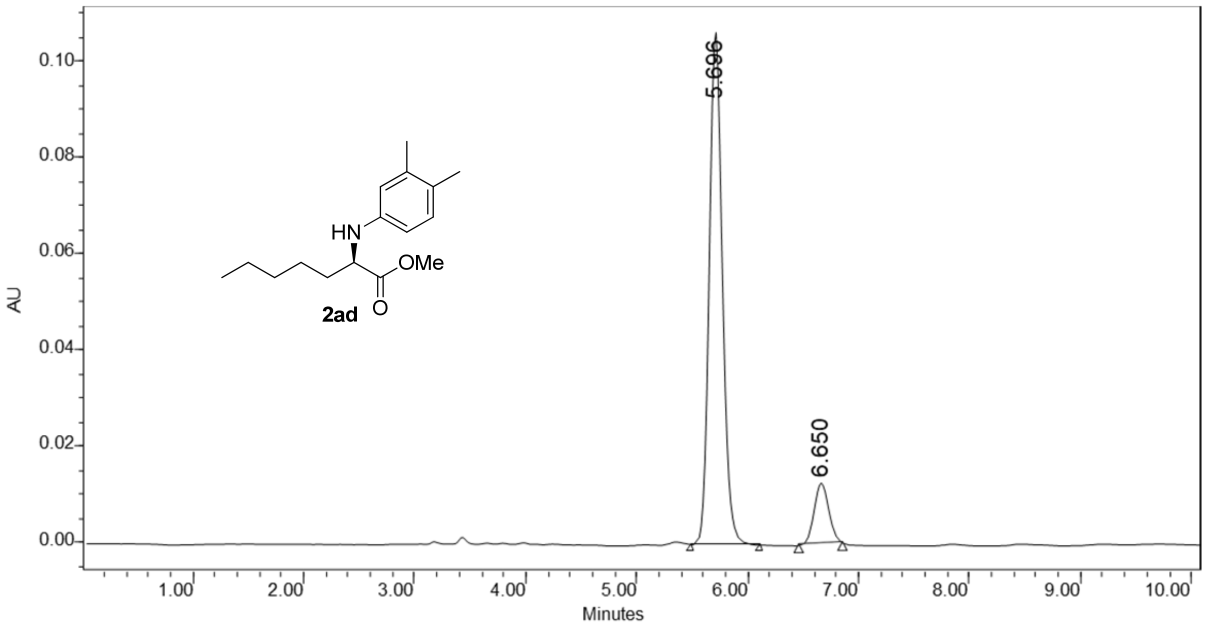

	RT (min)	Area (V*sec)	% Area	Height (V)	% Height
1	5.696	873784	88.11	106428	89.28
2	6.650	117885	11.89	12775	10.72

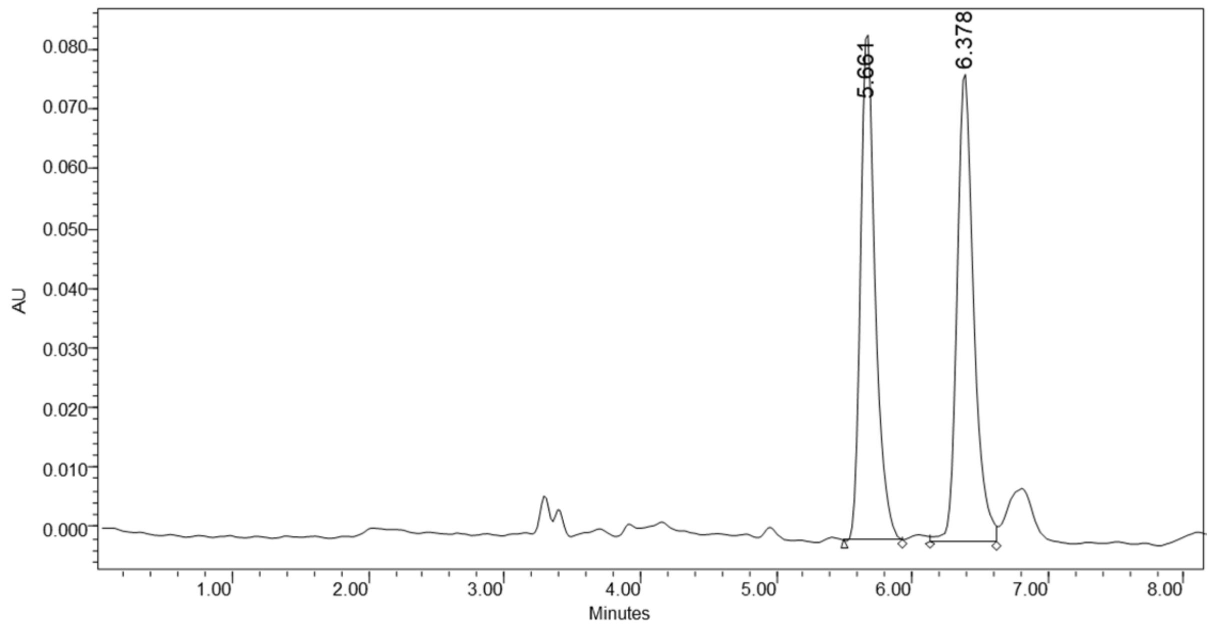

	RT (min)	Area (V*sec)	% Area	Height (V)	% Height
1	5.661	642314	48.76	85023	51.97
2	6.378	674885	51.24	78564	48.03

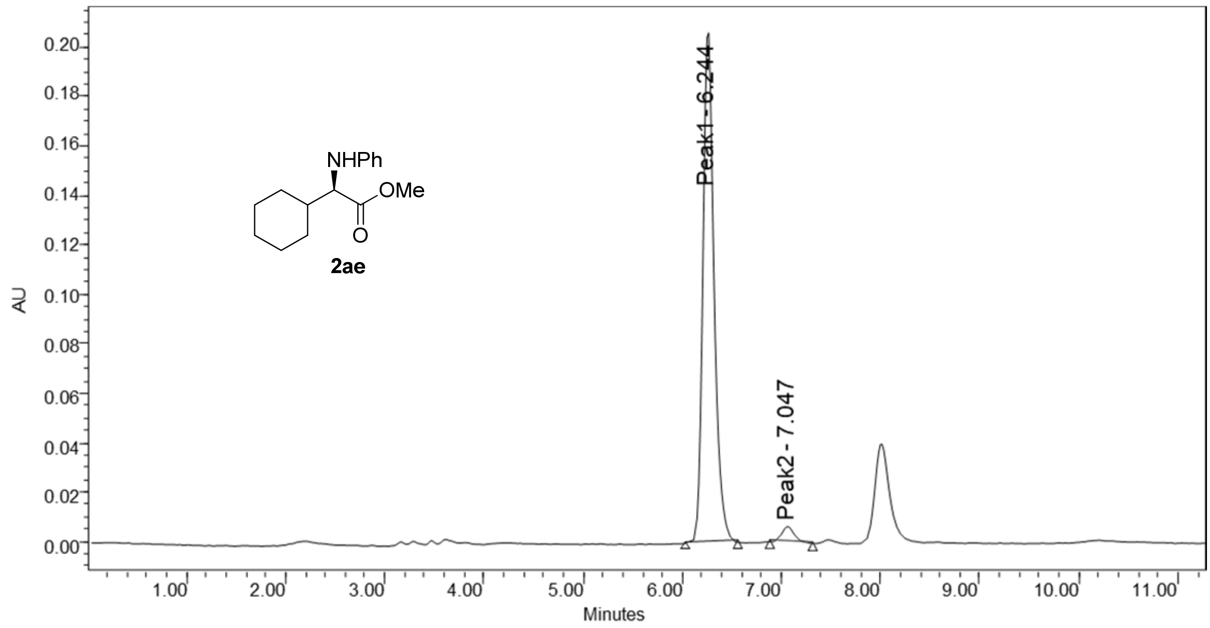

	Peak Name	RT (min)	Area (V*sec)	% Area	Height (V)	% Height
1	Peak1	6.244	1617065	96.67	206468	97.00
2	Peak2	7.047	55787	3.33	6378	3.00

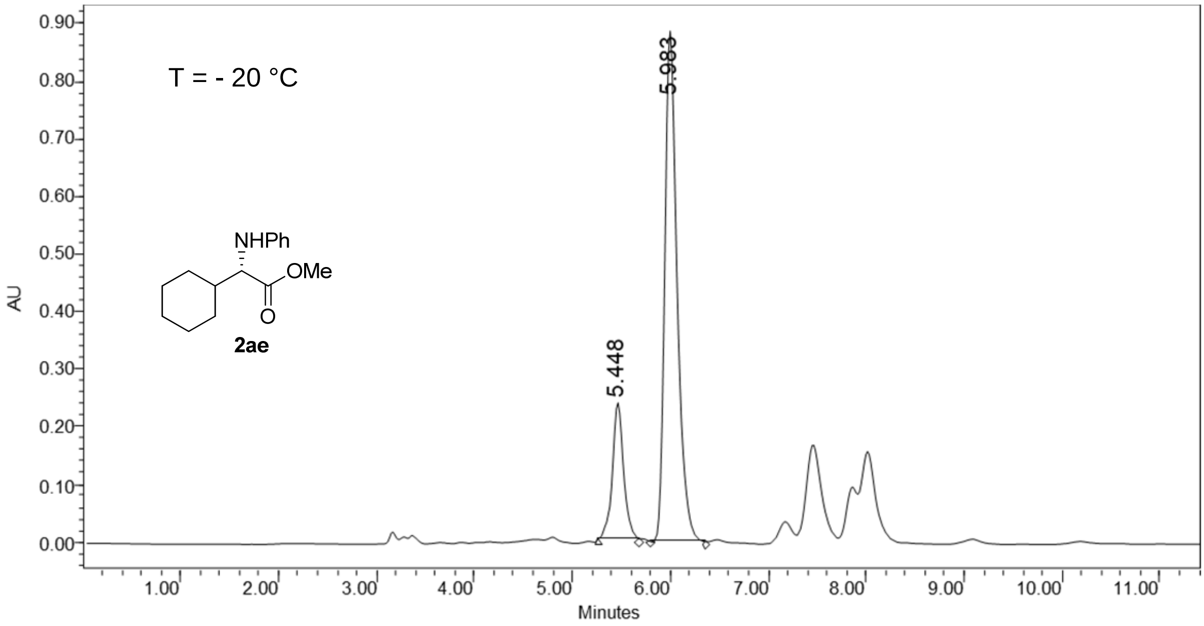

	RT (min)	Area (V*sec)	% Area	Height (V)	% Height
1	5.448	1905036	19.77	236787	21.15
2	5.983	7730984	80.23	882931	78.85

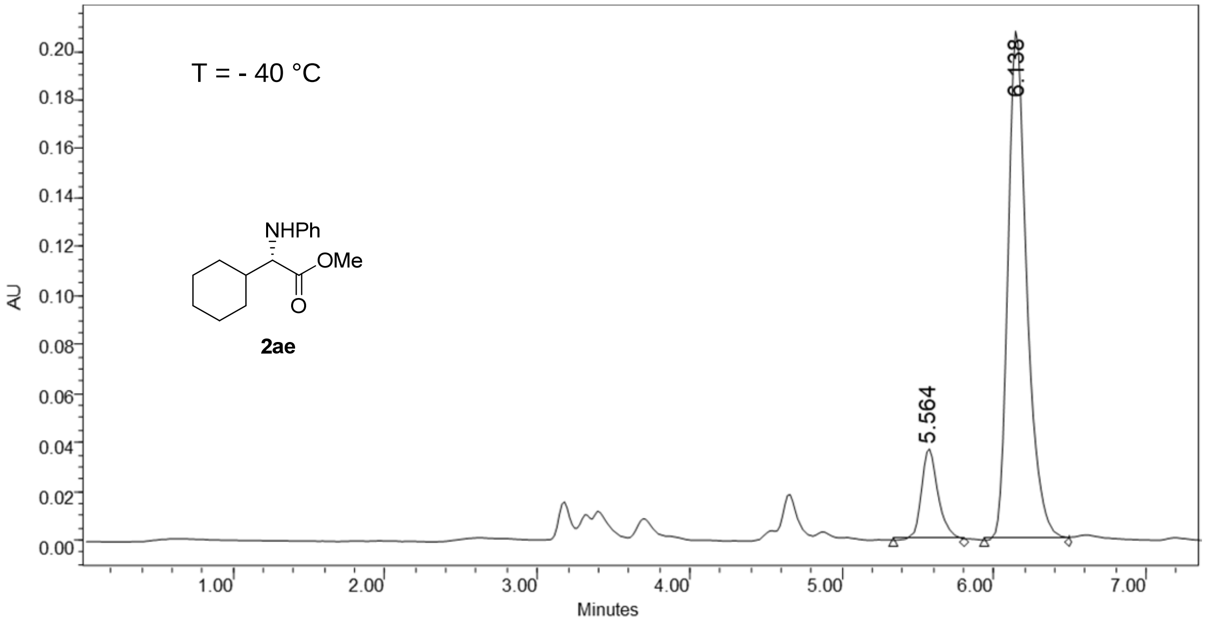

	RT (min)	Area (V*sec)	% Area	Height (V)	% Height
1	5.564	288299	13.74	37185	15.16
2	6.138	1809510	86.26	208169	84.84

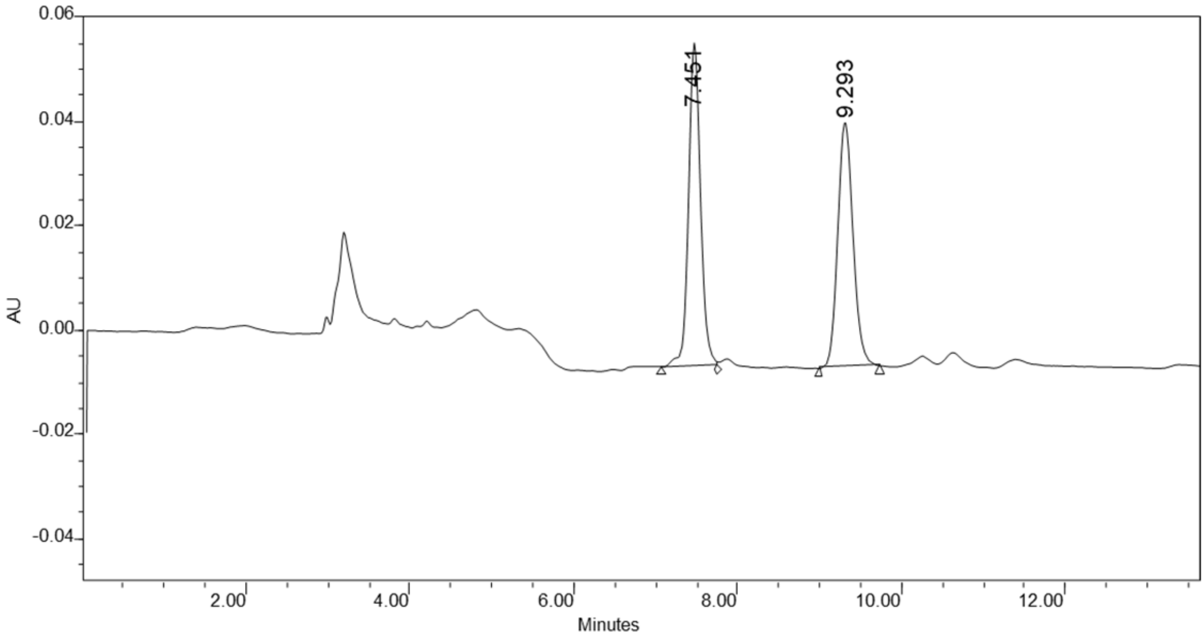

	RT (min)	Area (V*sec)	% Area	Height (V)	% Height
1	7.451	634950	50.93	62011	56.87
2	9.293	611734	49.07	47030	43.13

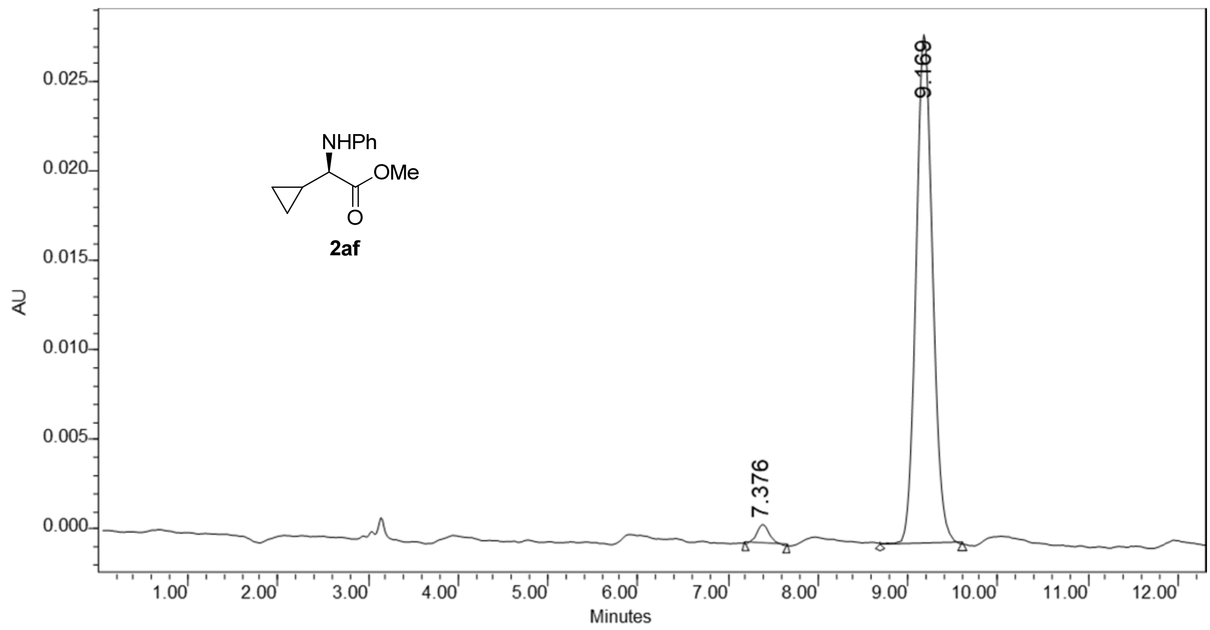

	RT (min)	Area (V*sec)	% Area	Height (V)	% Height
1	7.376	11359	3.00	1117	3.77
2	9.169	367316	97.00	28477	96.23

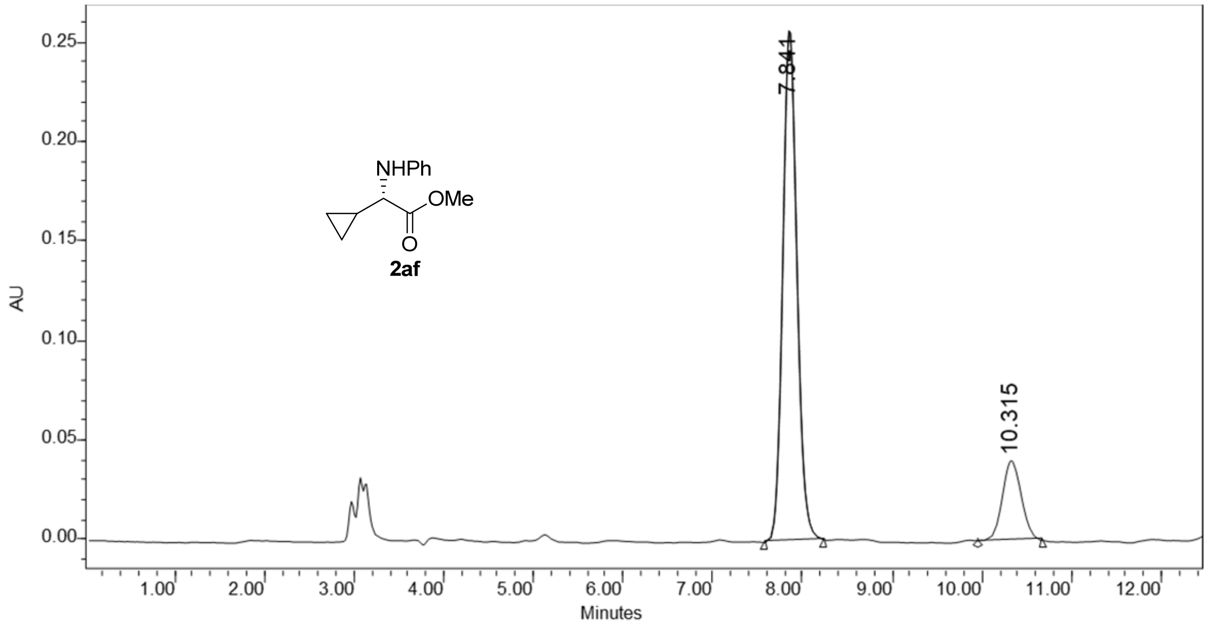

	RT (min)	Area (V*sec)	% Area	Height (V)	% Height
1	7.841	2843578	82.42	257725	86.41
2	10.315	606579	17.58	40527	13.59

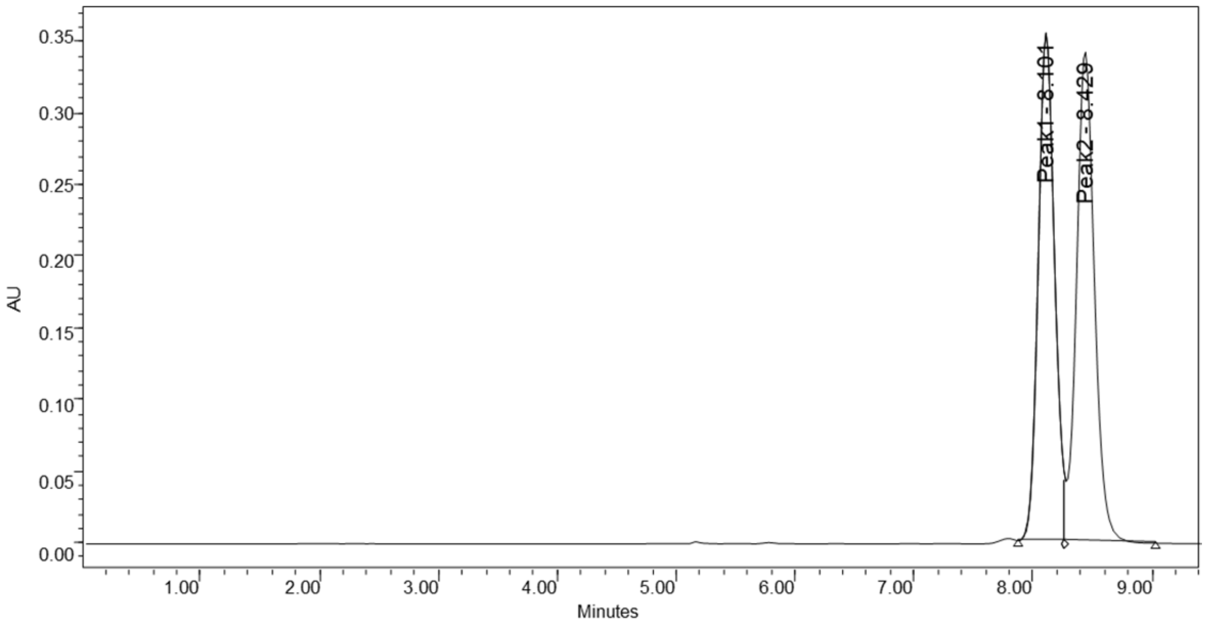

	Peak Name	RT (min)	Area (V*sec)	% Area	Height (V)	% Height
1	Peak1	8.101	3406150	48.75	353963	50.88
2	Peak2	8.429	3580787	51.25	341725	49.12

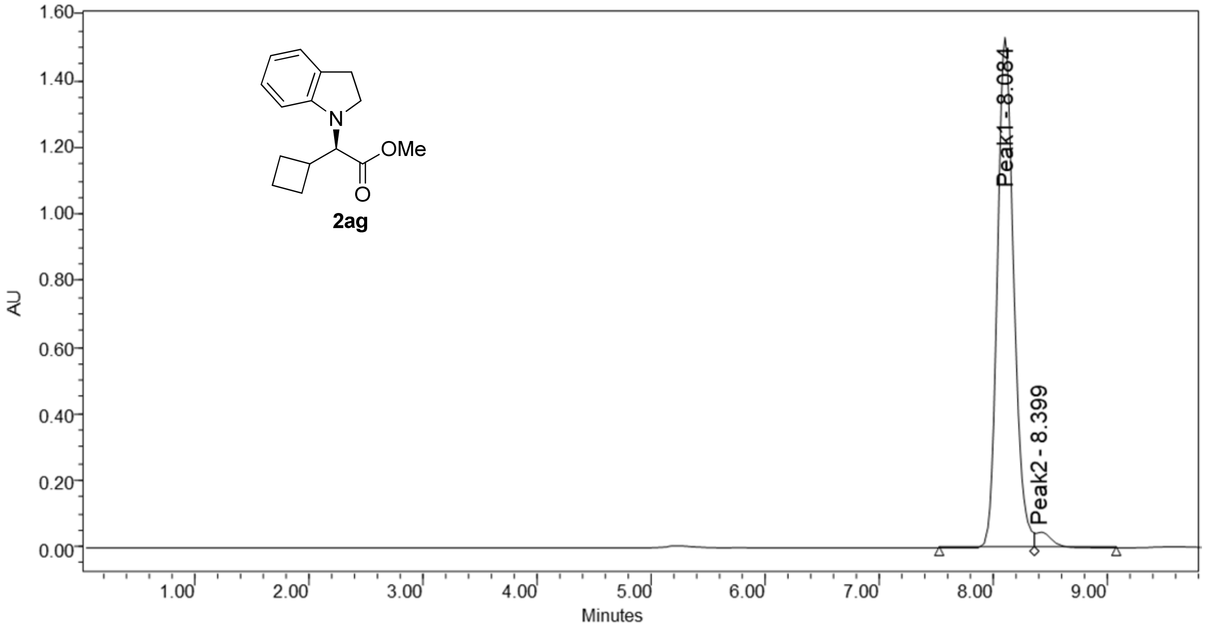

	Peak Name	RT (min)	Area (V*sec)	% Area	Height (V)	% Height
1	Peak1	8.084	15298621	97.08	1530545	97.02
2	Peak2	8.399	459828	2.92	47025	2.98

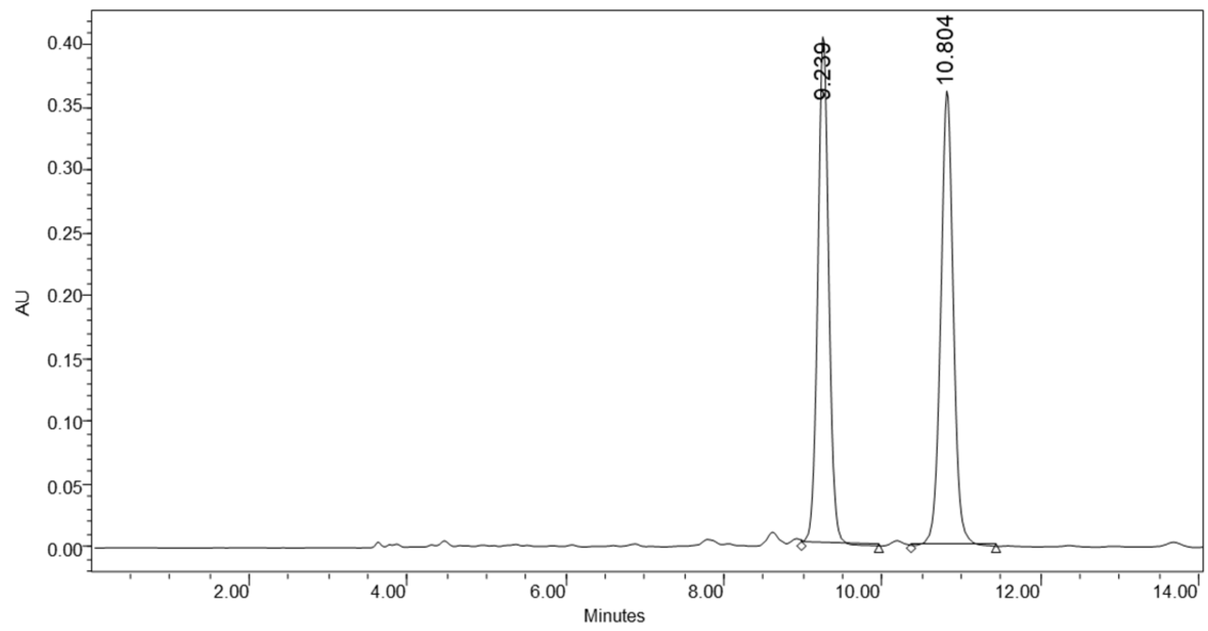

	RT (min)	Area (V*sec)	% Area	Height (V)	% Height
1	9.239	3922812	49.30	405973	52.78
2	10.804	4034794	50.70	363206	47.22

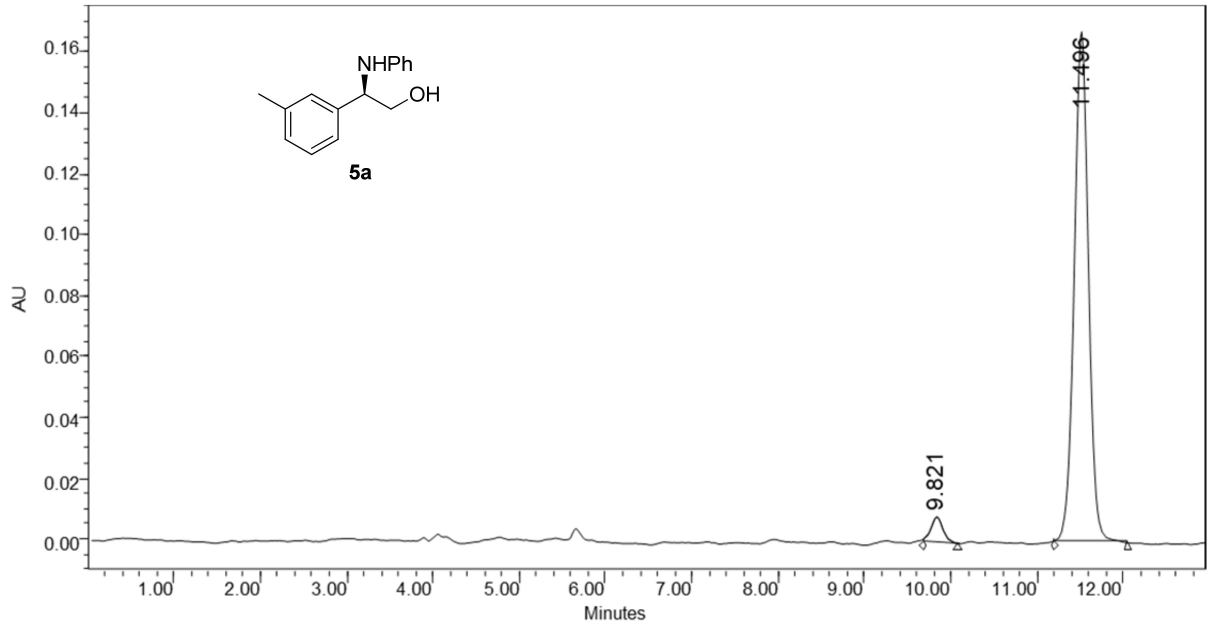

	RT (min)	Area (V*sec)	% Area	Height (V)	% Height
1	9.821	85691	4.25	8653	4.91
2	11.496	1930839	95.75	167676	95.09

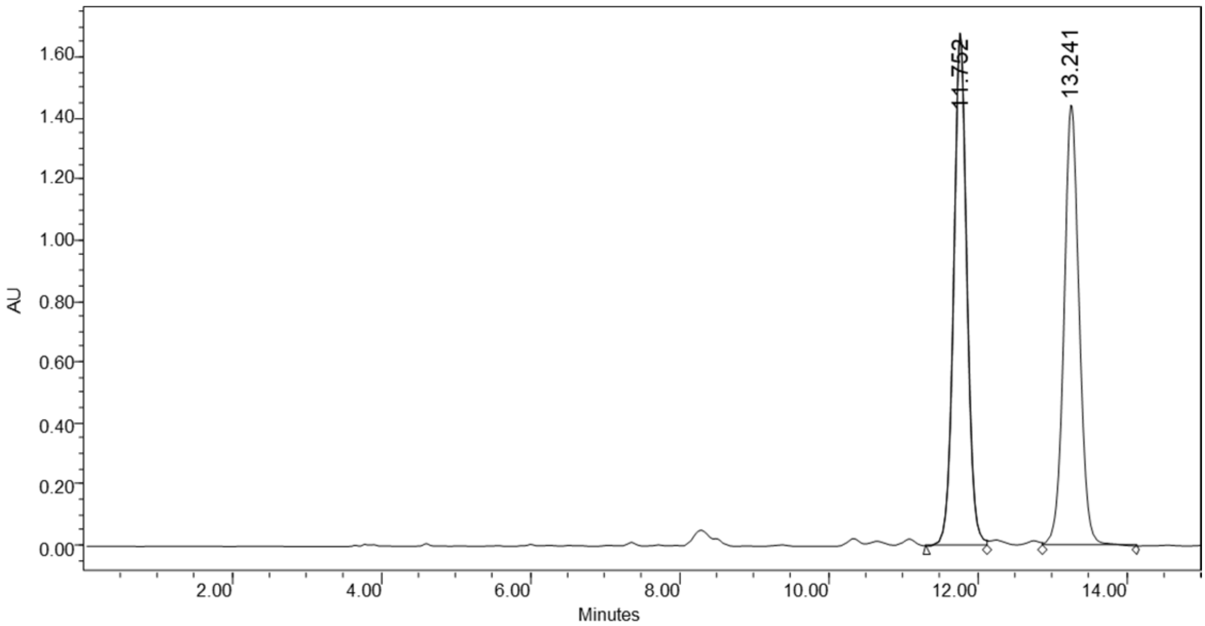

	RT (min)	Area (V*sec)	% Area	Height (V)	% Height
1	11.752	20786512	50.14	1678453	53.68
2	13.241	20668710	49.86	1448159	46.32

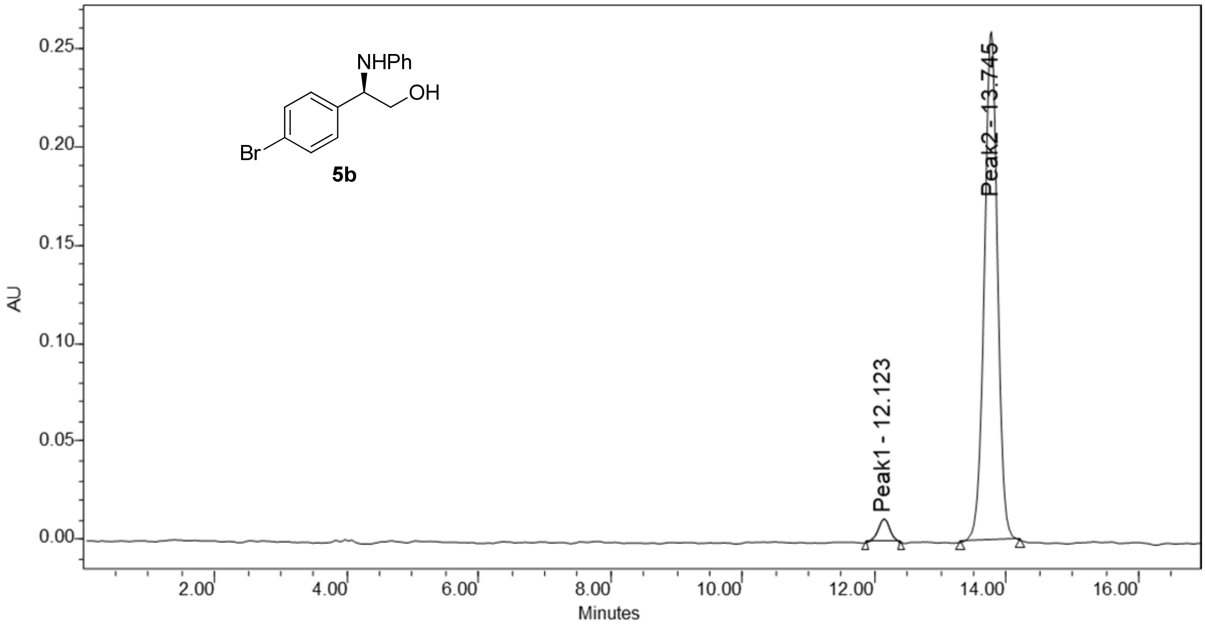

	Peak Name	RT (min)	Area (V*sec)	% Area	Height (V)	% Height
1	Peak1	12.123	152508	4.04	12119	4.46
2	Peak2	13.745	3618545	95.96	259607	95.54

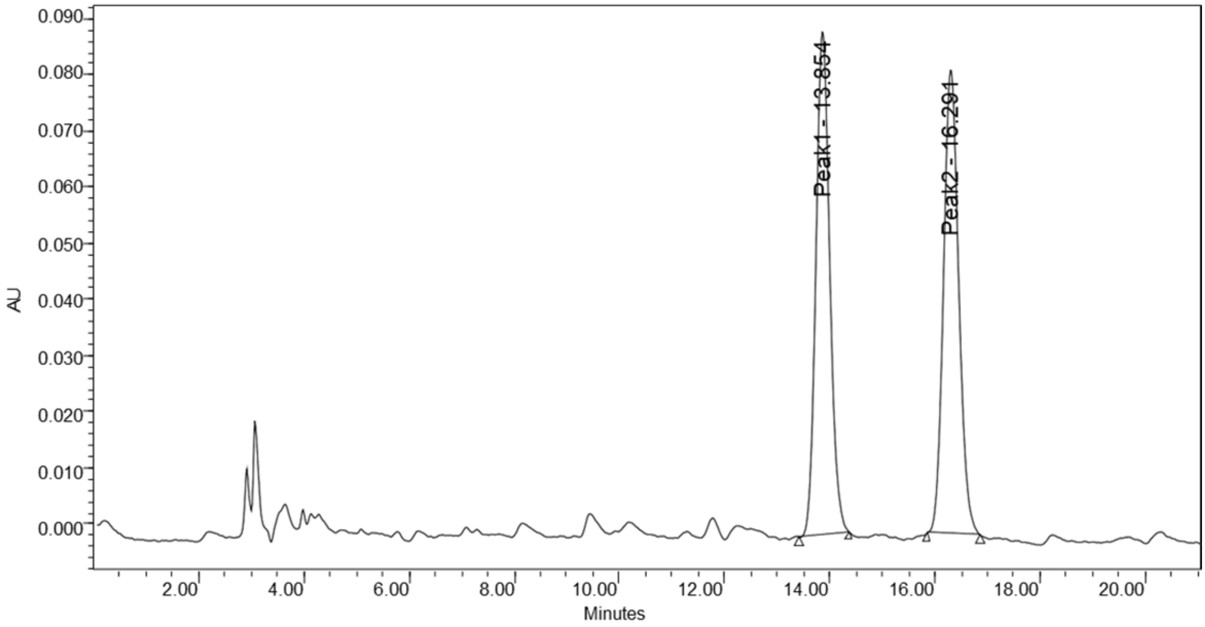

	Peak Name	RT (min)	Area (V*sec)	% Area	Height (V)	% Height
1	Peak1	13.854	1637433	49.59	89585	51.99
2	Peak2	16.291	1664352	50.41	82720	48.01

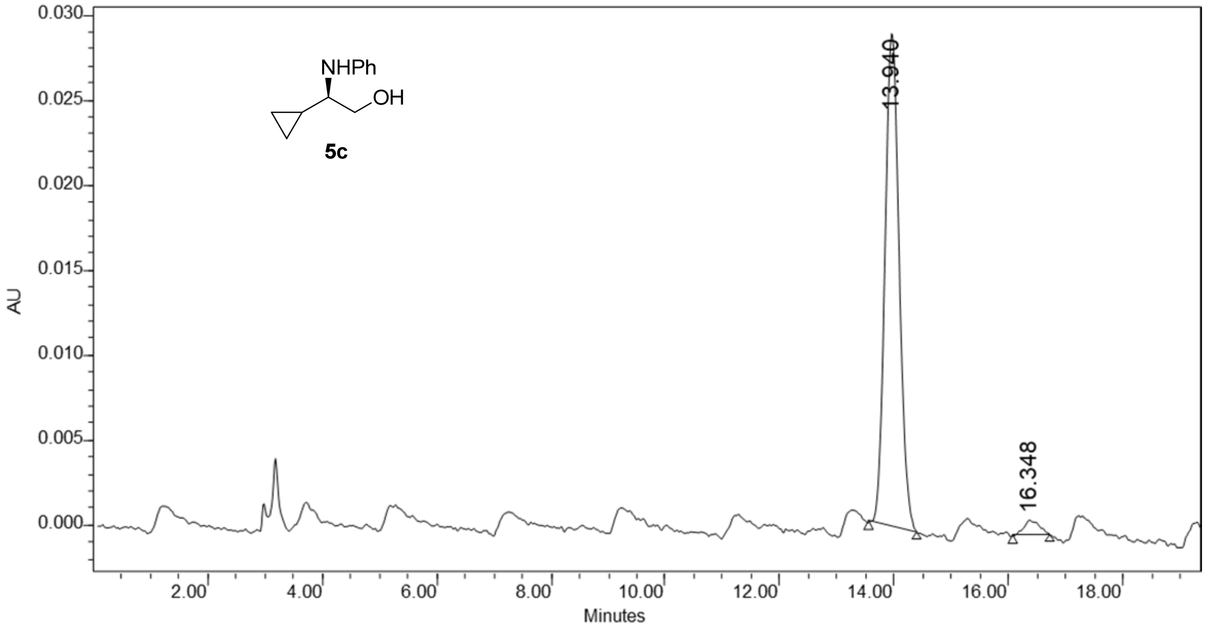

	RT (min)	Area (V*sec)	% Area	Height (V)	% Height
1	13.940	501402	96.46	29047	96.91
2	16.348	18399	3.54	925	3.09

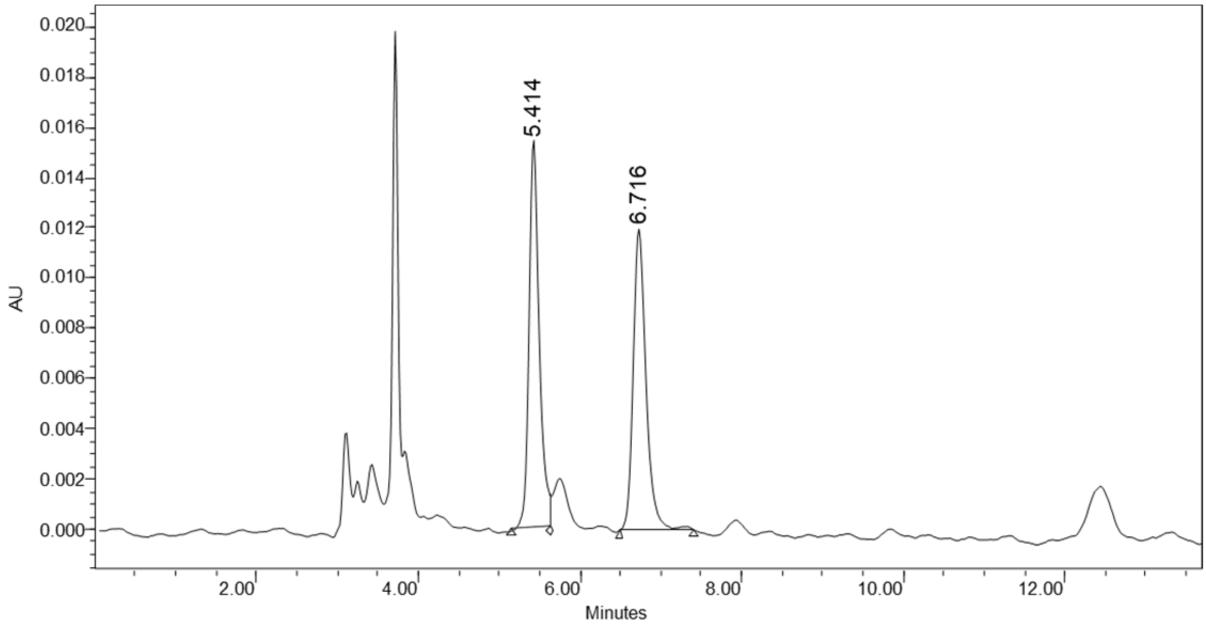

	RT (min)	Area (V*sec)	% Area	Height (V)	% Height
1	5.414	137307	50.90	15444	56.32
2	6.716	132435	49.10	11976	43.68

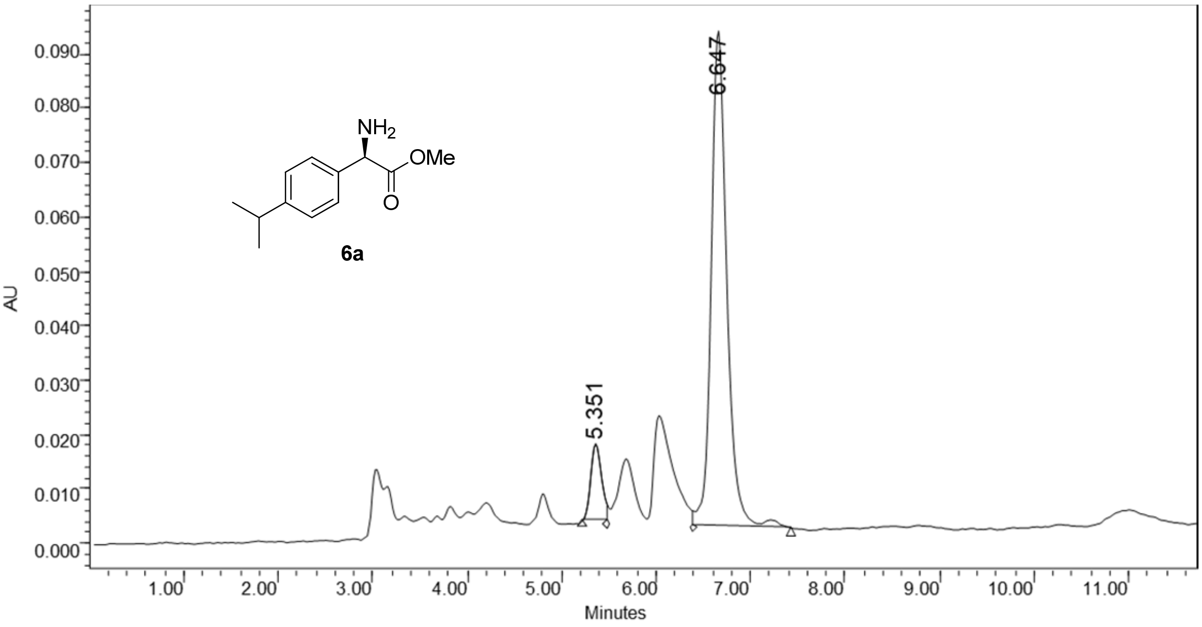

	RT (min)	Area (V*sec)	% Area	Height (V)	% Height
1	5.351	115911	10.00	14205	13.52
2	6.647	1042843	90.00	90890	86.48

DFT Calculations

Computational methods

Conformational space has been explored using CREST[17] meta-dynamic calculations at the semi-empiric level GFN2-xTB theory implicitly including the toluene solvation using the ALPB[18] theory, using default cut-off and lengths parameters. The conformers resulting from this calculations were then grouped thanks to the PCA clustering methodology.[19] The best cluster family was then optimised using Gaussian 16.A03[20] suite at B3LYP/6-31G(d) level. The toluene solvation was considered in an implicit way using the CPCM model. Energy was then corrected with a single point calculation[21] using the same functional but with the larger 6-311++G(2d,2p) basis set. Frequencies were then dumped using the mRRHO[22] theory, the thermal correction recalculated at 253K and the enantiomeric excess estimation was performed thanks the GoodVibes[23] script (version 3.2). Geometries and vibrational analysis were visualised using ChimeraX.[24]

Results

The conformational space was preliminary reduced by omitting the vinyl group of the catalyst and the TBHP have been used instead of the CHP. The reaction has been divided in two separate steps: i) the addition of the TBHP to the double C-C bond; ii) the S_N2 intramolecular reaction that result in the closure to the epoxide. The first step's reactive complexes (**Pre_complex**) show a strong interaction between the sulphone and the thiourea residue of the catalyst, which is characteristic for this type of reaction conditions. Furtherly, it's possible to notice that in the transition state (**TS1**) geometry the ternary amine of the quinuclidinic moiety is able to deprotonate the TBHP, allowing the peroxidic group to attack

---

[17] Grimme, S.; Bohle, F.; Hansen, A.; Pracht, P.; Spicher, S.; Stahn, M. *J. Phys. Chem. A* **2021**, *125*, 4039.
[18] Ehlert, S.; Stahn, M.; Spicher, S.; Grimme, S. *J. Chem. Theory Comput.* **2021**, *17*, 4250.
[19] Shao, J.; Tanner, S. W.; Thompson, N.; Cheatham, T. E. *J. Chem. Theory Comput.* **2007**, 3, 2312.
[20] M. J. Frisch; G. W. Trucks; H. B. Schlegel,; G. E. Scuseria; M. A. Robb; J. R. Cheeseman; G. Scalmani; V. Barone; G. A. Petersson; H. Nakatsuji; X. Li; M. Caricato; A. V. Marenich; J. Bloino; B. G. Janesko; R. Gomperts; B. Mennucci; H. P. Hratchian; J. V. Ortiz; A. F. Izmaylov; J. L. Sonnenberg; D. Williams-Young; F. Ding; F. Lipparini; F. Egidi; J. Goings; B. Peng; A. Petrone; T. Henderson; D. Ranasinghe; V. G. Zakrzewski; J. Gao; N. Rega; G. Zheng; W. Liang; M. Hada; M. Ehara; K. Toyota; R. Fukuda; J. Hasegawa; M. Ishida; T. Nakajima; Y. Honda; O. Kitao; H. Nakai; T. Vreven; K. Throssell; J. A. Montgomery; Jr., J. E. Peralta; F. Ogliaro; M. J. Bearpark; J. J. Heyd; E. N. Brothers; K. N. Kudin; V. N. Staroverov; T. A. Keith; R. Kobayashi; J. Normand; K. Raghavachari; A. P. Rendell; J. C. Burant; S. S. Iyengar; J. Tomasi; M. Cossi; J. M. Millam; M. Klene; C. Adamo; R. Cammi; J. W. Ochterski; R. L. Martin; K. Morokuma; O. Farkas; J. B. Foresman; D. J. Fox. Gaussian 16, Revision A.03. Gaussian, Inc. **2016**.
[21] Simón, L.; Goodman, J. M. *Org. Biomol. Chem.* **2011**, *9*, 689.
[22] Pracht, P.; Grimme, S. *Chem. Sci.* **2021**, *12*, 6551.
[23] Luchini, G.; Alegre-Requena, J. V.; Funes-Ardoiz, I.; Paton, R. S. *GoodVibes: Automated Thermochemistry for Heterogeneous Computational Chemistry Data*. F1000Research April 24, **2020**. https://doi.org/10.12688/f1000research.22758.1.
[24] Pettersen, E. F.; Goddard, T. D.; Huang, C. C.; Couch, G. S.; Greenblatt, D. M.; Meng, E. C.; Ferrin, T. E. *J. Comput. Chem.* **2004**, *25*, 1605.

the reagent. After the addition TS, a relaxed optimization (following the reaction coordinate dictated by the negative normal mode in **TS1**) was carried out to find the reaction intermediates.

Attack on the **Re** face	Attack on the **Si** face
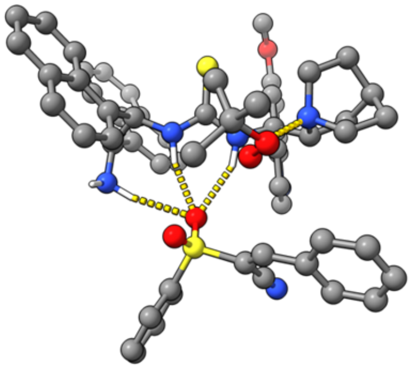 **PRE-complex R**	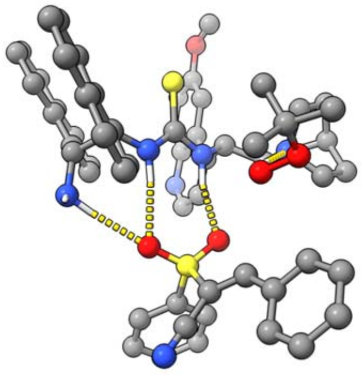 **PRE-complex S**
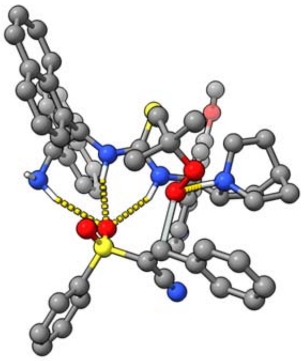 **TS1-R**	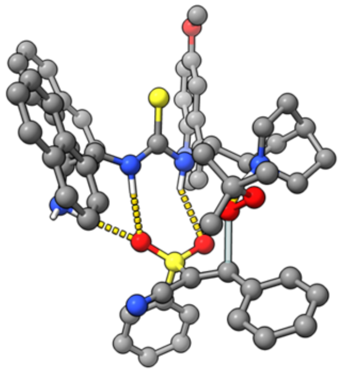 **TS1-S**
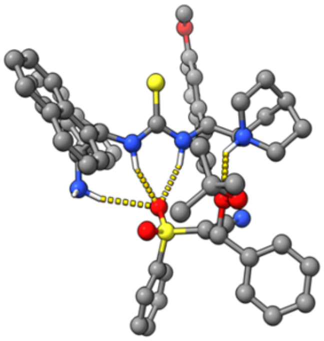 **Intermediate *R***	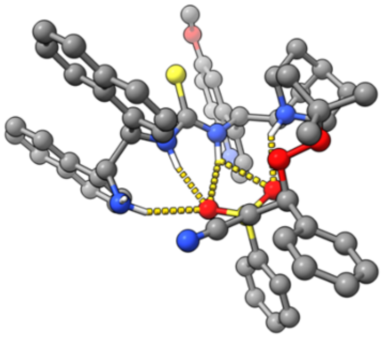 **Intermediate *S***

Figure S1. 3D representations of the optimized geometries for the addition step with TBHP. The electrophile results from condensation of phenylsulfonylacetonitrile with benzaldehyde. In catalyst **4** the vinyl moiety was removed to reduce computational times.

The following step is the closure of the epoxide ring with subsequential leaving of *tert*-butyl alcohol. The transition state geometries for the ring closure (**TS2**) are highly constrained and it is possible to observe the importance of the NH^+ pointing towards the peroxidic residue, and the hydrogen bond mesh formed in the middle of the catalytic pocket. This is the enantioselective and rate-determining step, in which the two centres generated by the reaction are locked. By relaxed optimization toward the reagents starting from **TS2**, it was checked

that the starting geometries for these TS corresponded to that of the reaction intermediated obtained in the first step after the addition of TBHP, with tiny differences in terms of energy and conformations. The calculated enantiomeric excess (**e.e.**) is 98.8% at 253°K, which corresponds to a difference of free energy of the two transition states of 2.55 kcal mol^{-1} (**TS2-SS** vs **TS2-RR**). This value can be compared to the experimental e.e. = 60% at the same temperature (exp. $\Delta\Delta G^{\ddagger}$=0.81 kcal mol^{-1}).

Ring Closure of the ***R*** intermediate	Ring Closure of the ***S*** intermediate
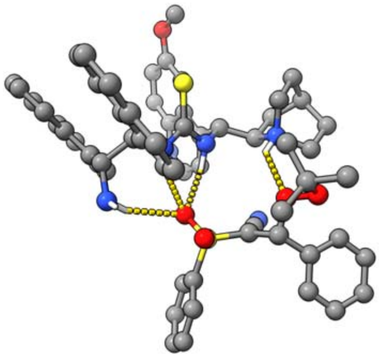<br>**Intermediate *R***	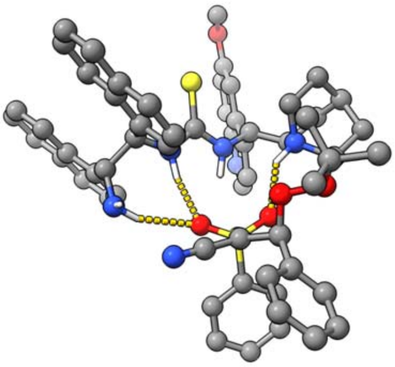<br>**Intermediate *S***
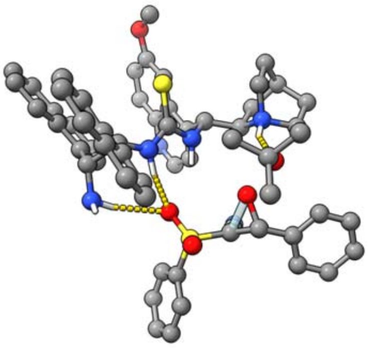<br>**TS2-RR**	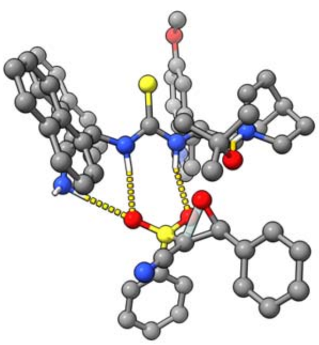<br>**TS2-SS**
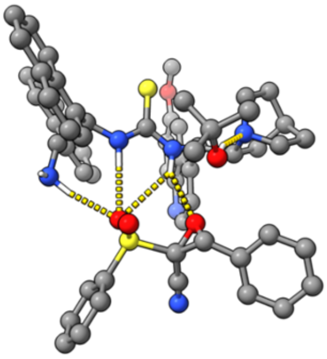<br>***R,R* epoxide**	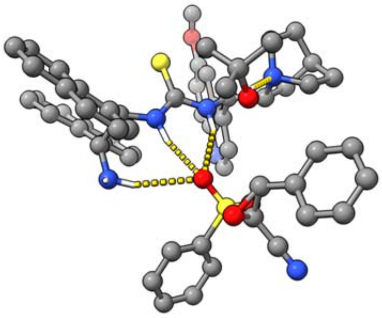<br>***S,S* epoxide**

Figure S2. 3D representations of the optimized geometries for the ring-closure step.

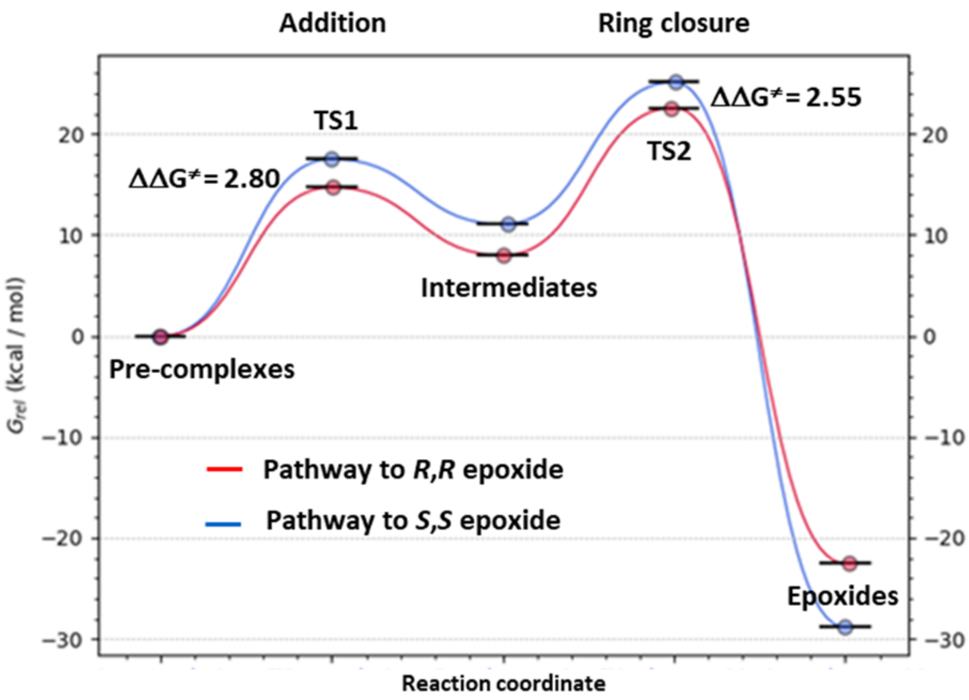

Figure S3. Reaction pathways for the model reaction calculated at the B3LYP/6-311++G(2d,2p)-CPCM[toluene]//B3LYP/6-31G(d)-CPCM[toluene] level, at 253K. Energies in kcal/mol.

After the geometries of the four TSs were determined, 1-naphthylsulphone and the vinyl branch were added to the calculation in order to simulate the best results experimentally obtained. Also in this pathway, the TSs with the highest energy remain the epoxide closure, but the $\Delta\Delta G^{\ddagger}$ between the two TSs is lowered to 2.41 kcal/mol, which corresponds to an *ee* value of 98.3% 253°K.

Table S3 Absolute and relative free energy corrected of the four TS including 1-naphtylsulphone and vinyl group of the catalyst.

ΔG [Eh]	**Addition to Re face** (a.u.)	**Addition to Si face** (a.u.)	$\Delta\Delta G^{\ddagger}$ [kcal/mol]
**TS1 (addition)**	-4055.554397	-4055.549696	2.95
	**Ring closure to *R,R epoxide***	**Ring closure to *S,S epoxide***	
**TS2 (ring closure)**	-4055.545125	-4055.541285	2.41

Structure	E_SPC	E	ZPE	H_SPC	T.S	T.qh-S	G(T)_SPC	qh-G(T)_SPC	im freq
pre_complex_R	-3825.578869	-3824.603643	1.088643	-3824.437992	0.142957	0.125051	-3824.580949	-3824.563043	
pre_complex_S	-3825.578870	-3824.603643	1.088643	-3824.437993	0.142944	0.125045	-3824.580937	-3824.563038	
TS1-R	-3825.557503	-3824.583750	1.087806	-3824.418914	0.135352	0.120712	-3824.554266	-3824.539627	-233.89
TS1-S	-3825.553874	-3824.580048	1.089024	-3824.414017	0.136433	0.121139	-3824.550450	-3824.535156	-100.41
post_attack_R	-3825.572403	-3824.600613	1.093463	-3824.428028	0.139242	0.122301	-3824.567270	-3824.550328	
post_attack_S	-3825.567220	-3824.595639	1.093049	-3824.423164	0.138826	0.122247	-3824.561990	-3824.545410	
pre_closure_RR	-3825.572373	-3824.600613	1.093459	-3824.428005	0.139004	0.122183	-3824.567009	-3824.550188	
pre_closure_SS	-3825.568600	-3824.596564	1.093064	-3824.424552	0.138659	0.122117	-3824.563212	-3824.546669	
TS2-RR	-3825.546944	-3824.577465	1.089847	-3824.406417	0.135526	0.120657	-3824.541943	-3824.527074	-265.18
TS2-SS	-3825.541902	-3824.571991	1.089408	-3824.401629	0.136829	0.121380	-3824.538458	-3824.523009	-304.57
epoxide_RR	-3825.616073	-3824.641646	1.089120	-3824.475005	0.141033	0.123910	-3824.616038	-3824.598916	
epoxide_SS	-3825.625428	-3824.650080	1.088870	-3824.484515	0.141978	0.124452	-3824.626494	-3824.608967	
TS1-R_naphtyl	-4056.647500	-4055.610900	1.166352	-4055.426800	0.143464	0.127627	-4055.570200	-4055.554400	-186.12
TS1-S_naphtyl	-4056.644000	-4055.607100	1.167371	-4055.422300	0.143200	0.127439	-4055.565500	-4055.549700	-103.54
TS2-RR_naphtyl	-4056.639446	-4055.605989	1.167780	-4055.417233	0.144226	0.127892	-4055.561460	-4055.545125	-265.13
TS2-RR_naphtyl	-4056.635086	-4055.601008	1.167439	-4055.413163	0.144651	0.128122	-4055.557813	-4055.541285	-303.43

Table S4. Summary of optimized structures at B3LYP/6-311++G(2d,2p)-CPCM[toluene]//B3LYP/6-31G(d)-CPCM[toluene], at 253K. Energies in a.u. For each pathway, the post_attack and pre_closure structures have very similar energies ($\Delta G° < 0.2$ kcal/mol) and they were grouped as "intermediate" in the graphical representation of Figure S3.

Geometries

Pre_complex R

```
Imaginary Frequency (cm-1)  : None
N      3.4072920000    0.0867470000    0.1996380000
C      4.1100330000   -1.1432970000   -0.1378770000
C      5.4711260000   -0.8458320000   -0.8289510000
H      3.4614220000   -1.6677220000   -0.8368740000
C      4.3444690000   -2.0172770000    1.1021430000
H      6.1445170000   -0.4253680000   -0.0736910000
C      6.1045910000   -2.1472400000   -1.4219230000
N      5.3683210000    0.2065980000   -1.8782420000
C      3.8902980000   -3.3706680000    1.2051490000
C      5.0428450000   -1.4931200000    2.1742090000
C      6.4208990000   -1.9125940000   -2.9115950000
H      7.0166330000   -2.4116330000   -0.8750950000
H      5.4201130000   -2.9972060000   -1.3217130000
C      4.5386570000   -0.2509560000   -3.0224770000
C      6.7366180000    0.5109970000   -2.3731570000
C      3.1275960000   -4.0356500000    0.2022050000
C      4.2354110000   -4.0889040000    2.4005500000
C      5.3092370000   -2.2828910000    3.3117690000
H      5.3989840000   -0.4663790000    2.1586390000
C      5.1135550000   -1.5565290000   -3.6464470000
H      6.8622570000   -2.8176870000   -3.3430670000
C      7.3998930000   -0.7309320000   -3.0346690000
H      3.5106900000   -0.3833490000   -2.6755060000
H      4.5258460000    0.5665820000   -3.7505810000
H      7.3224240000    0.8724000000   -1.5218950000
H      6.6466010000    1.3422490000   -3.0801100000
C      2.7457930000   -5.3529120000    0.3702490000
H      2.8082120000   -3.4890980000   -0.6741030000
C      3.8277200000   -5.4468070000    2.5306670000
N      4.9370740000   -3.5428220000    3.4325090000
H      5.8612440000   -1.8538440000    4.1472640000
H      4.3958270000   -2.3805700000   -3.5523260000
H      5.3069690000   -1.4220040000   -4.7172350000
H      7.6254380000   -0.5363400000   -4.0898270000
C      3.1086770000   -6.0677930000    1.5451630000
O      2.0127880000   -6.0719880000   -0.5237090000
H      4.1054480000   -5.9682680000    3.4412100000
H      2.7901260000   -7.1015060000    1.6367900000
C      1.5864020000   -5.4257300000   -1.7182700000
H      1.0249090000   -6.1757590000   -2.2776560000
H      2.4428920000   -5.0896240000   -2.3164890000
H      0.9385050000   -4.5685290000   -1.4990800000
N      1.7278890000    1.6188580000    0.2567740000
H      3.9669580000    0.8452800000    0.5815970000
H      2.4333800000    2.2689990000    0.5990180000
H      4.8839780000    1.8055730000   -1.3155660000
C      2.1065650000    0.3660190000   -0.0850340000
S     -6.6982250000   -0.0584240000    0.7058650000
O     -6.8253100000   -0.2953410000    2.1527720000
O     -5.9580630000    1.1162080000    0.2068510000
C     -8.3448570000   -0.0278090000   -0.0044310000
C     -9.3869450000   -0.6646550000    0.6740300000
C     -8.5487540000    0.6311050000   -1.2200430000
C    -10.6641490000   -0.6411600000    0.1127380000
H     -9.2014260000   -1.1532280000    1.6240750000
C     -9.8310300000    0.6468090000   -1.7667810000
H     -7.7229950000    1.1298660000   -1.7157960000
C    -10.8840630000    0.0101560000   -1.1032610000
H    -11.4858610000   -1.1286530000    0.6284190000
H    -10.0083640000    1.1592400000   -2.7075360000
H    -11.8808950000    0.0256500000   -1.5344050000
C     -6.5156030000   -2.7537800000    0.3457220000
N     -7.0593380000   -3.7346670000    0.6633020000
C     -5.9065980000   -1.5262800000   -0.0209830000
C     -4.8069590000   -1.3366880000   -0.7929070000
O      4.6806110000    2.7648910000   -1.0663650000
O      4.7123500000    2.6641710000    0.3905380000
C      5.5949200000    3.6862180000    0.9346920000
C      7.0291960000    3.4503740000    0.4550270000
H      7.7030630000    4.1954160000    0.8916770000
H      7.3780220000    2.4558390000    0.7556540000
H      7.0869020000    3.5291250000   -0.6334640000
C      5.4661130000    3.4596700000    2.4446000000
H      4.4332280000    3.6103450000    2.7738300000
H      5.7796940000    2.4463670000    2.7182700000
H      6.1041090000    4.1705810000    2.9795030000
C      5.0757560000    5.0711620000    0.5370820000
H      5.6697510000    5.8530490000    1.0229550000
H      5.1384740000    5.2119170000   -0.5451230000
H      4.0306390000    5.1805420000    0.8441970000
H     -4.5236940000   -0.2932590000   -0.9128450000
H      8.3492460000   -0.9729650000   -2.5425090000
C     -3.9395020000   -2.2844800000   -1.4746270000
C     -2.8005340000   -1.7521270000   -2.1158340000
C     -4.1665720000   -3.6759390000   -1.5412510000
C     -1.9131740000   -2.5796820000   -2.7972970000
H     -2.6120370000   -0.6831050000   -2.0683490000
C     -3.2808480000   -4.4969960000   -2.2294190000
H     -5.0341810000   -4.1148260000   -1.0634730000
C     -2.1542130000   -3.9540190000   -2.8579050000
H     -1.0380690000   -2.1515440000   -3.2761960000
H     -3.4682330000   -5.5656200000   -2.2774260000
H     -1.4684650000   -4.6032650000   -3.3950440000
S      1.0424980000   -0.7605380000   -0.8040490000
C      0.3998590000    2.1841240000    0.0883540000
H     -0.3056070000    1.3561340000    0.1708430000
C      0.1368820000    3.1772940000    1.2806010000
H     -0.8333880000    3.6448480000    1.0728260000
N      1.2195300000    4.1772590000    1.2887200000
H      1.1494140000    4.7470240000    0.4468570000
H      1.0912530000    4.8058080000    2.0797800000
C      0.2179310000    2.8777430000   -1.2687650000
C     -1.0905440000    3.0504930000   -1.8383130000
C      1.3148360000    3.3851970000   -1.9421390000
C     -2.2775910000    2.5199310000   -1.2562770000
C     -1.2246370000    3.7832350000   -3.0680890000
C      1.1763530000    4.0938400000   -3.1594580000
H      2.3137250000    3.2252020000   -1.5474980000
C     -3.5179770000    2.7254760000   -1.8244140000
H     -2.2169290000    1.9325910000   -0.3482430000
C     -2.5161730000    3.9751190000   -3.6304230000
C     -0.0663660000    4.2996830000   -3.7072210000
H      2.0654160000    4.4765790000   -3.6535490000
C     -3.6413340000    3.4673090000   -3.0226100000
H     -4.4028080000    2.3193590000   -1.3420920000
H     -2.5967030000    4.5363330000   -4.5585880000
H     -0.1821540000    4.8510910000   -4.6372730000
H     -4.6220160000    3.6274690000   -3.4622750000
C      0.0642700000    2.4561650000    2.6243770000
C     -1.1778190000    1.9388070000    3.1334670000
C      1.2194780000    2.2624510000    3.3602560000
C     -2.4365660000    2.1240050000    2.4930530000
C     -1.1678380000    1.1964440000    4.3659760000
C      1.2204220000    1.5499990000    4.5812270000
H      2.1528180000    2.6689230000    2.9871320000
C     -3.5974810000    1.5852150000    3.0049580000
H     -2.4998520000    2.7102440000    1.5847910000
C     -2.3823640000    0.6534620000    4.8676480000
C      0.0524220000    1.0169090000    5.0694110000
H      2.1535110000    1.4200930000    5.1227670000
C     -3.5725620000    0.8314720000    4.2009330000
H     -4.5369890000    1.7373140000    2.4828760000
H     -2.3493570000    0.0877570000    5.7959220000
H      0.0428390000    0.4546360000    6.0000230000
H     -4.4928510000    0.4021620000    4.5864990000
```

Pre_complex S

```
Imaginary Frequency (cm-1)  : None

N     -0.5769410000   -0.8171490000   -0.5886330000
C     -1.2004830000   -1.9969360000   -1.1859500000
C     -0.1302290000   -2.9842050000   -1.7135670000
H     -1.7903160000   -1.6277460000   -2.0216150000
C     -2.1167310000   -2.7043640000   -0.1755070000
H      0.4850860000   -3.2875200000   -0.8592970000
C     -0.7728320000   -4.2543460000   -2.3576940000
N      0.8216440000   -2.3497070000   -2.6646180000
C     -3.5128040000   -2.9243480000   -0.4049860000
C     -1.5820710000   -3.1670980000    1.0134940000
C     -0.0373800000   -4.5667870000   -3.6748200000
H     -0.7121310000   -5.1022440000   -1.6666190000
H     -1.8373030000   -4.0932360000   -2.5667550000
C      0.1866790000   -2.0881520000   -3.9805210000
C      1.9588430000   -3.2844740000   -2.8668950000
C     -4.2091270000   -2.4863440000   -1.5678000000
C     -4.2412790000   -3.6341970000    0.6096570000
C     -2.3950110000   -3.8405630000    1.9488990000
H     -0.5317680000   -3.0188110000    1.2470170000
C     -0.2914710000   -3.4070950000   -4.6561400000
H     -0.4093090000   -5.5078660000   -4.0948040000
C      1.4750350000   -4.6649960000   -3.3984950000
H     -0.6364450000   -1.3845270000   -3.8305400000
H      0.9332350000   -1.5745070000   -4.5924910000
H      2.4817810000   -3.3823210000   -1.9097850000
H      2.6484900000   -2.8000060000   -3.5639920000
C     -5.5568810000   -2.7498460000   -1.7202380000
H     -3.6865640000   -1.9044980000   -2.3138110000
C     -5.6271030000   -3.8923000000    0.4100040000
```

N	-3.6800340000	-4.0813460000	1.7679510000
H	-1.9573390000	-4.2000160000	2.8797540000
H	-1.3580200000	-3.3518890000	-4.9051850000
H	0.2483600000	-3.5821380000	-5.5946980000
H	2.0111860000	-4.9372690000	-4.3153840000
C	-6.2704170000	-3.4694850000	-0.7220930000
O	-6.3108350000	-2.3634310000	-2.7855850000
H	-6.1533230000	-4.4341620000	1.1896180000
H	-7.3274990000	-3.6583600000	-0.8816170000
C	-5.6829990000	-1.6032980000	-3.8138470000
H	-4.8791110000	-2.1740820000	-4.2956730000
H	-5.2756020000	-0.6636710000	-3.4223660000
H	-6.4642910000	-1.3883410000	-4.5443730000
N	-0.5402850000	1.3010090000	0.2657550000
H	0.1583750000	-1.0100540000	0.0873280000
H	0.1109740000	0.9106200000	0.9407400000
H	1.7374830000	-0.8999600000	-2.1227350000
C	-1.0828520000	0.4456710000	-0.6415440000
S	2.2147000000	-0.9998520000	2.2351530000
O	1.4856490000	0.2549250000	2.5362010000
O	1.7781750000	-1.7911320000	1.0629900000
C	2.1497700000	-2.0429430000	3.6854210000
C	2.3488720000	-3.4186650000	3.5315580000
C	1.8984110000	-1.4624460000	4.9311450000
C	2.2935430000	-4.2300840000	4.6633730000
H	2.5298910000	-3.8412870000	2.5491450000
C	1.8470110000	-2.2903900000	6.0526220000
H	1.7350500000	-0.3940590000	5.0162680000
C	2.0452820000	-3.6666200000	5.9186270000
H	2.4395720000	-5.3012270000	4.5642470000
H	1.6486870000	-1.8584670000	7.0284830000
H	2.0027210000	-4.3048430000	6.7962510000
C	4.6169140000	-0.1212140000	3.1528240000
N	5.1070840000	0.2673580000	4.1363690000
C	3.9649780000	-0.6044340000	1.9894940000
C	4.4668660000	-0.7242540000	0.7313480000
O	2.4330860000	-0.2179110000	-1.8713180000
O	3.1764420000	-0.1378050000	-3.1262920000
C	3.0734710000	1.2036420000	-3.6800230000
C	1.6178520000	1.5401090000	-4.0152240000
H	0.9976090000	1.5211820000	-3.1156330000
H	1.5520070000	2.5416710000	-4.4543230000
H	1.2087910000	0.8247480000	-4.7366390000
C	3.9234370000	1.0839820000	-4.9507980000
H	3.9351210000	2.0417560000	-5.4811860000
H	4.9561510000	0.8135210000	-4.7057680000
H	3.5140780000	0.3227670000	-5.6234310000
C	3.6724910000	2.2236240000	-2.7070680000
H	4.7112150000	1.9687820000	-2.4709180000
H	3.6543380000	3.2263240000	-3.1483500000
H	3.0981700000	2.2512870000	-1.7780580000
H	3.7488200000	-1.0327950000	-0.0278350000
H	1.6710440000	-5.4572310000	-2.6655770000
C	5.8096540000	-0.4734540000	0.2325470000
C	5.9787900000	-0.4999580000	-1.1697150000
C	6.9355200000	-0.2336680000	1.0500760000
C	7.2325570000	-0.2810490000	-1.7342040000
H	5.1159450000	-0.6641340000	-1.8094010000
C	8.1848180000	-0.0246200000	0.4777460000
H	6.8406980000	-0.2232500000	2.1292130000
C	8.3375540000	-0.0453120000	-0.9133750000
H	7.3475500000	-0.2973950000	-2.8140460000
H	9.0446450000	0.1528640000	1.1168470000
H	9.3170770000	0.1200790000	-1.3532280000
S	-2.2649610000	0.9151970000	-1.7782280000
C	-0.9587570000	2.6824630000	0.4818020000
H	-1.7911500000	2.8261060000	-0.2052450000
C	-1.5177670000	2.8826290000	1.9558010000
H	-1.7931260000	3.9390340000	2.0069220000
N	-0.5426820000	2.6922290000	3.0328530000
H	-0.1345780000	1.7600750000	3.0247570000
H	0.2331660000	3.3380870000	2.8979330000
C	0.1421650000	3.6949400000	0.1779460000
C	-0.1756440000	4.9757100000	-0.3944600000
C	1.4549220000	3.4262480000	0.5258060000
C	-1.4850120000	5.3465370000	-0.8178790000
C	0.8765540000	5.9413540000	-0.5675450000
C	2.4868780000	4.3775960000	0.3466270000
H	1.7174190000	2.4598290000	0.9443190000
C	-1.7425500000	6.5881650000	-1.3578690000
H	-2.3035610000	4.6416080000	-0.7326910000
C	0.5754000000	7.2131730000	-1.1265230000
C	2.2032780000	5.6130710000	-0.1824140000
H	3.5021600000	4.1223430000	0.6371730000
C	-0.7051490000	7.5370060000	-1.5108740000
H	-2.7515170000	6.8366990000	-1.6752320000
H	1.3849970000	7.9293530000	-1.2463300000
H	2.9871060000	6.3544750000	-0.3169130000
H	-0.9208510000	8.5128800000	-1.9370260000
C	-2.7680940000	2.0366830000	2.1801030000
C	-4.0781170000	2.5436690000	1.8724340000
C	-2.6517270000	0.7443170000	2.6579150000
C	-4.3234760000	3.8656230000	1.3999760000
C	-5.2182380000	1.6857270000	2.0559080000
C	-3.7742700000	-0.0967720000	2.8349210000
H	-1.6744770000	0.3433020000	2.9075090000
C	-5.5979410000	4.3081000000	1.1183530000
H	-3.4978570000	4.5534190000	1.2587490000
C	-6.5192080000	2.1734780000	1.7554150000
C	-5.0335080000	0.3629310000	2.5361820000
H	-3.6310490000	-1.1097670000	3.2000700000
C	-6.7117740000	3.4547120000	1.2934760000
H	-5.7475880000	5.3236010000	0.7615930000
H	-7.3658440000	1.5062950000	1.8996990000
H	-5.9035590000	-0.2767940000	2.6613870000
H	-7.7120930000	3.8136820000	1.0674660000

TS1-R

Imaginary Frequency (cm-1) : -233.89

N	-0.0545340000	-0.9001980000	-0.3291870000
C	0.0273640000	-2.3285210000	-0.6224260000
C	1.4782320000	-2.7890310000	-0.9174910000
H	-0.5709310000	-2.4718140000	-1.5194270000
C	-0.5549060000	-3.1729780000	0.5235520000
H	2.0764230000	-2.6906610000	-0.0079440000
C	1.5086050000	-4.2618550000	-1.4317740000
N	2.1830450000	-1.9174840000	-1.9309660000
C	-1.7308880000	-3.9785450000	0.3743970000
C	0.0680430000	-3.1654450000	1.7579620000
C	2.0704800000	-4.3017210000	-2.8656550000
H	2.1197430000	-4.8811110000	-0.7670260000
H	0.4993930000	-4.6850580000	-1.4180110000
C	1.4628290000	-1.8925950000	-3.2483960000
C	3.5788550000	-2.4453730000	-2.1440800000
C	-2.4941630000	-4.0698690000	-0.8244060000
C	-2.1489270000	-4.7419790000	1.5177380000
C	-0.4445490000	-3.9397920000	2.8209590000
H	0.9625600000	-2.5780490000	1.9412900000
C	1.2523440000	-3.3419350000	-3.7517230000
H	2.0085820000	-5.3219840000	-3.2572930000
C	3.5329340000	-3.8254860000	-2.8378460000
H	0.5250310000	-1.3506980000	-3.1129890000
H	2.0936680000	-1.3044530000	-3.9163560000
H	4.0509530000	-2.4886830000	-1.1607720000
H	4.1008210000	-1.6857070000	-2.7267980000
C	-3.6062870000	-4.8883760000	-0.8883050000
H	-2.2340170000	-3.4509060000	-1.6716330000
C	-3.2941860000	-5.5799660000	1.4069310000
N	-1.5088820000	-4.7125920000	2.7201910000
H	0.0599930000	-3.9226980000	3.7859680000
H	0.1905770000	-3.6117400000	-3.7169010000
H	1.5690190000	-3.4241530000	-4.7968540000
H	3.9279460000	-3.7586860000	-3.8576750000
C	-4.0031320000	-5.6598970000	0.2385160000
O	-4.4051670000	-5.0313300000	-1.9801890000
H	-3.5838550000	-6.1500410000	2.2840200000
H	-4.8797570000	-6.2933620000	0.1452590000
C	-4.1049760000	-4.2602680000	-3.1403600000
H	-4.1354010000	-3.1861750000	-2.9235340000
H	-4.8754420000	-4.5103950000	-3.8711400000
H	-3.1187130000	-4.5201370000	-3.5454690000
N	-1.1433200000	1.0863070000	-0.0866690000
H	0.4310520000	-0.5852670000	0.5086970000
H	-0.4807710000	1.2084640000	0.6756080000
H	2.3651100000	-0.8474400000	-1.5538170000
C	-1.0650740000	-0.0820580000	-0.7654080000
S	2.2298970000	1.4706060000	2.1481230000
O	2.3959930000	2.7548770000	1.4425870000
O	0.9075070000	0.7761420000	2.0689830000
C	2.5461160000	1.7410990000	3.8933010000
C	3.3771550000	2.8022870000	4.2627260000
C	1.9713070000	0.8899880000	4.8405580000
C	3.6362460000	3.0109460000	5.6165680000
H	3.7967190000	3.4553840000	3.5051920000
C	2.2431810000	1.1107660000	6.1911840000
H	1.3168920000	0.0843340000	4.5280050000
C	3.0732150000	2.1658320000	6.5771380000
H	4.2736640000	3.8357080000	5.9206690000
H	1.8010730000	0.4606480000	6.9399720000
H	3.2786010000	2.3334770000	7.6304240000
C	3.4182810000	-0.9313450000	2.1843670000
N	3.3187920000	-1.9686270000	2.7158760000
C	3.4834410000	0.3380540000	1.5751420000
C	4.2788580000	0.7357530000	0.5166720000
O	2.8769540000	0.4524960000	-1.2475690000
O	3.4351170000	0.7413870000	-2.5571640000
C	2.9074950000	1.9786420000	-3.1011200000
C	1.3918890000	1.8747320000	-3.2982490000
H	0.9887400000	2.8086390000	-3.7050820000
H	1.1446570000	1.0668040000	-3.9952830000
H	0.9003260000	1.6751530000	-2.3439130000
C	3.6339360000	2.0842440000	-4.4478390000
H	3.3257210000	2.9943150000	-4.9735790000
H	4.7190430000	2.1200820000	-4.3018980000
H	3.3993180000	1.2244400000	-5.0850170000

C	3.2589130000	3.1666860000	-2.1975320000
H	4.3454120000	3.2755680000	-2.0998530000
H	2.8663330000	4.0973730000	-2.6226330000
H	2.8256180000	3.0334900000	-1.2036550000
H	4.1550420000	1.7705040000	0.2273820000
H	4.1591980000	-4.5411900000	-2.2947980000
C	5.4795700000	0.0833960000	-0.0100010000
C	5.9881940000	-1.1471780000	0.4529710000
C	6.1937290000	0.7616260000	-1.0178610000
C	7.1590790000	-1.6774900000	-0.0835650000
H	5.4827730000	-1.6898160000	1.2423080000
C	7.3628650000	0.2280850000	-1.5537510000
H	5.8181280000	1.7111440000	-1.3802550000
C	7.8496270000	-0.9957360000	-1.0897760000
H	7.5380490000	-2.6238540000	0.2920550000
H	7.8960950000	0.7697040000	-2.3299300000
H	8.7642650000	-1.4124530000	-1.5024650000
S	-2.0825720000	-0.4765840000	-2.0737730000
C	-2.2126220000	2.0717280000	-0.1931340000
H	-2.9602390000	1.6145830000	-0.8397230000
C	-2.8745590000	2.2977250000	1.2345520000
H	-3.6078860000	3.0924890000	1.0799040000
N	-1.9696740000	2.8093380000	2.2672030000
H	-1.2132580000	2.1615940000	2.4786070000
H	-1.5327770000	3.6691240000	1.9393590000
C	-1.7508280000	3.3963700000	-0.7947190000
C	-2.6432540000	4.1864900000	-1.5998910000
C	-0.4904790000	3.8916350000	-0.5097580000
C	-3.9489950000	3.7603070000	-1.9792910000
C	-2.2026170000	5.4731940000	-2.0680270000
C	-0.0610770000	5.1559560000	-0.9784050000
H	0.2097250000	3.3030330000	0.0738710000
C	-4.7696610000	4.5536500000	-2.7520560000
H	-4.3142950000	2.7880010000	-1.6705180000
C	-3.0751160000	6.2662130000	-2.8618070000
C	-0.9009980000	5.9346100000	-1.7362580000
H	0.9377410000	5.5018570000	-0.7279110000
C	-4.3341120000	5.8235120000	-3.1961360000
H	-5.7580030000	4.1959490000	-3.0273950000
H	-2.7232080000	7.2369370000	-3.2032640000
H	-0.5832970000	6.9102230000	-2.0961660000
H	-4.9902350000	6.4403680000	-3.8039590000
C	-3.5994460000	1.0381130000	1.7024310000
C	-4.9896770000	0.8217870000	1.4025480000
C	-2.9109790000	0.0609630000	2.3986440000
C	-5.8084880000	1.7659690000	0.7172500000
C	-5.6092160000	-0.4080140000	1.8200020000
C	-3.5238190000	-1.1458730000	2.8073750000
H	-1.8629720000	0.2006150000	2.6439690000
C	-7.1371140000	1.5133570000	0.4516370000
H	-5.3922390000	2.7131990000	0.3951440000
C	-6.9817740000	-0.6370030000	1.5292970000
C	-4.8462660000	-1.3789020000	2.5198350000
H	-2.9377190000	-1.8879700000	3.3418990000
C	-7.7345610000	0.2977330000	0.8573770000
H	-7.7312540000	2.2581830000	-0.0709710000
H	-7.4275450000	-1.5746070000	1.8531480000
H	-5.3261210000	-2.3065900000	2.8214990000
H	-8.7827020000	0.1088960000	0.6422660000

TS1-S

Imaginary Frequency (cm-1) : -100.41

N	-0.5036120000	-0.9501420000	-0.3092040000
C	-0.9242880000	-2.2588260000	-0.7951560000
C	0.2946160000	-3.1356420000	-1.1750890000
H	-1.5120340000	-2.0620090000	-1.6887920000
C	-1.7902220000	-2.9998980000	0.2367070000
H	0.8832760000	-3.3255560000	-0.2749600000
C	-0.1404010000	-4.4710260000	-1.8511180000
N	1.2557480000	-2.4263390000	-2.1075210000
C	-3.1563020000	-3.3538930000	-0.0107940000
C	-1.2439340000	-3.3475410000	1.4590050000
C	0.4001000000	-4.5305050000	-3.2922350000
H	0.2284980000	-5.3237890000	-1.2718320000
H	-1.2320120000	-4.5462300000	-1.8628520000
C	0.6002680000	-2.0273520000	-3.4026150000
C	2.4144810000	-3.3461160000	-2.4055950000
C	-3.8641910000	-3.0358300000	-1.2049620000
C	-3.8400480000	-4.0795440000	1.0239740000
C	-2.0187890000	-4.0418430000	2.4127980000
H	-0.2194450000	-3.0899380000	1.7104990000
C	-0.0565690000	-3.2692080000	-4.0508590000
H	0.0219390000	-5.4283120000	-3.7908900000
C	1.9376750000	-4.5524340000	-3.2452720000
H	-0.1128410000	-1.2297270000	-3.1866760000
H	1.3988100000	-1.6042360000	-4.0122540000
H	2.8243170000	-3.6498560000	-1.4392850000
H	3.1570860000	-2.7309920000	-2.9139620000
C	-5.1787970000	-3.4309330000	-1.3681810000
H	-3.3876890000	-2.4316320000	-1.9642450000
C	-5.1898730000	-4.4781760000	0.8108930000
N	-3.2694490000	-4.4121070000	2.2154050000
H	-1.5760400000	-4.3073360000	3.3719800000
H	-1.1489770000	-3.1881140000	-4.0207030000
H	0.2305060000	-3.3330710000	-5.1056040000
H	2.3480970000	-4.5009850000	-4.2596220000
C	-5.8439000000	-4.1708950000	-0.3517580000
O	-5.9421180000	-3.1616670000	-2.4612080000
H	-5.6802140000	-5.0303570000	1.6063780000
H	-6.8746510000	-4.4660100000	-0.5216530000
C	-5.3707710000	-2.3805790000	-3.5072530000
H	-4.5082190000	-2.8889740000	-3.9565860000
H	-5.0608070000	-1.3940200000	-3.1435030000
H	-6.1559060000	-2.2667290000	-4.2560630000
N	-0.7539580000	1.2571670000	0.1859380000
H	0.0567290000	-0.9689500000	0.5372720000
H	-0.0767300000	1.0421700000	0.9151020000
H	1.7380840000	-1.5377950000	-1.6464690000
C	-1.1240840000	0.2339060000	-0.6147090000
S	2.1918350000	-0.6161000000	2.3168600000
O	1.1206840000	0.4180480000	2.3622380000
O	1.8451390000	-1.9315950000	1.7251060000
C	2.7018600000	-0.8887470000	4.0143380000
C	3.2098470000	-2.1402930000	4.3726040000
C	2.6030580000	0.1582700000	4.9345170000
C	3.6255010000	-2.3437640000	5.6877900000
H	3.2629070000	-2.9376480000	3.6393160000
C	3.0272460000	-0.0608450000	6.2454450000
H	2.1919190000	1.1156110000	4.6347560000
C	3.5372630000	-1.3061900000	6.6202000000
H	4.0147020000	-3.3131670000	5.9842700000
H	2.9532300000	0.7413940000	6.9732490000
H	3.8633540000	-1.4704740000	7.6430990000
C	3.9144310000	1.3570500000	1.7860510000
N	4.1456610000	2.4565930000	2.1090250000
C	3.5918200000	0.0276880000	1.4393250000
C	4.1467460000	-0.7540460000	0.4375110000
O	2.7486020000	-0.4310670000	-1.2884640000
O	3.2959980000	-0.3246640000	-2.6266570000
C	3.2631820000	1.0441990000	-3.1104260000
C	1.8187930000	1.5516290000	-3.1781580000
H	1.2188860000	0.9400020000	-3.8608540000
H	1.3616870000	1.5183790000	-2.1868460000
H	1.7896770000	2.5862900000	-3.5376140000
C	3.8745540000	0.9156310000	-4.5111600000
H	3.2721250000	0.2530740000	-5.1430220000
H	3.9219530000	1.8973030000	-4.9945980000
H	4.8902590000	0.5098240000	-4.4522550000
C	4.1188670000	1.9572750000	-2.2272730000
H	5.1580600000	1.6161790000	-2.2055300000
H	4.0975020000	2.9836770000	-2.6111910000
H	3.7331550000	1.9650990000	-1.2053620000
H	3.7215320000	-1.7449380000	0.3579600000
H	2.2947220000	-5.4860690000	-2.7983550000
C	5.4481160000	-0.5823680000	-0.2118410000
C	5.7985740000	-1.4879200000	-1.2316640000
C	6.3903320000	0.3941130000	0.1641780000
C	7.0382990000	-1.4188870000	-1.8602780000
H	5.0835330000	-2.2465830000	-1.5284660000
C	7.6314220000	0.4616240000	-0.4649830000
H	6.1657040000	1.0964890000	0.9574960000
C	7.9601160000	-0.4401150000	-1.4805450000
H	7.2867700000	-2.1285490000	-2.6445580000
H	8.3467020000	1.2184960000	-0.1556770000
H	8.9298950000	-0.3843640000	-1.9673840000
S	-2.2112220000	0.3937570000	-1.9176250000
C	-1.3458550000	2.5906870000	0.2079890000
H	-2.1471870000	2.5562590000	-0.5282450000
C	-2.0031070000	2.8671140000	1.6291910000
H	-2.3976720000	3.8843550000	1.5647940000
N	-1.0677170000	2.8908910000	2.7563890000
H	-0.5535350000	2.0172200000	2.8509740000
H	-0.3681090000	3.6129820000	2.5943810000
C	-0.3484060000	3.6860640000	-0.1577150000
C	-0.7673700000	4.8475090000	-0.8950030000
C	0.9612510000	3.6120180000	0.2844990000
C	-2.0815150000	5.0180430000	-1.4192960000
C	0.1855500000	5.8983120000	-1.1353480000
C	1.8958810000	4.6455650000	0.0414500000
H	1.3052870000	2.7391860000	0.8292350000
C	-2.4370090000	6.1517480000	-2.1179210000
H	-2.8252190000	4.2414050000	-1.2842810000
C	-0.2154010000	7.0542070000	-1.8591190000
C	1.5141950000	5.7693360000	-0.6500310000
H	2.9089500000	4.5357470000	0.4170910000
C	-1.4981710000	7.1860100000	-2.3385140000
H	-3.4466380000	6.2478150000	-2.5078720000
H	0.5194410000	7.8379530000	-2.0279190000
H	2.2207080000	6.5742740000	-0.8377610000
H	-1.7901560000	8.0754900000	-2.8899650000
C	-3.1589680000	1.9026250000	1.8815300000
C	-4.4936100000	2.2070610000	1.4412950000
C	-2.9268970000	0.6933880000	2.5112110000
C	-4.8566590000	3.4312550000	0.8084120000
C	-5.5358190000	1.2383190000	1.6534330000

C	-3.9536970000	-0.2567000000	2.7168750000
H	-1.9304330000	0.4450610000	2.8622610000
C	-6.1501580000	3.6796300000	0.4026430000
H	-4.1092310000	4.1981970000	0.6415450000
C	-6.8594490000	1.5261400000	1.2221990000
C	-5.2329160000	0.0075150000	2.2923760000
H	-3.7196830000	-1.1978020000	3.2066540000
C	-7.1661710000	2.7174490000	0.6066490000
H	-6.3913970000	4.6256780000	-0.0745130000
H	-7.6308760000	0.7784650000	1.3915250000
H	-6.0289340000	-0.7183950000	2.4385870000
H	-8.1825200000	2.9235680000	0.2823990000

Post_Attack R
Imaginary Frequency (cm-1) : None

N	0.2893020000	-1.0136300000	0.4440380000
C	0.4045590000	-2.4259790000	0.8141570000
C	-0.9735310000	-3.0138440000	1.1947660000
H	1.0470610000	-2.4439150000	1.6922460000
C	1.0297650000	-3.2715060000	-0.3033800000
H	-1.6099550000	-3.0665950000	0.3088560000
C	-0.8747830000	-4.3974340000	1.8919490000
N	-1.7363370000	-2.0747850000	2.1274540000
C	2.3088350000	-3.9029370000	-0.1676430000
C	0.3477620000	-3.4348900000	-1.4956530000
C	-1.5116670000	-4.3263100000	3.2929360000
H	-1.3772100000	-5.1541550000	1.2820740000
H	0.1744370000	-4.6990990000	1.9762870000
C	-1.0302380000	-1.8604970000	3.4494870000
C	-3.1236010000	-2.6320680000	2.3704090000
C	3.1357210000	-3.8052650000	0.9877430000
C	2.7684600000	-4.6859440000	-1.2812610000
C	0.9089560000	-4.2107200000	-2.5331210000
H	-0.6280070000	-2.9856650000	-1.6579830000
C	-0.7960160000	-3.2363380000	4.1141480000
H	-1.4106250000	-5.2946120000	3.7917610000
C	-3.0008930000	-3.9566240000	3.1576580000
H	-0.1064810000	-1.3139810000	3.2490160000
H	-1.6873660000	-1.2169700000	4.0368210000
H	-3.5770220000	-2.7616680000	1.3872930000
H	-3.6731720000	-1.8556680000	2.9039640000
C	4.3500640000	-4.4641930000	1.0389070000
H	2.8354150000	-3.1701030000	1.8097200000
C	4.0202280000	-5.3564420000	-1.1834200000
N	2.0712790000	-4.8275440000	-2.4427930000
H	0.3618370000	-4.3302240000	-3.4670440000
H	0.2764470000	-3.4501910000	4.1702800000
H	-1.1767790000	-3.2202520000	5.1400750000
H	-3.4537480000	-3.8522540000	4.1489120000
C	4.7908390000	-5.2568450000	-0.0565800000
O	5.2141730000	-4.4227140000	2.0885110000
H	4.3402750000	-5.9454810000	-2.0370880000
H	5.7486500000	-5.7609360000	0.0261170000
C	4.8723050000	-3.6171110000	3.2129750000
H	4.7475340000	-2.5662470000	2.9269670000
H	5.7052590000	-3.7121770000	3.9110480000
H	3.9516060000	-3.9739880000	3.6917600000
N	1.2078580000	1.0363830000	0.0465500000
H	-0.1602150000	-0.8283350000	-0.4559010000
H	0.5402210000	1.0402550000	-0.7239110000
H	-1.8434600000	-1.1673300000	1.6528590000
C	1.2165220000	-0.0620510000	0.8248290000
S	-2.1790700000	0.9811550000	-1.8708950000
O	-2.2158310000	2.3309280000	-1.2576680000
O	-0.8384220000	0.3032360000	-1.9776120000
C	-2.7004680000	1.1843310000	-3.5863910000
C	-3.4270630000	2.3233560000	-3.9410780000
C	-2.4130710000	0.1853320000	-4.5210230000
C	-3.8716300000	2.4616610000	-5.2569100000
H	-3.6209560000	3.0918540000	-3.2007120000
C	-2.8659830000	0.3346240000	-5.8317770000
H	-1.8345000000	-0.6848680000	-4.2308050000
C	-3.5949230000	1.4689160000	-6.1997440000
H	-4.4290410000	3.3483670000	-5.5449140000
H	-2.6437320000	-0.4335790000	-6.5667080000
H	-3.9423590000	1.5809660000	-7.2228520000
C	-3.2054600000	-1.4119630000	-1.2885280000
N	-3.1298620000	-2.5726950000	-1.4680850000
C	-3.2809490000	-0.0433290000	-1.0371230000
C	-4.2742170000	0.5448280000	-0.0876340000
O	-3.6593050000	0.3588400000	1.2298570000
O	-4.5473710000	0.8839330000	2.2855330000
C	-3.9981520000	2.0904110000	2.8835050000
C	-2.6101410000	1.8210190000	3.4711170000
H	-2.2395620000	2.7084580000	3.9952700000
H	-2.6525890000	0.9949120000	4.1901210000
H	-1.8934440000	1.5736470000	2.6832460000
C	-5.0238170000	2.3607880000	3.9935340000
H	-4.7404600000	3.2618960000	4.5472060000
H	-6.0217900000	2.5138590000	3.5704460000
H	-5.0679590000	1.5218420000	4.6956330000
C	-3.9647670000	3.2462570000	1.8781760000
H	-4.9598890000	3.4128710000	1.4515930000
H	-3.6509990000	4.1683990000	2.3805150000
H	-3.2605060000	3.0467040000	1.0659460000
H	-4.3411080000	1.6201340000	-0.2675160000
H	-3.5438830000	-4.7487710000	2.6333600000
C	-5.6665930000	-0.0630520000	-0.2059530000
C	-6.1660570000	-1.0250700000	0.6759180000
C	-6.4715900000	0.3507020000	-1.2769710000
C	-7.4401120000	-1.5684520000	0.4878080000
H	-5.5708190000	-1.3319820000	1.5267200000
C	-7.7420040000	-0.1899270000	-1.4672520000
H	-6.0927490000	1.0953350000	-1.9737800000
C	-8.2320090000	-1.1556890000	-0.5834050000
H	-7.8134920000	-2.3139030000	1.1854050000
H	-8.3501660000	0.1435160000	-2.3040300000
H	-9.2227410000	-1.5780330000	-0.7280500000
S	2.2253970000	-0.2395710000	2.1856710000
C	2.1585590000	2.1407020000	0.1118020000
H	3.0265240000	1.7500010000	0.6420750000
C	2.6194150000	2.5147210000	-1.3618460000
H	3.2505530000	3.3969020000	-1.2353440000
N	1.5481810000	2.9351870000	-2.2664150000
H	0.8996980000	2.1828780000	-2.4878630000
H	1.0016040000	3.6777220000	-1.8335850000
C	1.6154910000	3.3603190000	0.8515980000
C	2.4948230000	4.2148530000	1.6032710000
C	0.2772130000	3.6956250000	0.7484980000
C	3.8812150000	3.9478540000	1.7953380000
C	1.9564800000	5.4013470000	2.2122820000
C	-0.2453280000	4.8636570000	1.3532560000
H	-0.4045980000	3.0536720000	0.1995780000
C	4.6845100000	4.7983130000	2.5244670000
H	4.3264100000	3.0551190000	1.3722690000
C	2.8132080000	6.2571010000	2.9565130000
C	0.5761670000	5.7023160000	2.0659620000
H	-1.3018150000	5.0903210000	1.2413920000
C	4.1499340000	5.9693990000	3.1093910000
H	5.7371210000	4.5625600000	2.6550070000
H	2.3865740000	7.1504320000	3.4067470000
H	0.1847750000	6.6047800000	2.5294030000
H	4.7930780000	6.6328030000	3.6809900000
C	3.4545810000	1.3885710000	-1.9691220000
C	4.8883100000	1.3751510000	-1.8486970000
C	2.8325650000	0.3347320000	-2.6151040000
C	5.6423220000	2.4148500000	-1.2300230000
C	5.6221850000	0.2605970000	-2.3876450000
C	3.5584530000	-0.7577280000	-3.1430740000
H	1.7531150000	0.3191650000	-2.7274620000
C	7.0162810000	2.3563070000	-1.1380500000
H	5.1383100000	3.2829520000	-0.8219720000
C	7.0390470000	0.2315490000	-2.2752380000
C	4.9262340000	-0.7975230000	-3.0282870000
H	3.0226160000	-1.5667450000	-3.6315230000
C	7.7274670000	1.2523590000	-1.6622210000
H	7.5574140000	3.1695690000	-0.6619910000
H	7.5711850000	-0.6218880000	-2.6889580000
H	5.4943380000	-1.6353710000	-3.4248060000
H	8.8105530000	1.2166530000	-1.5839050000

Post_Attack S
Imaginary Frequency (cm-1) : None

N	0.9941060000	-1.1135170000	0.3151270000
C	1.3244780000	-2.3643400000	0.9866250000
C	0.0500040000	-3.1612910000	1.3836670000
H	1.8470120000	-2.0696220000	1.8949690000
C	2.2233570000	-3.2696150000	0.1359820000
H	-0.3830110000	-3.6137480000	0.4919560000
C	0.3157360000	-4.2278850000	2.4799750000
N	-1.0536140000	-2.2417900000	1.8804830000
C	3.5566930000	-3.6182800000	0.5221200000
C	1.7364110000	-3.7839860000	-1.0522430000
C	-0.5965830000	-3.9630010000	3.6945830000
H	0.1326380000	-5.2289030000	2.0778120000
H	1.3659110000	-4.1951510000	2.7886390000
C	-0.6698690000	-1.5048840000	3.1408390000
C	-2.3192220000	-3.0407810000	2.1145900000
C	4.2060240000	-3.1302540000	1.6916550000
C	4.2677310000	-4.5214010000	-0.3403960000
C	2.5353910000	-4.6429480000	-1.8363800000
H	0.7356000000	-3.5421950000	-1.4011760000
C	-0.3234370000	-2.5447200000	4.2329730000
H	-0.3918630000	-4.7027000000	4.4741560000
C	-2.0694960000	-4.0565830000	3.2530620000
H	0.1657610000	-0.8444890000	2.9046960000
H	-1.5301480000	-0.8866690000	3.3978530000
H	-2.5593990000	-3.5120000000	1.1610220000
H	-3.1001290000	-2.3111370000	2.3399130000
C	5.4919530000	-3.5351080000	1.9981900000
H	3.7109860000	-2.3958540000	2.3123820000
C	5.5849830000	-4.9236200000	0.0196640000

N	3.7532490000	-5.0198760000	-1.4985230000
H	2.1405480000	-5.0402100000	-2.7703570000
H	0.7279990000	-2.4470750000	4.5226990000
H	-0.9239940000	-2.3583520000	5.1288130000
H	-2.7297650000	-3.8451470000	4.1003390000
C	6.1823880000	-4.4518990000	1.1572410000
O	6.2024270000	-3.1161510000	3.0783830000
H	6.0981410000	-5.6117280000	-0.6444670000
H	7.1886950000	-4.7473900000	1.4371210000
C	5.6025600000	-2.1646770000	3.9540900000
H	4.6969780000	-2.5716090000	4.4212160000
H	5.3518360000	-1.2384950000	3.4240700000
H	6.3476850000	-1.9581670000	4.7235290000
N	1.1760180000	1.0849400000	-0.2323350000
H	0.5630630000	-1.1830350000	-0.6039270000
H	0.5144130000	0.8242140000	-0.9638120000
H	-1.2561090000	-1.5852720000	1.1114950000
C	1.5249360000	0.1171080000	0.6349380000
S	-1.9428220000	-0.8735010000	-1.8235940000
O	-0.6319670000	-0.2173640000	-2.1497420000
O	-1.8571740000	-1.9884040000	-0.8227510000
C	-2.4347890000	-1.6552330000	-3.3812710000
C	-1.4489330000	-2.2503010000	-4.1743740000
C	-3.7793900000	-1.6908760000	-3.7497940000
C	-1.8243460000	-2.8984840000	-5.3504480000
H	-0.4056520000	-2.1895860000	-3.8835610000
C	-4.1437780000	-2.3441680000	-4.9291870000
H	-4.5282980000	-1.2019250000	-3.1345070000
C	-3.1698960000	-2.9478710000	-5.7266140000
H	-1.0651520000	-3.3579380000	-5.9768630000
H	-5.1880310000	-2.3715040000	-5.2264070000
H	-3.4567970000	-3.4497170000	-6.6463300000
C	-3.0194070000	1.5336450000	-1.8747280000
N	-2.9353950000	2.6069610000	-2.3408210000
C	-3.1036360000	0.2576600000	-1.3009960000
C	-4.1493600000	-0.1623830000	-0.3088060000
O	-3.7272230000	0.3917040000	0.9788550000
O	-4.5345100000	-0.3074950000	2.0058220000
C	-4.9054990000	0.6071170000	3.0699650000
C	-3.6571940000	1.1305470000	3.7897490000
H	-3.9410680000	1.8285320000	4.5850500000
H	-3.1014930000	0.3071930000	4.2520040000
H	-2.9962600000	1.6565740000	3.0954480000
C	-5.7271110000	-0.3089250000	3.9878480000
H	-5.1272770000	-1.1581500000	4.3325580000
H	-6.0624170000	0.2528540000	4.8658350000
H	-6.6083950000	-0.6927860000	3.4642880000
C	-5.7652720000	1.7607450000	2.5473520000
H	-6.6385490000	1.3816520000	2.0099920000
H	-6.1104360000	2.3741820000	3.3870670000
H	-5.1941350000	2.3988210000	1.8691080000
H	-4.0996050000	-1.2498810000	-0.2030870000
H	-2.2988010000	-5.0695360000	2.9076250000
C	-5.5788280000	0.2108070000	-0.6716280000
C	-6.5271760000	-0.8087160000	-0.8272010000
C	-5.9842870000	1.5386150000	-0.8730390000
C	-7.8453600000	-0.5179510000	-1.1876620000
H	-6.2315040000	-1.8424690000	-0.6611260000
C	-7.2998040000	1.8322590000	-1.2309410000
H	-5.2706310000	2.3463450000	-0.7529340000
C	-8.2354300000	0.8059950000	-1.3912140000
H	-8.5646410000	-1.3246480000	-1.3030100000
H	-7.5949790000	2.8665650000	-1.3865330000
H	-9.2594770000	1.0386180000	-1.6708930000
S	2.4825300000	0.3901370000	2.0168010000
C	1.5784940000	2.4848450000	-0.1683190000
H	2.5744270000	2.4930240000	0.2749130000
C	1.6979730000	3.0511590000	-1.6488080000
H	1.9326210000	4.1103090000	-1.5185410000
N	0.4707030000	3.0241570000	-2.4403550000
H	0.1204060000	2.0858810000	-2.6187500000
H	-0.2803920000	3.5190020000	-1.9636230000
C	0.6314250000	3.3351730000	0.6765050000
C	1.1111790000	4.4758620000	1.4084630000
C	-0.7225560000	3.0547420000	0.6855310000
C	2.4878370000	4.8295670000	1.5040340000
C	0.1627600000	5.3045920000	2.1032570000
C	-1.6503070000	3.8718980000	1.3726790000
H	-1.0988730000	2.1816680000	0.1636630000
C	2.9005510000	5.9345970000	2.2172920000
H	3.2395210000	4.2166720000	1.0211160000
C	0.6221880000	6.4385020000	2.8268310000
C	-1.2190580000	4.9789340000	2.0620060000
H	-2.7057650000	3.6183680000	1.3344700000
C	1.9600410000	6.7548760000	2.8821460000
H	3.9591720000	6.1729880000	2.2738340000
H	-0.1116580000	7.0547620000	3.3411300000
H	-1.9244740000	5.6194580000	2.5858310000
H	2.2965450000	7.6249150000	3.4392120000
C	2.8547230000	2.3768250000	-2.3874800000
C	4.1928060000	2.8997490000	-2.3146240000
C	2.6329180000	1.2229880000	-3.1174630000
C	4.5364340000	4.1001490000	-1.6271930000
C	5.2560090000	2.1936980000	-2.9794370000
C	3.6797600000	0.5366640000	-3.7746800000
H	1.6322470000	0.8118310000	-3.2008190000
C	5.8340660000	4.5623350000	-1.5816120000
H	3.7670340000	4.6803430000	-1.1319970000
C	6.5838570000	2.6968100000	-2.9110210000
C	4.9679320000	1.0070730000	-3.7027380000
H	3.4541410000	-0.3671440000	-4.3340350000
C	6.8744740000	3.8530330000	-2.2250060000
H	6.0575710000	5.4838320000	-1.0508300000
H	7.3710320000	2.1445160000	-3.4190880000
H	5.7816050000	0.4842320000	-4.1995180000
H	7.8940900000	4.2259040000	-2.1818150000

Pre_Closure_RR

Imaginary Frequency (cm-1) : None

N	0.2870730000	1.0096150000	-0.4467150000
C	0.4050730000	2.4210490000	-0.8195040000
C	-0.9715410000	3.0103250000	-1.2031510000
H	1.0488210000	2.4363560000	-1.6967320000
C	1.0301150000	3.2675440000	0.2974210000
H	-1.6093350000	3.0653800000	-0.3183700000
C	-0.8695910000	4.3927790000	-1.9021060000
N	-1.7342310000	2.0710100000	-2.1356580000
C	2.3111790000	3.8952220000	0.1629950000
C	0.3461740000	3.4351850000	1.4879940000
C	-1.5051650000	4.3208680000	-3.3036310000
H	-1.3712520000	5.1512220000	-1.2937490000
H	0.1802600000	4.6924360000	-1.9858200000
C	-1.0266950000	1.8542300000	-3.4564950000
C	-3.1204330000	2.6296660000	-2.3812970000
C	3.1400030000	3.7931900000	-0.9906120000
C	2.7708080000	4.6790260000	1.2760540000
C	0.9074530000	4.2114650000	2.5250830000
H	-0.6311100000	2.9889210000	1.6493170000
C	-0.7901610000	3.2289130000	-4.1227540000
H	-1.4022530000	5.2884170000	-3.8035350000
C	-2.9950510000	3.9534850000	-3.1693820000
H	-0.1038170000	1.3069180000	-3.2541370000
H	-1.6838100000	1.2107000000	-4.0438470000
H	-3.5755170000	2.7604170000	-1.3991030000
H	-3.6697860000	1.8534470000	-2.9153530000
C	4.3562920000	4.4486200000	-1.0406030000
H	2.8394810000	3.1574580000	-1.8120680000
C	4.0246340000	5.3458360000	1.1795140000
N	2.0717370000	4.8247620000	2.4359520000
H	0.3587850000	4.3343310000	3.4576610000
H	0.2826170000	3.4413560000	-4.1781800000
H	-1.1700130000	3.2119730000	-5.1490060000
H	-3.4472220000	3.8490670000	-4.1609420000
C	4.7971620000	5.2420150000	0.0543050000
O	5.2223090000	4.4028390000	-2.0884450000
H	4.3446420000	5.9355690000	2.0326560000
H	5.7565360000	5.7432810000	-0.0274450000
C	4.8805220000	3.5959140000	-3.2119960000
H	4.7522510000	2.5459850000	-2.9241190000
H	5.7151750000	3.6872750000	-3.9085330000
H	3.9618090000	3.9543760000	-3.6933990000
N	1.2021910000	-1.0407760000	-0.0433740000
H	-0.1633580000	0.8271670000	0.4533720000
H	0.5347420000	-1.0408800000	0.7272570000
H	-1.8433850000	1.1642380000	-1.6600460000
C	1.2132030000	0.0557650000	-0.8243740000
S	-2.1838050000	-0.9721850000	1.8758080000
O	-2.2229450000	-2.3251850000	1.2699090000
O	-0.8416520000	-0.2966660000	1.9796540000
C	-2.7067840000	-1.1645260000	3.5920060000
C	-3.4378780000	-2.2990280000	3.9519760000
C	-2.4160840000	-0.1619690000	4.5217560000
C	-3.8836790000	-2.4290510000	5.2682120000
H	-3.6342690000	-3.0705000000	3.2153610000
C	-2.8703420000	-0.3029530000	5.8329850000
H	-1.8339470000	0.7044810000	4.2275040000
C	-3.6037680000	-1.4326020000	6.2062100000
H	-4.4445770000	-3.3121980000	5.5603520000
H	-2.6455930000	0.4680840000	6.5641800000
H	-3.9522140000	-1.5381890000	7.2296610000
C	-3.2083700000	1.4190580000	1.2824780000
N	-3.1330010000	2.5803760000	1.4583160000
C	-3.2835590000	0.0495670000	1.0357700000
C	-4.2724710000	-0.5413920000	0.0836170000
O	-3.6513720000	-0.3599430000	-1.2317440000
O	-4.5335110000	-0.8896940000	-2.2901170000
C	-3.9814760000	-2.0991990000	-2.8794700000
C	-2.5892410000	-1.8338740000	-3.4588350000
H	-2.2161750000	-2.7240930000	-3.9764580000
H	-2.6257820000	-1.0110030000	-4.1818390000
H	-1.8775680000	-1.5837850000	-2.6672910000
C	-5.0000080000	-2.3730000000	-3.9952220000
H	-6.0009750000	-2.5232120000	-3.5782190000
H	-5.0384690000	-1.5369350000	-4.7010820000
H	-4.7139690000	-3.2767170000	-4.5432320000

C	-3.9561300000	-3.2509900000	-1.8692650000
H	-4.9541360000	-3.4143320000	-1.4481840000
H	-3.6406050000	-4.1756180000	-2.3658870000
H	-3.2565280000	-3.0492550000	-1.0535680000
H	-4.3403460000	-1.6160770000	0.2666510000
H	-3.5372800000	4.7468450000	-2.6461330000
C	-5.6652990000	0.0671190000	0.1938090000
C	-6.1632240000	1.0219990000	-0.6966140000
C	-6.4727820000	-0.3390340000	1.2659120000
C	-7.4381570000	1.5659120000	-0.5158440000
H	-5.5662190000	1.3227640000	-1.5483410000
C	-7.7440550000	0.2020550000	1.4488140000
H	-6.0951670000	-1.0780050000	1.9693750000
C	-8.2324920000	1.1607570000	0.5564260000
H	-7.8102500000	2.3057740000	-1.2200490000
H	-8.3540900000	-0.1254470000	2.2865740000
H	-9.2238720000	1.5834950000	0.6953690000
S	2.2233380000	0.2285200000	-2.1849500000
C	2.1530810000	-2.1452830000	-0.1030890000
H	3.0191070000	-1.7581450000	-0.6390310000
C	2.6187930000	-2.5077500000	1.3719920000
H	3.2504280000	-3.3902900000	1.2504040000
N	1.5507010000	-2.9223010000	2.2829700000
H	0.9010940000	-2.1694120000	2.4991810000
H	1.0047260000	-3.6698370000	1.8580960000
C	1.6083620000	-3.3708360000	-0.8317100000
C	2.4855750000	-4.2296440000	-1.5810260000
C	0.2710430000	-3.7073340000	-0.7201480000
C	3.8707250000	-3.9621600000	-1.7812860000
C	1.9462330000	-5.4213770000	-2.1788560000
C	-0.2524930000	-4.8805190000	-1.3139750000
H	-0.4094160000	-3.0625220000	-0.1729190000
C	4.6719620000	-4.8168770000	-2.5076910000
H	4.3165810000	-3.0657130000	-1.3668560000
C	2.8008500000	-6.2814210000	-2.9205700000
C	0.5670330000	-5.7232290000	-2.0241610000
H	-1.3081460000	-5.1078260000	-1.1957190000
C	4.1364660000	-5.9930080000	-3.0815470000
H	5.7236420000	-4.5806260000	-2.6446730000
H	2.3734760000	-7.1786480000	-3.3622750000
H	0.1749020000	-6.6296540000	-2.4791680000
H	4.7780040000	-6.6597280000	-3.6510890000
C	3.4546210000	-1.3761020000	1.9679690000
C	4.8877820000	-1.3611960000	1.8413660000
C	2.8335760000	-0.3187660000	2.6091440000
C	5.6408120000	-2.4039870000	1.2267280000
C	5.6220970000	-0.2417300000	2.3693920000
C	3.5598680000	0.7785870000	3.1263190000
H	1.7545680000	-0.3042490000	2.7258470000
C	7.0142710000	-2.3439060000	1.1285020000
H	5.1364290000	-3.2756730000	0.8268430000
C	7.0384220000	-0.2112330000	2.2507860000
C	4.9270960000	0.8197570000	3.0054850000
H	3.0248180000	1.5902080000	3.6112840000
C	7.7258810000	-1.2352100000	1.6419780000
H	7.5547030000	-3.1595910000	0.6558030000
H	7.5709360000	0.6459110000	2.6562750000
H	5.4954560000	1.6613460000	3.3936150000
H	8.8085690000	-1.1983430000	1.5588320000

Pre_closure_SS

Imaginary Frequency (cm-1) : None

N	-0.9897460000	-1.1765810000	-0.2399280000
C	-1.2828690000	-2.4739490000	-0.8370640000
C	0.0136930000	-3.2502360000	-1.2057440000
H	-1.8228100000	-2.2473270000	-1.7546980000
C	-2.1416330000	-3.3602920000	0.0724160000
H	0.4589780000	-3.6550260000	-0.2973450000
C	-0.2239720000	-4.3665370000	-2.2580720000
N	1.0925800000	-2.3230150000	-1.7405330000
C	-3.4625690000	-3.7818400000	-0.2821800000
C	-1.6285610000	-3.7837400000	1.2852810000
C	0.6886590000	-4.1326040000	-3.4788890000
H	-0.0215820000	-5.3461580000	-1.8144800000
H	-1.2730160000	-4.3688950000	-2.5722580000
C	0.6940830000	-1.6516510000	-3.0325740000
C	2.3803530000	-3.0964270000	-1.9364180000
C	-4.1384950000	-3.3853100000	-1.4711520000
C	-4.1316280000	-4.6620580000	0.6357140000
C	-2.3888510000	-4.6258610000	2.1242490000
H	-0.6371030000	-3.4810310000	1.6125920000
C	0.3845710000	-2.7464860000	-4.0815010000
H	0.5064760000	-4.9108400000	-4.2258380000
C	2.1609340000	-4.1698140000	-3.0272720000
H	-0.1626150000	-1.0077390000	-2.8286260000
H	1.5382420000	-1.0198570000	-3.3102810000
H	2.6301090000	-3.5178310000	-0.9622170000
H	3.1429250000	-2.3574780000	-2.1927070000
C	-5.4091310000	-3.8572480000	-1.7435840000
H	-3.6774730000	-2.6661790000	-2.1345840000
C	-5.4333500000	-5.1368460000	0.3090530000
N	-3.5915440000	-5.0715050000	1.8168250000
H	-1.9746130000	-4.9497490000	3.0778820000
H	-0.6661790000	-2.6886400000	-4.3839840000
H	0.9887140000	-2.5842610000	-4.9796250000
H	2.8205380000	-3.9821140000	-3.8806870000
C	-6.0562710000	-4.7535980000	-0.8479280000
O	-6.1434170000	-3.5262610000	-2.8384120000
H	-5.9142220000	-5.8067220000	1.0147130000
H	-7.0513970000	-5.1045000000	-1.1026840000
C	-5.5892410000	-2.5984590000	-3.7679050000
H	-4.6709950000	-2.9921170000	-4.2214890000
H	-5.3729620000	-1.6363870000	-3.2890690000
H	-6.3475620000	-2.4628900000	-4.5402210000
N	-1.2401510000	1.0460690000	0.1701560000
H	-0.5654650000	-1.1799810000	0.6846160000
H	-0.5618180000	0.8581690000	0.9088380000
H	1.2727060000	-1.6276940000	-1.0009680000
C	-1.5606560000	0.0149380000	-0.6326200000
S	1.9413330000	-0.7105550000	1.8458210000
O	0.6317070000	-0.0564350000	2.1723800000
O	1.8767400000	-1.9063400000	0.9451390000
C	2.5285480000	-1.3433300000	3.4316040000
C	3.1913670000	-2.5716800000	3.4844220000
C	2.3638570000	-0.5573590000	4.5768170000
C	3.6912160000	-3.0212540000	4.7078590000
H	3.2978270000	-3.1675130000	2.5848180000
C	2.8700620000	-1.0174120000	5.7917880000
H	1.8409340000	0.3912640000	4.5196490000
C	3.5332860000	-2.2461830000	5.8587300000
H	4.2008310000	-3.9791770000	4.7602990000
H	2.7424170000	-0.4161580000	6.6871570000
H	3.9240560000	-2.5998430000	6.8085390000
C	2.9119660000	1.7360900000	1.6693560000
N	2.8011770000	2.8334580000	2.0704810000
C	3.0206890000	0.4303660000	1.1748830000
C	4.1323610000	-0.0517430000	0.2869600000
O	3.7506210000	0.3548050000	-1.0667710000
O	4.6211450000	-0.4124820000	-1.9886820000
C	4.9944350000	0.4065470000	-3.1264920000
C	3.7507350000	0.8213410000	-3.9219050000
H	4.0358590000	1.4440750000	-4.7769330000
H	3.2283460000	-0.0599020000	-4.3106940000
H	3.0602440000	1.3956420000	-3.2979500000
C	5.8633850000	-0.5672310000	-3.9346480000
H	6.7353570000	-0.8844890000	-3.3540760000
H	5.2917680000	-1.4561560000	-4.2224210000
H	6.2152650000	-0.0752390000	-4.8474300000
C	5.8087460000	1.6291310000	-2.6964960000
H	5.2019630000	2.3128300000	-2.0985180000
H	6.6746730000	1.3274800000	-2.1012920000
H	6.1630300000	2.1689130000	-3.5817580000
H	4.1220740000	-1.1459690000	0.2902360000
H	2.4126280000	-5.1598840000	-2.6343390000
C	5.5284350000	0.4074810000	0.6756500000
C	6.4881160000	-0.5543790000	1.0142930000
C	5.8880120000	1.7617300000	0.7320250000
C	7.7739580000	-0.1800920000	1.4106050000
H	6.2261860000	-1.6092840000	0.9685200000
C	7.1716720000	2.1392540000	1.1257090000
H	5.1633000000	2.5252150000	0.4707120000
C	8.1195250000	1.1706280000	1.4677430000
H	8.5032840000	-0.9431960000	1.6699800000
H	7.4318080000	3.1936570000	1.1671430000
H	9.1185880000	1.4680920000	1.7751680000
S	-2.5297970000	0.1711870000	-2.0238840000
C	-1.6793390000	2.4268310000	0.0067500000
H	-2.6824560000	2.3767770000	-0.4177950000
C	-1.7881070000	3.1058930000	1.4391350000
H	-2.0385880000	4.1477180000	1.2276910000
N	-0.5499860000	3.1574030000	2.2129960000
H	-0.1945640000	2.2400890000	2.4712060000
H	0.1935250000	3.6087940000	1.6837550000
C	-0.7696660000	3.2287050000	-0.9225210000
C	-1.2891320000	4.3107030000	-1.7139840000
C	0.5859340000	2.9600570000	-0.9625270000
C	-2.6720120000	4.6464660000	-1.7787170000
C	-0.3773400000	5.0942720000	-2.5034000000
C	1.4777020000	3.7326380000	-1.7428030000
H	0.9908750000	2.1291520000	-0.3949380000
C	-3.1240840000	5.6935690000	-2.5529710000
H	-3.3973430000	4.0662540000	-1.2207770000
C	-0.8767770000	6.1688430000	-3.2883350000
C	1.0084360000	4.7828310000	-2.4933810000
H	2.5365850000	3.4906800000	-1.7288370000
C	-2.2191300000	6.4698910000	-3.3131040000
H	-4.1864290000	5.9198180000	-2.5834520000
H	-0.1703320000	6.7517930000	-3.8747620000
H	1.6861690000	5.3884420000	-3.0902300000
H	-2.5863840000	7.2943680000	-3.9179910000
C	-2.9244260000	2.4783510000	2.2476460000
C	-4.2597040000	3.0117340000	2.2012000000
C	-2.6856860000	1.3558240000	3.0203720000
C	-4.6204710000	4.1817110000	1.4710680000
C	-5.3024580000	2.3484090000	2.9389140000

C -3.7121900000 0.7114400000 3.7480350000
H -1.6867810000 0.9367150000 3.0840660000
C -5.9149520000 4.6544540000 1.4542160000
H -3.8672300000 4.7293460000 0.9174000000
C -6.6276100000 2.8616610000 2.8987760000
C -4.9975000000 1.1929910000 3.7044740000
H -3.4733160000 -0.1692000000 4.3379350000
C -6.9351780000 3.9875060000 2.1710280000
H -6.1514500000 5.5517490000 0.8887290000
H -7.3987750000 2.3417760000 3.4625340000
H -5.7961170000 0.7027520000 4.2559240000
H -7.9524360000 4.3686450000 2.1501680000

TS2-RR

Imaginary Frequency (cm-1) : -265.18

N 0.0060350000 -0.8330010000 -0.5300730000
C 0.0711630000 -2.2861170000 -0.6800340000
C 1.4712390000 -2.7731130000 -1.1305270000
H -0.6474410000 -2.5295210000 -1.4593010000
C -0.3255730000 -3.0064560000 0.6204320000
H 2.2126410000 -2.4918130000 -0.3796110000
C 1.4800540000 -4.3135260000 -1.3783400000
N 1.9639080000 -2.1040620000 -2.4031780000
C -1.5191970000 -3.7924900000 0.7349590000
C 0.4891700000 -2.9063640000 1.7322940000
C 1.7790920000 -4.6139150000 -2.8587690000
H 2.2258010000 -4.7915940000 -0.7354150000
H 0.5100410000 -4.7389570000 -1.1062280000
C 1.0245110000 -2.3204550000 -3.5613030000
C 3.3282440000 -2.6612250000 -2.7555580000
C -2.4702710000 -3.9661420000 -0.3105030000
C -1.7504960000 -4.4470020000 1.9930600000
C 0.1488630000 -3.5731570000 2.9291350000
H 1.4084860000 -2.3304240000 1.7179930000
C 0.7820800000 -3.8375270000 -3.7414840000
H 1.6842250000 -5.6884970000 -3.0419890000
C 3.2054560000 -4.1387970000 -3.1805130000
H 0.1091300000 -1.7591310000 -3.3625510000
H 1.5131610000 -1.8714410000 -4.4286440000
H 3.9528500000 -2.5189900000 -1.8721230000
H 3.7166490000 -2.0114970000 -3.5394000000
C -3.5853790000 -4.7612030000 -0.1206530000
H -2.3496020000 -3.4248180000 -1.2386630000
C -2.9048200000 -5.2659730000 2.1433760000
N -0.9233050000 -4.3283590000 3.0694550000
H 0.8040640000 -3.4829170000 3.7942030000
H -0.2457400000 -4.0967140000 -3.4643220000
H 0.9090610000 -4.1108150000 -4.7939120000
H 3.4116650000 -4.2508710000 -4.2504770000
C -3.7972990000 -5.4273360000 1.1177750000
O -4.5547610000 -4.9762730000 -1.0500470000
H -3.0506440000 -5.7541710000 3.1017860000
H -4.6821780000 -6.0471910000 1.2235950000
C -4.4454330000 -4.3104560000 -2.3054020000
H -4.4296700000 -3.2218560000 -2.1787460000
H -5.3290590000 -4.6026230000 -2.8746290000
H -3.5428030000 -4.6255760000 -2.8442780000
N -1.1994030000 1.0743380000 -0.1858370000
H 0.5693420000 -0.4507280000 0.2275920000
H -0.4990970000 1.2363750000 0.5349340000
H 2.1528020000 -1.0530550000 -2.3005120000
C -1.1060930000 -0.0923930000 -0.8600550000
S 2.2377930000 1.6865420000 1.8754080000
O 2.3118200000 2.9411270000 1.1048010000
O 0.9416690000 0.9441950000 1.9093770000
C 2.6661880000 2.0340240000 3.5809770000
C 3.4613640000 3.1508780000 3.8540550000
C 2.2124140000 1.1850710000 4.5942970000
C 3.8087850000 3.4183110000 5.1774610000
H 3.7843980000 3.8001850000 3.0475500000
C 2.5721200000 1.4653910000 5.9125940000
H 1.5824040000 0.3353580000 4.3568350000
C 3.3680560000 2.5763870000 6.2024970000
H 4.4191010000 4.2863450000 5.4076810000
H 2.2248340000 0.8180450000 6.7120120000
H 3.6420360000 2.7900080000 7.2315480000
C 3.6338760000 -0.6486890000 1.9110860000
N 3.7276070000 -1.6753070000 2.4648780000
C 3.4981080000 0.5935380000 1.2711720000
C 4.2374940000 0.9651230000 0.0406550000
O 3.1036870000 0.3819540000 -0.5771920000
O 3.1011860000 0.2950400000 -2.5617270000
C 2.6934680000 1.4569100000 -3.2577610000
C 1.1771760000 1.6792070000 -3.1480130000
H 0.8628510000 2.5846630000 -3.6810730000
H 0.6232070000 0.8328290000 -3.5703730000
H 0.8864580000 1.7863960000 -2.1001990000
C 3.0821500000 1.2148630000 -4.7365670000
H 4.1635270000 1.0659310000 -4.8291750000
H 2.5764860000 0.3247300000 -5.1289070000
H 2.7941240000 2.0697090000 -5.3610730000
C 3.4392090000 2.7028420000 -2.7434940000
H 4.5210000000 2.5272760000 -2.7375870000
H 3.2365430000 3.5716210000 -3.3811840000
H 3.1160900000 2.9499860000 -1.7276040000
H 4.2670470000 2.0481880000 -0.1106520000
H 3.9421880000 -4.7439290000 -2.6427040000
C 5.5978020000 0.3326120000 -0.1671520000
C 5.9436000000 -0.2353170000 -1.3989090000
C 6.5504590000 0.3845130000 0.8589100000
C 7.2250930000 -0.7574690000 -1.5917570000
H 5.2008730000 -0.2409920000 -2.1900140000
C 7.8297870000 -0.1364650000 0.6615860000
H 6.2900260000 0.8283470000 1.8166160000
C 8.1700320000 -0.7119370000 -0.5648840000
H 7.4869120000 -1.1975340000 -2.5508470000
H 8.5578420000 -0.0965600000 1.4673130000
H 9.1655370000 -1.1199890000 -0.7182430000
S -2.2160580000 -0.5837130000 -2.0552800000
C -2.3098260000 2.0197790000 -0.2496910000
H -3.0832250000 1.5124260000 -0.8239750000
C -2.8800130000 2.2751940000 1.2116200000
H -3.6561470000 3.0326140000 1.0778550000
N -1.9319130000 2.8639640000 2.1600510000
H -1.1287890000 2.2631720000 2.3348390000
H -1.5666590000 3.7346130000 1.7783290000
C -1.9396130000 3.3354240000 -0.9298590000
C -2.9005510000 4.0434070000 -1.7325570000
C -0.6944220000 3.9023240000 -0.7206480000
C -4.1989680000 3.5408450000 -2.0366760000
C -2.5406900000 5.3250330000 -2.2779140000
C -0.3443170000 5.1597120000 -1.2665850000
H 0.0557610000 3.3771410000 -0.1384640000
C -5.0866480000 4.2582180000 -2.8095040000
H -4.5054730000 2.5687480000 -1.6690440000
C -3.4809570000 6.0384830000 -3.0699270000
C -1.2502230000 5.8608590000 -2.0240010000
H 0.6465630000 5.5623100000 -1.0762880000
C -4.7300070000 5.5237430000 -3.3297940000
H -6.0672250000 3.8431820000 -3.0258280000
H -3.1892190000 7.0064870000 -3.4705980000
H -0.9937090000 6.8307360000 -2.4432650000
H -5.4384660000 6.0802750000 -3.9370910000
C -3.5192400000 1.0082990000 1.7750090000
C -4.9078150000 0.7102350000 1.5467940000
C -2.7559210000 0.1065170000 2.4939160000
C -5.7977690000 1.5747890000 0.8454810000
C -5.4493390000 -0.5209600000 2.0576740000
C -3.2929040000 -1.1008960000 2.9968710000
H -1.7071440000 0.3088960000 2.6858470000
C -7.1218800000 1.2458610000 0.6500190000
H -5.4408070000 2.5205850000 0.4549980000
C -6.8194210000 -0.8298140000 1.8377680000
C -4.6125990000 -1.4122790000 2.7788450000
H -2.6505230000 -1.7811050000 3.5485090000
C -7.6426640000 0.0288180000 1.1470220000
H -7.7718560000 1.9311110000 0.1125850000
H -7.2061450000 -1.7668030000 2.2317390000
H -5.0334250000 -2.3426210000 3.1518860000
H -8.6879880000 -0.2205950000 0.9868960000

TS2-SS

Imaginary Frequency (cm-1) : -304.57

N -0.3931390000 -0.9221680000 -0.3619220000
C -0.8035670000 -2.2877290000 -0.6758560000
C 0.4172990000 -3.1325810000 -1.1187170000
H -1.4989810000 -2.2026680000 -1.5070390000
C -1.5171920000 -2.9654590000 0.5071140000
H 1.1782860000 -3.0817100000 -0.3361010000
C 0.0411660000 -4.6135950000 -1.4325410000
N 1.1084790000 -2.5666510000 -2.3531480000
C -2.8852930000 -3.3892160000 0.4393130000
C -0.8286800000 -3.1940970000 1.6841100000
C 0.2952960000 -4.9286750000 -2.9177340000
H 0.6221910000 -5.2881310000 -0.7954570000
H -1.0117780000 -4.7876680000 -1.1967170000
C 0.1790300000 -2.5009620000 -3.5404940000
C 2.2928700000 -3.4466290000 -2.6977700000
C -3.7325210000 -3.1956230000 -0.6891130000
C -3.4194930000 -4.0563330000 1.5947010000
C -1.4639900000 -3.8394620000 2.7668240000
H 0.2005970000 -2.8744890000 1.8040330000
C -0.4527090000 -3.8914950000 -3.7762740000
H -0.0640940000 -5.9364530000 -3.1464450000
C 1.8030530000 -4.8198620000 -3.2015830000
H -0.5543180000 -1.7148850000 -3.3501950000
H 0.7972430000 -2.1841760000 -4.3810790000
H 2.8933900000 -3.5203440000 -1.7886360000
H 2.8729320000 -2.8875340000 -3.4309700000
C -5.0372620000 -3.6524180000 -0.6751520000
H -3.3726350000 -2.6311940000 -1.5379380000
C -4.7642570000 -4.5221990000 1.5635180000

N	-2.7096400000	-4.2719700000	2.7372020000
H	-0.9088630000	-4.0104100000	3.6883150000
H	-1.5159160000	-3.8831590000	-3.5120800000
H	-0.3875060000	-4.1501730000	-4.8379290000
H	1.9965450000	-4.9281840000	-4.2742920000
C	-5.5536820000	-4.3342030000	0.4611260000
O	-5.9256810000	-3.4999690000	-1.6934450000
H	-5.1392530000	-5.0276330000	2.4478260000
H	-6.5816920000	-4.6814280000	0.4293630000
C	-5.5084170000	-2.7824270000	-2.8517700000
H	-4.6776300000	-3.2921230000	-3.3563740000
H	-5.2061180000	-1.7595680000	-2.5990280000
H	-6.3749090000	-2.7573090000	-3.5140660000
N	-0.8184420000	1.2743000000	0.0987560000
H	0.2743570000	-0.8546840000	0.4034330000
H	-0.0769980000	1.1619950000	0.7878560000
H	1.5531470000	-1.5955580000	-2.2215160000
C	-1.1550770000	0.1889710000	-0.6307580000
S	2.3434230000	-0.0389760000	2.1993160000
O	1.3347110000	1.0512690000	2.2435840000
O	1.8803290000	-1.3871070000	1.7917060000
C	3.0521920000	-0.1601720000	3.8393660000
C	3.5559230000	-1.3917260000	4.2683930000
C	3.1097320000	0.9822800000	4.6428470000
C	4.1278930000	-1.4755990000	5.5369640000
H	3.4857210000	-2.2637430000	3.6274140000
C	3.6888030000	0.8809770000	5.9077540000
H	2.6989820000	1.9220630000	4.2911830000
C	4.1967360000	-0.3422620000	6.3522870000
H	4.5159890000	-2.4265240000	5.8892510000
H	3.7375020000	1.7578320000	6.5459870000
H	4.6443440000	-0.4140400000	7.3392190000
C	4.1806400000	1.7112150000	1.2537440000
N	4.5881980000	2.7951080000	1.4136490000
C	3.6592350000	0.4122250000	1.1074630000
C	4.0780860000	-0.5545390000	0.0612150000
O	3.0818640000	0.1526050000	-0.6507660000
O	2.8065210000	-0.5007590000	-2.5176860000
C	2.6869330000	0.6343030000	-3.3605210000
C	1.3499760000	1.3573820000	-3.1276060000
H	1.2409520000	2.2254640000	-3.7895200000
H	0.5015090000	0.6880820000	-3.3106370000
H	1.2852330000	1.7095360000	-2.0947580000
C	2.7577690000	0.1229290000	-4.8196980000
H	3.6883860000	-0.4312190000	-4.9866960000
H	1.9147760000	-0.5361470000	-5.0548720000
H	2.7262030000	0.9618050000	-5.5259480000
C	3.8501510000	1.6238530000	-3.1451190000
H	3.8564960000	1.9889420000	-2.1159510000
H	4.8142320000	1.1442890000	-3.3421670000
H	3.7515770000	2.4827710000	-3.8199620000
H	3.7518250000	-1.5740540000	0.2989780000
H	2.3491190000	-5.6198030000	-2.6915660000
C	5.5181880000	-0.5491920000	-0.4062760000
C	5.8293530000	-0.9115040000	-1.7237420000
C	6.5579900000	-0.3076150000	0.4997930000
C	7.1610740000	-1.0074170000	-2.1290790000
H	5.0138150000	-1.0912670000	-2.4160860000
C	7.8902340000	-0.4071290000	0.0921450000
H	6.3308850000	-0.0423750000	1.5284850000
C	8.1955660000	-0.7552240000	-1.2241880000
H	7.3919920000	-1.2802990000	-3.1557390000
H	8.6865630000	-0.2093240000	0.8045620000
H	9.2318350000	-0.8302130000	-1.5429210000
S	-2.3719580000	0.1873670000	-1.8248500000
C	-1.4928980000	2.5707620000	0.0701880000
H	-2.3246190000	2.4361550000	-0.6191010000
C	-2.1040620000	2.9073150000	1.4984810000
H	-2.5950170000	3.8756340000	1.3699230000
N	-1.1264190000	3.1143460000	2.5691610000
H	-0.5179190000	2.3082960000	2.6974230000
H	-0.5133510000	3.8862650000	2.3134820000
C	-0.5873360000	3.6975100000	-0.4209070000
C	-1.1161810000	4.7715470000	-1.2175890000
C	0.7446450000	3.7380160000	-0.0448110000
C	-2.4628320000	4.8222000000	-1.6812330000
C	-0.2459000000	5.8567620000	-1.5853380000
C	1.5986270000	4.8022020000	-0.4177620000
H	1.1696990000	2.9324040000	0.5441070000
C	-2.9248050000	5.8760670000	-2.4398150000
H	-3.1472120000	4.0140820000	-1.4509350000
C	-0.7558420000	6.9281310000	-2.3682290000
C	1.1115300000	5.8446640000	-1.1677130000
H	2.6370610000	4.7804670000	-0.0991910000
C	-2.0666260000	6.9455950000	-2.7854860000
H	-3.9566200000	5.8809820000	-2.7803830000
H	-0.0818840000	7.7393760000	-2.6334840000
H	1.7555810000	6.6719080000	-1.4559000000
H	-2.4422180000	7.7713760000	-3.3834170000
C	-3.1531460000	1.8704050000	1.8913200000
C	-4.5258610000	2.0068840000	1.4860450000
C	-2.7838990000	0.7555430000	2.6212450000
C	-5.0272440000	3.1261590000	0.7603630000
C	-5.4619630000	0.9714900000	1.8341320000
C	-3.7064980000	-0.2605630000	2.9609940000
H	-1.7565930000	0.6384230000	2.9508320000
C	-6.3525420000	3.2147520000	0.3927900000
H	-4.3631050000	3.9392760000	0.4911240000
C	-6.8219080000	1.0936720000	1.4385700000
C	-5.0194500000	-0.1584580000	2.5705300000
H	-3.3660790000	-1.1238000000	3.5259510000
C	-7.2635280000	2.1871590000	0.7303980000
H	-6.7007000000	4.0845930000	-0.1576910000
H	-7.5115770000	0.2981360000	1.7111480000
H	-5.7358420000	-0.9378350000	2.8180860000
H	-8.3061560000	2.2670340000	0.4351950000

Epoxide_RR

Imaginary Frequency (cm-1) : None

N	0.0489370000	0.9925270000	-0.6072690000
C	-0.0199130000	2.4572570000	-0.6025410000
C	-1.3201080000	3.0182300000	-1.2339100000
H	0.8215900000	2.7808340000	-1.2098590000
C	0.1426090000	2.9890310000	0.8309410000
H	-2.1644370000	2.7105120000	-0.6085560000
C	-1.2569040000	4.5840160000	-1.2898700000
N	-1.6194560000	2.4615690000	-2.5795920000
C	1.2864240000	3.7382400000	1.2596550000
C	-0.8423460000	2.7340860000	1.7678660000
C	-1.5450070000	5.0453210000	-2.7303890000
H	-1.9823390000	5.0212430000	-0.5944140000
H	-0.2690970000	4.9479200000	-0.9832820000
C	-0.6028150000	2.8764220000	-3.5767760000
C	-2.9409380000	2.9878500000	-3.0059950000
C	2.3941480000	4.0536580000	0.4221800000
C	1.2992990000	4.1939280000	2.6221680000
C	-0.7149040000	3.2093860000	3.0902400000
H	-1.7298180000	2.1668260000	1.5044400000
C	-0.4874750000	4.4276590000	-3.6654330000
H	-1.5056700000	6.1391080000	-2.7842500000
C	-2.9366470000	4.5381440000	-3.1461340000
H	0.3510840000	2.4107870000	-3.3156490000
H	-0.9102310000	2.4511490000	-4.5383210000
H	-3.6816700000	2.6578160000	-2.2709070000
H	-3.1934030000	2.5053560000	-3.9553320000
C	3.4534780000	4.7961240000	0.9088350000
H	2.4270310000	3.6662750000	-0.5866440000
C	2.4037890000	4.9660180000	3.0806960000
N	0.3084090000	3.9236650000	3.5182980000
H	-1.4990200000	2.9932840000	3.8151200000
H	0.5143090000	4.7612900000	-3.3673440000
H	-0.6452830000	4.7717720000	-4.6947800000
H	-3.1546800000	4.8381100000	-4.1783870000
C	3.4512620000	5.2660460000	2.2512190000
O	4.5598520000	5.1359800000	0.1932280000
H	2.3847990000	5.3046010000	4.1118930000
H	4.2992020000	5.8519280000	2.5923770000
C	4.6610930000	4.6703350000	-1.1501300000
H	4.6369170000	3.5753590000	-1.1947930000
H	5.6218150000	5.0330380000	-1.5186680000
H	3.8537080000	5.0759690000	-1.7727280000
N	1.1836780000	-0.9816730000	-0.3728620000
H	-0.6804750000	0.5202840000	-0.0804240000
H	0.4406460000	-1.2117530000	0.2810650000
H	-2.1788380000	0.5710120000	-2.6429970000
C	1.1798560000	0.2777310000	-0.8824250000
S	-2.2629350000	-2.1152100000	1.6342750000
O	-2.4539170000	-2.9567690000	0.4433750000
O	-0.9509480000	-1.4663700000	1.8658810000
C	-2.7299360000	-3.0287010000	3.0914980000
C	-3.6887230000	-4.0393680000	2.9638120000
C	-2.1264920000	-2.7208560000	4.3146980000
C	-4.0533260000	-4.7553300000	4.1025830000
H	-4.1226490000	-4.2670030000	1.9962290000
C	-2.5026710000	-3.4505290000	5.4413040000
H	-1.3751270000	-1.9417050000	4.3770850000
C	-3.4633170000	-4.4599270000	5.3349270000
H	-4.7917600000	-5.5471620000	4.0254620000
H	-2.0424800000	-3.2317100000	6.3995720000
H	-3.7506970000	-5.0236440000	6.2174920000
C	-3.8851400000	-0.1460010000	2.7702610000
N	-4.1725270000	0.2633870000	3.8183440000
C	-3.4671900000	-0.6783900000	1.4905040000
C	-4.2001310000	-0.5557440000	0.2068980000
O	-3.0126760000	0.2484690000	0.5345930000
O	-2.7213040000	-0.2557870000	-2.6163430000
C	-2.4315520000	-1.0723910000	-3.7650710000
C	-0.9135290000	-1.2449270000	-3.9167810000
H	-0.6730190000	-1.9014690000	-4.7611700000
H	-0.4257050000	-0.2792060000	-4.0939980000
H	-0.4835100000	-1.6806130000	-3.0100820000
C	-3.0303080000	-0.4337060000	-5.0297560000
H	-4.1084450000	-0.2798250000	-4.9073920000
H	-2.5658990000	0.5384140000	-5.2338030000
H	-2.8713770000	-1.0692080000	-5.9091730000

C	-3.1024970000	-2.4213140000	-3.4843810000
H	-4.1840640000	-2.2921680000	-3.3591120000
H	-2.9349540000	-3.1226980000	-4.3091020000
H	-2.6995900000	-2.8614360000	-2.5659130000
H	-3.9415810000	-1.3123440000	-0.5284370000
H	-3.7088290000	4.9891880000	-2.5111460000
C	-5.4995680000	0.1427430000	-0.0001790000
C	-5.7413860000	0.6469370000	-1.2887950000
C	-6.4814720000	0.2685800000	0.9895630000
C	-6.9407640000	1.2994890000	-1.5683930000
H	-4.9804310000	0.5168560000	-2.0542570000
C	-7.6842690000	0.9161830000	0.7005600000
H	-6.3247270000	-0.1429430000	1.9807100000
C	-7.9131230000	1.4371600000	-0.5737180000
H	-7.1201510000	1.6937370000	-2.5646630000
H	-8.4421010000	1.0089430000	1.4730530000
H	-8.8497140000	1.9417290000	-0.7941450000
S	2.4550940000	0.9008210000	-1.8296090000
C	2.2871760000	-1.9329280000	-0.4811640000
H	3.0871460000	-1.3725630000	-0.9628510000
C	2.8101200000	-2.3650810000	0.9551850000
H	3.6248280000	-3.0639240000	0.7498080000
N	1.8584830000	-3.1215850000	1.7735720000
H	1.0166170000	-2.5875110000	1.9782700000
H	1.5557850000	-3.9453110000	1.2564370000
C	1.9396620000	-3.1580760000	-1.3231190000
C	2.9389080000	-3.7975190000	-2.1364170000
C	0.6749520000	-3.7165450000	-1.2569230000
C	4.2606120000	-3.2916620000	-2.3035770000
C	2.5965710000	-5.0058950000	-2.8378240000
C	0.3413860000	-4.9002550000	-1.9566030000
H	-0.1010970000	-3.2387110000	-0.6683700000
C	5.1861050000	-3.9411140000	-3.0917910000
H	4.5538580000	-2.3684290000	-1.8180330000
C	3.5761030000	-5.6509450000	-3.6409140000
C	1.2843870000	-5.5373680000	-2.7253720000
H	-0.6659190000	-5.2989110000	-1.8742060000
C	4.8462690000	-5.1372950000	-3.7651450000
H	6.1837910000	-3.5252080000	-3.2019800000
H	3.2975090000	-6.5645830000	-4.1608570000
H	1.0412660000	-6.4513560000	-3.2618190000
H	5.5846370000	-5.6407090000	-4.3830680000
C	3.3701460000	-1.1660620000	1.7164950000
C	4.7528530000	-0.7873370000	1.6010630000
C	2.5368240000	-0.4009890000	2.5121810000
C	5.7116640000	-1.5148370000	0.8378100000
C	5.2152370000	0.3833320000	2.2981890000
C	2.9955160000	0.7477350000	3.1968410000
H	1.4914490000	-0.6694340000	2.6238690000
C	7.0273070000	-1.1126270000	0.7557980000
H	5.4153280000	-2.4129130000	0.3085570000
C	6.5786790000	0.7712830000	2.1911180000
C	4.3079870000	1.1371360000	3.0876750000
H	2.2986530000	1.3228940000	3.7997320000
C	7.4700220000	0.0455940000	1.4355360000
H	7.7310560000	-1.6938700000	0.1660420000
H	6.9046060000	1.6611400000	2.7246350000
H	4.6686980000	2.0258310000	3.5993260000
H	8.5092710000	0.3543020000	1.3631360000

Epoxide_SS

Imaginary Frequency (cm-1) : None

N	-0.3356150000	-1.0881300000	-0.5402430000
C	-0.6240740000	-2.5209530000	-0.5939130000
C	0.6632820000	-3.3419710000	-0.8523740000
H	-1.3038490000	-2.6498280000	-1.4327960000
C	-1.3071420000	-2.9976310000	0.6981270000
H	1.3605650000	-3.1279590000	-0.0328790000
C	0.3698830000	-4.8780520000	-0.8802860000
N	1.3804650000	-2.9276740000	-2.0887680000
C	-2.5968440000	-3.6205160000	0.7170410000
C	-0.6546220000	-2.8371780000	1.9069270000
C	1.1115330000	-5.5029770000	-2.0762280000
H	0.6864590000	-5.3418710000	0.0602150000
H	-0.7047340000	-5.0709910000	-0.9813760000
C	0.6495350000	-3.3656430000	-3.3032100000
C	2.7147440000	-3.5793340000	-2.0804130000
C	-3.3945180000	-3.8419530000	-0.4420450000
C	-3.1009580000	-4.0479240000	1.9925280000
C	-1.2517260000	-3.2817490000	3.1053800000
H	0.3257350000	-2.3736450000	1.9626480000
C	0.5194780000	-4.9165720000	-3.3714660000
H	0.9897440000	-6.5916730000	-2.0622190000
C	2.6029480000	-5.1297650000	-1.9943040000
H	-0.3278910000	-2.8757380000	-3.3117900000
H	1.1984420000	-2.9800920000	-4.1682130000
H	3.2811520000	-3.1713920000	-1.2366950000
H	3.2315070000	-3.2699180000	-2.9942220000
C	-4.6236140000	-4.4650100000	-0.3410780000
H	-3.0528780000	-3.4787810000	-1.4007410000
C	-4.3695370000	-4.6912980000	2.0527100000
N	-2.4306710000	-3.8722080000	3.1660420000
H	-0.7233880000	-3.1469960000	4.0485460000
H	-0.5310600000	-5.2142150000	-3.4736570000
H	1.0519070000	-5.3146850000	-4.2438180000
H	3.1593000000	-5.6053910000	-2.8108040000
C	-5.1115060000	-4.9016290000	0.9215840000
O	-5.4610960000	-4.7103790000	-1.3859080000
H	-4.7250400000	-5.0074830000	3.0284020000
H	-6.0807180000	-5.3893310000	0.9576320000
C	-5.0656490000	-4.2727030000	-2.6823310000
H	-4.1420660000	-4.7697620000	-3.0052240000
H	-4.9206460000	-3.1861980000	-2.7090580000
H	-5.8809360000	-4.5505000000	-3.3519950000
N	-0.8908420000	1.1176020000	-0.4104140000
H	0.4378470000	-0.8066340000	0.0550180000
H	-0.1126750000	1.1520190000	0.2393120000
H	2.0788030000	-1.0798920000	-2.2414280000
C	-1.2038820000	-0.1057150000	-0.9152500000
S	2.4695370000	0.1454190000	2.3767560000
O	1.6287880000	0.7175570000	1.3007890000
O	2.3858640000	-1.2902940000	2.6870350000
C	2.2335960000	1.1041280000	3.8627740000
C	2.2543770000	0.4461710000	5.0953350000
C	2.0494580000	2.4876290000	3.7565140000
C	2.0727420000	1.2013780000	6.2536860000
H	2.3967800000	-0.6278110000	5.1391990000
C	1.8741310000	3.2249370000	4.9259590000
H	2.0407250000	2.9716660000	2.7862080000
C	1.8847590000	2.5830560000	6.1680010000
H	2.0768160000	0.7087770000	7.2208990000
H	1.7237990000	4.2981340000	4.8660360000
H	1.7435570000	3.1642290000	7.0743740000
C	5.1729120000	-0.0047010000	2.8476940000
N	5.9040360000	-0.4051380000	3.6558170000
C	4.2209650000	0.4808590000	1.8718060000
C	4.5293090000	0.6106830000	0.4119580000
O	4.4263550000	1.7510670000	1.3048440000
O	2.6695770000	-0.2869470000	-2.2224110000
C	2.6471150000	0.3723100000	-3.5049130000
C	1.1970330000	0.6624760000	-3.9177050000
H	1.1597050000	1.1975550000	-4.8738350000
H	0.6268760000	-0.2668320000	-4.0314700000
H	0.6987940000	1.2750570000	-3.1602950000
C	3.3467820000	-0.5029070000	-4.5583850000
H	4.3750260000	-0.7245660000	-4.2517720000
H	2.8171450000	-1.4526630000	-4.6961560000
H	3.3801820000	0.0019790000	-5.5310390000
C	3.4225490000	1.6793270000	-3.3128540000
H	2.9345980000	2.3052930000	-2.5588400000
H	4.4476700000	1.4747550000	-2.9852040000
H	3.4673570000	2.2449060000	-4.2499670000
H	3.6791050000	0.4931510000	-0.2610910000
H	3.0312180000	-5.5011790000	-1.0554160000
C	5.8687600000	0.2810420000	-0.1445740000
C	7.0470400000	0.8280760000	0.3811520000
C	5.9329370000	-0.6065550000	-1.2281360000
C	8.2805010000	0.4764040000	-0.1655060000
H	6.9964500000	1.5377460000	1.2007720000
C	7.1712580000	-0.9602010000	-1.7646150000
H	5.0104620000	-0.9921770000	-1.6526490000
C	8.3457260000	-0.4220780000	-1.2336870000
H	9.1909710000	0.9064930000	0.2417920000
H	7.2175970000	-1.6497020000	-2.6028860000
H	9.3089280000	-0.6951890000	-1.6555980000
S	-2.5252690000	-0.3837670000	-1.9510920000
C	-1.6620030000	2.3407550000	-0.5801850000
H	-2.5211160000	2.0495370000	-1.1834170000
C	-2.2022310000	2.8590990000	0.8215460000
H	-2.6798290000	3.8163560000	0.6027080000
N	-1.1589770000	3.1717130000	1.8049850000
H	-0.6235000000	2.3458910000	2.0641970000
H	-0.4980470000	3.8294880000	1.3937720000
C	-0.8778730000	3.4444360000	-1.2867770000
C	-1.5556120000	4.4040780000	-2.1170480000
C	0.4820290000	3.5915050000	-1.0714930000
C	-2.9426450000	4.3277850000	-2.4340420000
C	-0.8044180000	5.4952210000	-2.6769160000
C	1.2168630000	4.6654020000	-1.6285380000
H	1.0211760000	2.8608570000	-0.4762840000
C	-3.5507030000	5.2709960000	-3.2343730000
H	-3.5423810000	3.5088700000	-2.0547620000
C	-1.4634390000	6.4512560000	-3.4966390000
C	0.5860290000	5.6029160000	-2.4089030000
H	2.2821230000	4.7410720000	-1.4290060000
C	-2.8080200000	6.3488880000	-3.7692930000
H	-4.6093030000	5.1803700000	-3.4613540000
H	-0.8779280000	7.2698130000	-3.9085440000
H	1.1398560000	6.4349830000	-2.8365770000
H	-3.2986720000	7.0864170000	-4.3982880000
C	-3.2439540000	1.9085070000	1.4039320000
C	-4.6501880000	2.1094450000	1.1764790000
C	-2.8421520000	0.8128280000	2.1459610000
C	-5.1870300000	3.2122080000	0.4504110000
C	-5.5849580000	1.1570960000	1.7148720000

C	-3.7632720000	-0.1206250000	2.6736270000
H	-1.7875730000	0.6378010000	2.3334580000
C	-6.5437480000	3.3608670000	0.2585710000
H	-4.5258370000	3.9635060000	0.0349150000
C	-6.9778800000	1.3401740000	1.4981480000
C	-5.1092980000	0.0461910000	2.4591700000
H	-3.3965310000	-0.9713130000	3.2405780000
C	-7.4538560000	2.4149510000	0.7839490000
H	-6.9165850000	4.2157740000	-0.2991280000
H	-7.6645610000	0.6058920000	1.9128050000
H	-5.8278450000	-0.6679450000	2.8536050000
H	-8.5213030000	2.5416830000	0.6258650000

TS1-R_Naphtyl

Imaginary Frequency (cm-1) : -186.12

N	0.5872740000	0.9137120000	-0.0384880000
C	0.9175920000	2.3210440000	0.1682110000
C	-0.2587060000	3.2768610000	-0.1602660000
H	1.7293720000	2.5292720000	-0.5250800000
C	1.3915290000	2.5816330000	1.6085160000
H	-1.0465790000	3.1508200000	0.5863960000
C	0.2319000000	4.7558300000	-0.1929240000
N	-0.9397850000	2.9721470000	-1.4738450000
C	2.7387980000	2.9530580000	1.9272760000
C	0.4967990000	2.4457480000	2.6541870000
C	-0.1917760000	5.4225340000	-1.5157150000
H	-0.1888690000	5.3078360000	0.6531560000
H	1.3194290000	4.7916850000	-0.0864700000
C	-0.0210560000	3.1763660000	-2.6402200000
C	-2.1461930000	3.8639730000	-1.6240420000
C	3.7797030000	3.0974420000	0.9668640000
C	3.0401500000	3.1909780000	3.3121940000
C	0.9098110000	2.6830200000	3.9829960000
H	-0.5378990000	2.1621570000	2.4873350000
C	0.4107820000	4.6751540000	-2.7299350000
H	0.1284700000	6.4686760000	-1.5221050000
C	-1.7242240000	5.3499270000	-1.6250270000
H	0.8297680000	2.5025040000	-2.5157740000
H	-0.5771520000	2.8506490000	-3.5196280000
H	-2.8224090000	3.6186990000	-0.8042330000
H	-2.6298980000	3.5519950000	-2.5503500000
C	5.0510180000	3.4717110000	1.3604830000
H	3.5859540000	2.8619820000	-0.0702550000
C	4.3581590000	3.5884040000	3.6735410000
N	2.1309920000	3.0525380000	4.3177160000
H	0.1908180000	2.5715450000	4.7933730000
H	-0.0625960000	5.0939830000	-3.6305290000
H	-2.0663260000	5.8422870000	-2.5416330000
C	5.3387280000	3.7311510000	2.7287540000
O	6.1126260000	3.6186860000	0.5227250000
H	4.5557550000	3.7690290000	4.7254700000
H	6.3482810000	4.0287720000	2.9947320000
C	5.9252580000	3.3189330000	-0.8583950000
H	5.6147490000	2.2771990000	-0.9998590000
H	6.8943650000	3.4828950000	-1.3320420000
H	5.1778140000	3.9825710000	-1.3103230000
N	1.0854260000	-1.2832670000	-0.3861290000
H	-0.1181220000	0.5204790000	0.5818920000
H	0.2694220000	-1.4273890000	0.2042380000
H	-1.3986160000	1.9307760000	-1.5092820000
C	1.4331220000	0.0041060000	-0.6198900000
S	-2.6590560000	-1.3197130000	1.1860070000
O	-2.9477520000	-2.2651960000	0.0897540000
O	-1.2220240000	-1.0411940000	1.4933760000
C	-3.3853880000	-1.9209700000	2.7230690000
C	-4.7309540000	-2.4153430000	2.7768340000
C	-2.5850970000	-1.8537550000	3.8451290000
C	-5.2138700000	-2.8345490000	4.0644790000
C	-5.6100410000	-2.5190400000	1.6642330000
C	-3.0772730000	-2.2834880000	5.0972460000
H	-1.5755130000	-1.4704090000	3.7597610000
C	-4.3630720000	-2.7578620000	5.2000540000
C	-6.5418930000	-3.3247290000	4.1848200000
H	-2.4338760000	-2.2292910000	5.9695620000
H	-4.7524540000	-3.0838210000	6.1610650000
C	-3.2590070000	1.2096290000	1.8298920000
N	-3.0528500000	1.9929430000	2.6737680000
C	-3.4667380000	0.2316360000	0.8358840000
C	-4.0556570000	0.3957820000	-0.4053590000
O	-2.2674330000	0.7800530000	-1.7354510000
O	-2.5676040000	1.0504710000	-3.1309090000
C	-2.2212250000	-0.0735260000	-3.9815340000
C	-3.0153530000	-1.3249120000	-3.5880600000
H	-2.7960880000	-1.6092370000	-2.5564250000
H	-4.0934200000	-1.1532620000	-3.6886250000
H	-2.7485190000	-2.1647610000	-4.2393670000
C	-0.7151840000	-0.3460320000	-3.9224330000
H	-0.4490220000	-1.1943220000	-4.5626080000
H	-0.1472180000	0.5263890000	-4.2633360000
H	-0.4141370000	-0.5799630000	-2.8995400000
C	-2.6392260000	0.4220520000	-5.3716340000
H	-2.4344340000	-0.3467290000	-6.1244120000
H	-3.7097600000	0.6531320000	-5.3959510000
H	-2.0845400000	1.3260900000	-5.6462920000
H	-4.0922600000	-0.5019150000	-1.0074820000
H	-2.1836260000	5.8783090000	-0.7829100000
C	-4.9459050000	1.4732750000	-0.8462510000
C	-5.2991870000	2.5875510000	-0.0580750000
C	-5.5244620000	1.3541450000	-2.1256490000
C	-6.1848790000	3.5474450000	-0.5423970000
H	-4.8986200000	2.7043300000	0.9413240000
C	-6.4075330000	2.3165320000	-2.6081150000
H	-5.2684210000	0.5003320000	-2.7411180000
C	-6.7399360000	3.4194580000	-1.8188010000
H	-6.4491120000	4.3947040000	0.0842170000
H	-6.8401840000	2.2025340000	-3.5980850000
H	-7.4328890000	4.1692800000	-2.1904580000
S	2.7588290000	0.4536430000	-1.5920360000
C	1.8943860000	-2.4577050000	-0.6933260000
H	2.8350740000	-2.0630870000	-1.0738240000
C	2.2083640000	-3.2566880000	0.6446420000
H	2.7569420000	-4.1441310000	0.3201120000
N	1.0341150000	-3.7725110000	1.3532820000
H	0.4075150000	-3.0300750000	1.6578490000
H	0.4943560000	-4.3654050000	0.7251100000
C	1.2573810000	-3.3703360000	-1.7375350000
C	2.0735320000	-4.0928790000	-2.6762680000
C	-0.1116770000	-3.5727790000	-1.7461160000
C	3.4867210000	-3.9383210000	-2.7758530000
C	1.4414920000	-5.0148490000	-3.5817400000
C	-0.7274760000	-4.4771020000	-2.6434700000
H	-0.7520500000	-3.0209270000	-1.0658350000
C	4.2272290000	-4.6542440000	-3.6918500000
H	4.0034330000	-3.2368510000	-2.1316010000
C	2.2354710000	-5.7379940000	-4.5128800000
C	0.0326580000	-5.1890600000	-3.5387350000
H	-1.8060420000	-4.6022230000	-2.6113750000
C	3.5996740000	-5.5690310000	-4.5686520000
H	5.3026720000	-4.5084020000	-3.7441870000
H	1.7378340000	-6.4317480000	-5.1865430000
H	-0.4299320000	-5.8911390000	-4.2282220000
H	4.1939700000	-6.1283480000	-5.2859040000
C	3.1015440000	-2.4328400000	1.5689520000
C	4.5353840000	-2.5099240000	1.4840550000
C	2.5370450000	-1.5641040000	2.4851720000
C	5.2297560000	-3.3868050000	0.6007840000
C	5.3302510000	-1.6688320000	2.3390560000
C	3.3216210000	-0.7400450000	3.3244940000
H	1.4578820000	-1.4906940000	2.5743450000
C	6.6067130000	-3.4246910000	0.5536130000
H	4.6752790000	-4.0504800000	-0.0522850000
C	6.7484190000	-1.7323440000	2.2643260000
C	4.6920980000	-0.7867280000	3.2497170000
H	2.8308990000	-0.0655250000	4.0202800000
C	7.3794790000	-2.5870970000	1.3907040000
H	7.1020680000	-4.1081490000	-0.1307810000
H	7.3283640000	-1.0857600000	2.9187730000
H	5.3055850000	-0.1500450000	3.8822030000
H	8.4643810000	-2.6247580000	1.3447510000
C	-7.3694830000	-3.4021670000	3.0889530000
C	-6.8935860000	-2.9960590000	1.8211620000
H	-6.8906290000	-3.6384800000	5.1652670000
H	-8.3834640000	-3.7772520000	3.1921850000
H	-7.5460800000	-3.0657610000	0.9555140000
H	-5.2647320000	-2.2397880000	0.6777580000
C	1.9037530000	4.8132030000	-2.9349550000
C	2.7159640000	5.7204600000	-2.3885300000
H	2.3746020000	6.4692350000	-1.6787930000
H	3.7710790000	5.7518760000	-2.6461910000
H	2.3269410000	4.1004870000	-3.6445470000

TS1-S_Naphtyl

Imaginary Frequency (cm-1) : -103.54

N	-0.6726880000	-0.7971120000	-0.3470780000
C	-1.0134260000	-2.1176200000	-0.8600750000
C	0.1962290000	-2.8085570000	-1.5358080000
H	-1.7840340000	-1.9499720000	-1.6091010000
C	-1.5609340000	-3.0304960000	0.2496470000
H	0.9554970000	-3.0131300000	-0.7783680000
C	-0.2397910000	-4.1152280000	-2.2610920000
N	0.9086150000	-1.9259630000	-2.5392930000
C	-2.9116040000	-3.5079610000	0.2638650000
C	-0.7311020000	-3.4077800000	1.2903900000
C	0.2852590000	-4.1188430000	-3.7089850000
H	0.1455060000	-4.9857680000	-1.7215430000
H	-1.3300220000	-4.1974270000	-2.2594900000
C	0.0452280000	-1.6142080000	-3.7283530000
C	2.1646260000	-2.6247960000	-2.9988000000
C	-3.8882480000	-3.1795090000	-0.7183200000
C	-3.2863560000	-4.3735080000	1.3480510000
C	-1.2144030000	-4.2472760000	2.3172020000
H	0.2988220000	-3.0665680000	1.3431370000
C	-0.2925860000	-2.9248870000	-4.5056270000
H	0.0196540000	-5.0607290000	-4.1978500000

C	1.8161690000	-3.9712640000	-3.6710460000
H	-0.8463050000	-1.1009940000	-3.3609040000
H	0.6142410000	-0.9046210000	-4.3280150000
H	2.7899850000	-2.7496590000	-2.1125760000
H	2.6608720000	-1.9223290000	-3.6689440000
C	-5.1683190000	-3.6945950000	-0.6347320000
H	-3.6406910000	-2.4820090000	-1.5060960000
C	-4.6111780000	-4.8922110000	1.3904140000
N	-2.4414820000	-4.7299290000	2.3557110000
H	-0.5501360000	-4.5340870000	3.1314110000
H	0.2512710000	-2.8828990000	-5.4611350000
C	-5.5288700000	-4.5695450000	0.4270030000
O	-6.1720640000	-3.4278050000	-1.5128420000
H	-4.8658180000	-5.5482940000	2.2167780000
H	-6.5430430000	-4.9558710000	0.4531570000
C	-5.9087070000	-2.5178060000	-2.5782880000
H	-5.1159640000	-2.8960630000	-3.2355670000
H	-5.6207970000	-1.5314790000	-2.1960940000
H	-6.8415990000	-2.4382110000	-3.1383380000
N	-1.0624770000	1.3382760000	0.3213920000
H	-0.0127660000	-0.7837780000	0.4257800000
H	-0.2367860000	1.1638950000	0.8882700000
H	1.2962200000	-0.9790480000	-2.1083190000
C	-1.4616880000	0.3173440000	-0.4714350000
S	2.4638280000	-0.4820230000	1.7159130000
O	1.1736240000	0.2218840000	1.9756270000
O	2.3643690000	-1.8134120000	1.0703100000
C	3.2770750000	-0.6395780000	3.3169510000
C	2.6908750000	0.0375760000	4.3668170000
C	4.4846310000	-1.3966280000	3.4803430000
C	3.2728820000	-0.0017320000	5.6530110000
H	1.7792120000	0.5983810000	4.2008980000
C	5.0646080000	-1.4118990000	4.7956690000
C	4.4345460000	-0.7072230000	5.8567170000
H	2.7972450000	0.5331900000	6.4687390000
H	4.8936270000	-0.7362980000	6.8414650000
C	3.6171870000	1.8710360000	1.1839200000
N	3.6819020000	2.9576130000	1.6102340000
C	3.5054630000	0.5458740000	0.7114490000
C	3.9706270000	0.0295960000	-0.4878570000
O	2.2013480000	0.2615610000	-1.8630430000
O	2.4573130000	0.5225110000	-3.2667310000
C	2.1234490000	1.8900470000	-3.6262070000
C	2.9695150000	2.8886520000	-2.8309770000
H	4.0355230000	2.7367530000	-3.0226660000
H	2.7076260000	3.9148550000	-3.1131630000
H	2.7873160000	2.7692190000	-1.7606600000
C	0.6293180000	2.1489060000	-3.4062420000
H	0.0183630000	1.4770580000	-4.0191440000
H	0.3703890000	1.9937880000	-2.3568220000
H	0.3724430000	3.1791570000	-3.6769840000
C	2.4826780000	1.9393050000	-5.1164880000
H	3.5432620000	1.7096550000	-5.2651050000
H	1.8877010000	1.2180280000	-5.6884080000
H	2.2855240000	2.9384580000	-5.5196360000
H	3.7337090000	-1.0134740000	-0.6451050000
H	2.2567890000	-4.8031050000	-3.1116250000
C	5.0508660000	0.5509310000	-1.3273470000
C	5.3512730000	-0.1401990000	-2.5170340000
C	5.8444580000	1.6623640000	-0.9837410000
C	6.3977660000	0.2665300000	-3.3398880000
H	4.7519040000	-1.0005980000	-2.7913560000
C	6.8921860000	2.0672680000	-1.8073210000
H	5.6570660000	2.2077040000	-0.0668520000
C	7.1715470000	1.3754570000	-2.9890700000
H	6.6117150000	-0.2823180000	-4.2528320000
H	7.4967040000	2.9232990000	-1.5211230000
H	7.9909530000	1.6941410000	-3.6275250000
S	-2.7714180000	0.4160680000	-1.5547780000
C	-1.8059000000	2.5623150000	0.5959840000
H	-2.7316860000	2.4659250000	0.0315840000
C	-2.1780740000	2.6225450000	2.1413770000
H	-2.6773840000	3.5850510000	2.2789910000
N	-1.0311490000	2.6524640000	3.0531030000
H	-0.4249950000	1.8429120000	2.9345910000
H	-0.4587540000	3.4689020000	2.8460440000
C	-1.0571810000	3.8191580000	0.1626740000
C	-1.7634360000	4.9543280000	-0.3670070000
C	0.3112960000	3.9112290000	0.3481300000
C	-3.1646680000	4.9617040000	-0.6266110000
C	-1.0251320000	6.1514960000	-0.6701770000
C	1.0333820000	5.0890340000	0.0455380000
H	0.8708800000	3.0631340000	0.7280970000
C	-3.7974520000	6.0759720000	-1.1342970000
H	-3.7569860000	4.0741590000	-0.4374340000
C	-1.7100550000	7.2827170000	-1.1915640000
C	0.3773110000	6.1899740000	-0.4484240000
H	2.1052170000	5.1060610000	0.2189410000
C	-3.0669260000	7.2533460000	-1.4167990000
H	-4.8667280000	6.0460550000	-1.3250500000
H	-1.1339000000	8.1784470000	-1.4116540000
H	0.9190790000	7.1040890000	-0.6790320000
H	-3.5768270000	8.1257960000	-1.8159940000
C	-3.1511140000	1.5033050000	2.5032440000
C	-4.5713100000	1.6763470000	2.3574170000
C	-2.6728010000	0.2836850000	2.9460860000
C	-5.1793150000	2.8955120000	1.9393860000
C	-5.4428040000	0.5717440000	2.6569590000
C	-3.5327380000	-0.8000210000	3.2389340000
H	-1.6064060000	0.1307730000	3.0759540000
C	-6.5465990000	3.0155820000	1.8135560000
H	-4.5647200000	3.7602260000	1.7175290000
C	-6.8485190000	0.7291270000	2.5159020000
C	-4.8912450000	-0.6616030000	3.0924750000
H	-3.1083520000	-1.7425400000	3.5731740000
C	-7.3949430000	1.9213020000	2.1006320000
H	-6.9758610000	3.9616330000	1.4948070000
H	-7.4879690000	-0.1199370000	2.7460180000
H	-5.5611930000	-1.4907430000	3.3060050000
H	-8.4714220000	2.0268300000	1.9975460000
C	5.1402080000	-2.1244540000	2.4501440000
C	6.2658140000	-2.1368010000	5.0192000000
C	6.8800040000	-2.8215730000	3.9965900000
C	6.3069650000	-2.8127890000	2.7042920000
H	4.7121380000	-2.1549640000	1.4572160000
H	6.7896840000	-3.3601110000	1.8996460000
H	7.7991200000	-3.3709400000	4.1779680000
H	6.6903090000	-2.1382770000	6.0197440000
C	-1.7625170000	-2.9946250000	-4.8584740000
C	-2.5563440000	-4.0667030000	-4.8190510000
H	-2.1814600000	-2.0533010000	-5.2177480000
H	-3.5928170000	-4.0051560000	-5.1392110000
H	-2.2174440000	-5.0426010000	-4.4819100000
H	2.2300050000	-4.0066710000	-4.6843140000

TS2-RR_Naphtyl

Imaginary Frequency (cm-1) : -265.13

N	-0.5327850000	0.8931860000	0.1744040000
C	-0.8631780000	2.2622840000	-0.2148070000
C	0.2617510000	3.2775320000	0.0960590000
H	-1.7365680000	2.5259500000	0.3770450000
C	-1.2096480000	2.3602730000	-1.7109130000
H	1.1562030000	3.0155030000	-0.4733480000
C	-0.2008600000	4.7273120000	-0.2388030000
N	0.7307910000	3.2711430000	1.5396850000
C	-2.5243670000	2.6865690000	-2.1788540000
C	-0.2238970000	2.1408190000	-2.6556720000
C	0.1756010000	5.6873210000	0.9025350000
H	0.2665900000	5.0526320000	-1.1724260000
H	-1.2816400000	4.7476720000	-0.4096480000
C	-0.3251330000	3.7940430000	2.4765880000
C	1.9772580000	4.1267390000	1.6497900000
C	-3.6506910000	2.8909100000	-1.3314690000
C	-2.6973170000	2.8135810000	-3.5994650000
C	-0.5160480000	2.2613410000	-4.0315270000
H	0.7927270000	1.8864720000	-2.3717700000
C	-0.5732480000	5.3078490000	2.2126220000
H	-0.0798050000	6.7137220000	0.6230460000
C	1.6854240000	5.5594970000	1.1594250000
H	-1.2210660000	3.1851740000	2.3341920000
H	0.0393440000	3.6083760000	3.4880600000
H	2.7484520000	3.6169290000	1.0730570000
H	2.2679020000	4.0796740000	2.6988580000
C	-4.8808440000	3.2239840000	-1.8679740000
H	-3.5572160000	2.7220110000	-0.2671960000
C	-3.9757180000	3.1765620000	-4.1094930000
N	-1.7007370000	2.5986290000	-4.5034410000
H	0.2721120000	2.0859820000	-4.7621870000
H	-0.1145170000	5.8837030000	3.0254860000
H	2.0126800000	6.2862040000	1.9101620000
C	-5.0391390000	3.3834670000	-3.2724990000
O	-6.0173290000	3.4181690000	-1.1473360000
H	-4.0754590000	3.2767910000	-5.1856640000
H	-6.0193550000	3.6550660000	-3.6517710000
C	-5.9648210000	3.2197400000	0.2631980000
H	-5.6499760000	2.1994350000	0.5102450000
H	-6.9796410000	3.3907830000	0.6251420000
H	-5.2818270000	3.9346000000	0.7394920000
N	-1.1828780000	-1.2747780000	0.4865730000
H	0.2032270000	0.4500130000	-0.3736180000
H	-0.3590570000	-1.4682750000	-0.0780630000
H	1.0624030000	2.3182320000	1.8984080000
C	-1.4636650000	0.0286760000	0.7050570000
S	2.6081760000	-1.4701460000	-0.9455090000
O	2.7808840000	-2.3969130000	0.1892470000
O	1.2101930000	-1.1333440000	-1.3500020000
C	3.4125170000	-2.1225760000	-2.4198450000
C	4.7313110000	-2.6858250000	-2.3726770000
C	2.7030900000	-2.0166850000	-3.5992850000
C	5.2887830000	-3.1287800000	-3.6215350000
C	3.2662150000	-2.4725390000	-4.8112140000
H	1.7107870000	-1.5821040000	-3.5894500000
C	4.5313250000	-3.0100110000	-4.8178770000
H	2.6941310000	-2.3875260000	-5.7294940000
H	4.9759380000	-3.3547170000	-5.7478240000

C	3.4726270000	1.0348030000	-1.5585290000
N	3.4427090000	1.8536770000	-2.3935030000
C	3.4770040000	0.0351740000	-0.5703880000
C	4.0130210000	0.2490410000	0.7977930000
O	2.6993640000	0.7254280000	1.0065970000
O	2.2024100000	1.4701260000	2.8071710000
C	2.0385780000	0.5366050000	3.8523530000
C	1.2144820000	-0.6796540000	3.4057400000
H	1.0732440000	-1.3900110000	4.2290760000
H	0.2258840000	-0.3661560000	3.0551170000
H	1.7183680000	-1.1977280000	2.5855530000
C	1.2873540000	1.2752040000	4.9869210000
H	1.8334380000	2.1784470000	5.2832650000
H	0.2820750000	1.5626860000	4.6593450000
H	1.1825820000	0.6322220000	5.8696590000
C	3.4119520000	0.0779530000	4.3841410000
H	3.9736500000	-0.4530310000	3.6082340000
H	4.0077400000	0.9399730000	4.7048980000
H	3.3018000000	-0.6003820000	5.2390250000
H	4.1881650000	-0.6968680000	1.3224400000
H	2.2401990000	5.7705490000	0.2391760000
C	5.1769980000	1.2053580000	0.9478870000
C	5.1462300000	2.2228070000	1.9082050000
C	6.3267580000	1.0275040000	0.1670450000
C	6.2519210000	3.0611660000	2.0721550000
H	4.2562250000	2.3288650000	2.5200460000
C	7.4292670000	1.8658370000	0.3355310000
H	6.3590480000	0.2348190000	-0.5763760000
C	7.3936320000	2.8875740000	1.2876690000
H	6.2215100000	3.8504100000	2.8192150000
H	8.3131940000	1.7235200000	-0.2803280000
H	8.2513450000	3.5422940000	1.4169740000
S	-2.8054010000	0.5663880000	1.6075270000
C	-2.0477580000	-2.4068760000	0.8072180000
H	-2.9894530000	-1.9620050000	1.1231620000
C	-2.3295000000	-3.2535540000	-0.5083270000
H	-2.9168320000	-4.1099320000	-0.1676800000
N	-1.1449320000	-3.8343600000	-1.1448810000
H	-0.4805940000	-3.1271680000	-1.4530060000
H	-0.6534050000	-4.4222590000	-0.4743390000
C	-1.4977200000	-3.2991920000	1.9167450000
C	-2.3806100000	-3.9147920000	2.8714130000
C	-0.1467840000	-3.5954280000	1.9658660000
C	-3.7806330000	-3.6558050000	2.9358310000
C	-1.8319660000	-4.8347450000	3.8319260000
C	0.3870870000	-4.4945570000	2.9188580000
H	0.5433470000	-3.1210160000	1.2760810000
C	-4.5860100000	-4.2735250000	3.8684290000
H	-4.2350430000	-2.9492440000	2.2515540000
C	-2.6916170000	-5.4554530000	4.7785530000
C	-0.4385010000	-5.1087020000	3.8282250000
H	1.4550610000	-4.6934500000	2.9191030000
C	-4.0410020000	-5.1884880000	4.7985260000
H	-5.6488010000	-4.0487950000	3.8925840000
H	-2.2562550000	-6.1495290000	5.4937020000
H	-0.0397140000	-5.8066850000	4.5603650000
H	-4.6860030000	-5.6699200000	5.5283600000
C	-3.1624260000	-2.4534790000	-1.5065870000
C	-4.5997340000	-2.4643070000	-1.4526260000
C	-2.5400400000	-1.6810960000	-2.4698160000
C	-5.3500010000	-3.2412350000	-0.5228950000
C	-5.3383930000	-1.6607510000	-2.3897620000
C	-3.2696430000	-0.8956770000	-3.3916780000
H	-1.4570620000	-1.6596270000	-2.5364970000
C	-6.7280550000	-3.2191720000	-0.5081040000
H	-4.8389150000	-3.8737640000	0.1934150000
C	-6.7593010000	-1.6603890000	-2.3467530000
C	-4.6422900000	-0.8810690000	-3.3501140000
H	-2.7346530000	-0.3004430000	-4.1257980000
C	-7.4454800000	-2.4182400000	-1.4264040000
H	-7.2672210000	-3.8266060000	0.2138190000
H	-7.2965010000	-1.0440000000	-3.0638330000
H	-5.2131640000	-0.2731740000	-4.0473860000
H	-8.5317610000	-2.4081730000	-1.4055780000
C	-2.0432160000	5.6390780000	2.1944930000
C	-2.6257140000	6.5158840000	3.0134450000
H	-2.0608880000	7.0541140000	3.7722460000
H	-3.6896720000	6.7287380000	2.9575420000
H	-2.6537800000	5.1173160000	1.4567330000
C	5.5162770000	-2.8363240000	-1.1964690000
C	6.5965070000	-3.6836740000	-3.6429420000
C	7.3343600000	-3.8019210000	-2.4881840000
C	6.7837330000	-3.3754830000	-1.2579350000
H	7.0024950000	-4.0139590000	-4.5954820000
H	8.3336930000	-4.2260890000	-2.5162060000
H	7.3627810000	-3.4811200000	-0.3450480000
H	5.1072200000	-2.5501150000	-0.2363450000

TS2_SS_Naphtyl
Imaginary Frequency (cm-1) : -303.43

N	-0.6103750000	-0.7827640000	-0.2253170000
C	-0.9340630000	-2.2045430000	-0.2440190000
C	0.2943390000	-3.0496980000	-0.6567940000
H	-1.7173090000	-2.3169430000	-0.9901480000
C	-1.4528360000	-2.7058600000	1.1164900000
H	1.1178030000	-2.8208860000	0.0247720000
C	-0.0195040000	-4.5743700000	-0.6469670000
N	0.8454590000	-2.7108400000	-2.0357280000
C	-2.7426280000	-3.3079020000	1.2801170000
C	-0.6431400000	-2.5987680000	2.2327080000
C	0.5983380000	-5.2579010000	-1.8788950000
H	0.3764650000	-5.0180910000	0.2707040000
H	-1.1009920000	-4.7379370000	-0.6280320000
C	-0.0708620000	-3.1934950000	-3.1301220000
C	2.2014810000	-3.3678640000	-2.1941090000
C	-3.7020220000	-3.4493430000	0.2365210000
C	-3.0723750000	-3.7969870000	2.5902880000
C	-1.0816040000	-3.0944900000	3.4793100000
H	0.3359580000	-2.1335770000	2.1750270000
C	-0.0843920000	-4.7476350000	-3.1789260000
H	0.4772460000	-6.3423820000	-1.8027640000
C	2.0887510000	-4.8861030000	-1.9457470000
H	-1.0591580000	-2.7704960000	-2.9399690000
H	0.2989710000	-2.7588930000	-4.0585460000
H	2.8723980000	-2.8696080000	-1.4938510000
H	2.5326900000	-3.1148880000	-3.2009490000
C	-4.9192550000	-4.0580640000	0.4791600000
H	-3.5029200000	-3.0162470000	-0.7337260000
C	-4.3299640000	-4.4327720000	2.7936010000
N	-2.2454290000	-3.6869040000	3.6670070000
H	-0.4326170000	-3.0048550000	4.3495030000
H	0.5324740000	-5.0759020000	-4.0242730000
H	2.5893380000	-5.4329580000	-2.7512080000
C	-5.2287550000	-4.5684230000	1.7703140000
O	-5.9073160000	-4.2182000000	-0.4417010000
H	-4.5498820000	-4.7993720000	3.7912580000
H	-6.1929870000	-5.0449140000	1.9171550000
C	-5.7126600000	-3.6747430000	-1.7441410000
H	-4.8693930000	-4.1572160000	-2.2548630000
H	-5.5400750000	-2.5930220000	-1.7027620000
H	-6.6331720000	-3.8796410000	-2.2925520000
N	-1.1826000000	1.4180480000	-0.0126030000
H	0.1663750000	-0.5369290000	0.3833250000
H	-0.3140750000	1.4764050000	0.5154000000
H	1.0583960000	-1.6692140000	-2.2156270000
C	-1.5307100000	0.2043180000	-0.4924940000
S	2.4394390000	0.6658680000	1.6292010000
O	1.3105930000	1.6309280000	1.6782650000
O	2.0794830000	-0.7708260000	1.5367390000
C	3.8540150000	2.3909300000	0.0944050000
N	4.1504810000	3.5181580000	0.0084860000
C	3.4767390000	1.0427440000	0.2430270000
C	3.7968810000	-0.0294640000	-0.7327550000
O	2.6041300000	0.4604070000	-1.3067920000
O	2.0393190000	-0.5262710000	-2.9698610000
C	1.6165970000	0.4286110000	-3.9291410000
C	0.2851810000	1.0756690000	-3.5170240000
H	-0.0628960000	1.7939620000	-4.2698070000
H	-0.4956450000	0.3177190000	-3.3883750000
H	0.3997640000	1.6082710000	-2.5694570000
C	1.4324750000	-0.3241790000	-5.2694000000
H	2.3519140000	-0.8545960000	-5.5421590000
H	0.6139830000	-1.0504360000	-5.2104670000
H	1.1890480000	0.3779060000	-6.0764450000
C	2.6805750000	1.5255150000	-4.1370190000
H	2.8755710000	2.0490110000	-3.1984800000
H	3.6240230000	1.0970210000	-4.4904240000
H	2.3384330000	2.2557630000	-4.8803050000
H	3.6499400000	-1.0262450000	-0.2991170000
H	2.5848900000	-5.1616250000	-1.0090000000
C	5.1080250000	0.0337650000	-1.4876270000
C	5.1963380000	-0.4933080000	-2.7829490000
C	6.2681870000	0.5015300000	-0.8584300000
C	6.4251980000	-0.5295310000	-3.4429580000
H	4.2884610000	-0.8472780000	-3.2596250000
C	7.4972330000	0.4616990000	-1.5207610000
H	6.2165220000	0.8962390000	0.1521690000
C	7.5788210000	-0.0518900000	-2.8157010000
H	6.4819410000	-0.9317210000	-4.4513190000
H	8.3880490000	0.8353560000	-1.0232030000
H	8.5343480000	-0.0803490000	-3.3326320000
S	-2.9449800000	-0.0931880000	-1.3918010000
C	-1.9744840000	2.6418050000	-0.1123920000
H	-2.8942530000	2.3384620000	-0.6095390000
C	-2.3615000000	3.1712250000	1.3347190000
H	-2.9273680000	4.0887860000	1.1545690000
N	-1.2321510000	3.5781050000	2.1752420000
H	-0.5637710000	2.8219190000	2.3086050000
H	-0.7197360000	4.3213290000	1.7035310000
C	-1.2776300000	3.7312690000	-0.9245340000
C	-2.0352960000	4.6189720000	-1.7648720000
C	0.0880930000	3.9270030000	-0.8068370000
C	-3.4389300000	4.4972190000	-1.9797030000
C	-1.3499090000	5.6870950000	-2.4429100000
C	0.7587980000	4.9724300000	-1.4837530000

H	0.6841840000	3.2616560000	-0.1915070000
C	-4.1226070000	5.3759520000	-2.7921200000
H	-3.9927030000	3.6927390000	-1.5100410000
C	-2.0864760000	6.5756150000	-3.2728260000
C	0.0528100000	5.8399090000	-2.2808080000
H	1.8330160000	5.0760890000	-1.3590410000
C	-3.4438270000	6.4313380000	-3.4441570000
H	-5.1922150000	5.2515800000	-2.9374470000
H	-1.5495220000	7.3772570000	-3.7745730000
H	0.5550200000	6.6514590000	-2.8017430000
H	-3.9934930000	7.1182190000	-4.0817980000
C	-3.2613890000	2.1707860000	2.0549220000
C	-4.6910110000	2.2096720000	1.9049280000
C	-2.7032270000	1.1836180000	2.8460070000
C	-5.3786640000	3.1952950000	1.1390540000
C	-5.4888990000	1.2129480000	2.5678150000
C	-3.4904870000	0.2047840000	3.4949600000
H	-1.6274970000	1.1416410000	2.9831520000
C	-6.7521220000	3.1937790000	1.0245530000
H	-4.8216620000	3.9754760000	0.6334010000
C	-6.9033440000	1.2398670000	2.4285350000
C	-4.8568710000	0.2157800000	3.3558050000
H	-3.0052000000	-0.5578090000	4.0974650000
C	-7.5276350000	2.2045900000	1.6723870000
H	-7.2429230000	3.9626800000	0.4339740000
H	-7.4860610000	0.4754790000	2.9374390000
H	-5.4721460000	-0.5353060000	3.8450110000
H	-8.6097950000	2.2126660000	1.5750980000
C	3.4293560000	0.9637500000	3.1036760000
C	3.1019920000	2.0922050000	3.8289070000
C	4.5221130000	0.1109210000	3.4745060000
C	3.8447050000	2.4385930000	4.9785900000
H	2.2705810000	2.7103500000	3.5124580000
C	5.2708600000	0.4923230000	4.6411170000
C	4.9062080000	1.6573030000	5.3684260000
H	3.5719580000	3.3259000000	5.5406520000
H	5.4890720000	1.9222890000	6.2466320000
C	4.9123550000	-1.0689900000	2.7845510000
C	6.3715490000	-0.3052630000	5.0541290000
C	6.7301120000	-1.4341630000	4.3548040000
C	5.9892730000	-1.8152750000	3.2129450000
H	4.3449600000	-1.4015040000	1.9254690000
H	6.2677580000	-2.7121500000	2.6671020000
H	7.5747960000	-2.0352870000	4.6780150000
H	6.9263530000	-0.0024670000	5.9382490000
C	-1.4751900000	-5.2861960000	-3.3938090000
C	-1.8388460000	-6.0317840000	-4.4381430000
H	-2.8535280000	-6.4047430000	-4.5463200000
H	-1.1360820000	-6.2977300000	-5.2253970000
H	-2.2180110000	-5.0391500000	-2.6344710000
